# Supplementary material for: Unraveling the Ancient Introgression History of Xanthoceras (Sapindaceae): Insights from Phylogenomic Analysis
Source: Int J Mol Sci. 2025 Feb 13;26(4):1581. doi: 10.3390/ijms26041581 (PMC11855356; doi:10.3390/ijms26041581)
Supplement: Supplementary file 1 [file ijms-26-01581-s001.zip › Figure_S10.pdf]

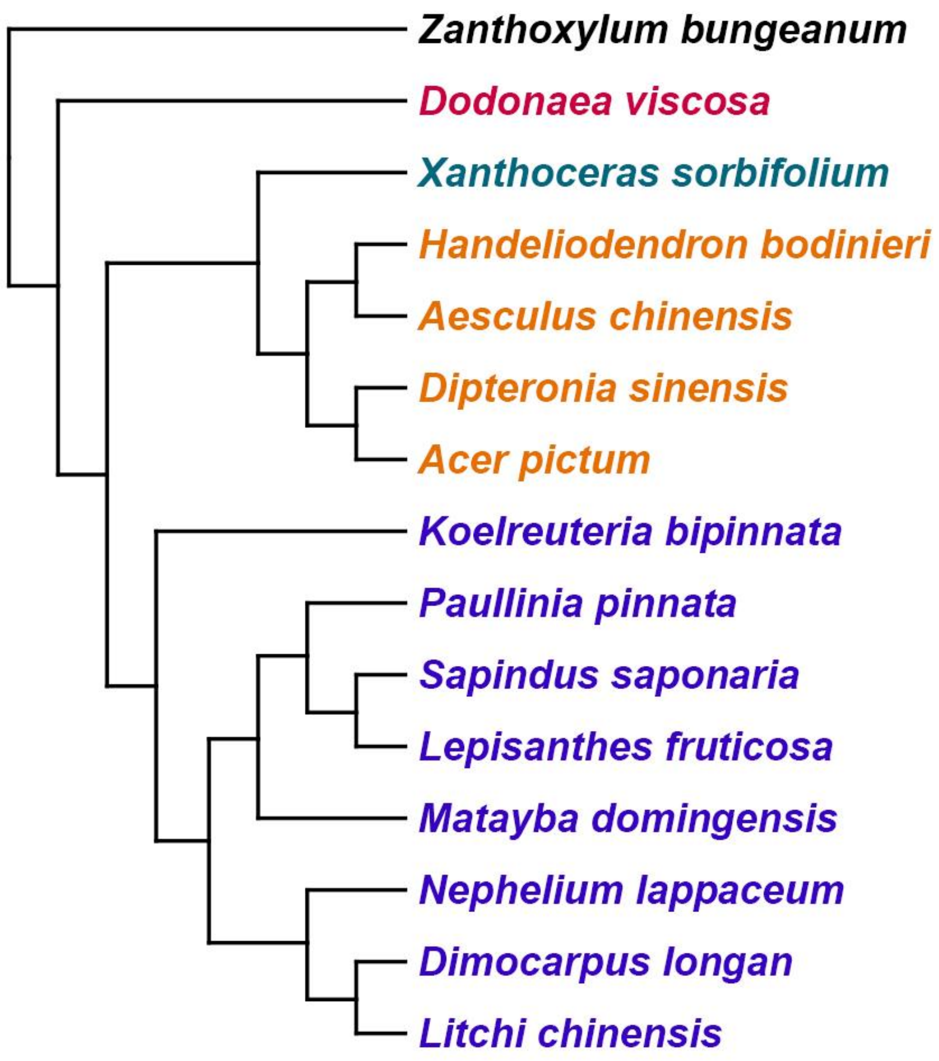

Max allowed hybrids: 0 times.

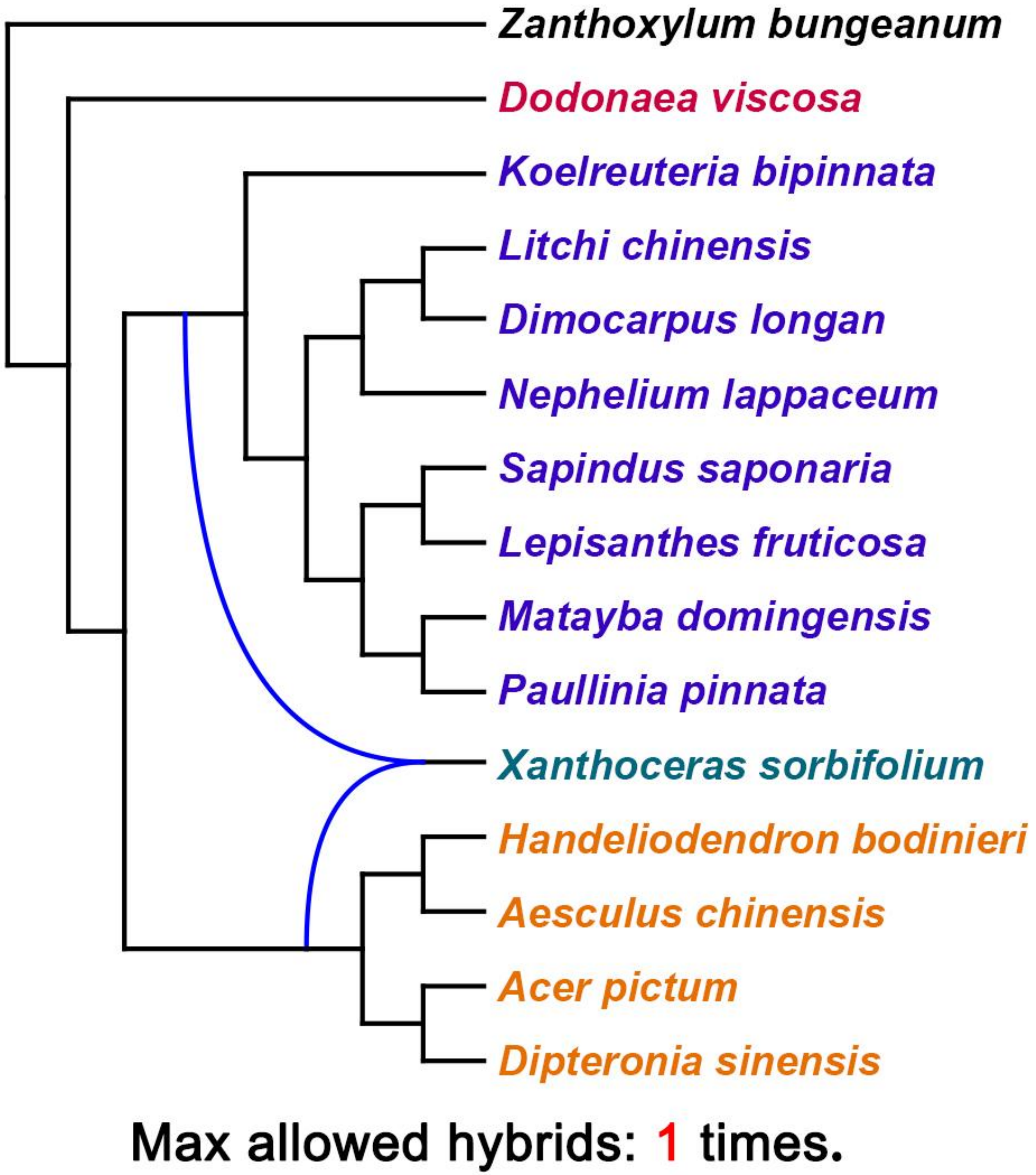

Zanthoxylum bungeanum
Dodonaea viscosa
Koelreuteria bipinnata
Litchi chinensis
Dimocarpus longan
Nephelium lappaceum
Sapindus saponaria
Lepisanthes fruticosa
Matayba domingensis
Paullinia pinnata
Xanthoceras sorbifolium
Handeliodendron bodinieri
Aesculus chinensis
Acer pictum
Dipteronia sinensis
Max allowed hybrids: 1 times.

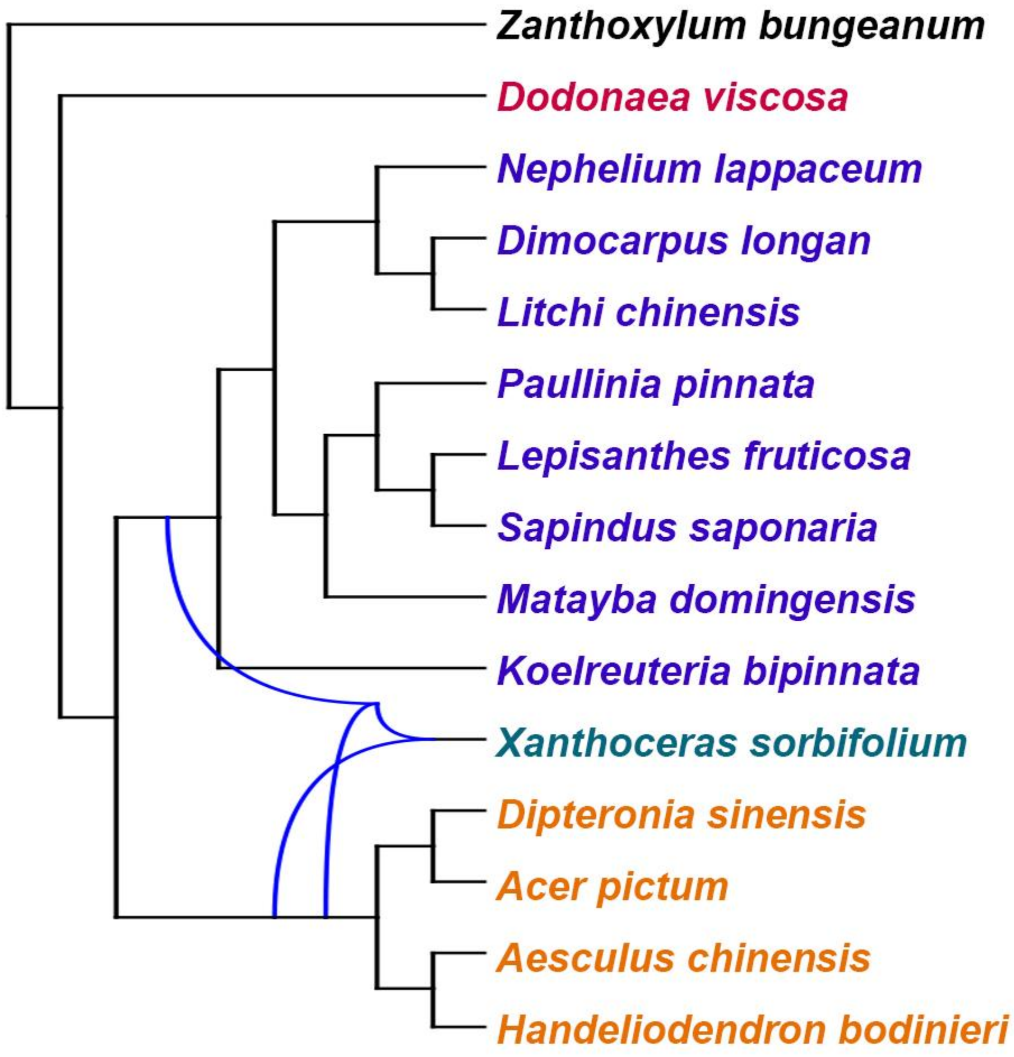

Max allowed hybrids: 2 times.

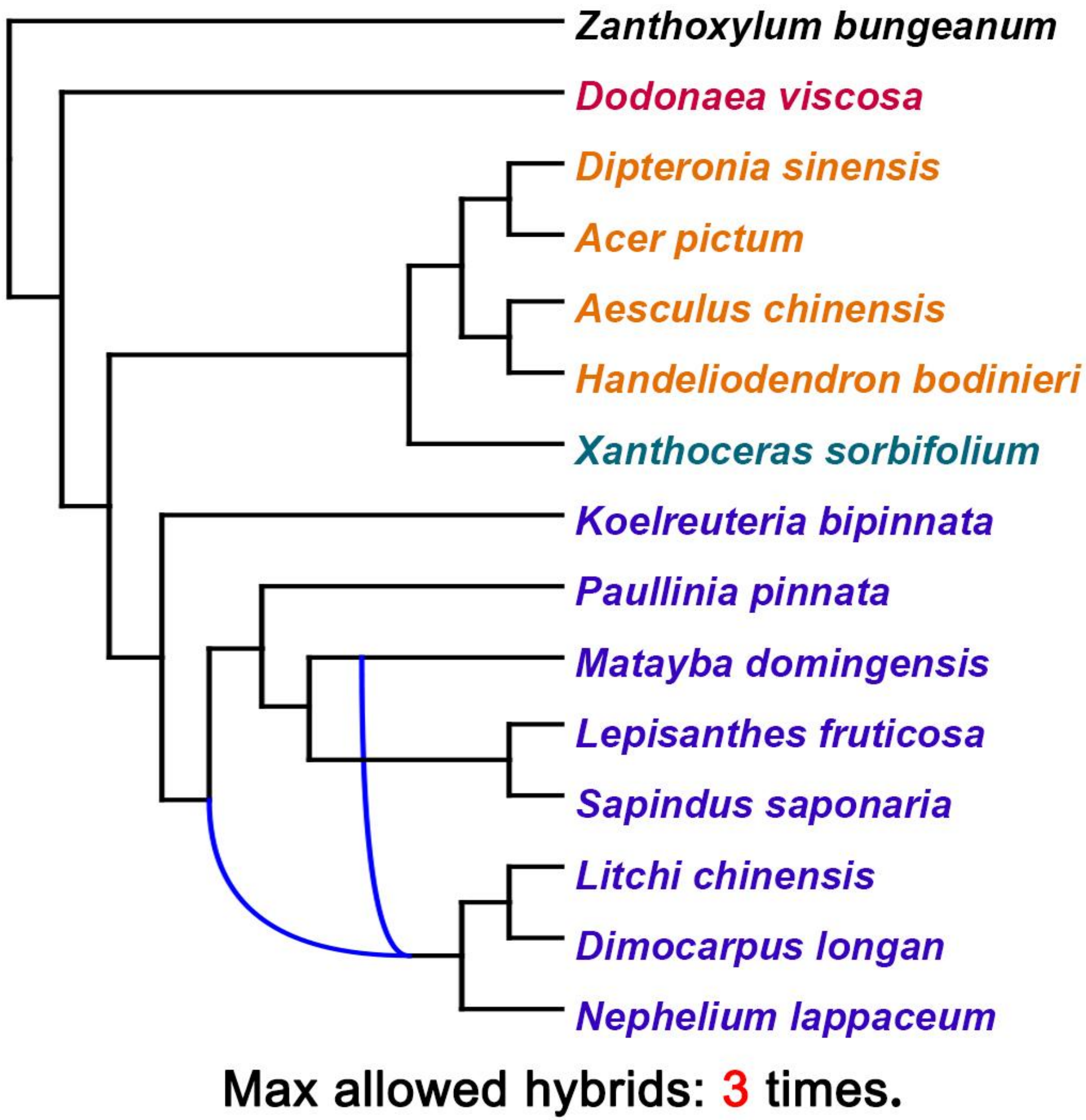
Zanthoxylum bungeanum
Dodonaea viscosa
Dipteronia sinensis
Acer pictum
Aesculus chinensis
Handeliodendron bodinieri
Xanthoceras sorbifolium
Koelreuteria bipinnata
Paullinia pinnata
Matayba domingensis
Lepisanthes fruticosa
Sapindus saponaria
Litchi chinensis
Dimocarpus longan
Nephelium lappaceum
Max allowed hybrids: 3 times.

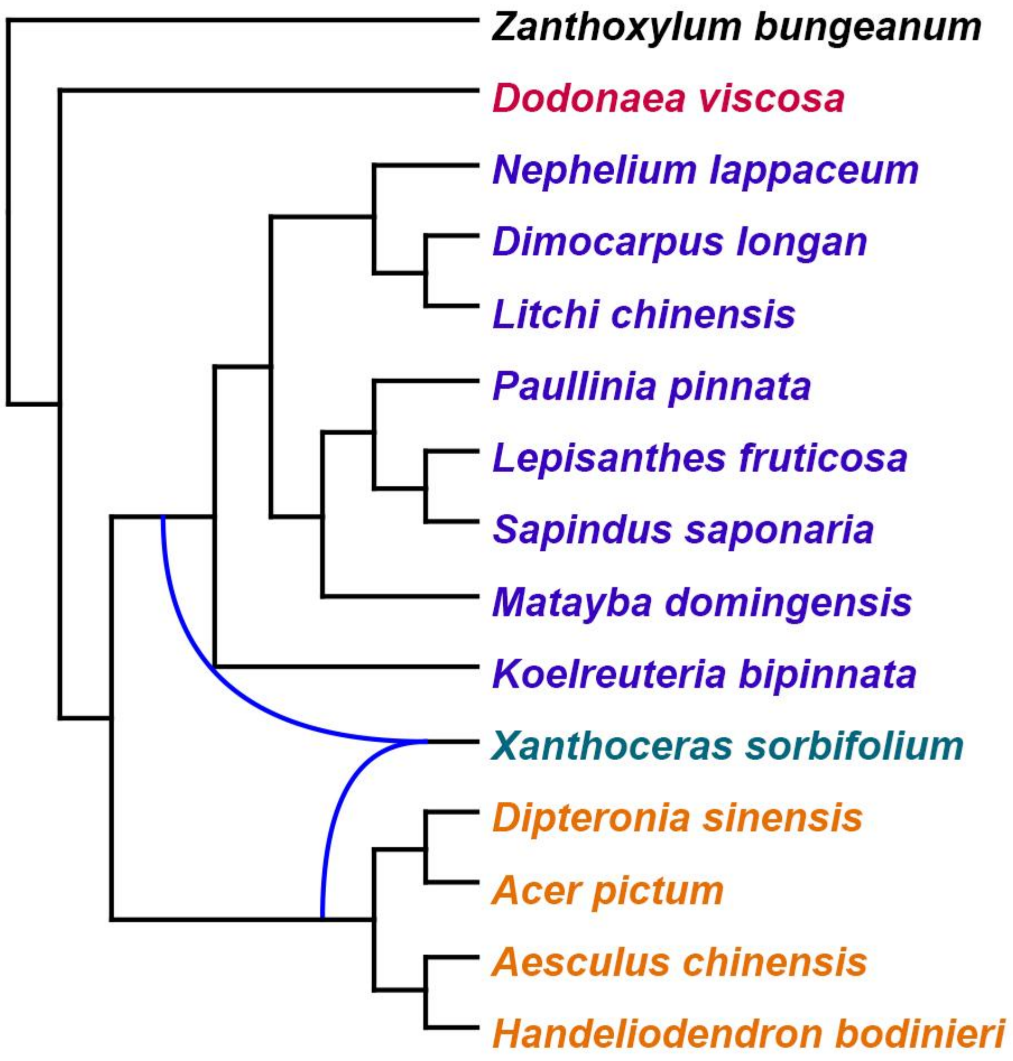

Max allowed hybrids: 4 times.

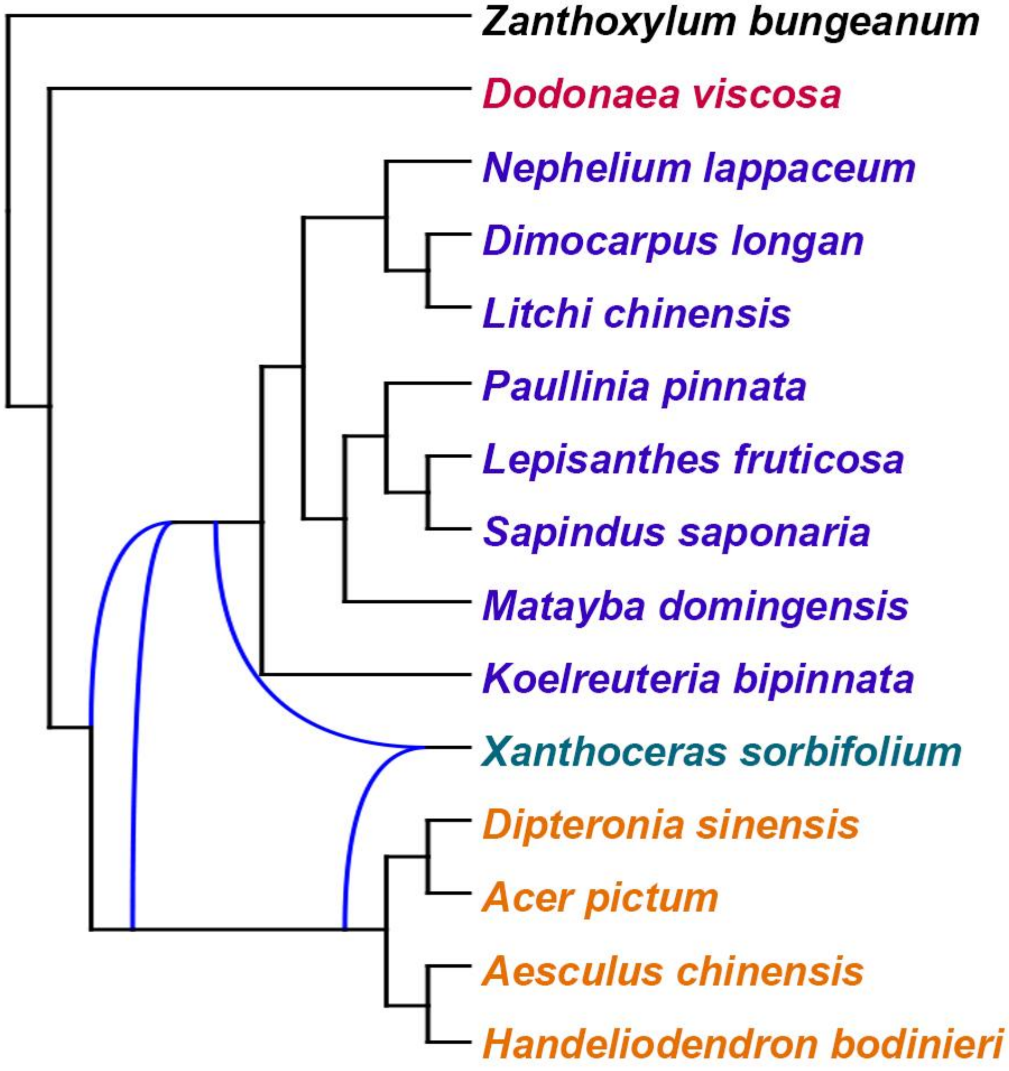

Max allowed hybrids: 5 times.

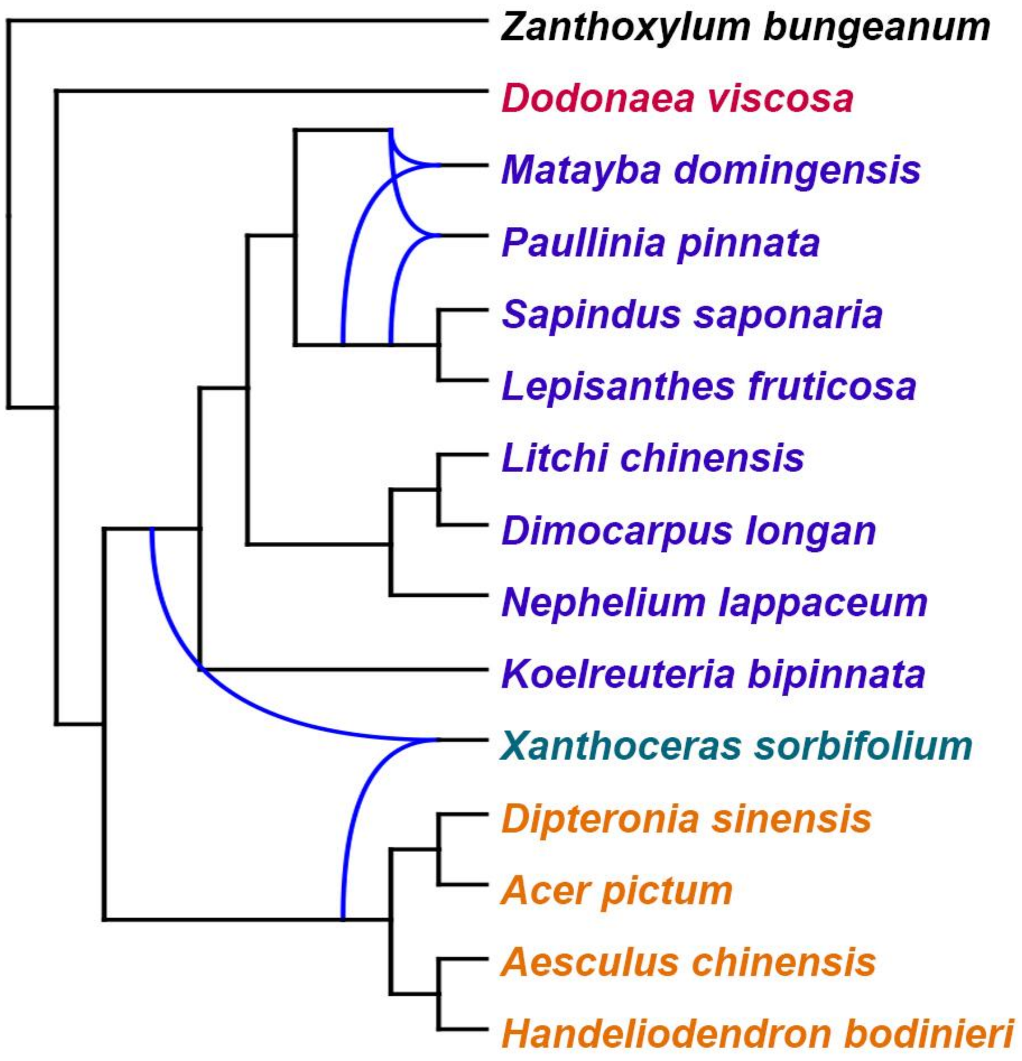

Max allowed hybrids: 6 times.

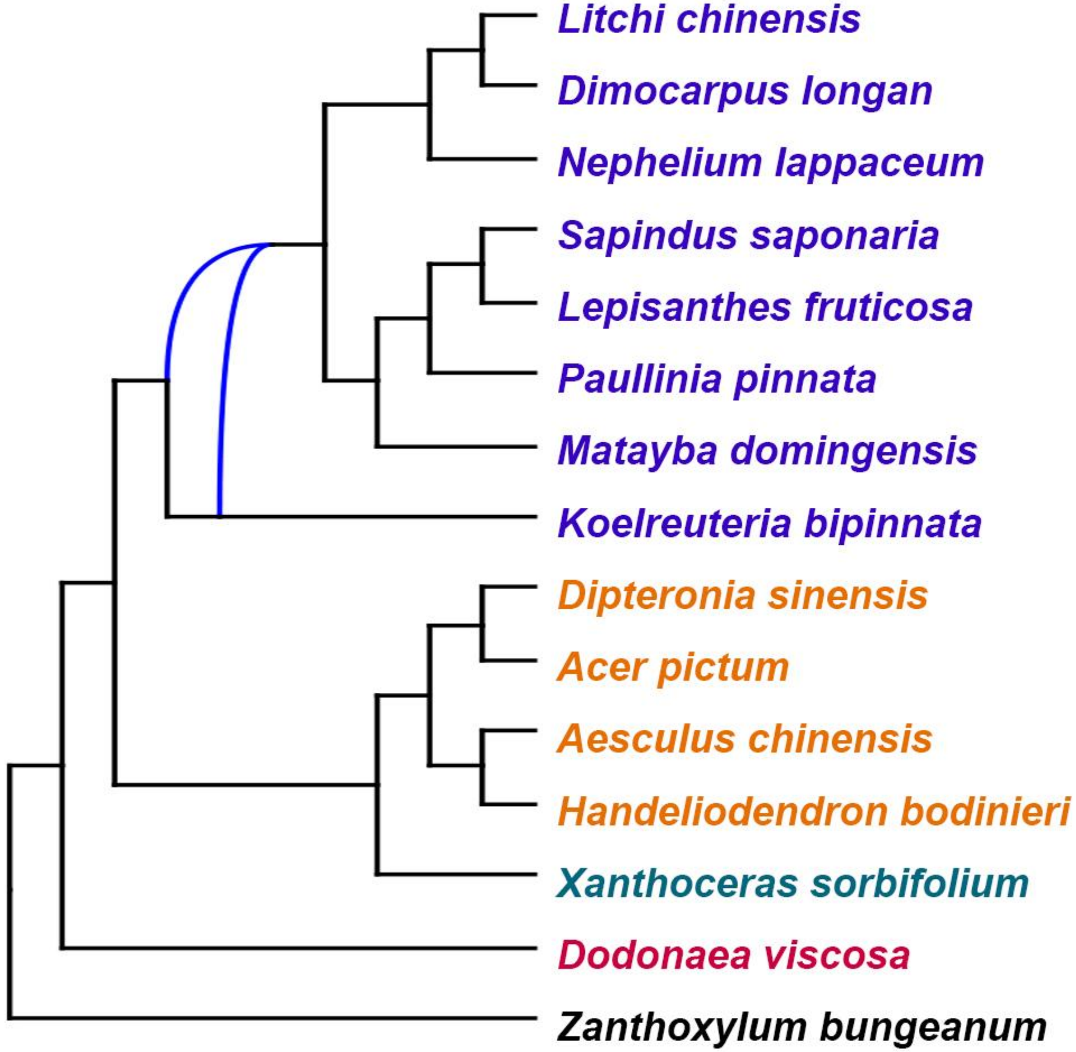

Max allowed hybrids: 7 times.

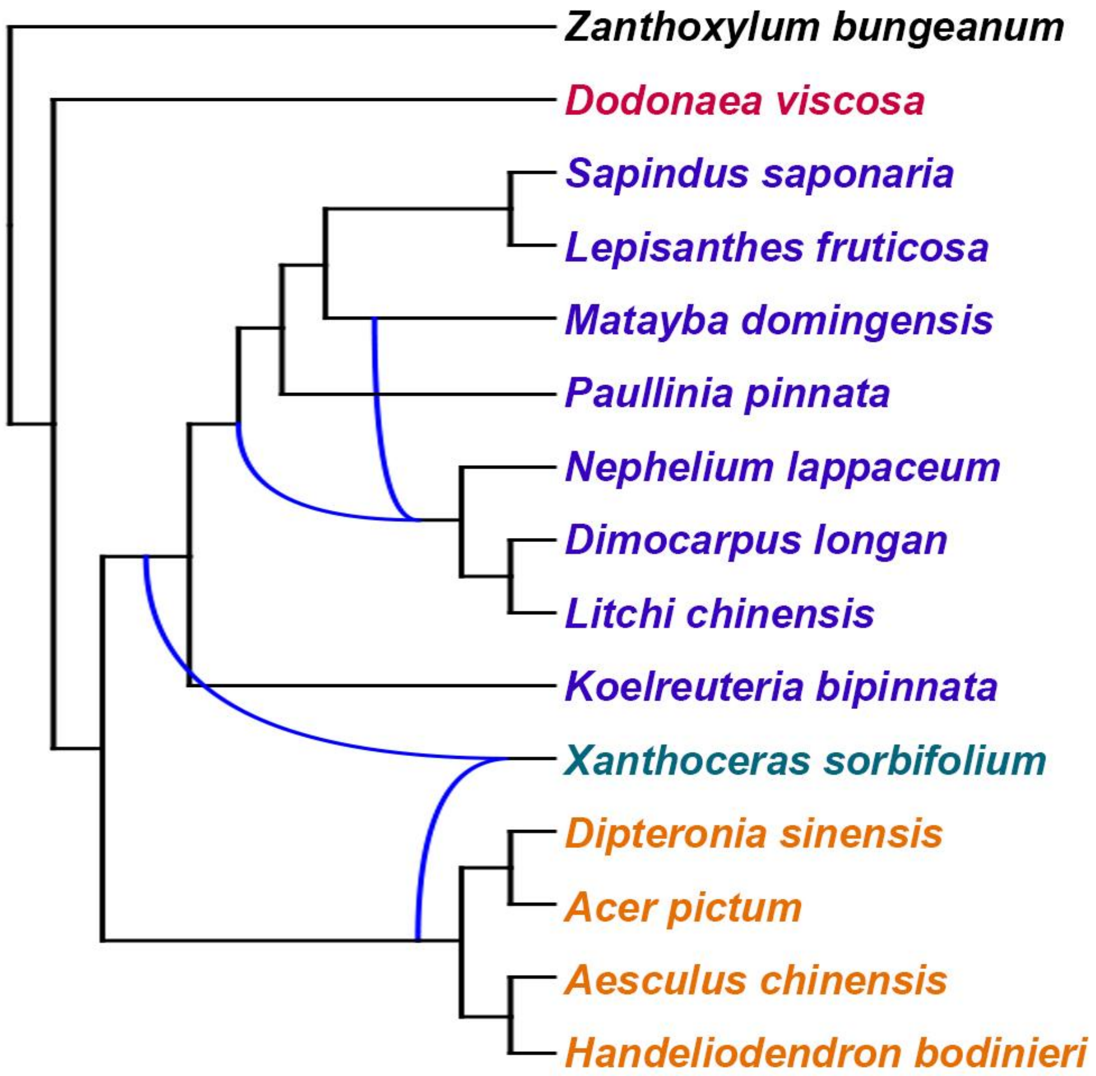

Max allowed hybrids: 8 times.

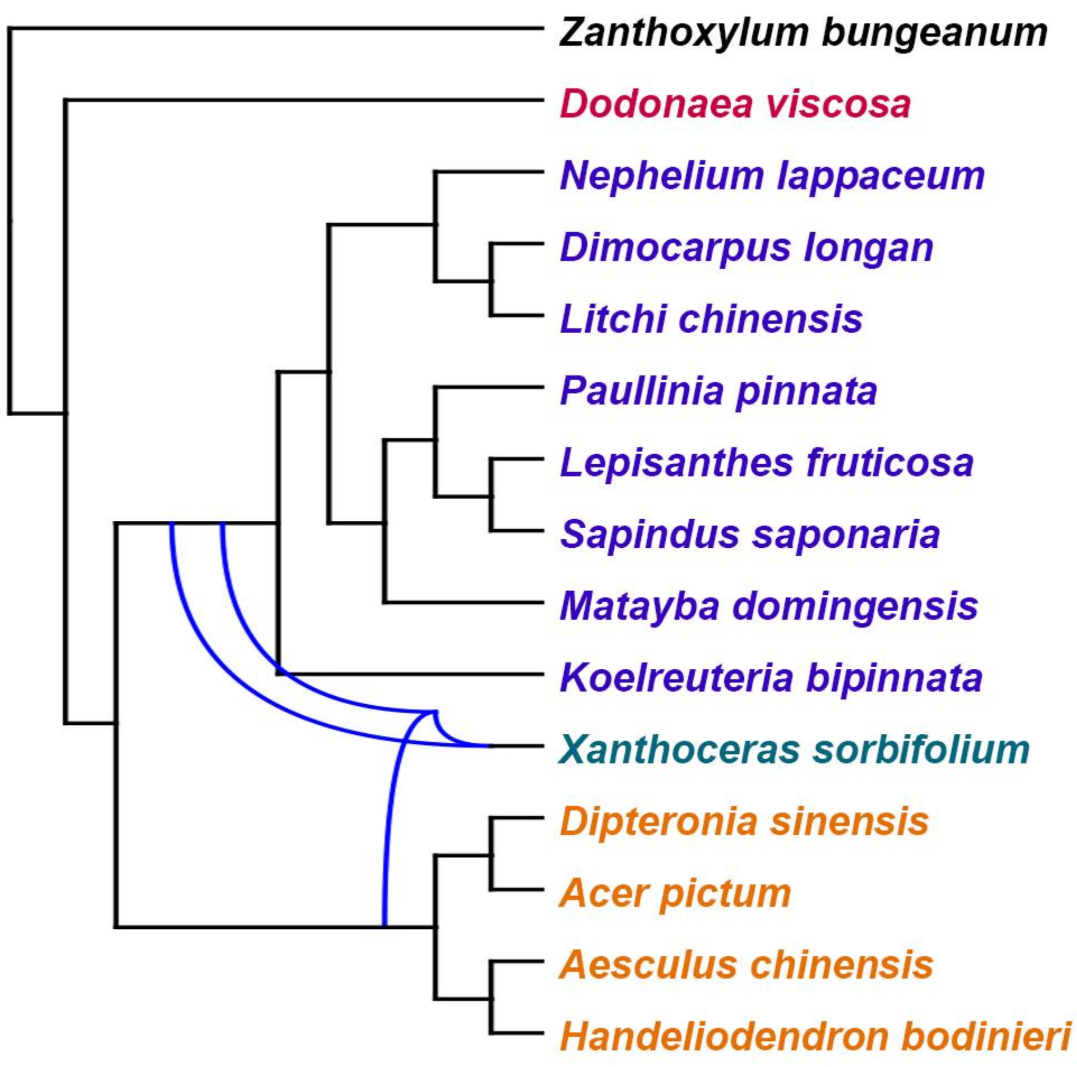

Max allowed hybrids: 9 times.

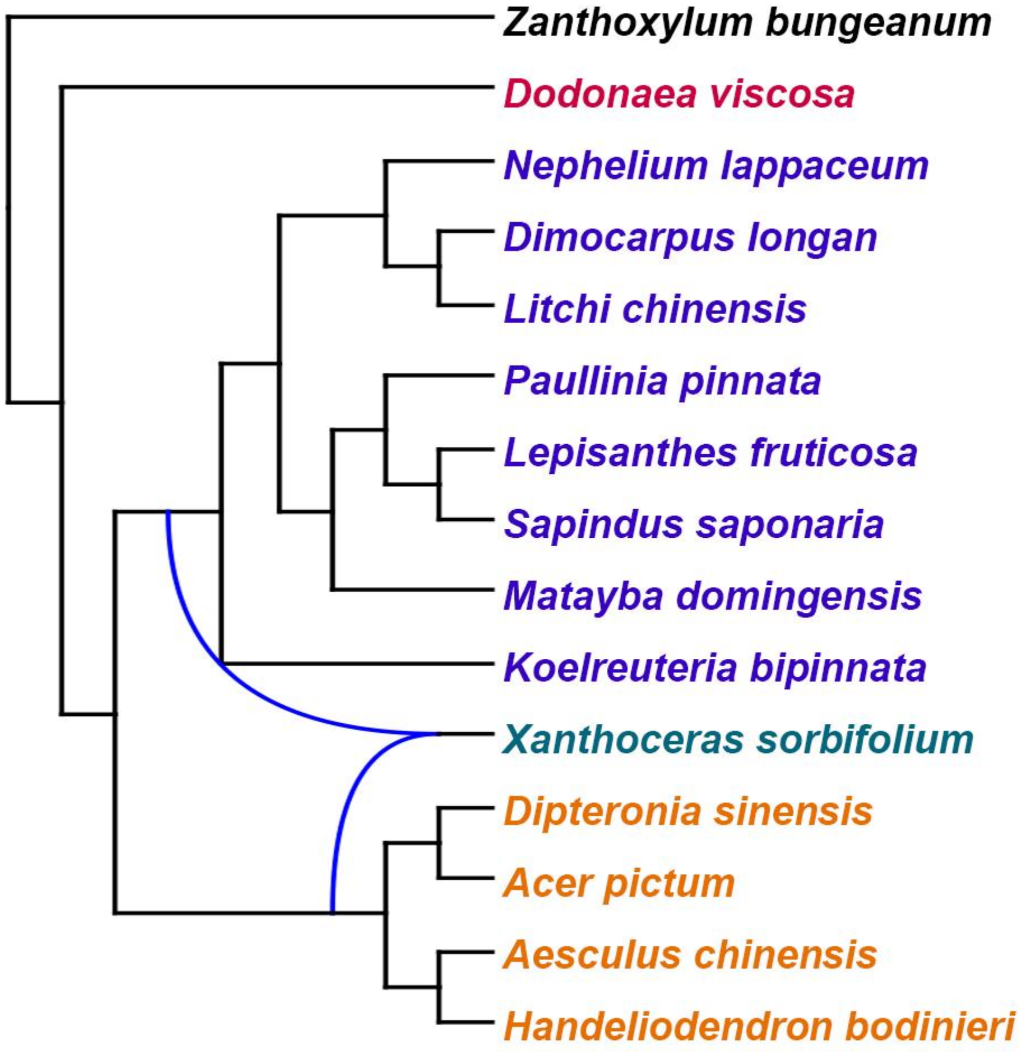

Max allowed hybrids: 10 times.
