# Supplementary figures and images for: Unraveling the Ancient Introgression History of Xanthoceras (Sapindaceae): Insights from Phylogenomic Analysis
Source: Int J Mol Sci. 2025 Feb 13;26(4):1581. doi: 10.3390/ijms26041581 (PMC11855356; doi:10.3390/ijms26041581)

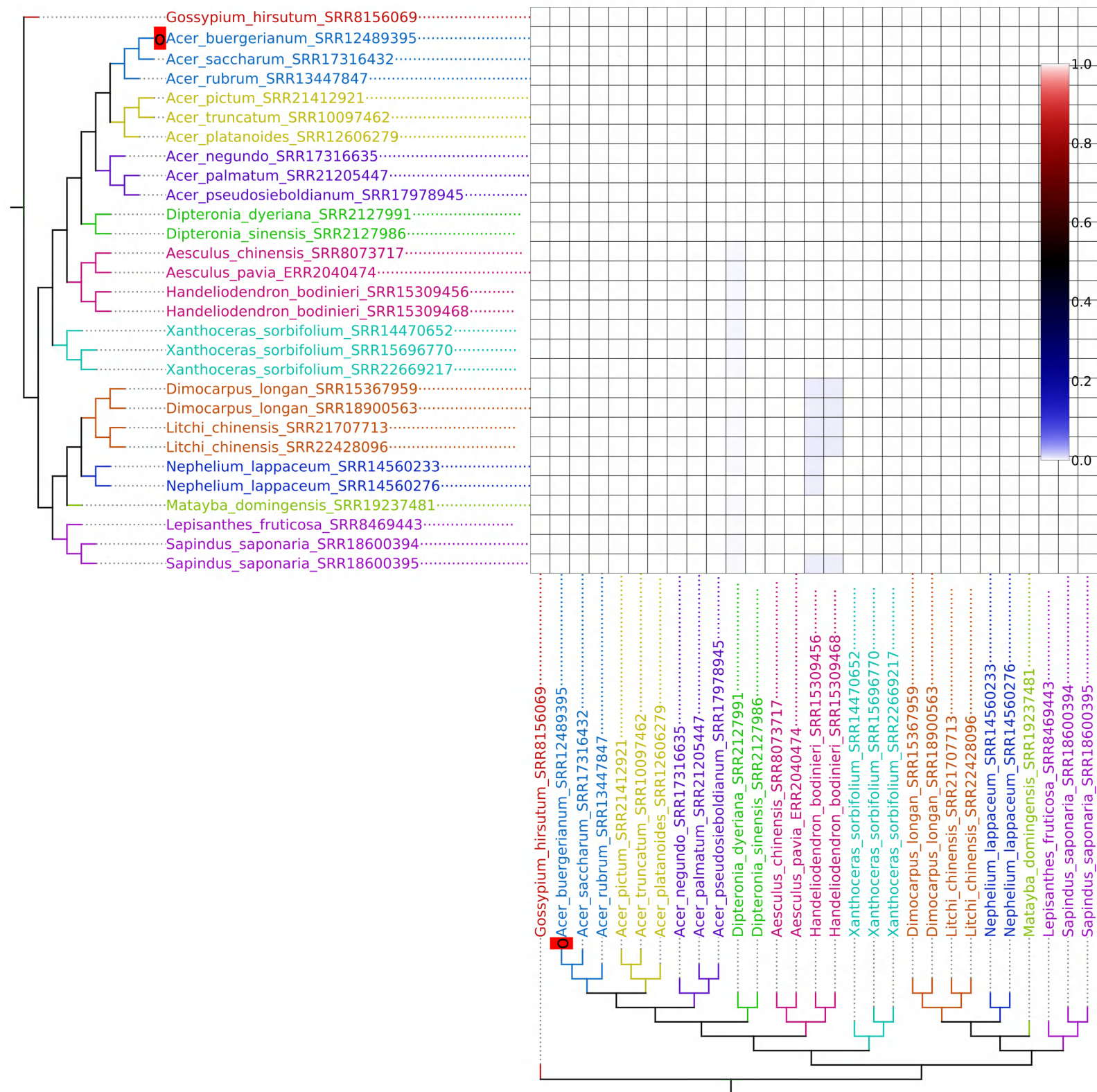

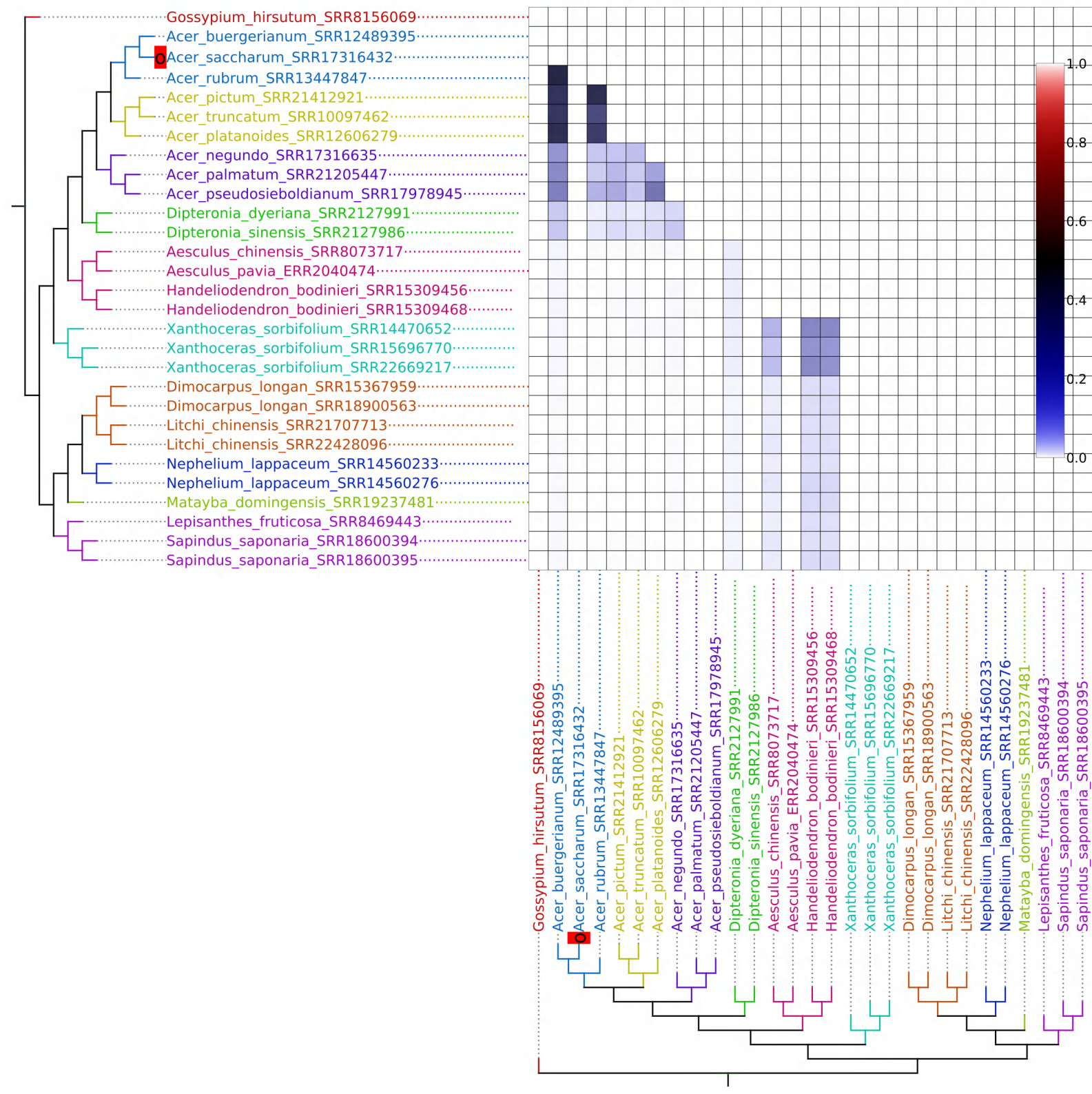

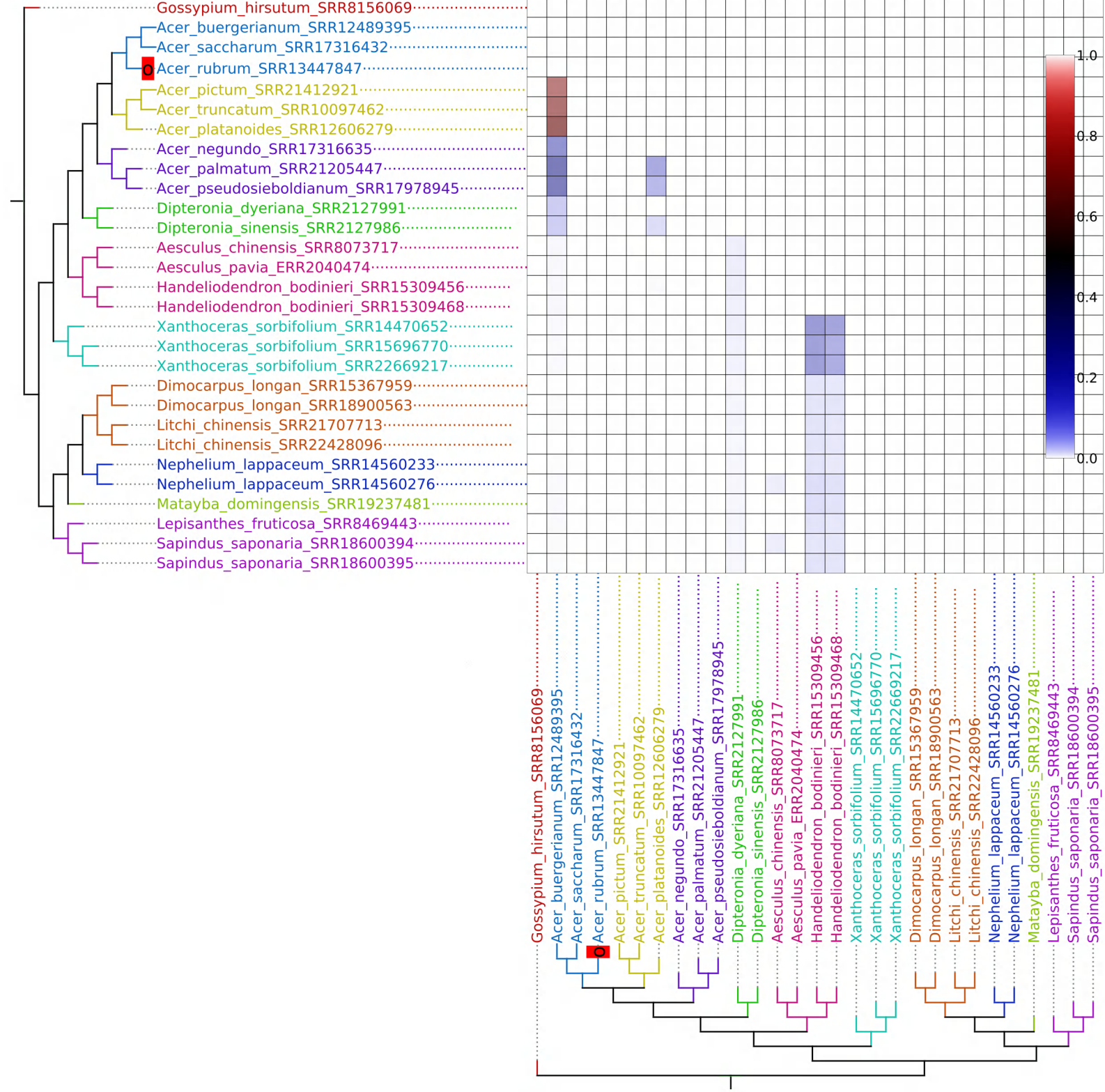

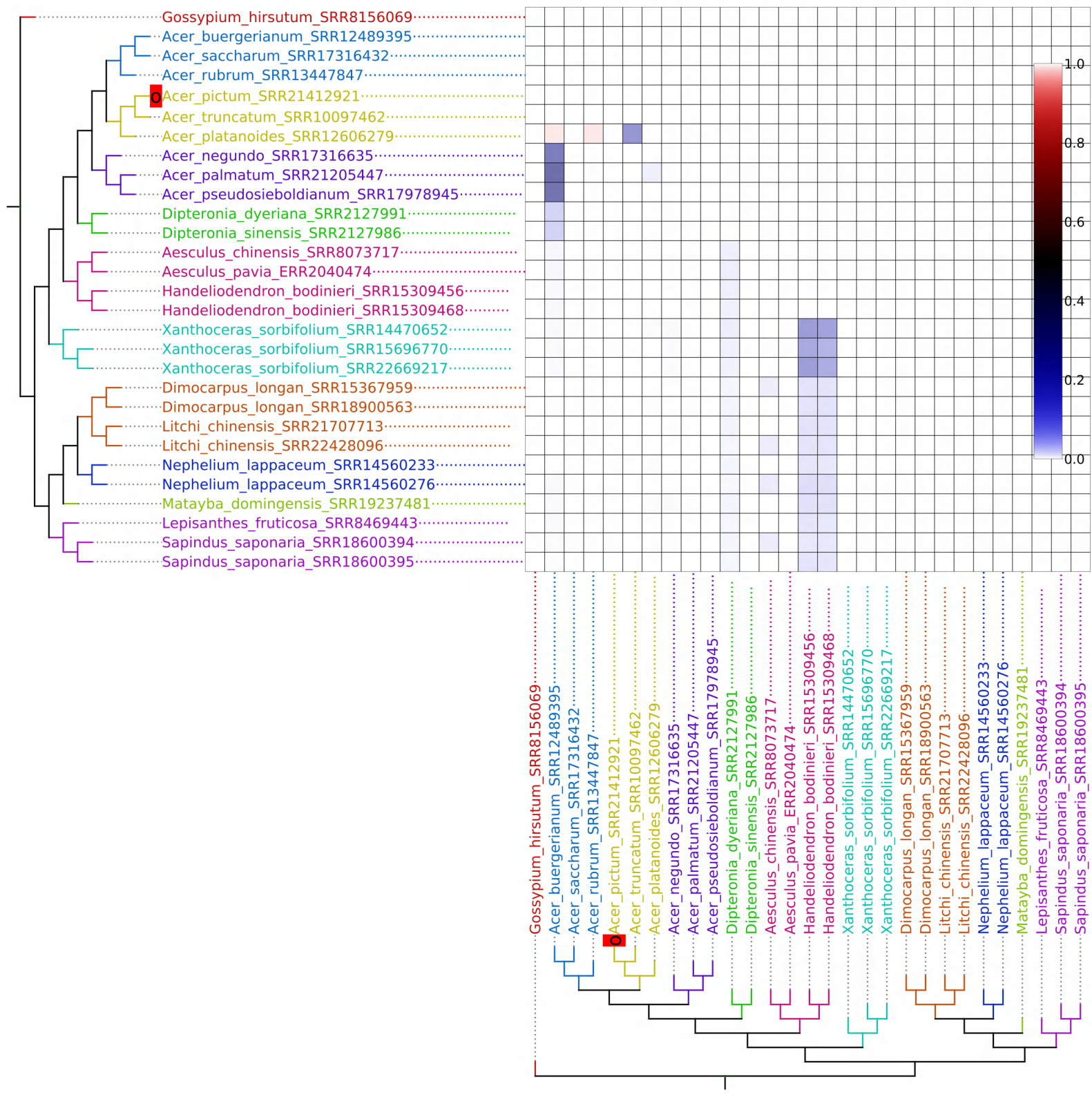

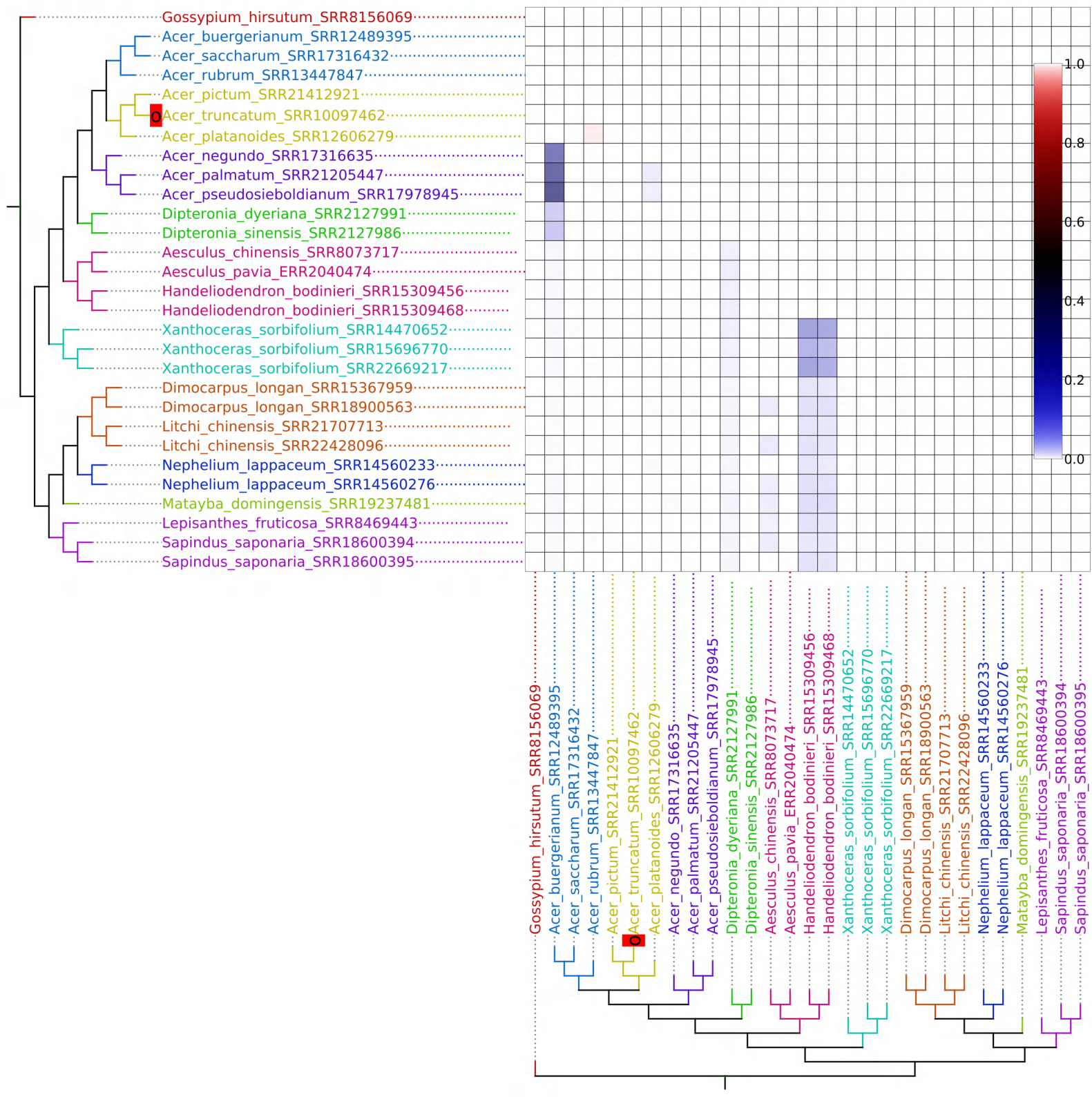

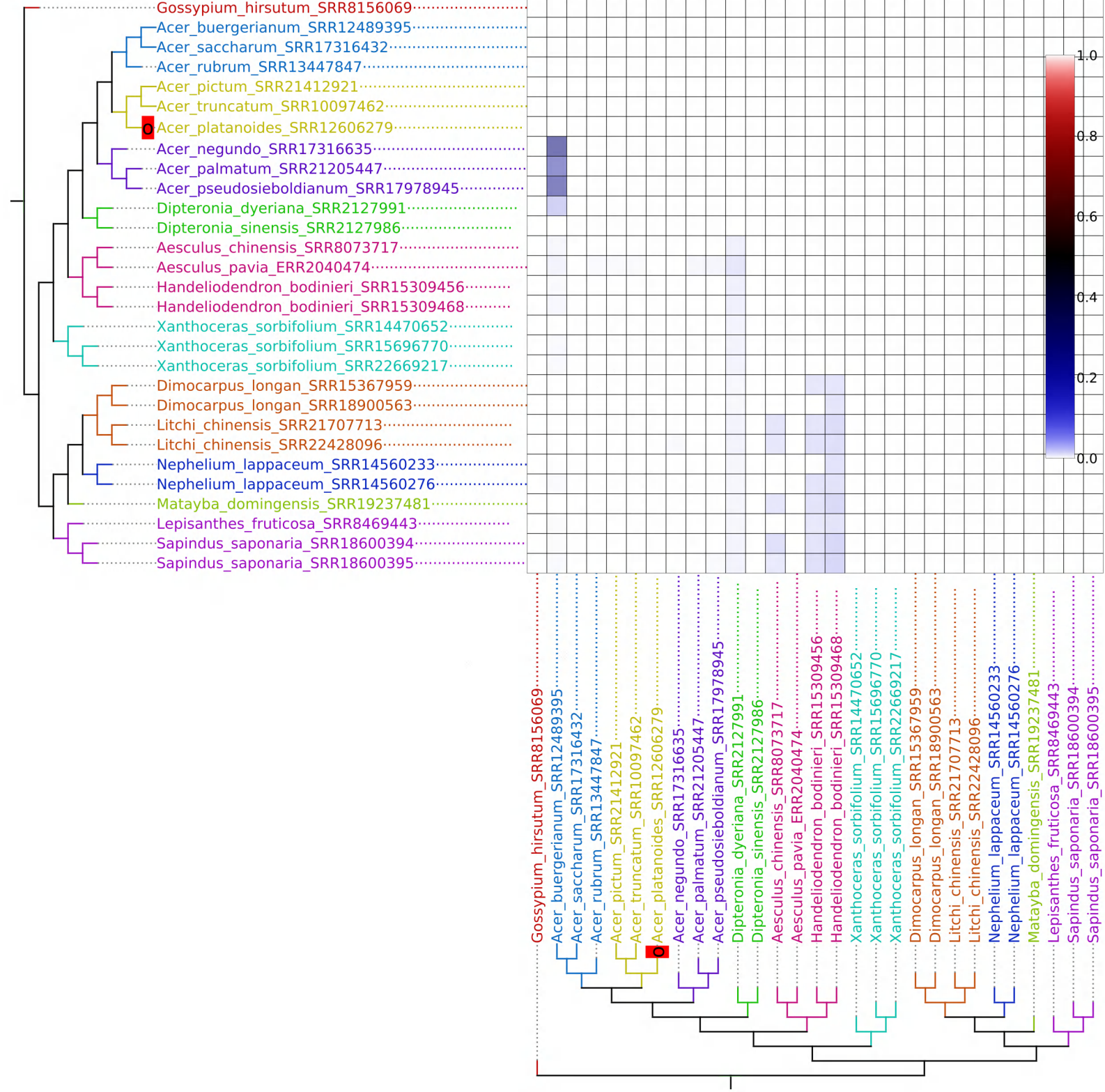

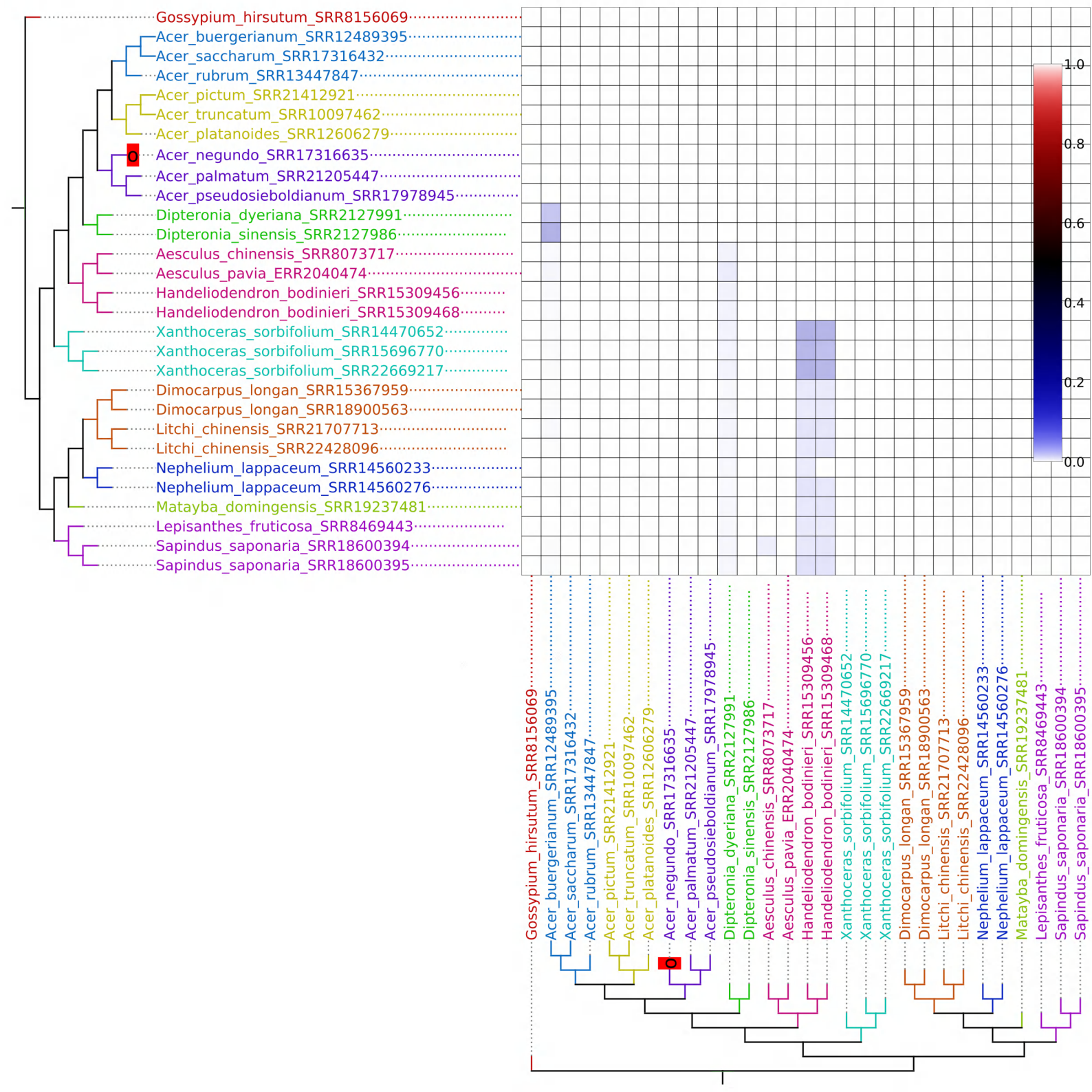

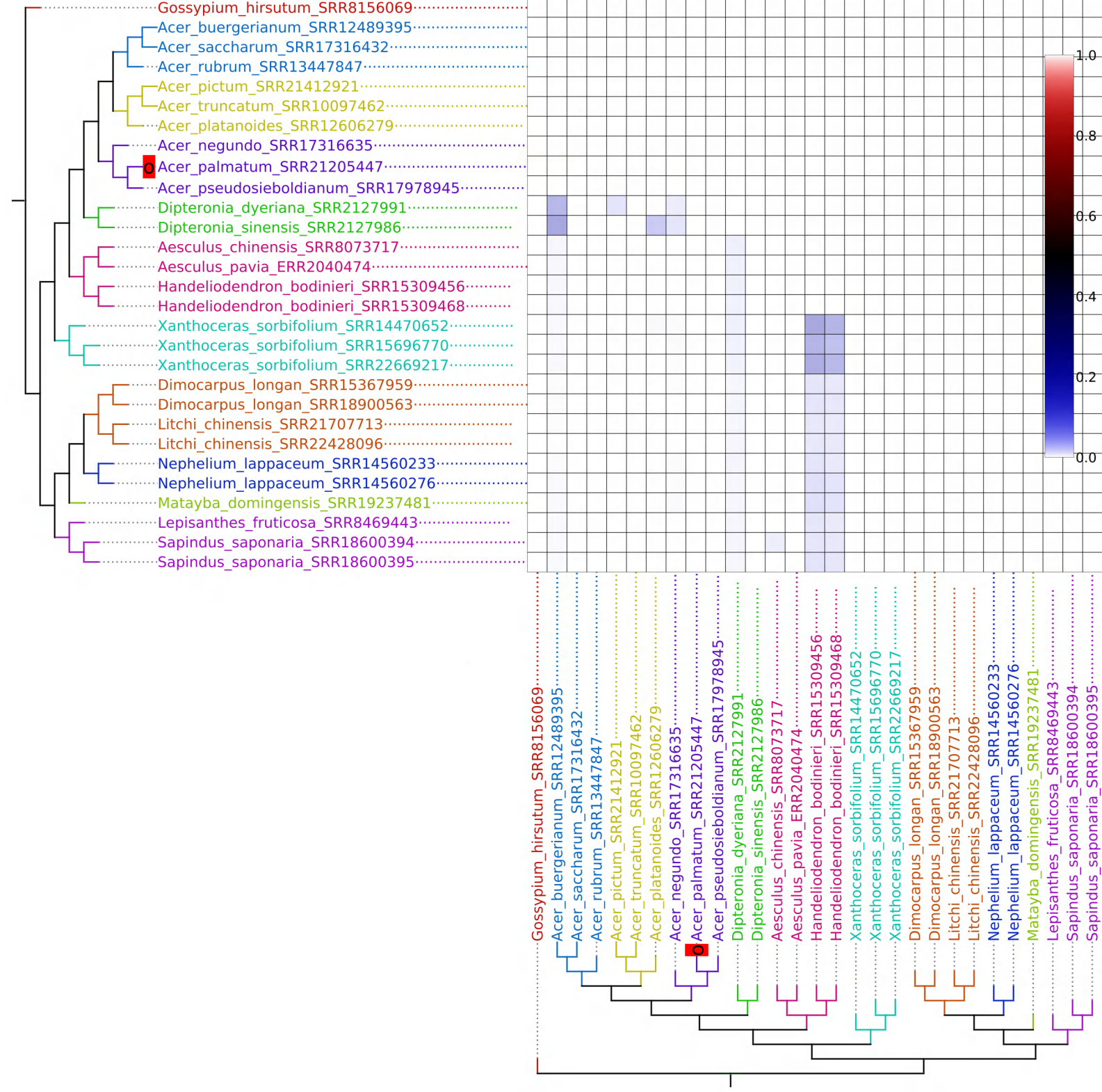



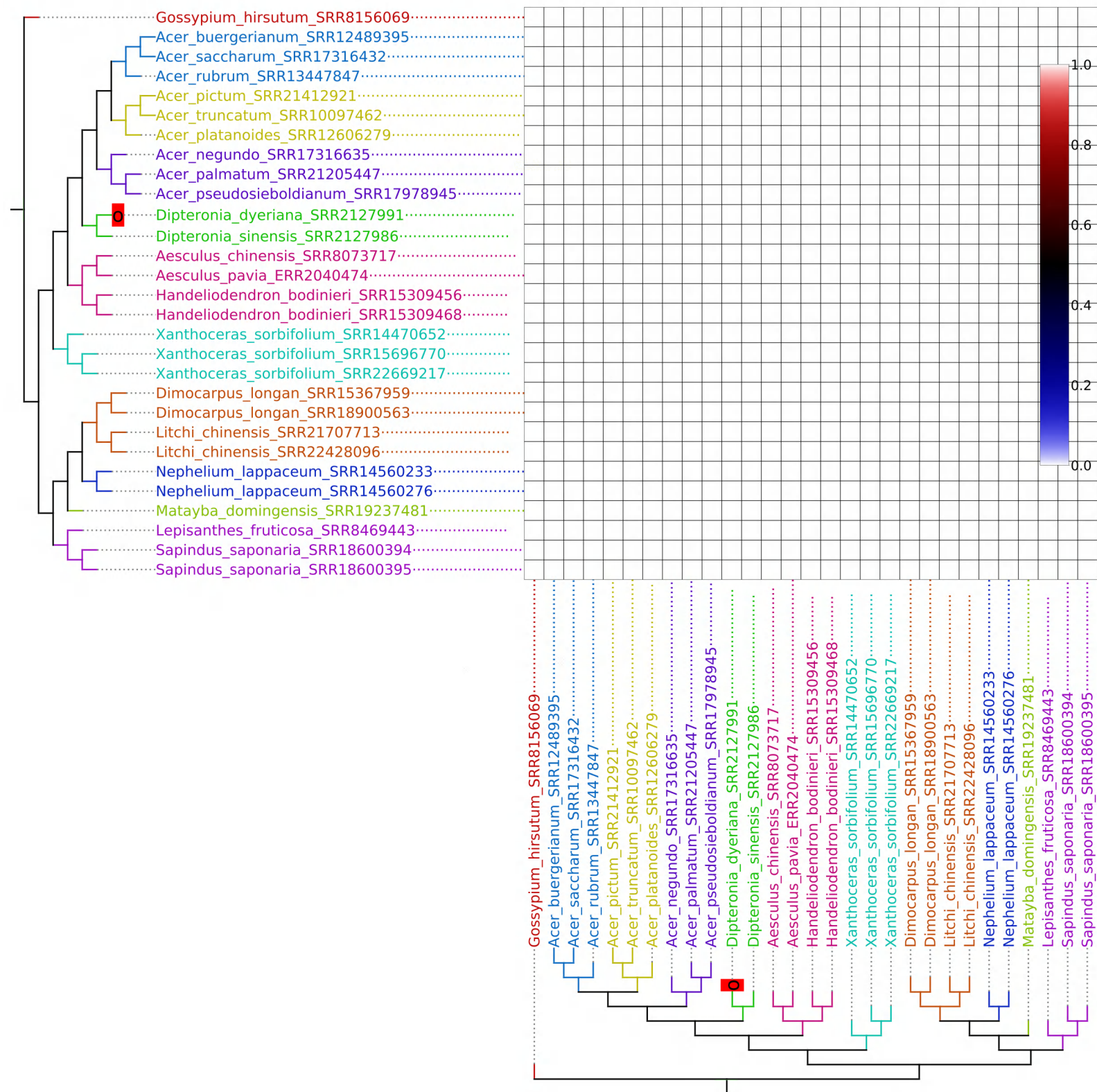

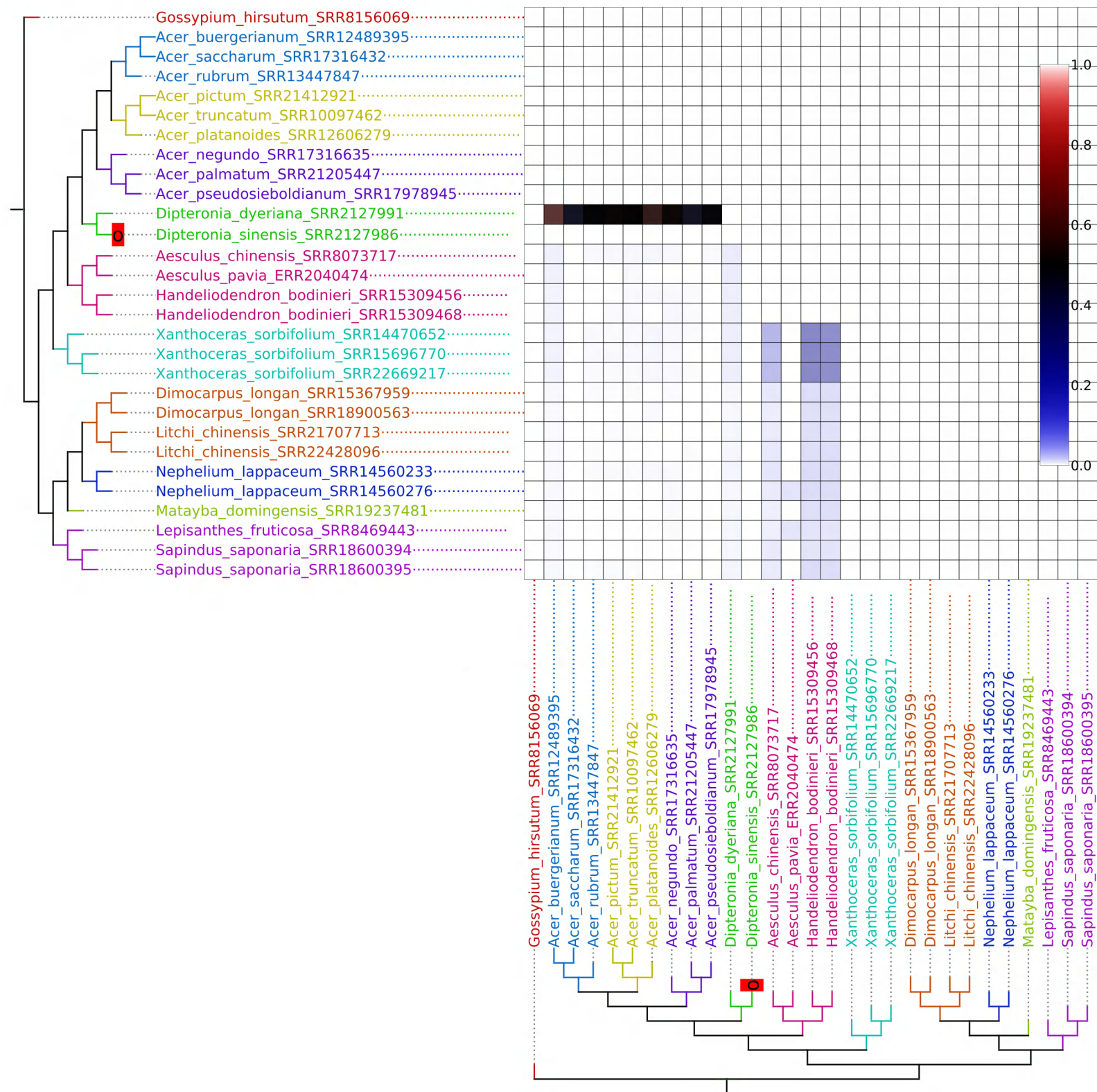

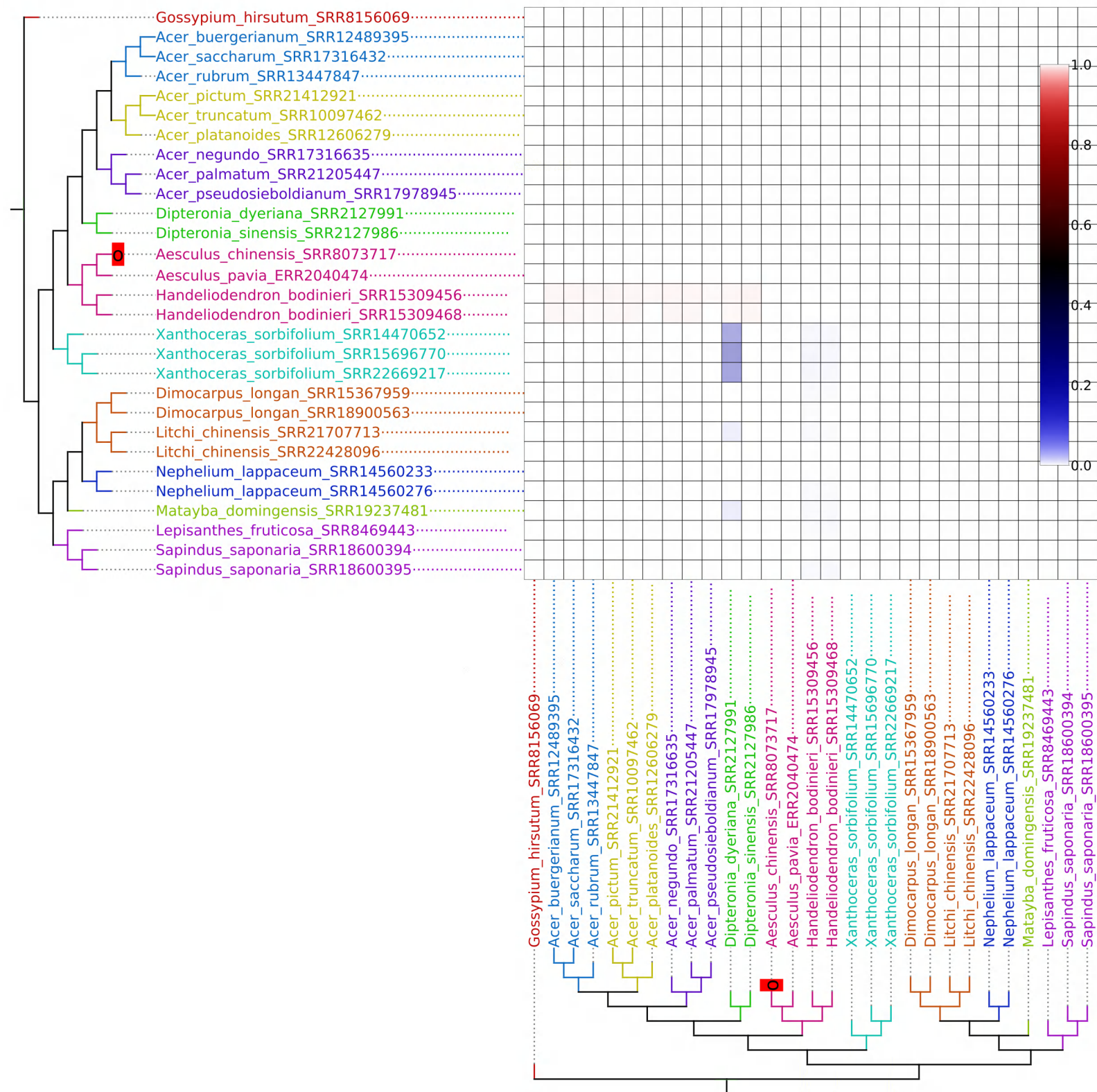

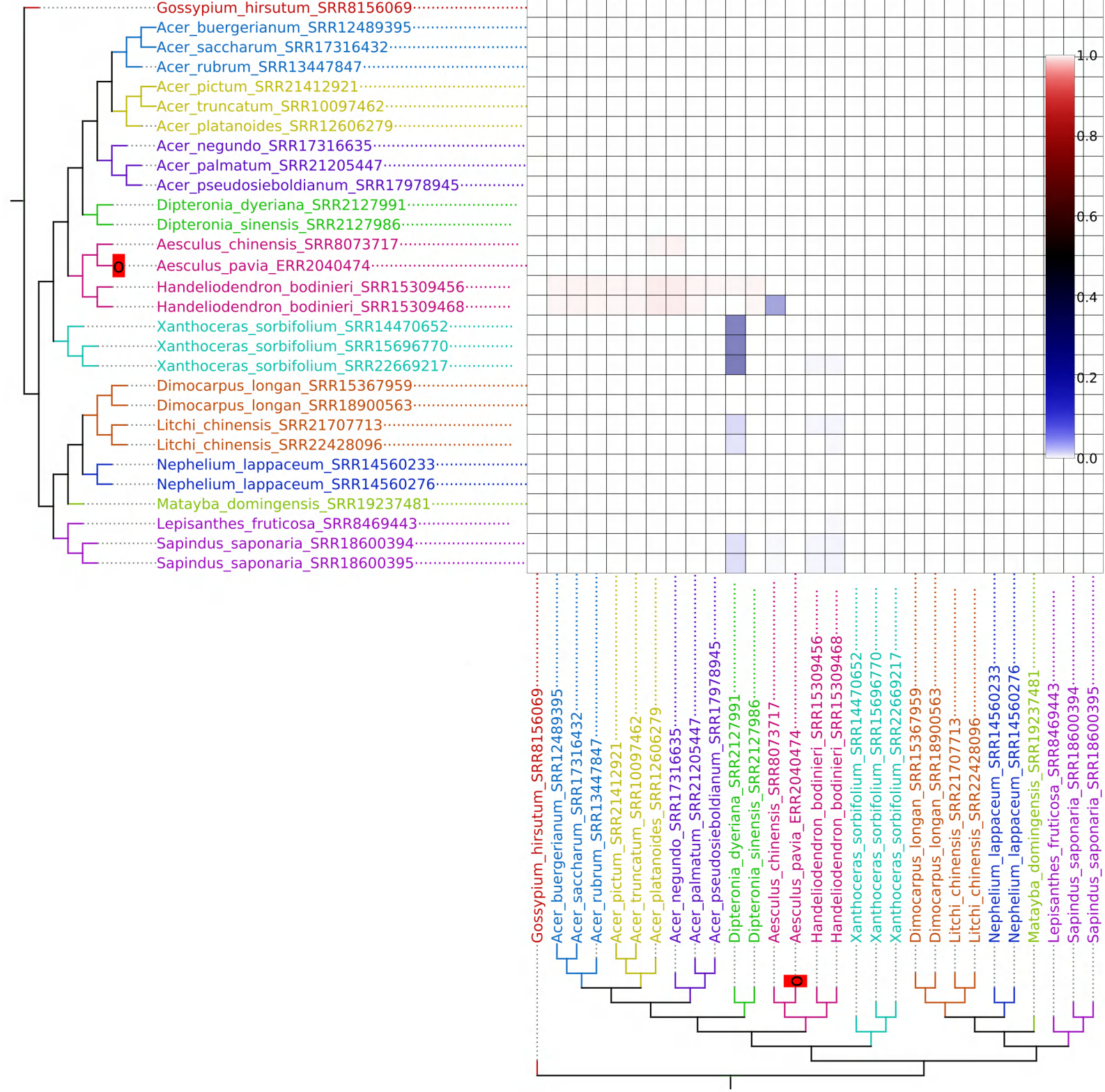

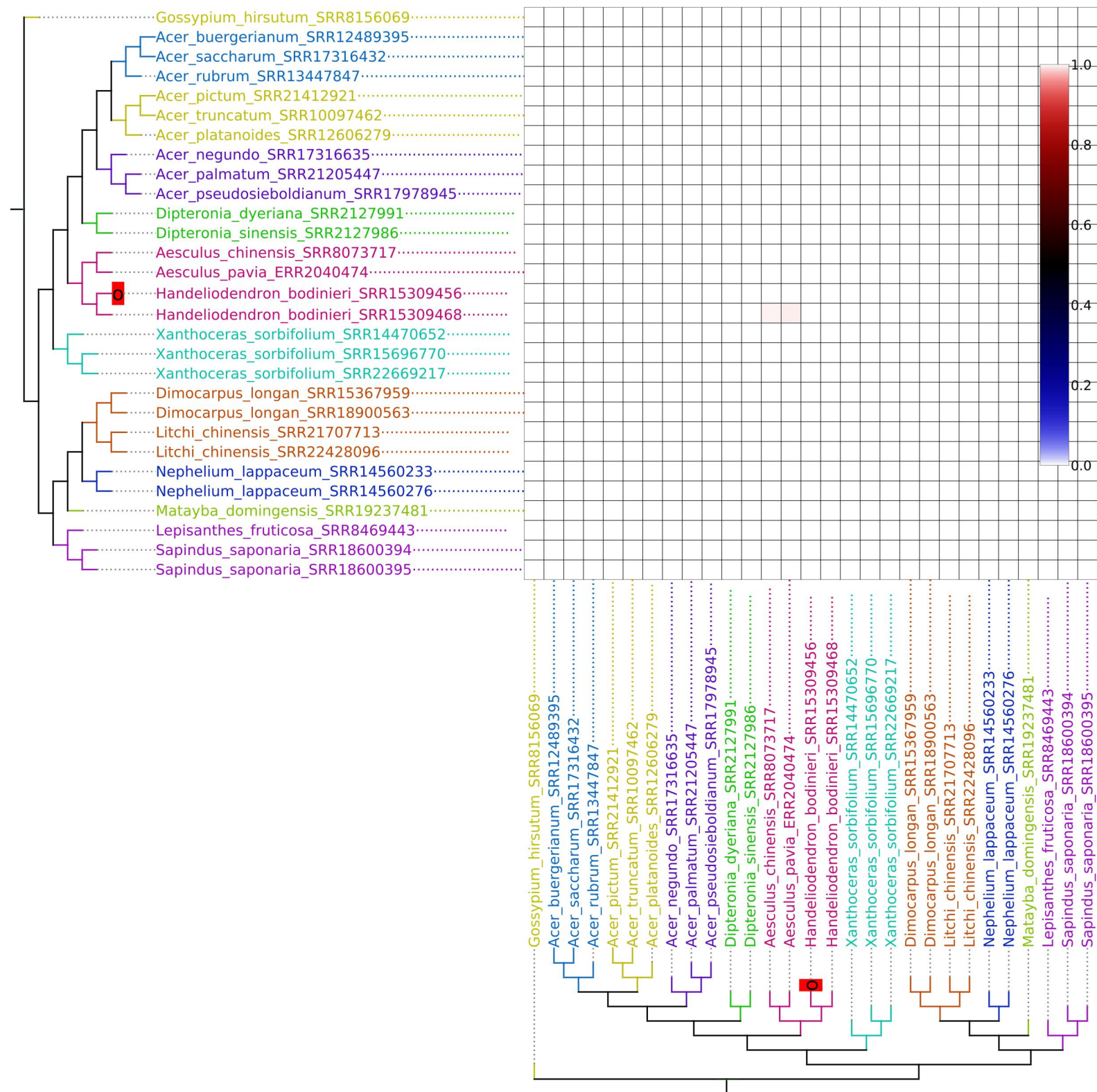

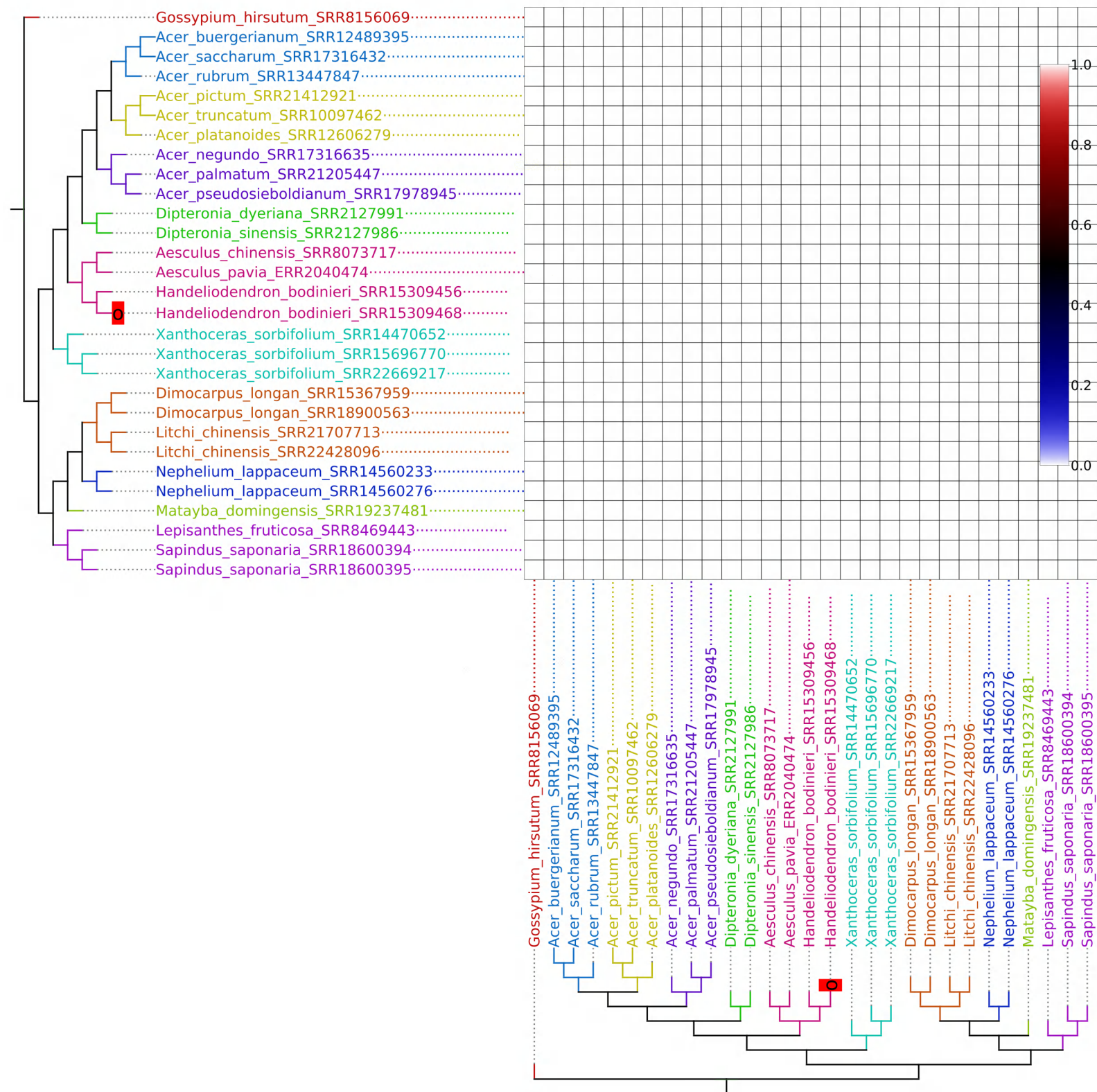

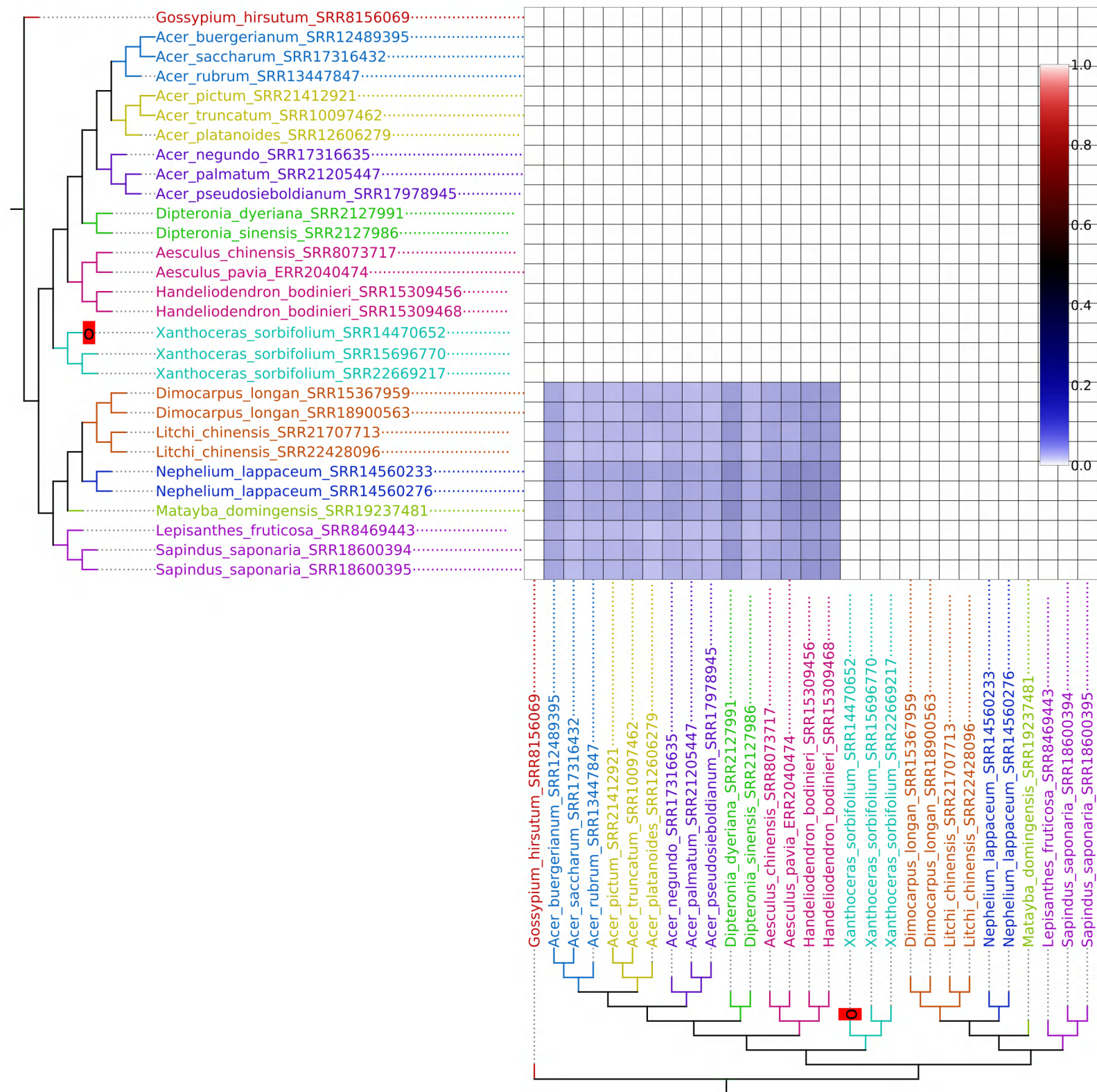

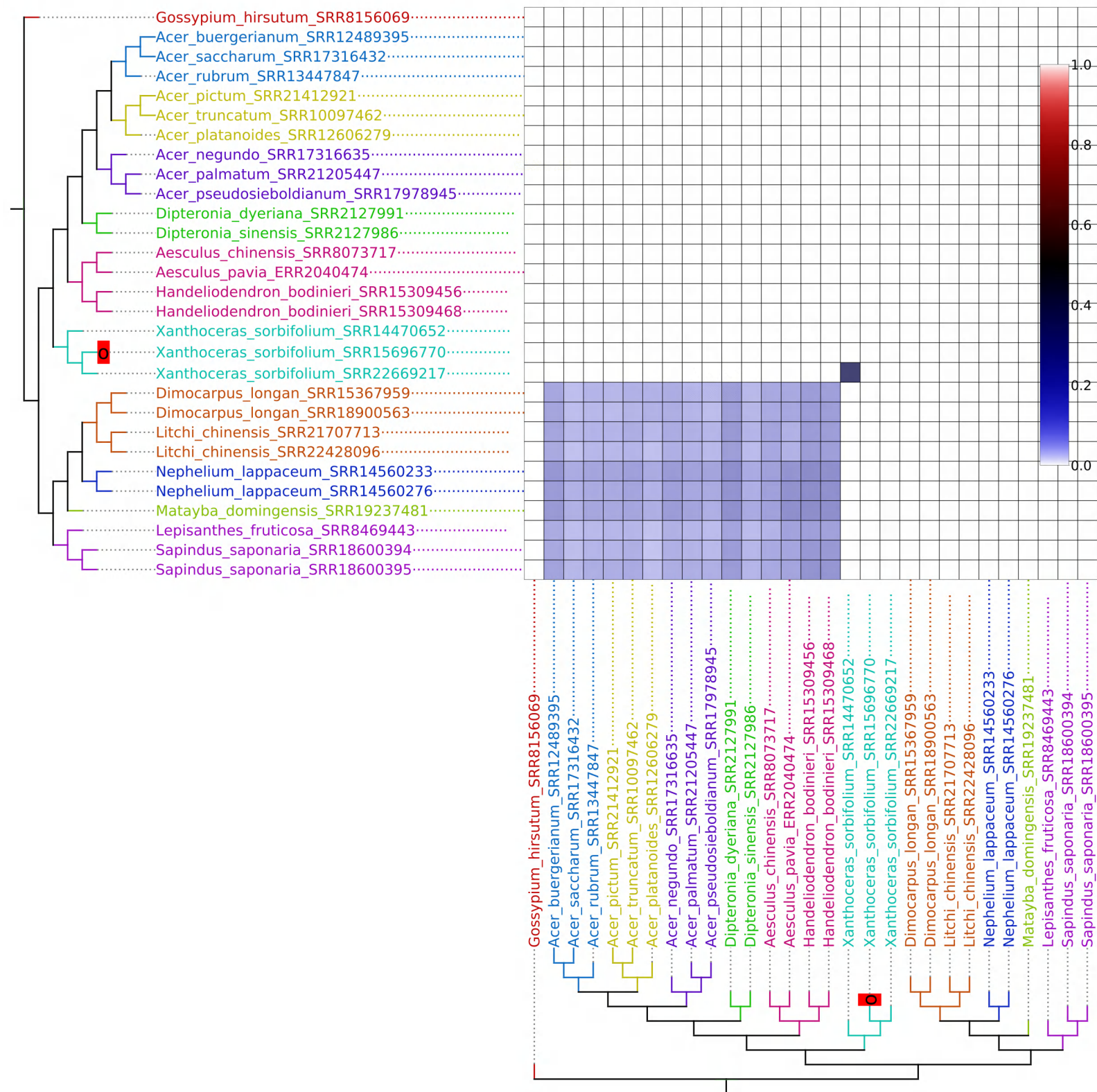

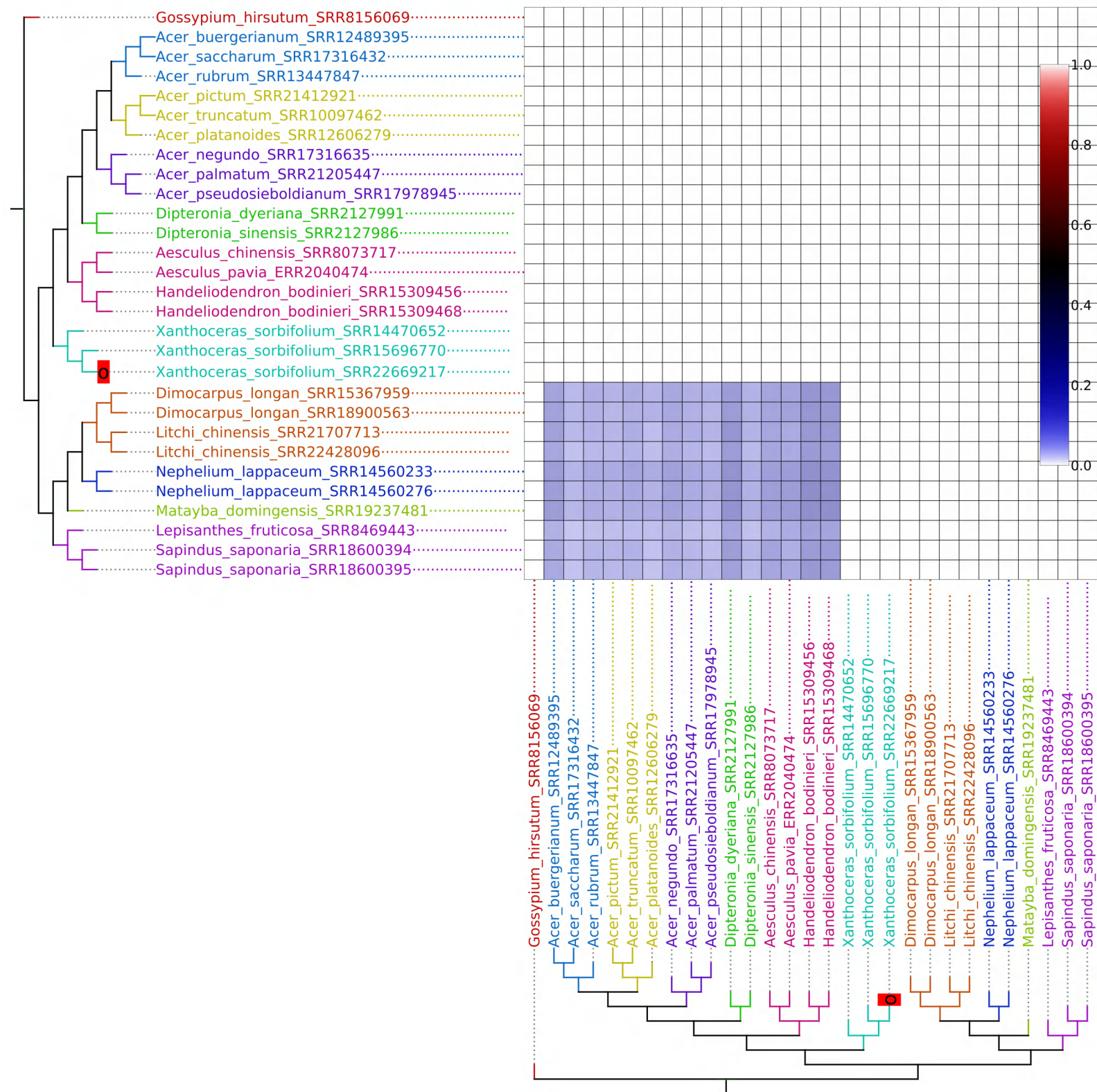

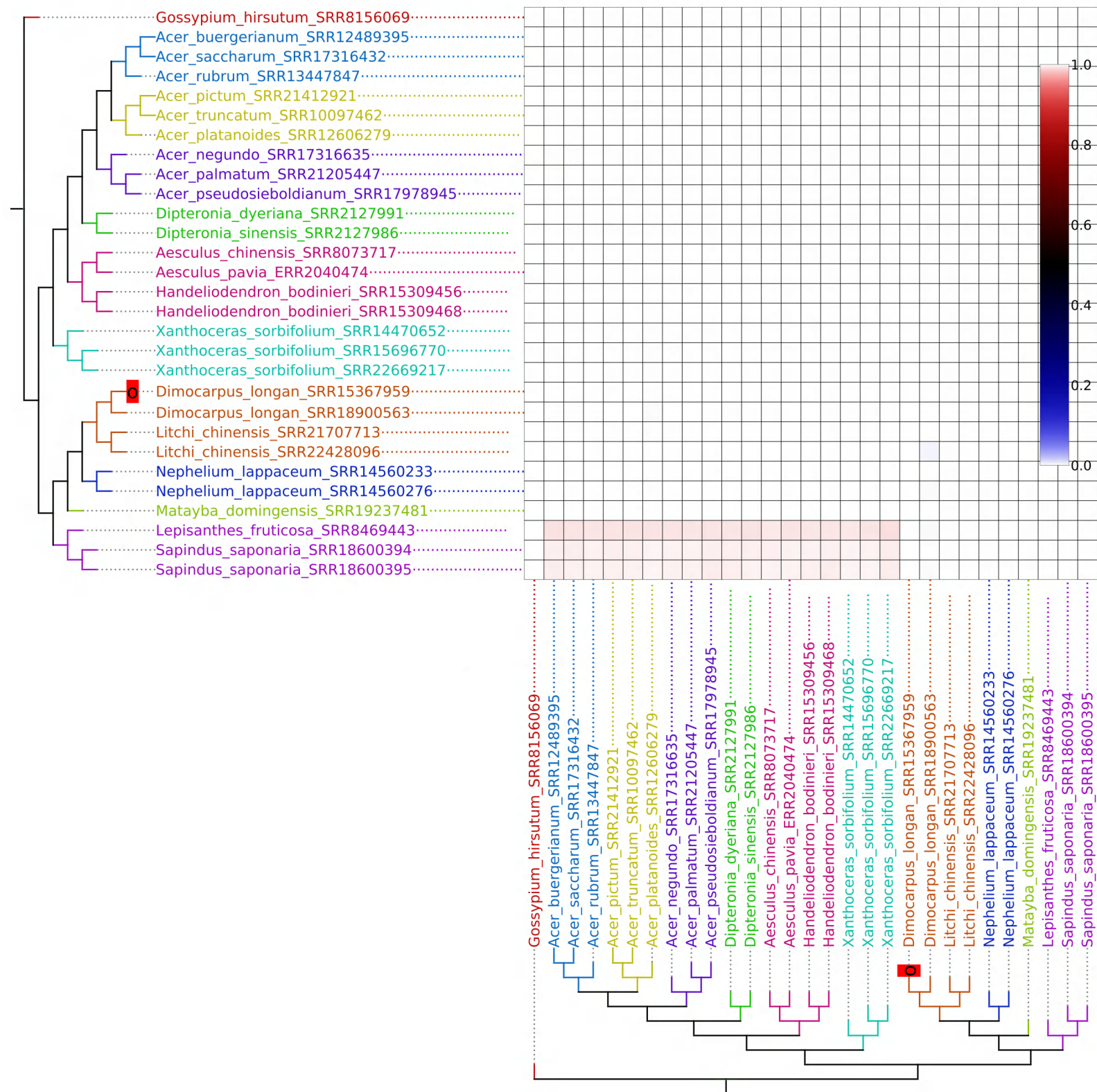

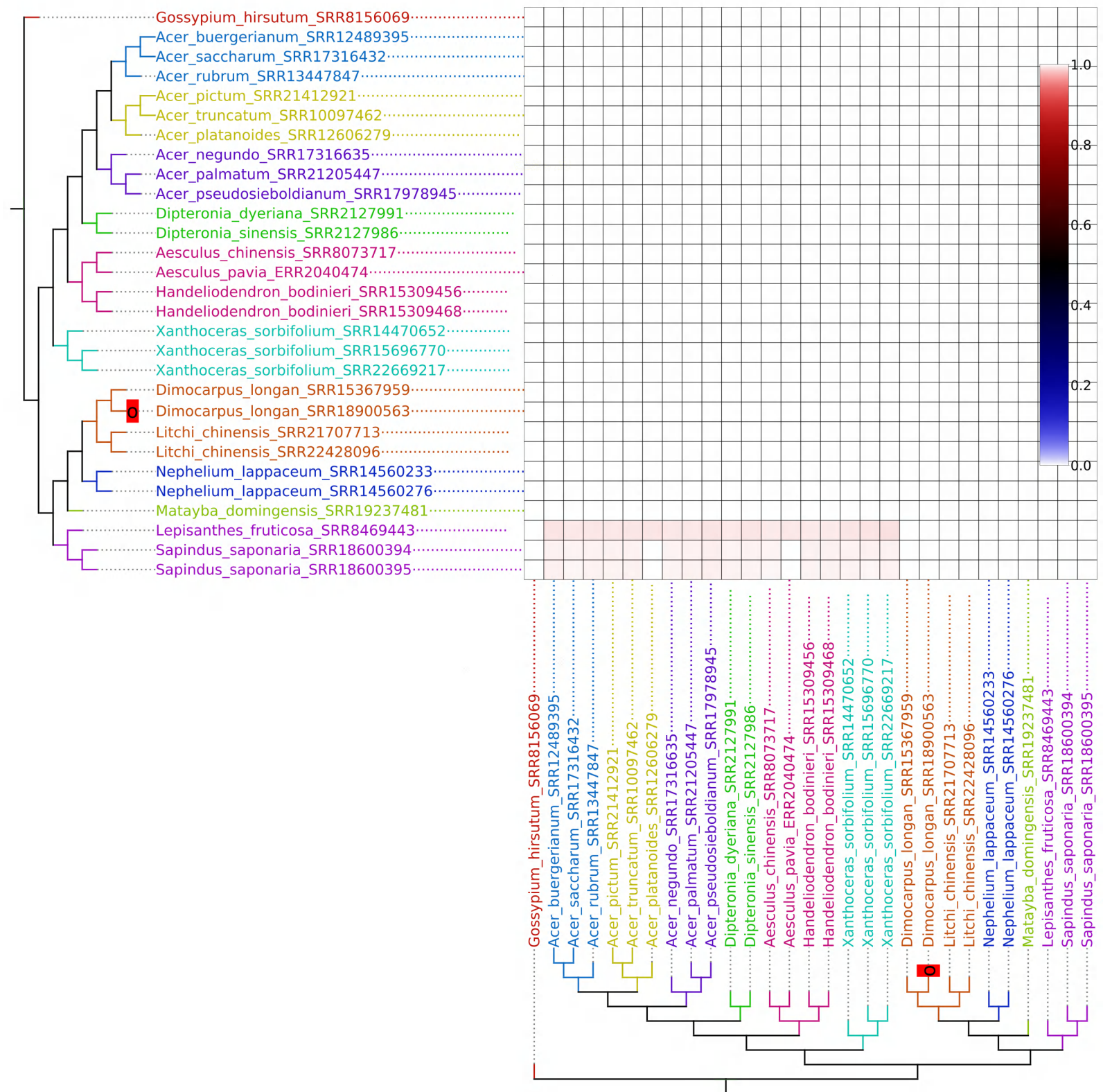

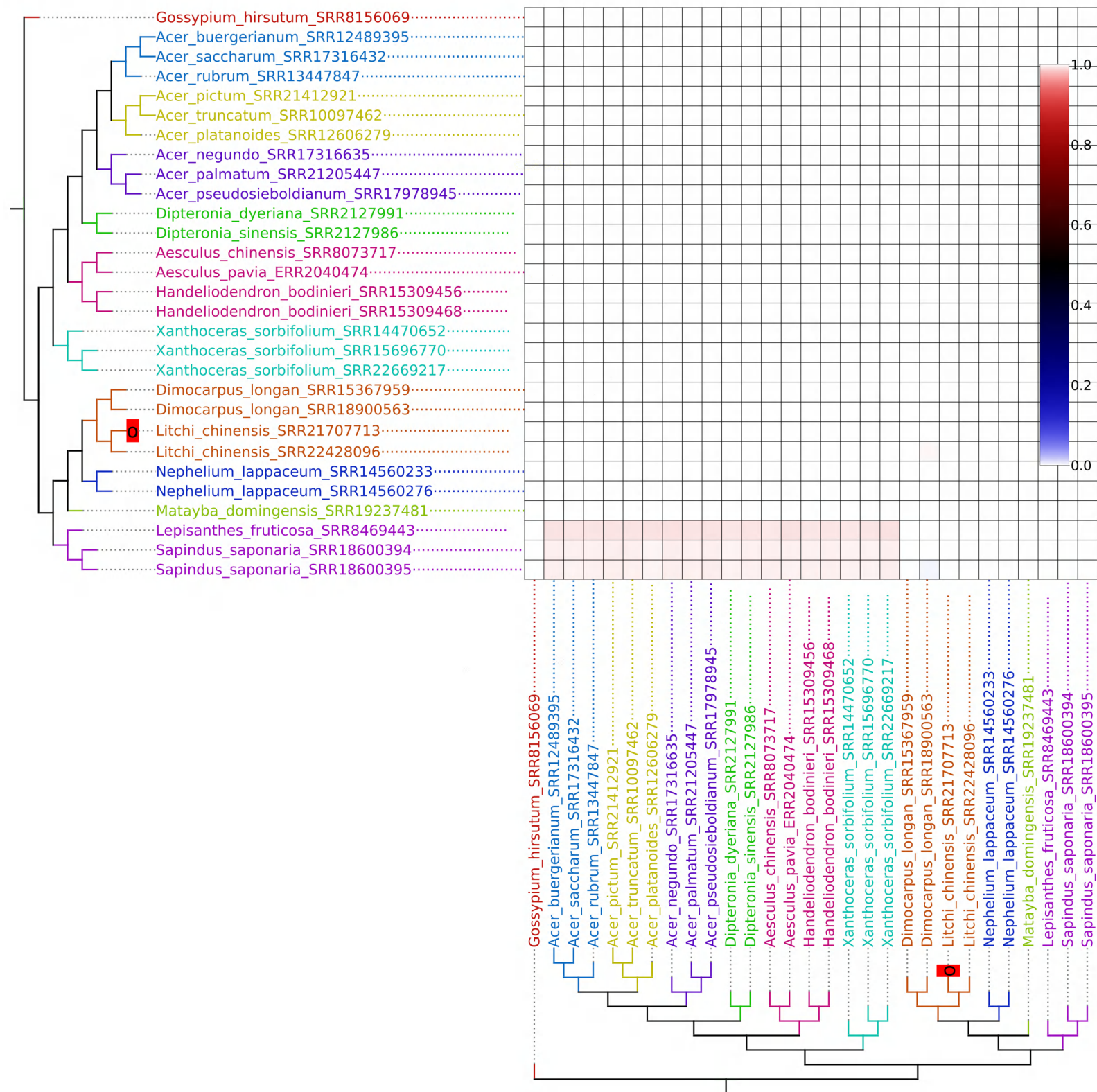

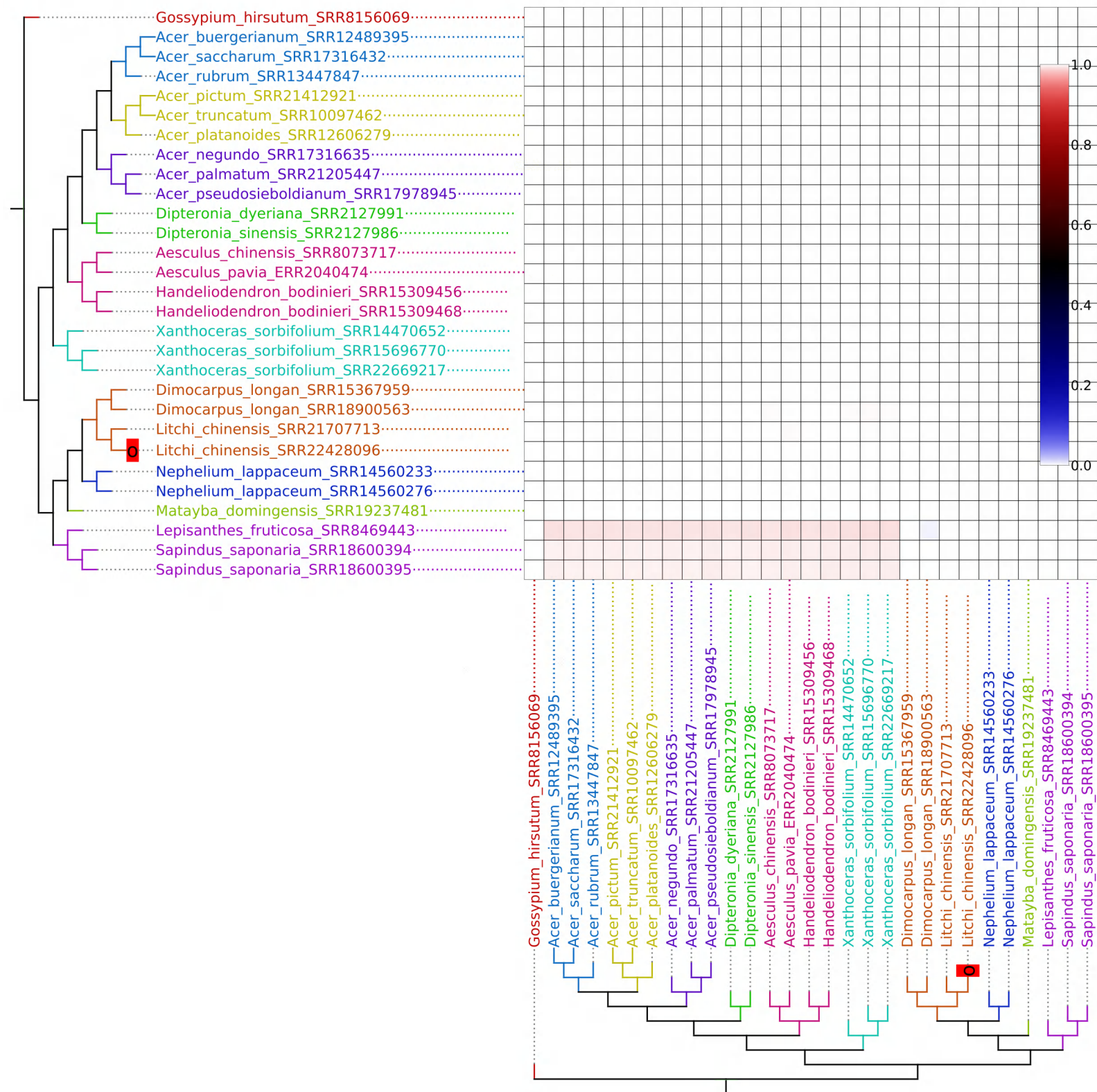

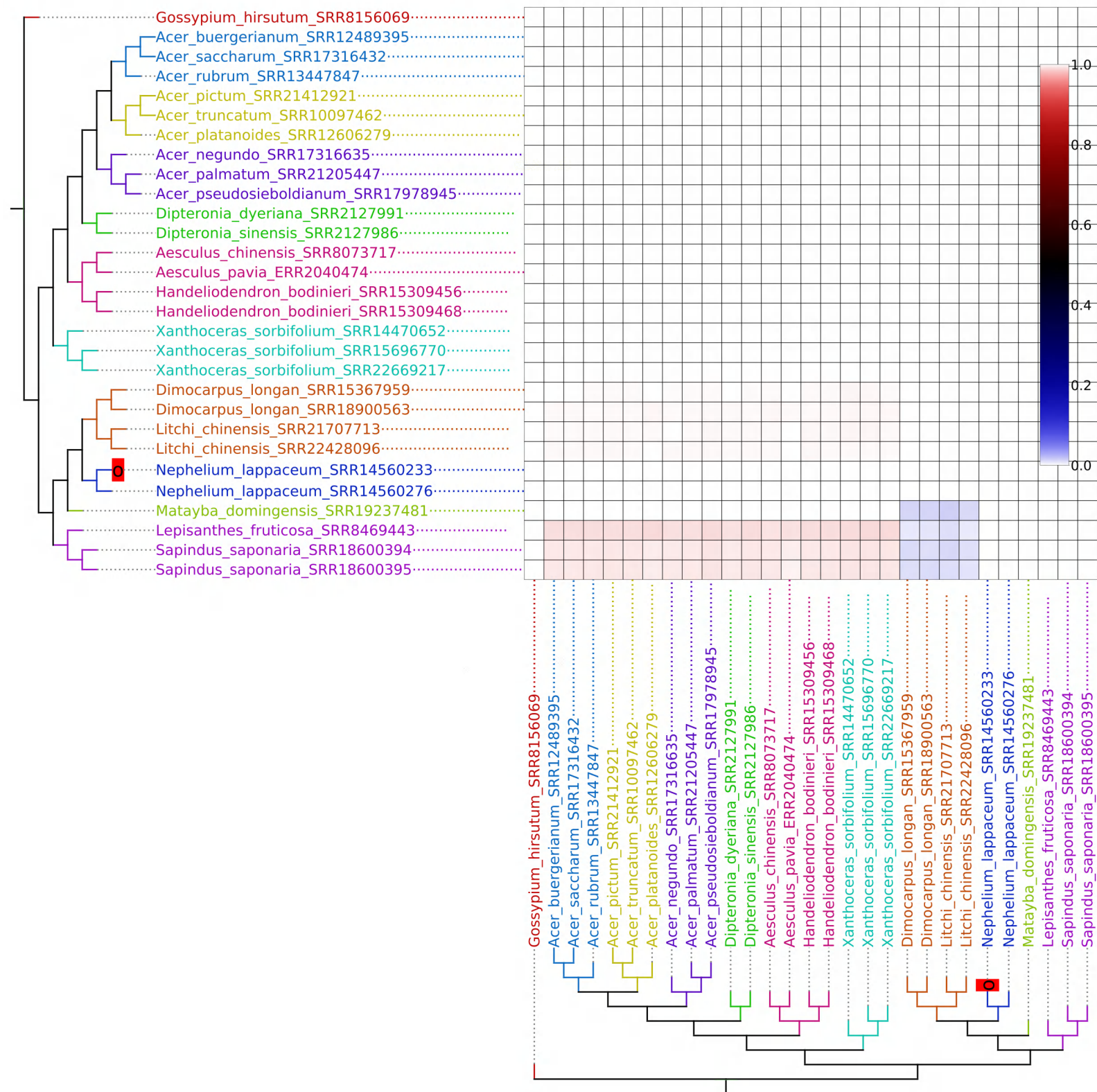

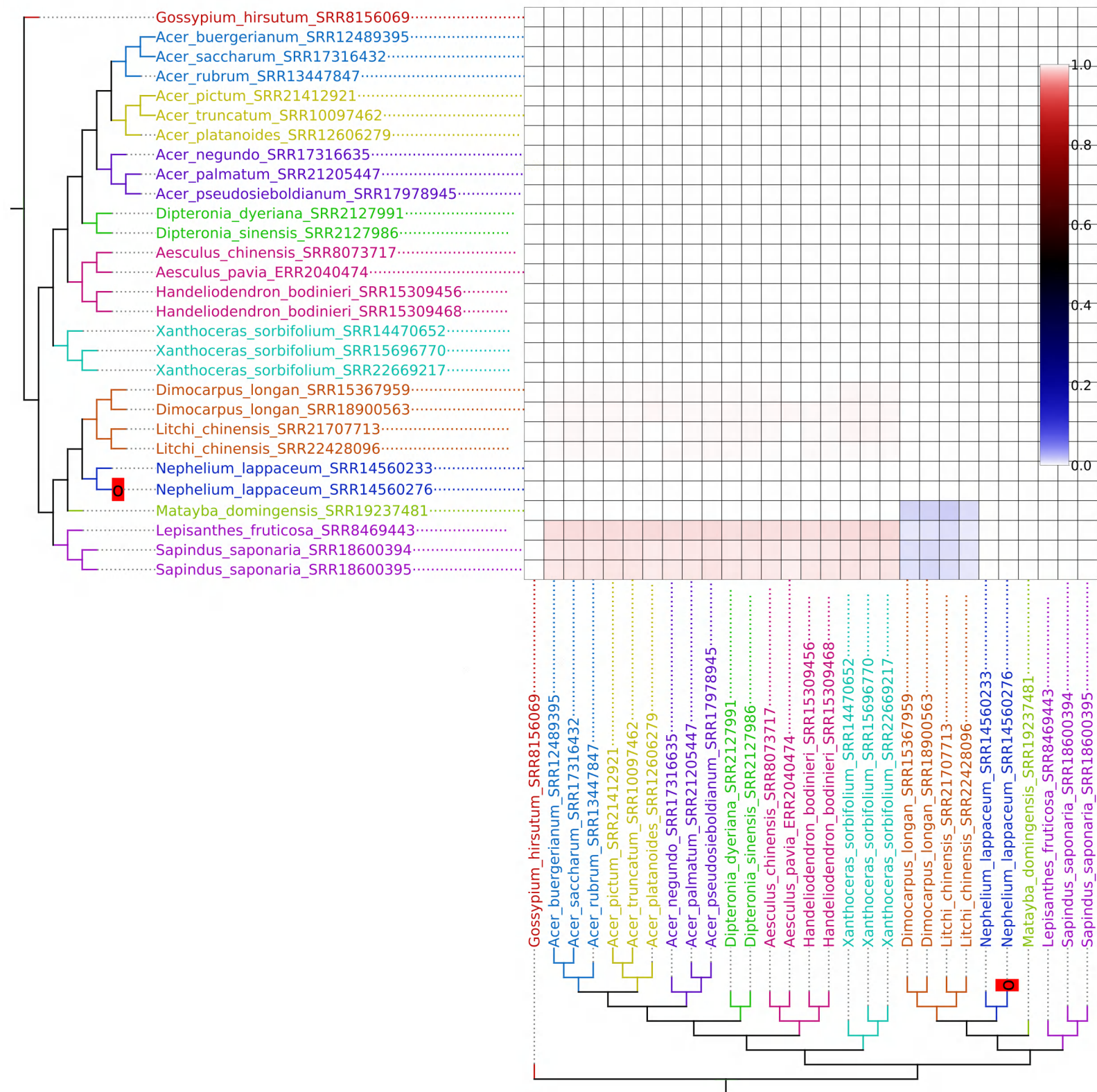

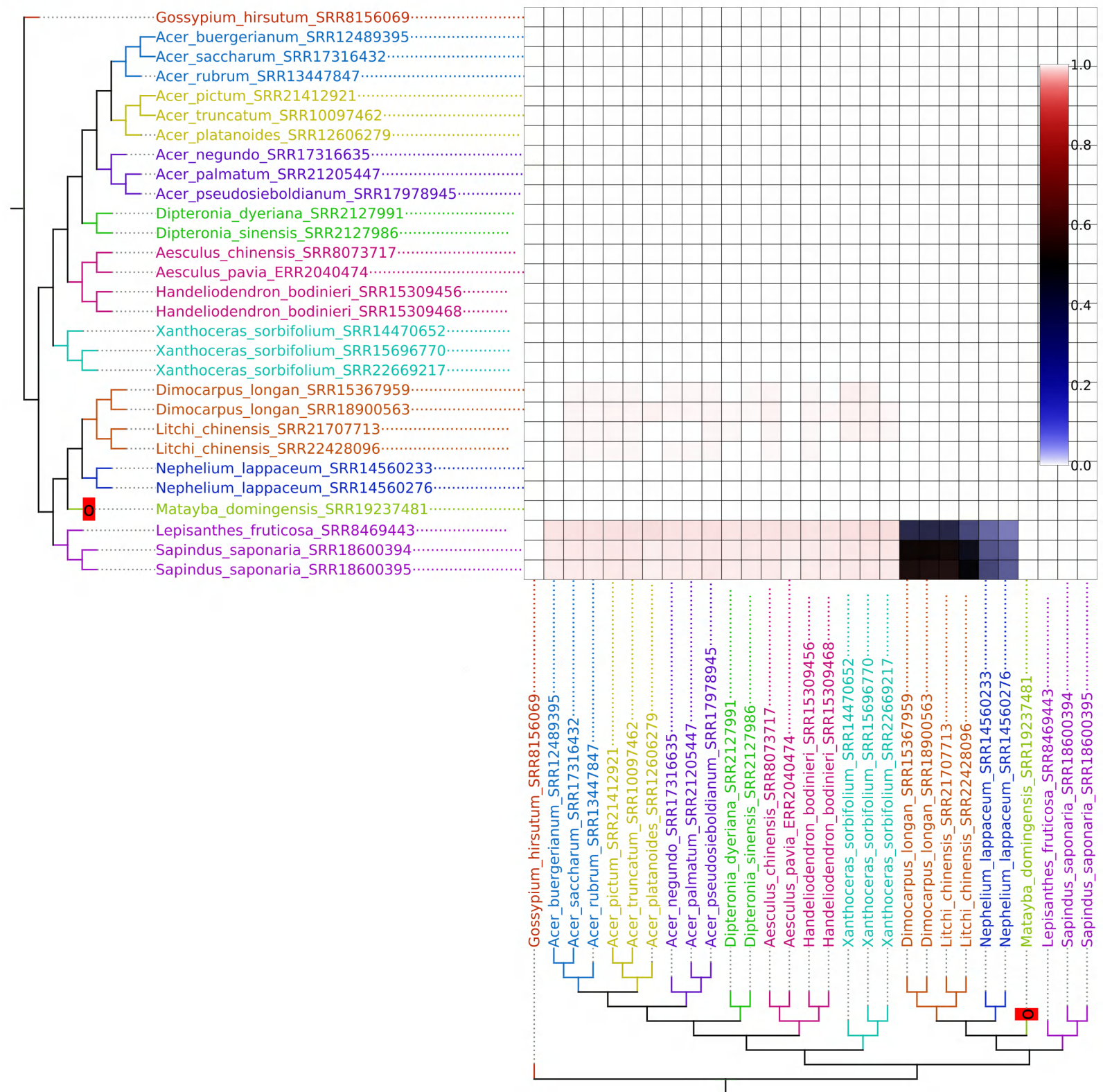

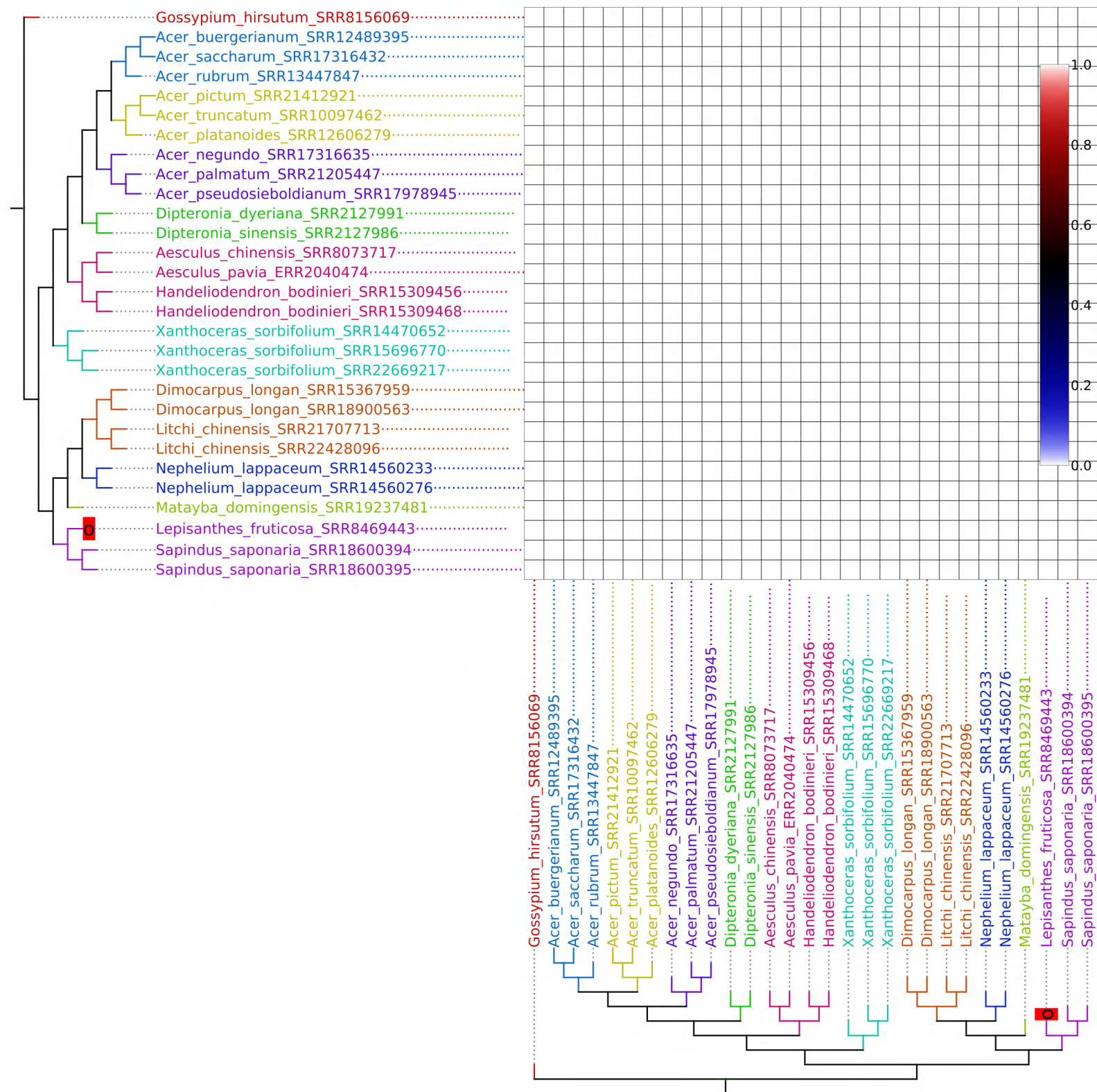

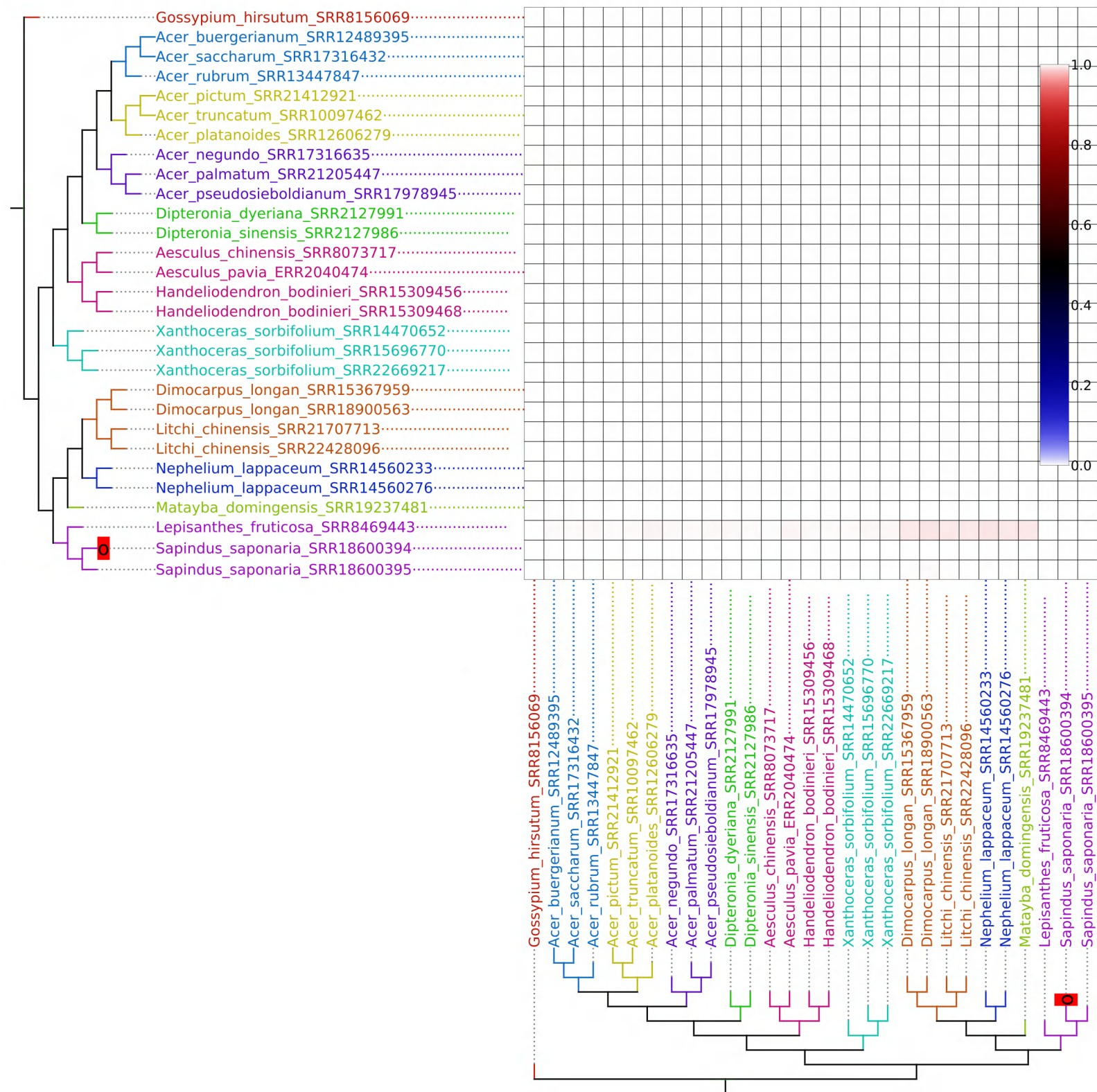

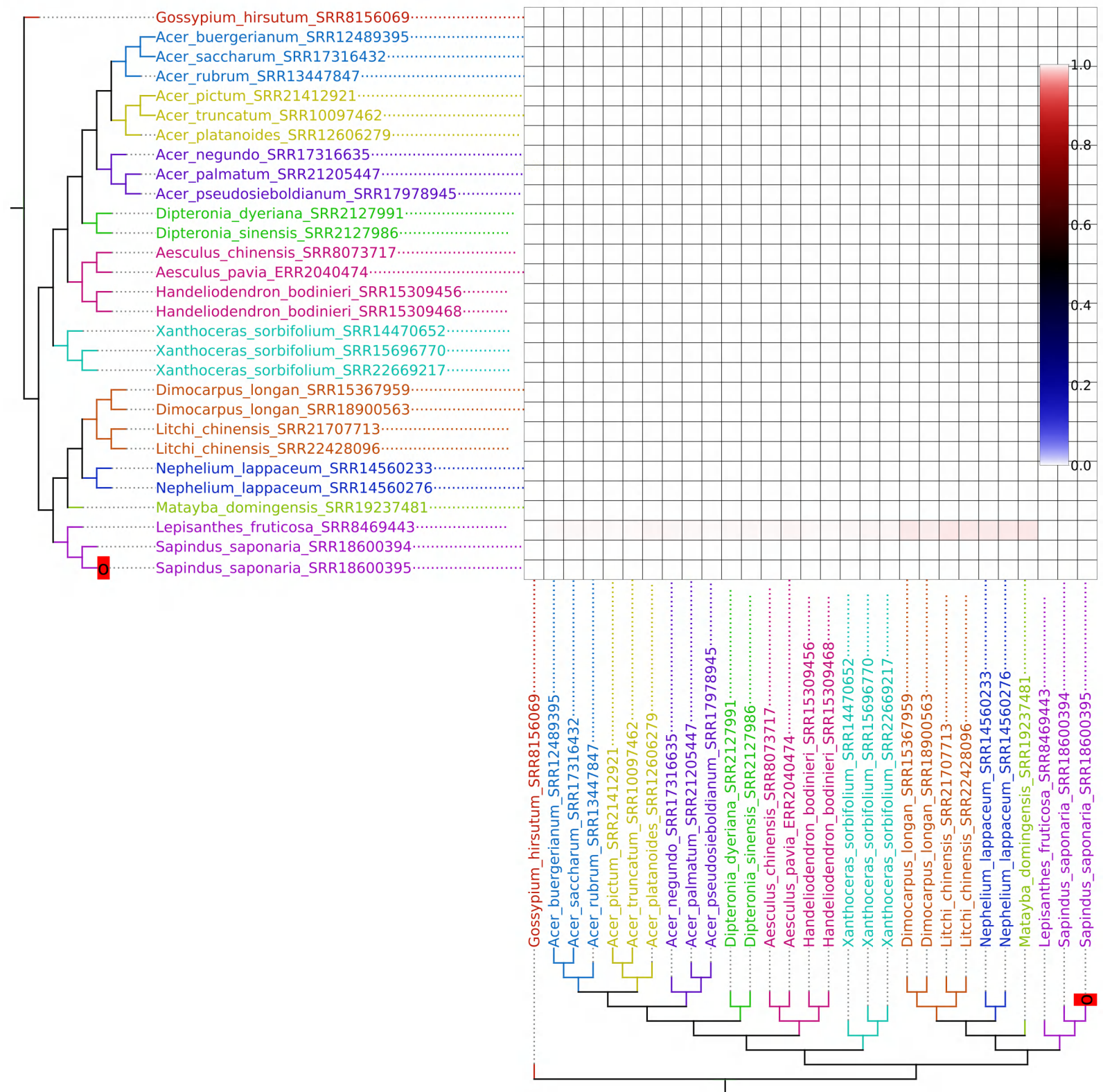

Supplement: Supplementary file 1 [file ijms-26-01581-s001.zip › Figure_S9.pdf]

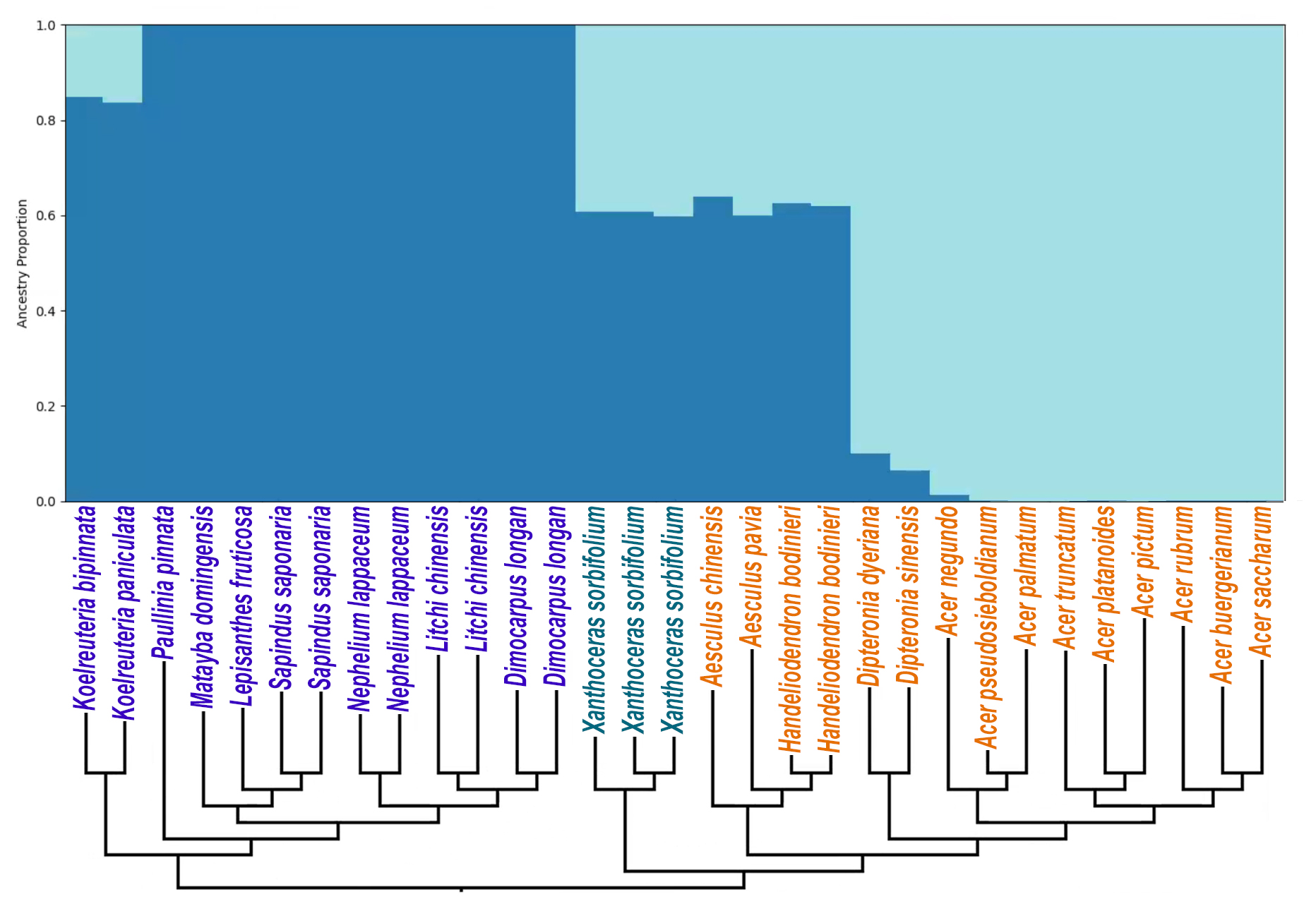

Supplement: Supplementary file 1 [file ijms-26-01581-s001.zip › Figure_S11.jpg]

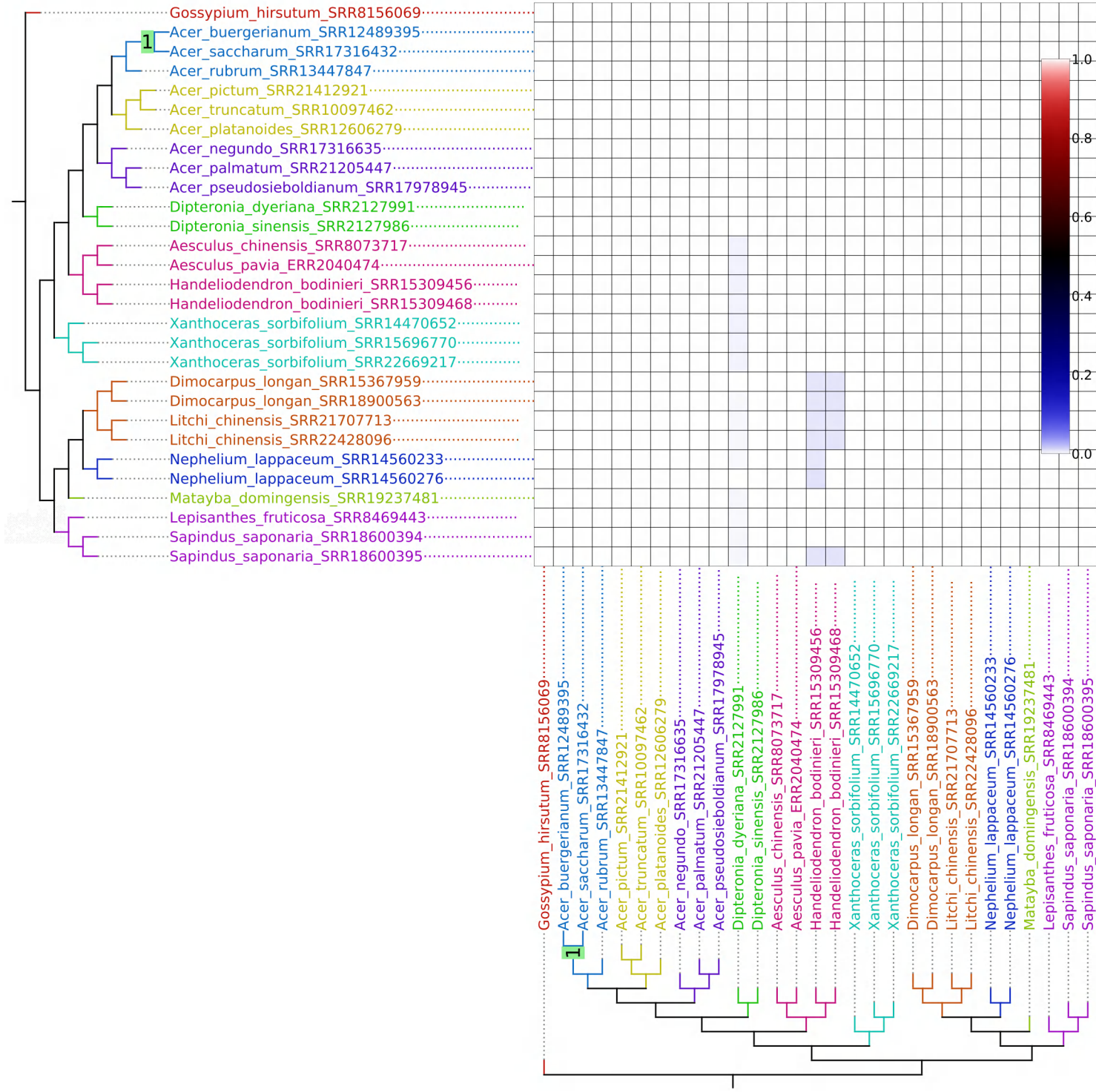

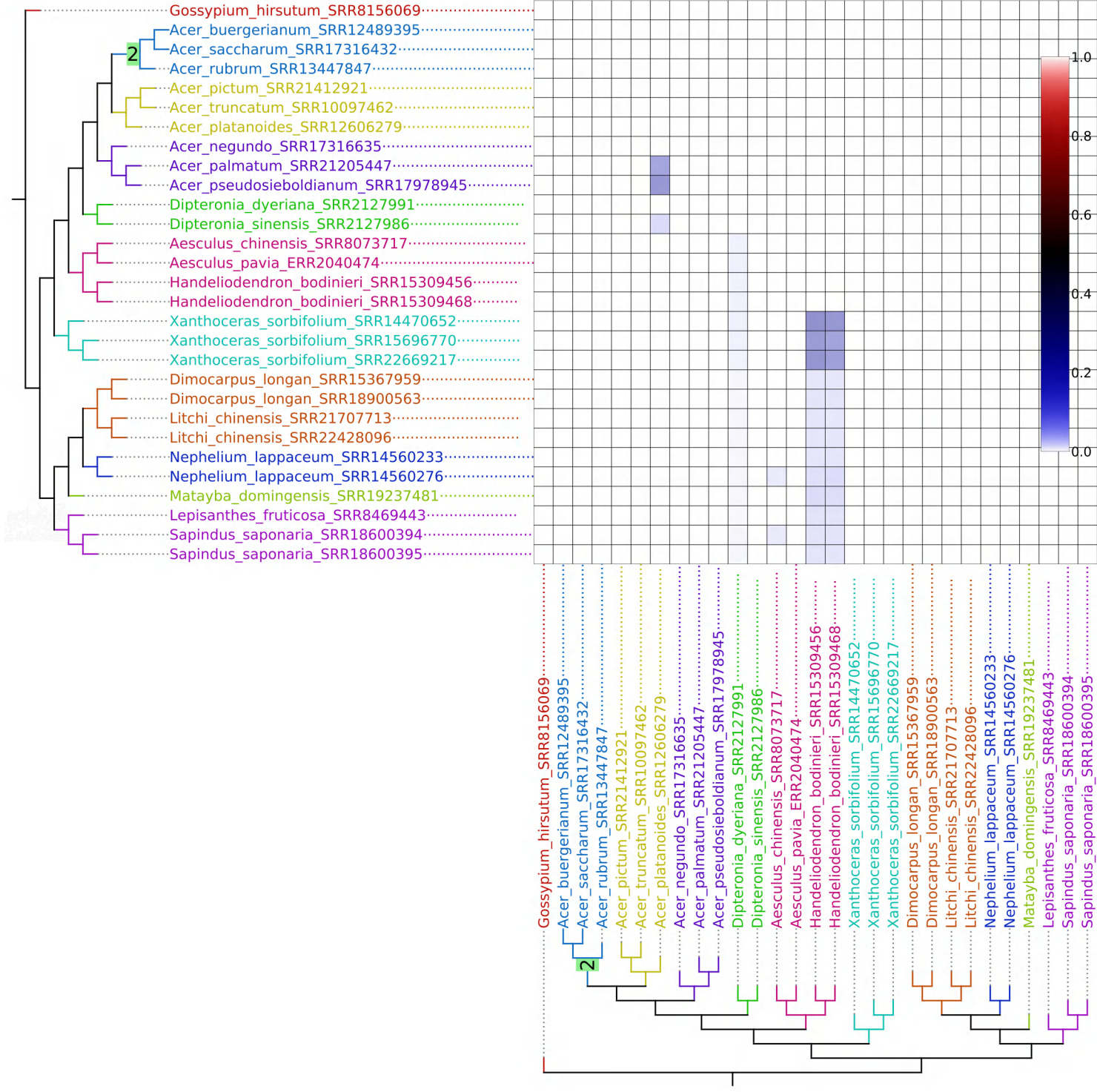

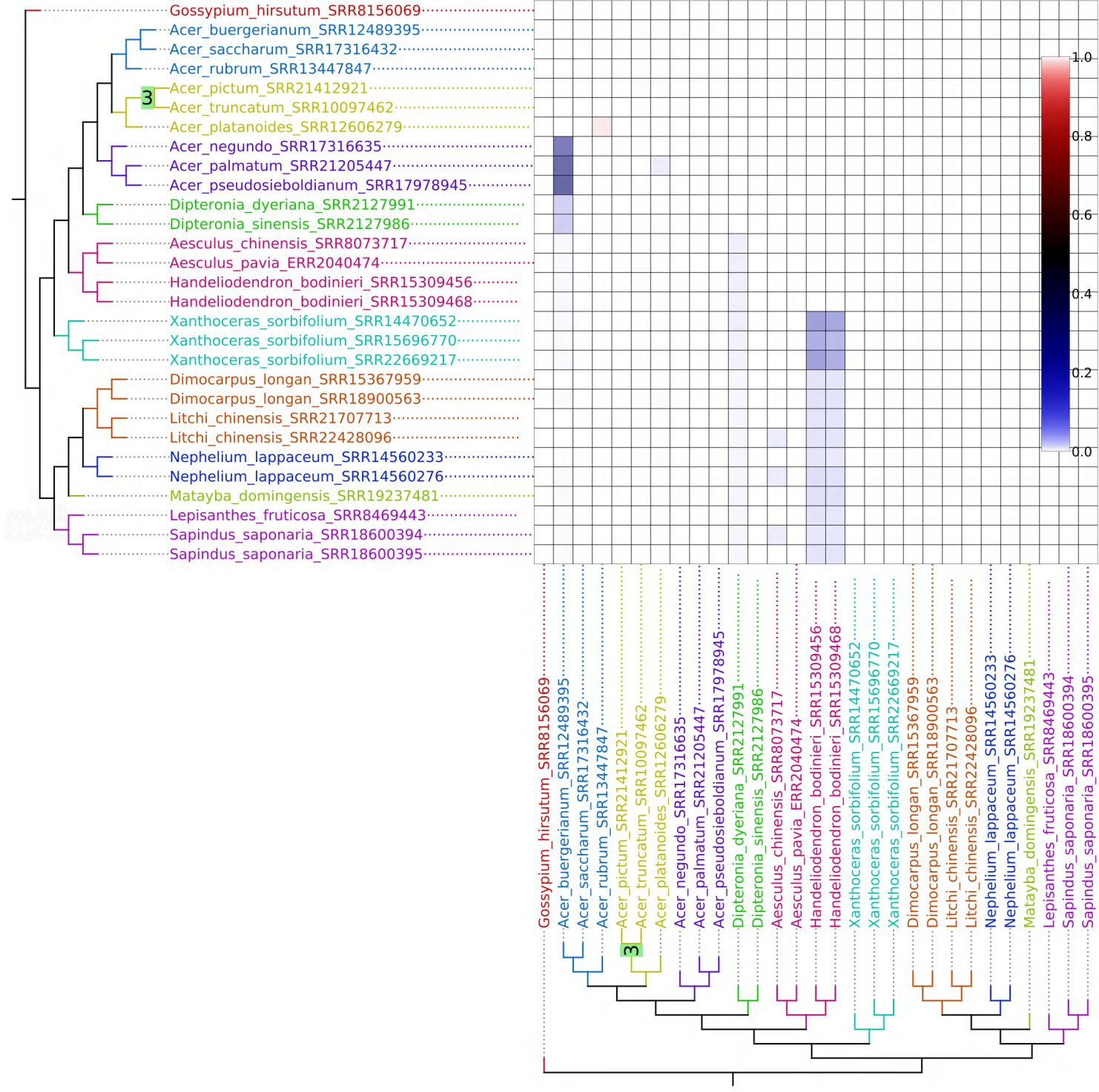

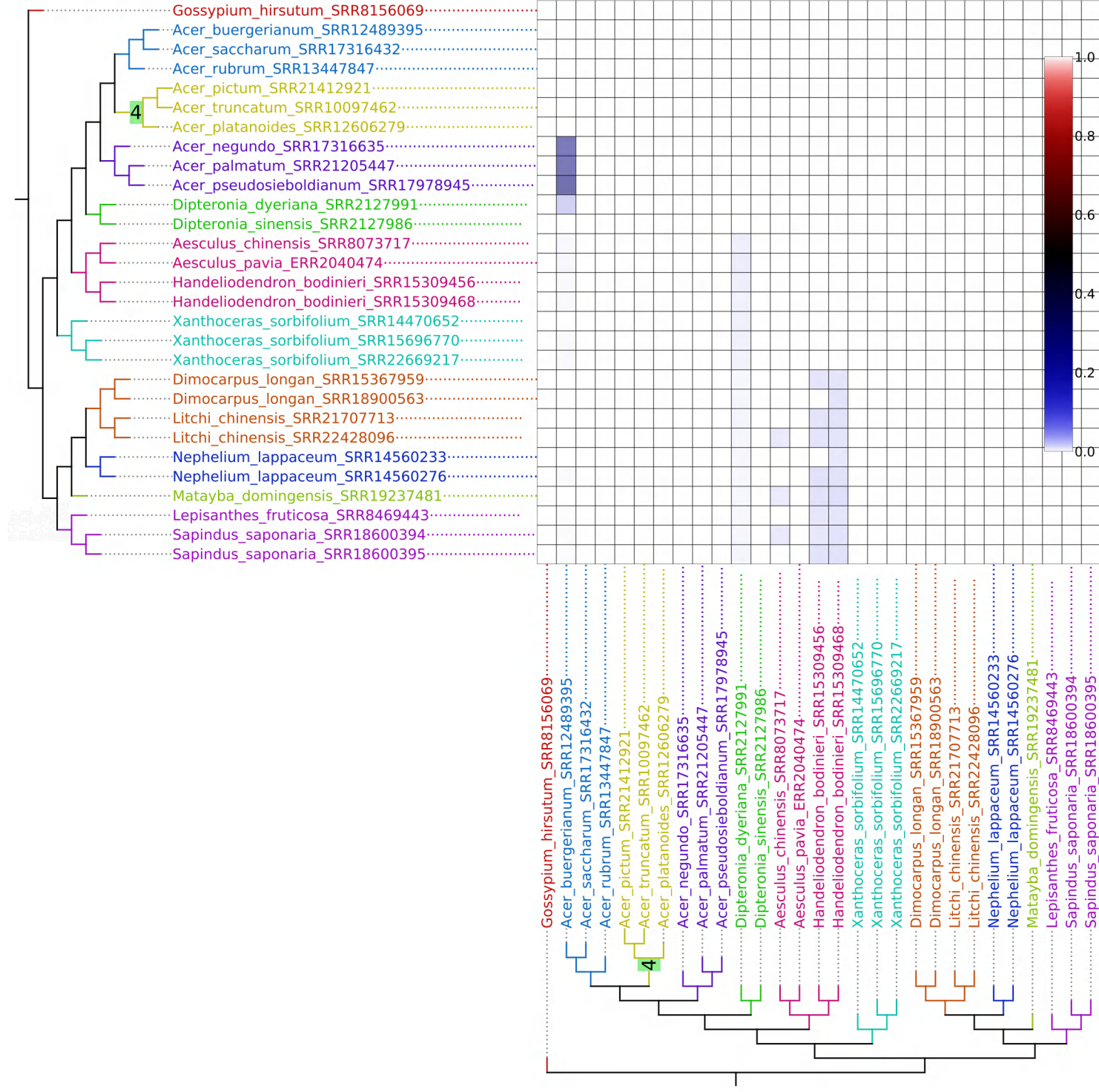

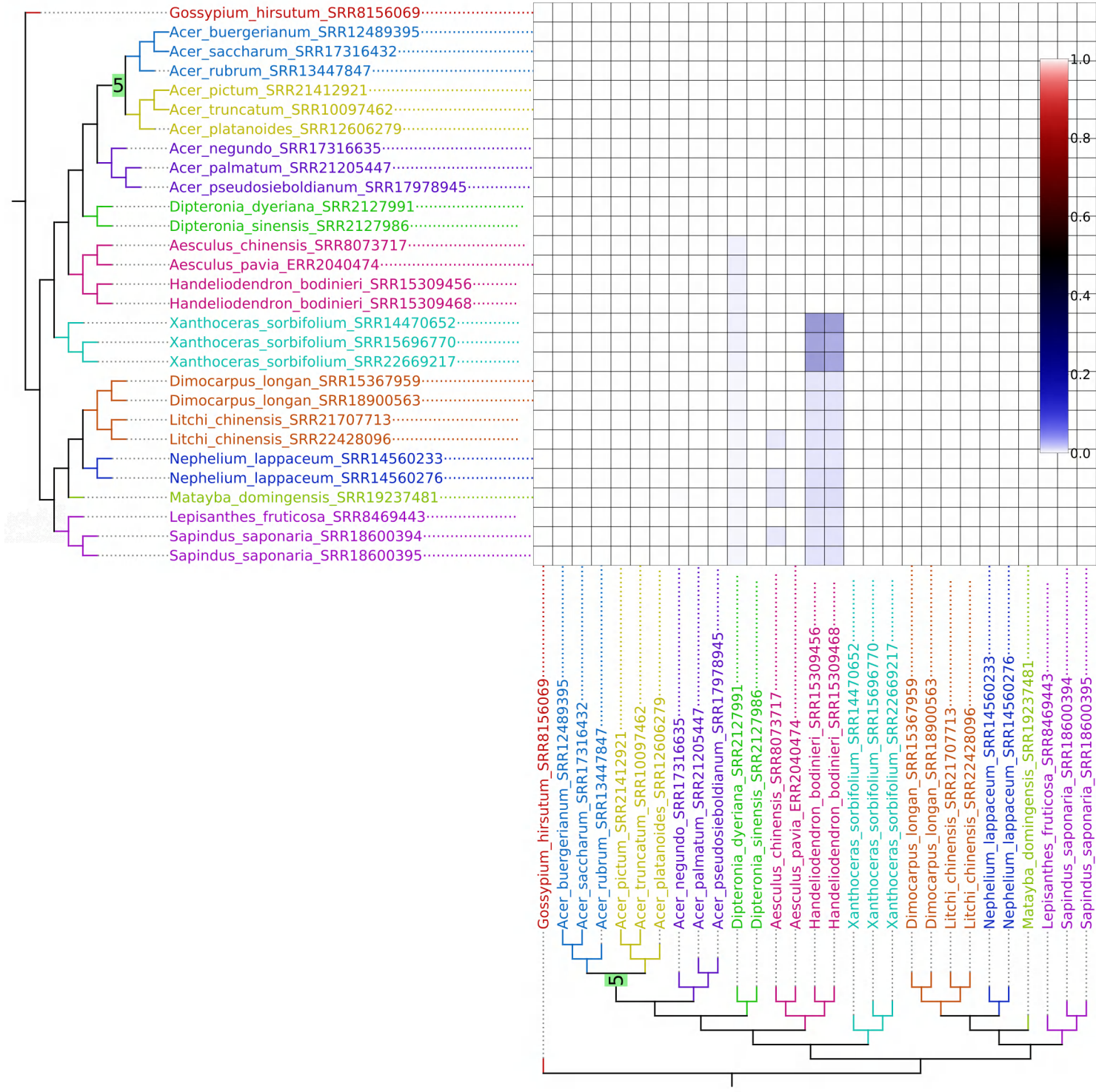

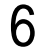

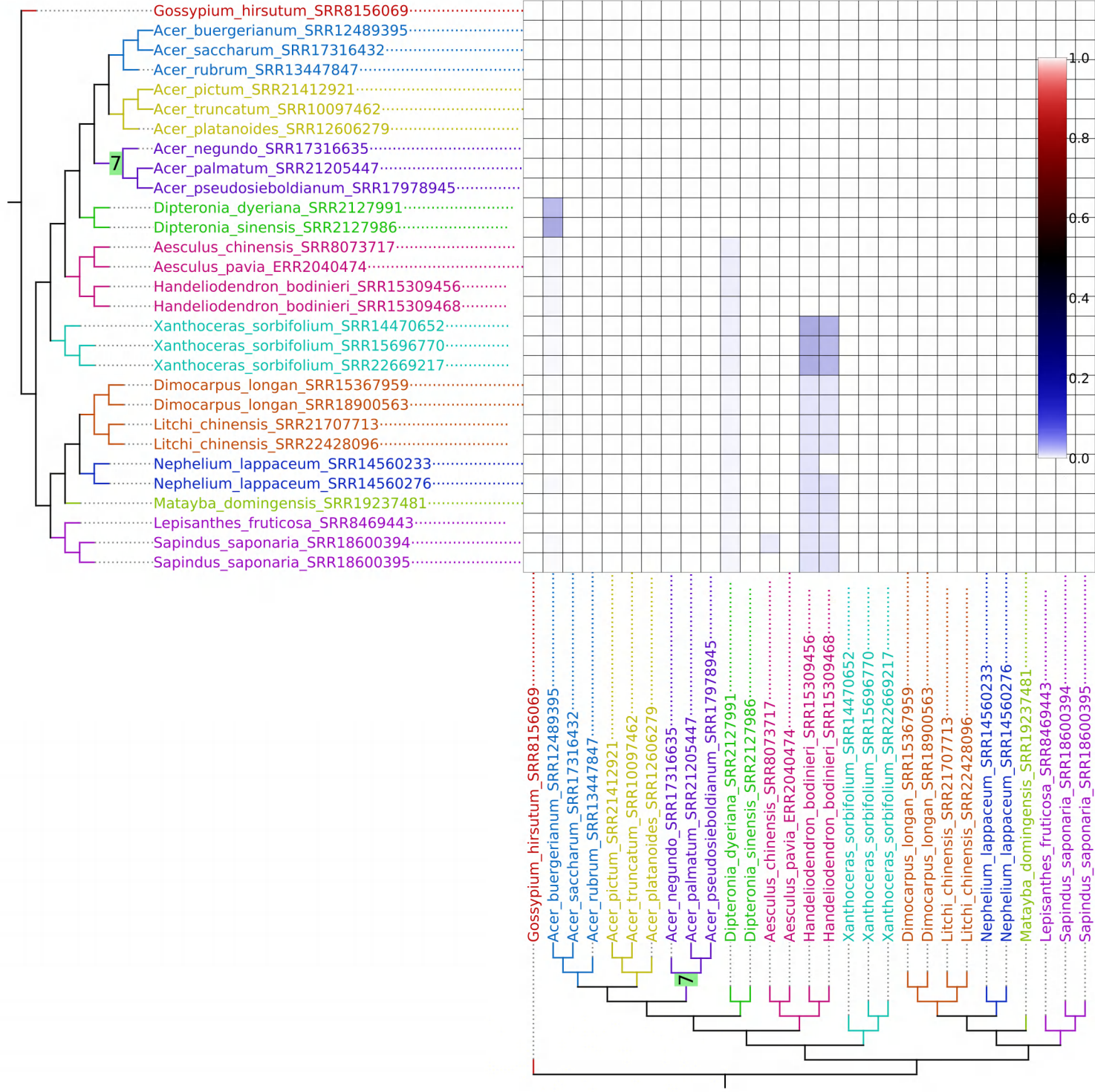

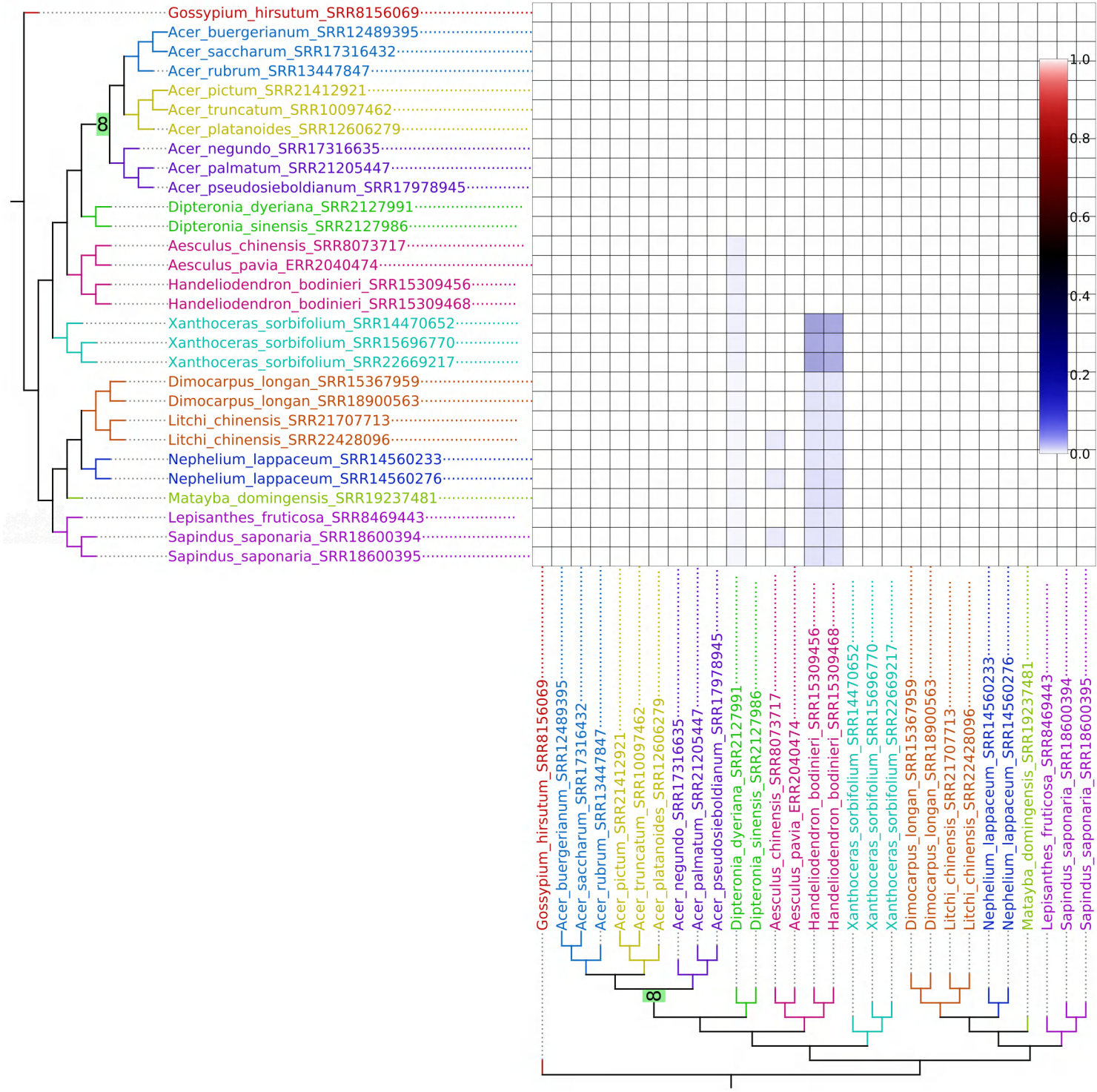

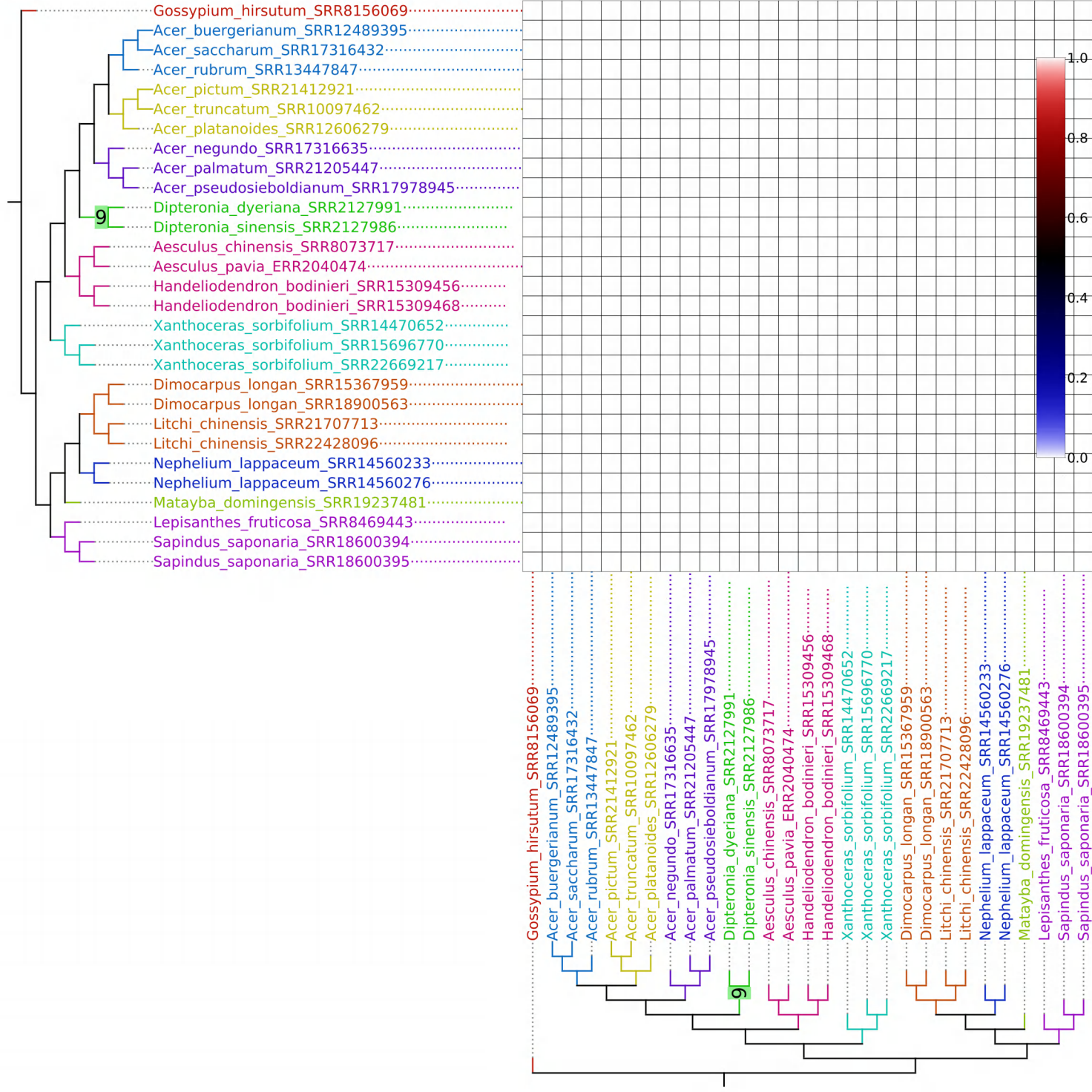

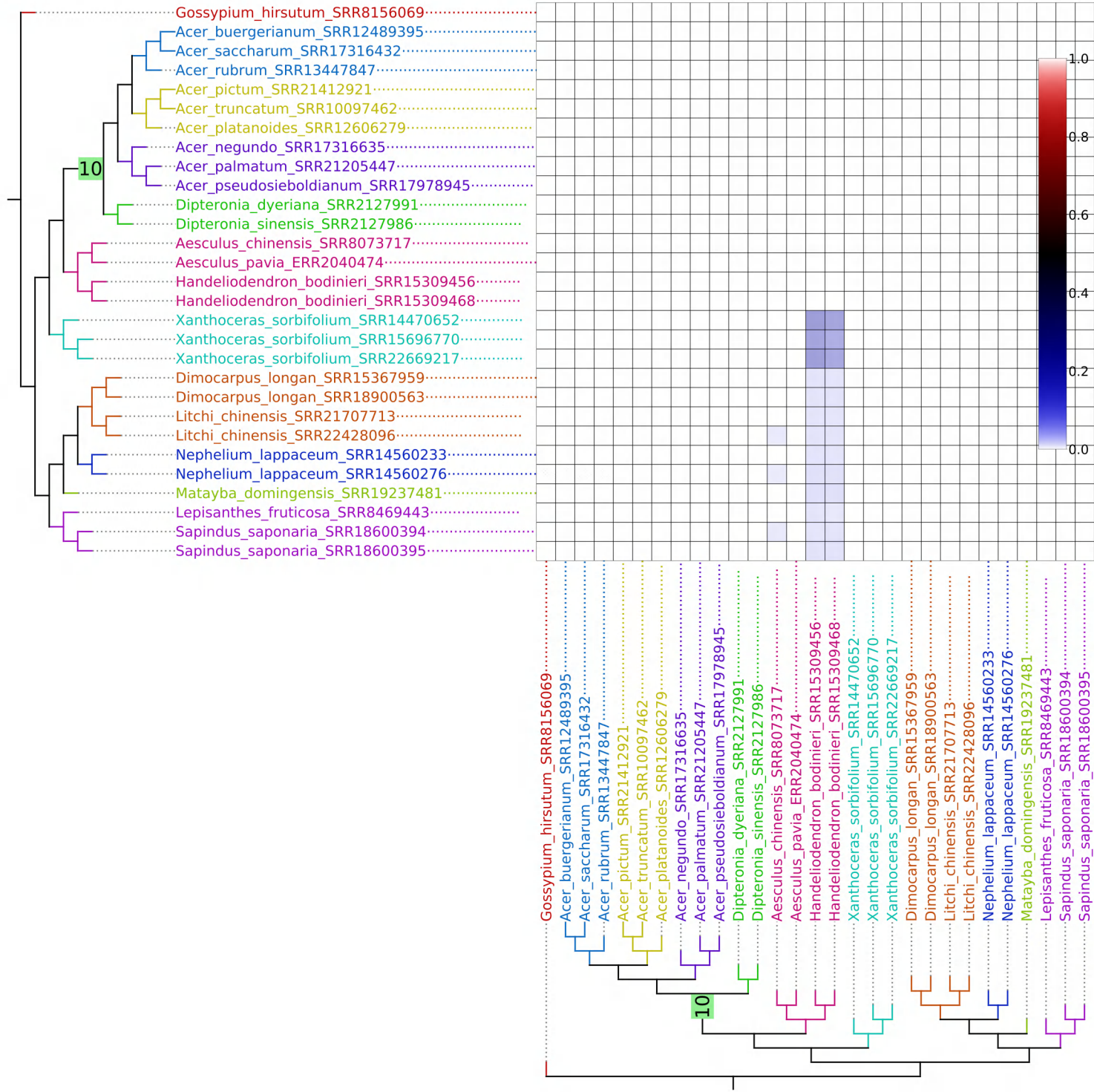

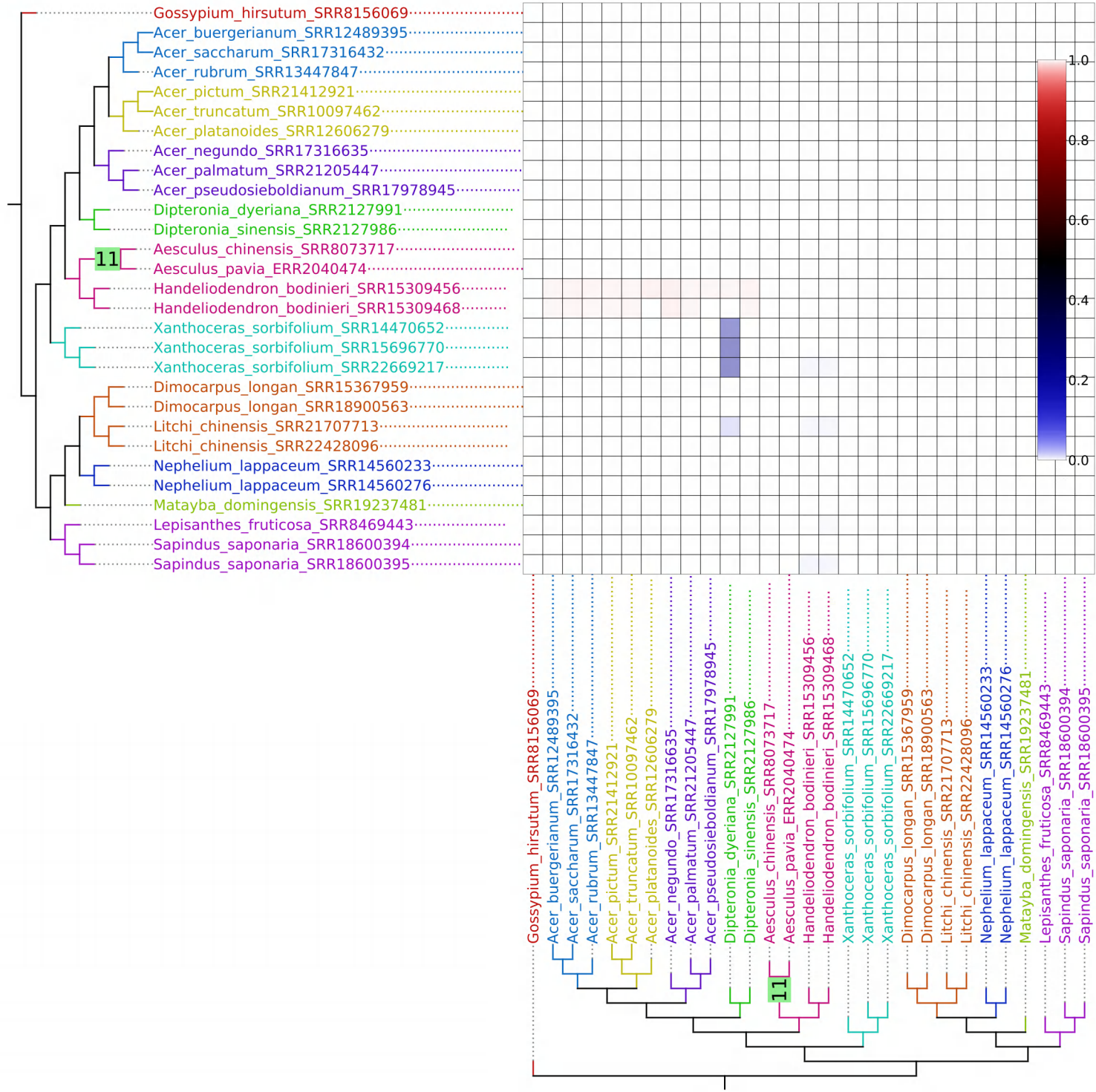

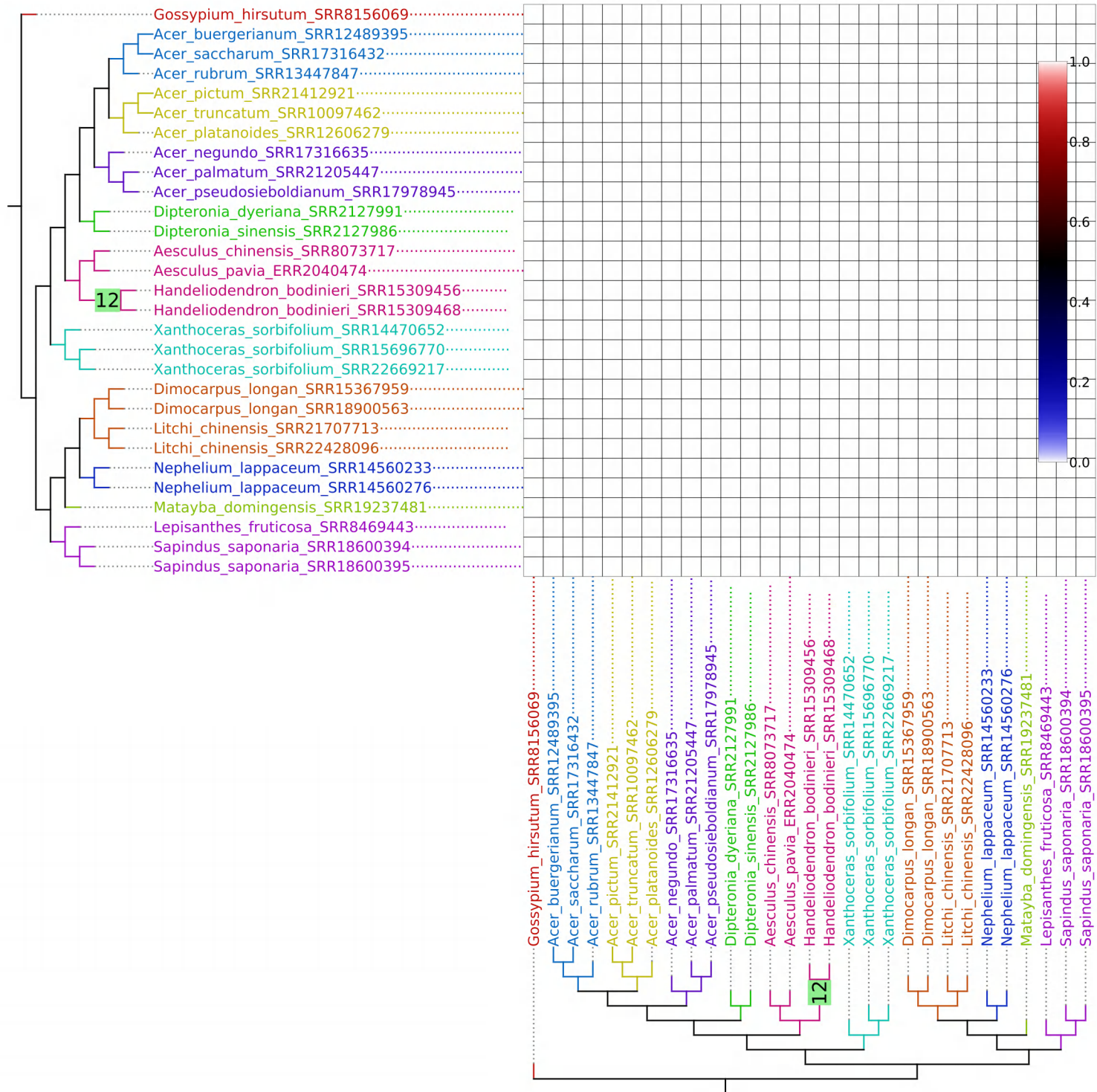

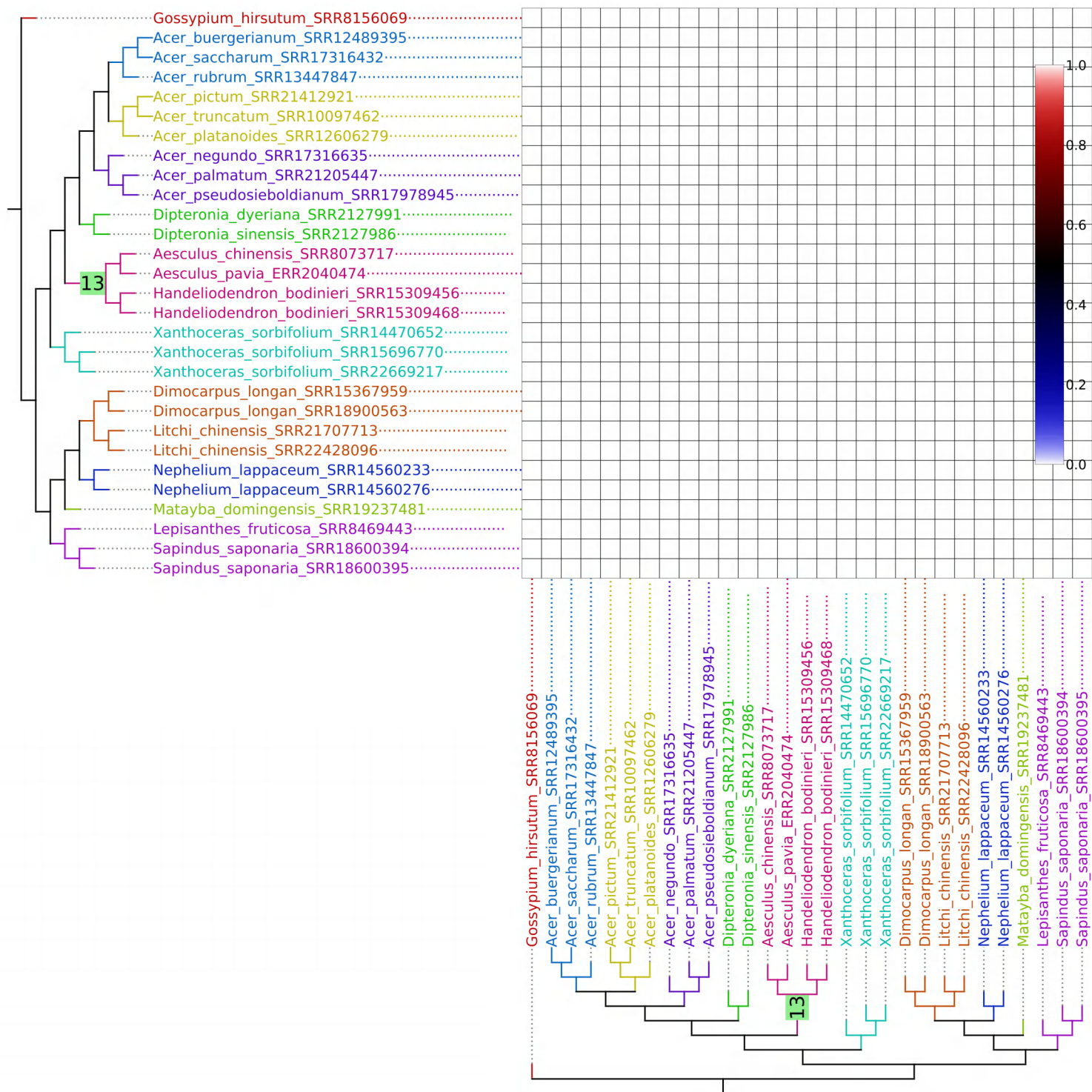

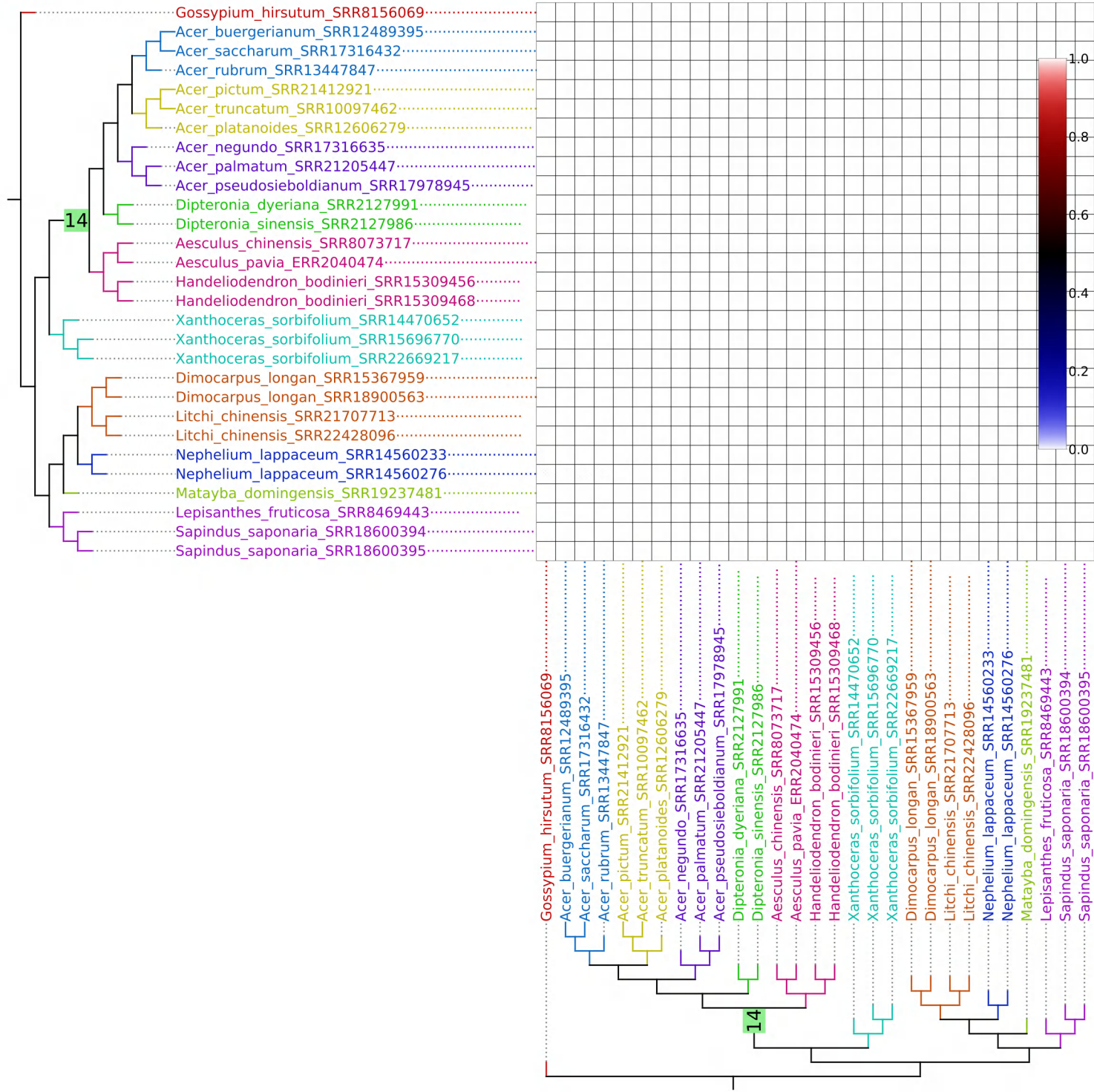

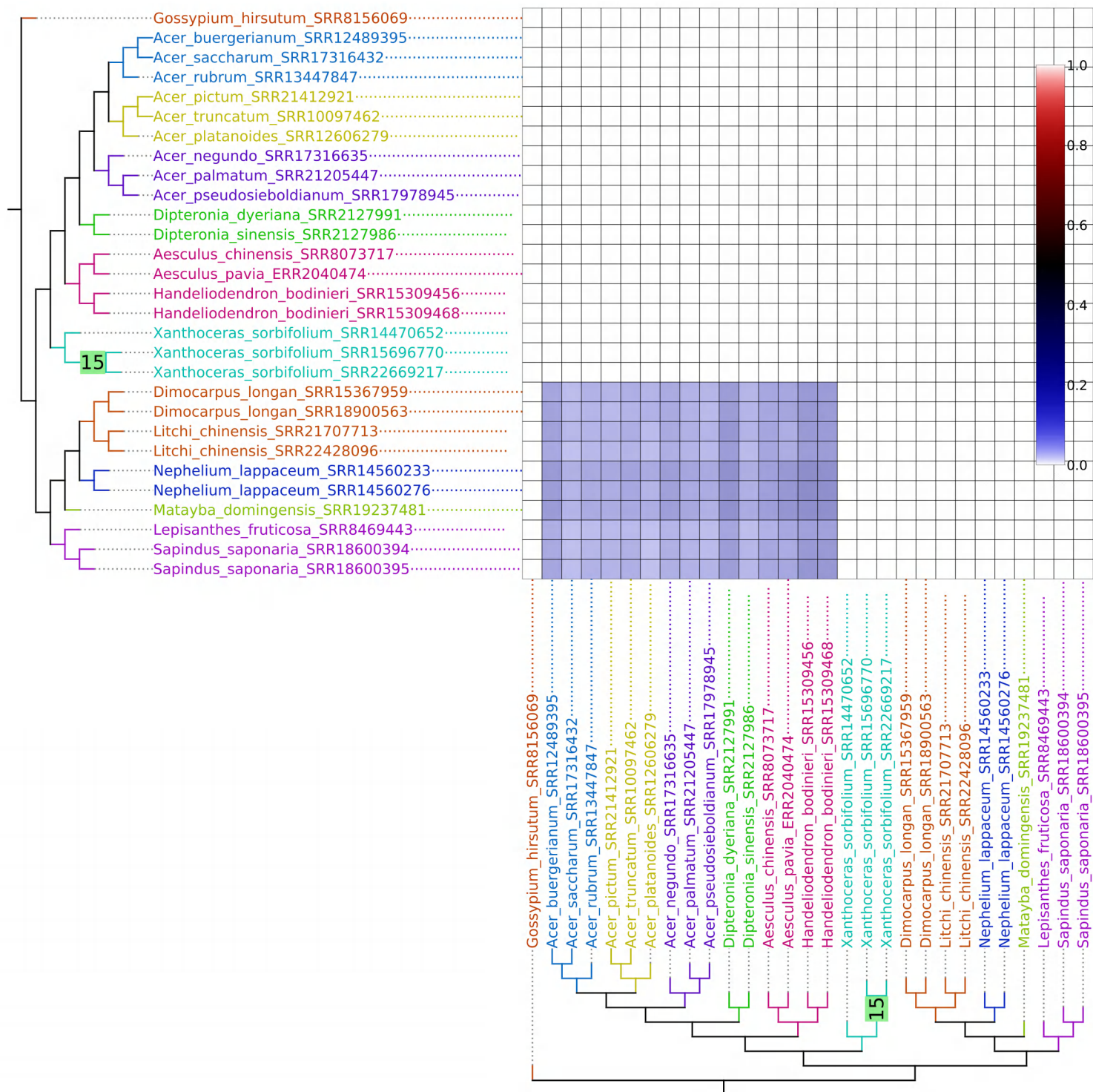

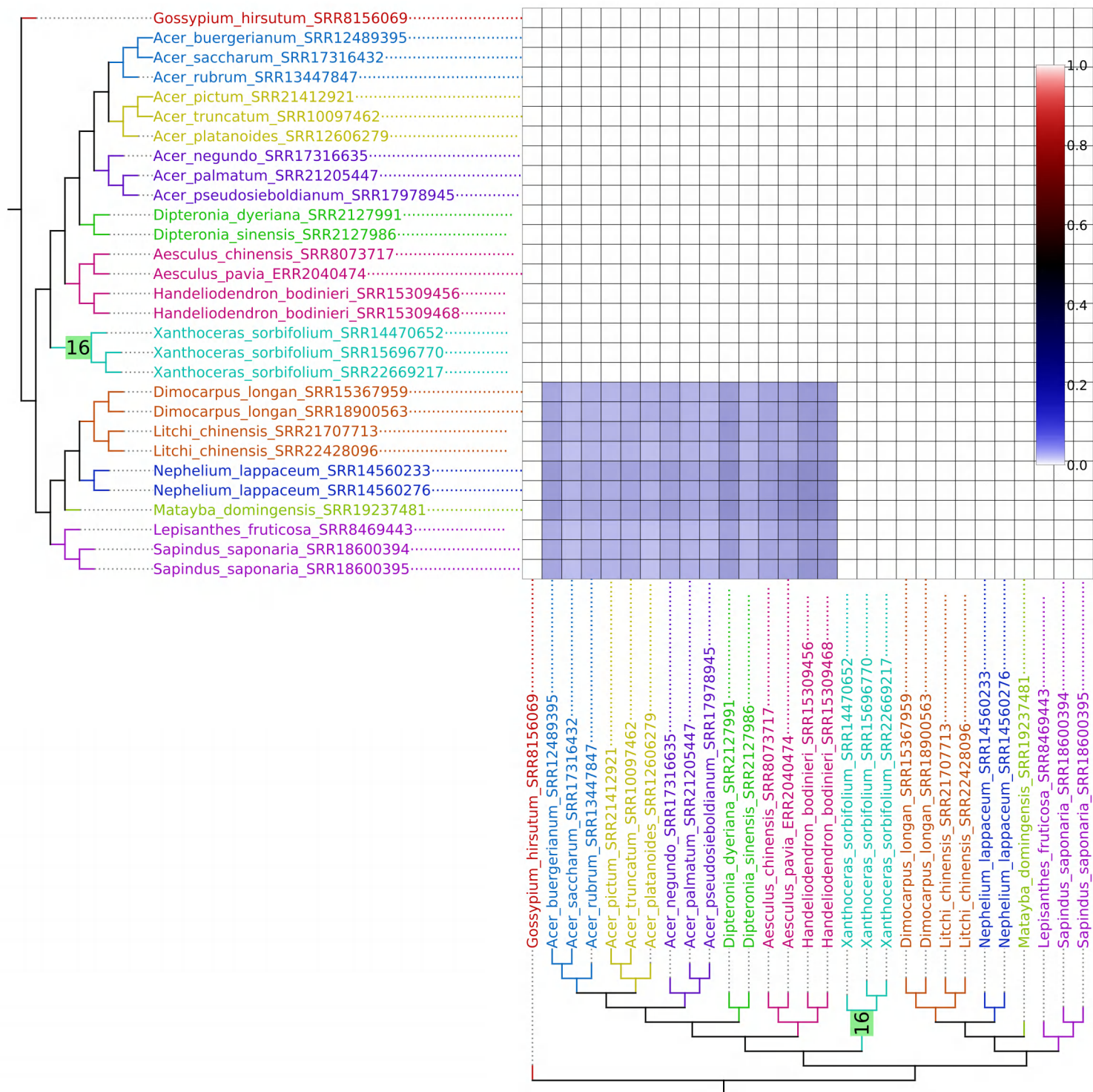

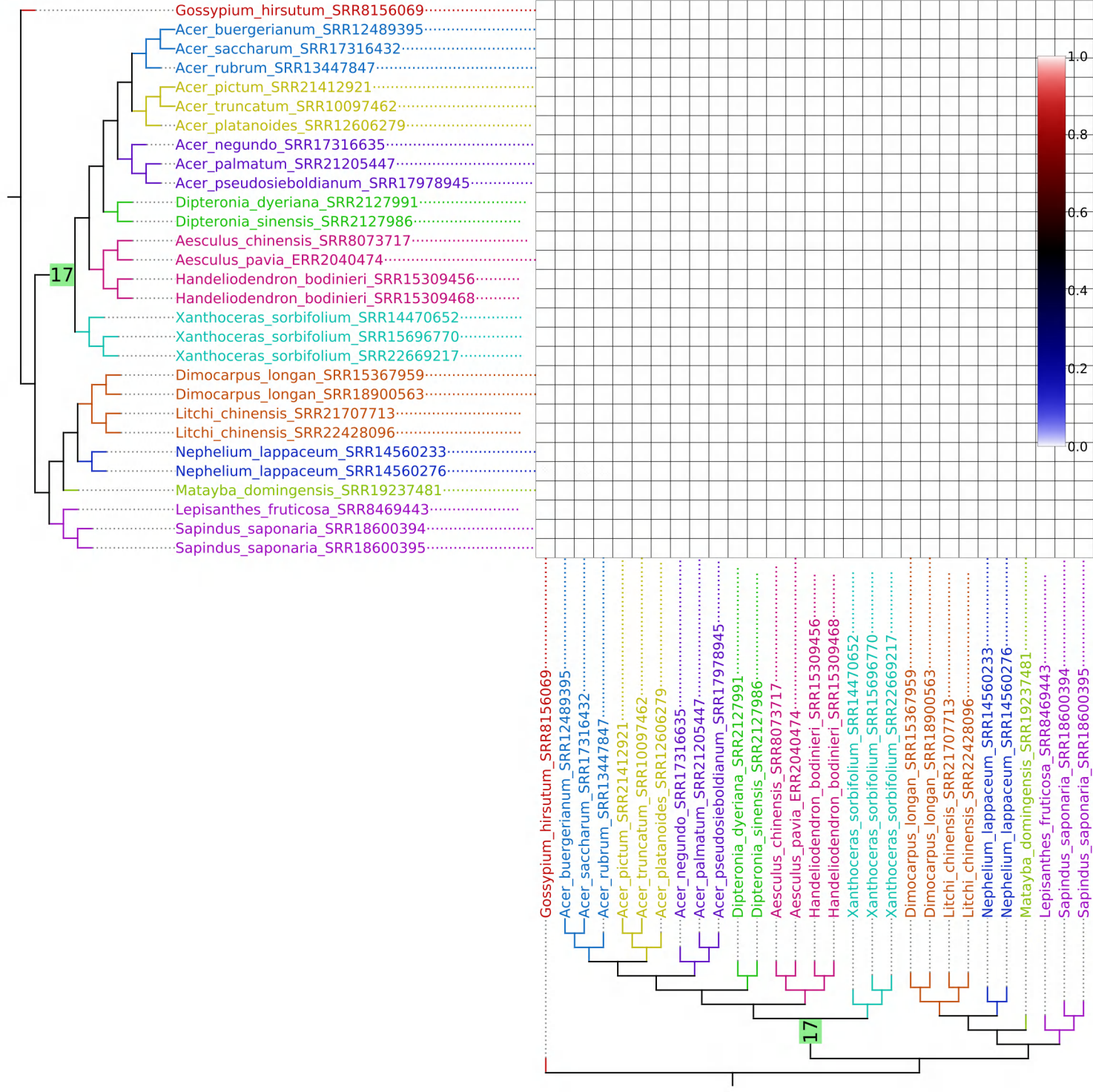

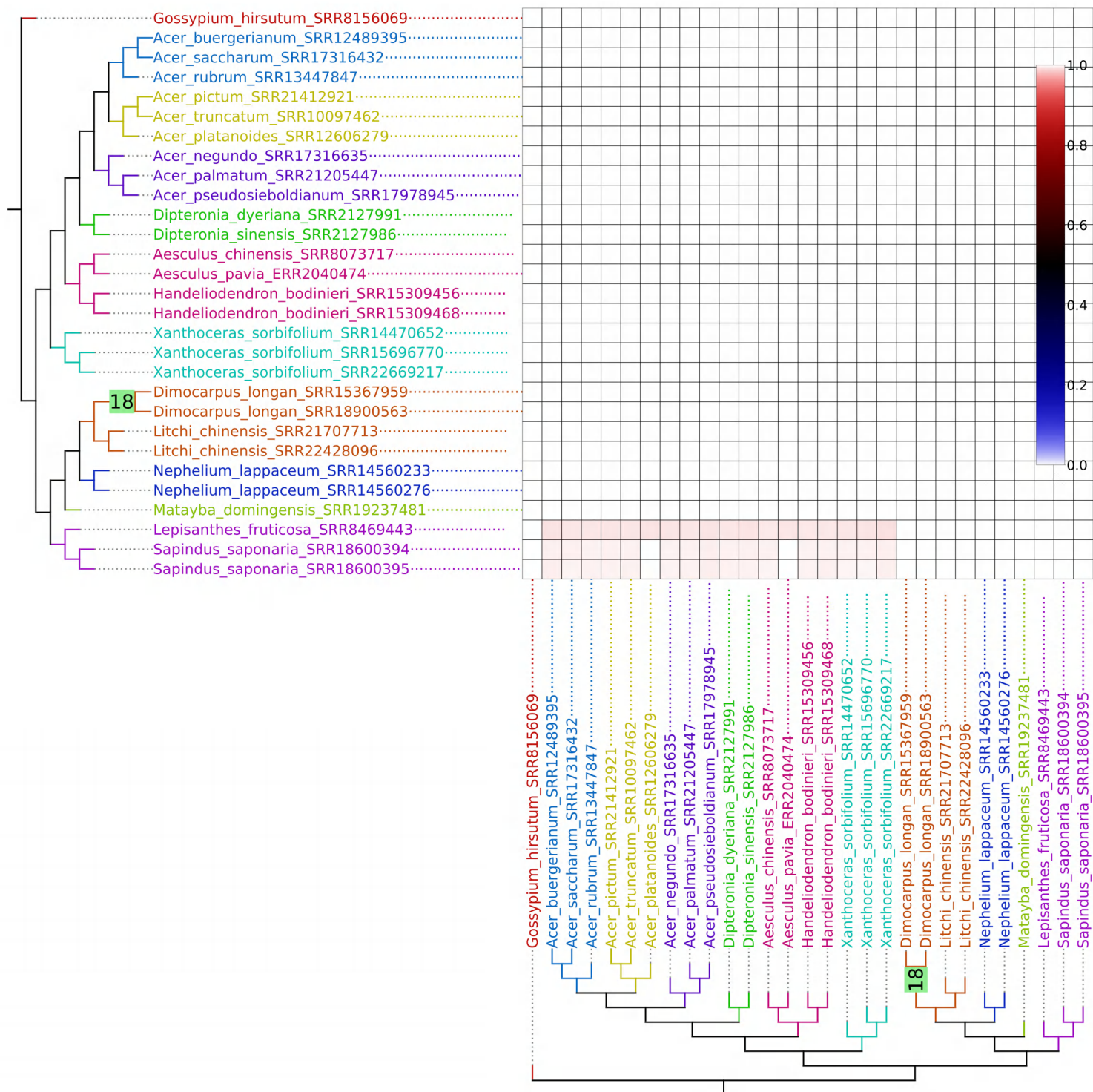

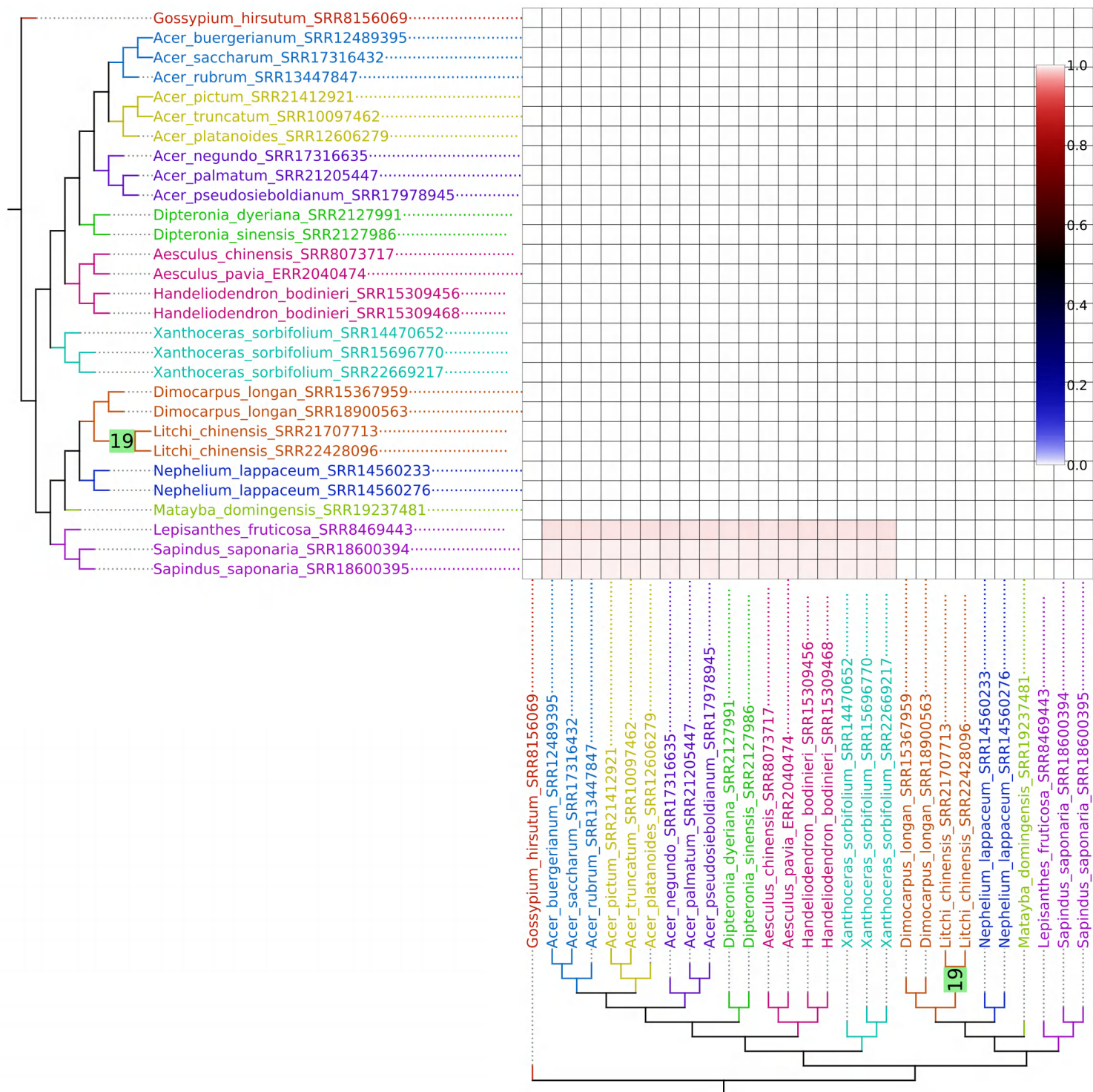

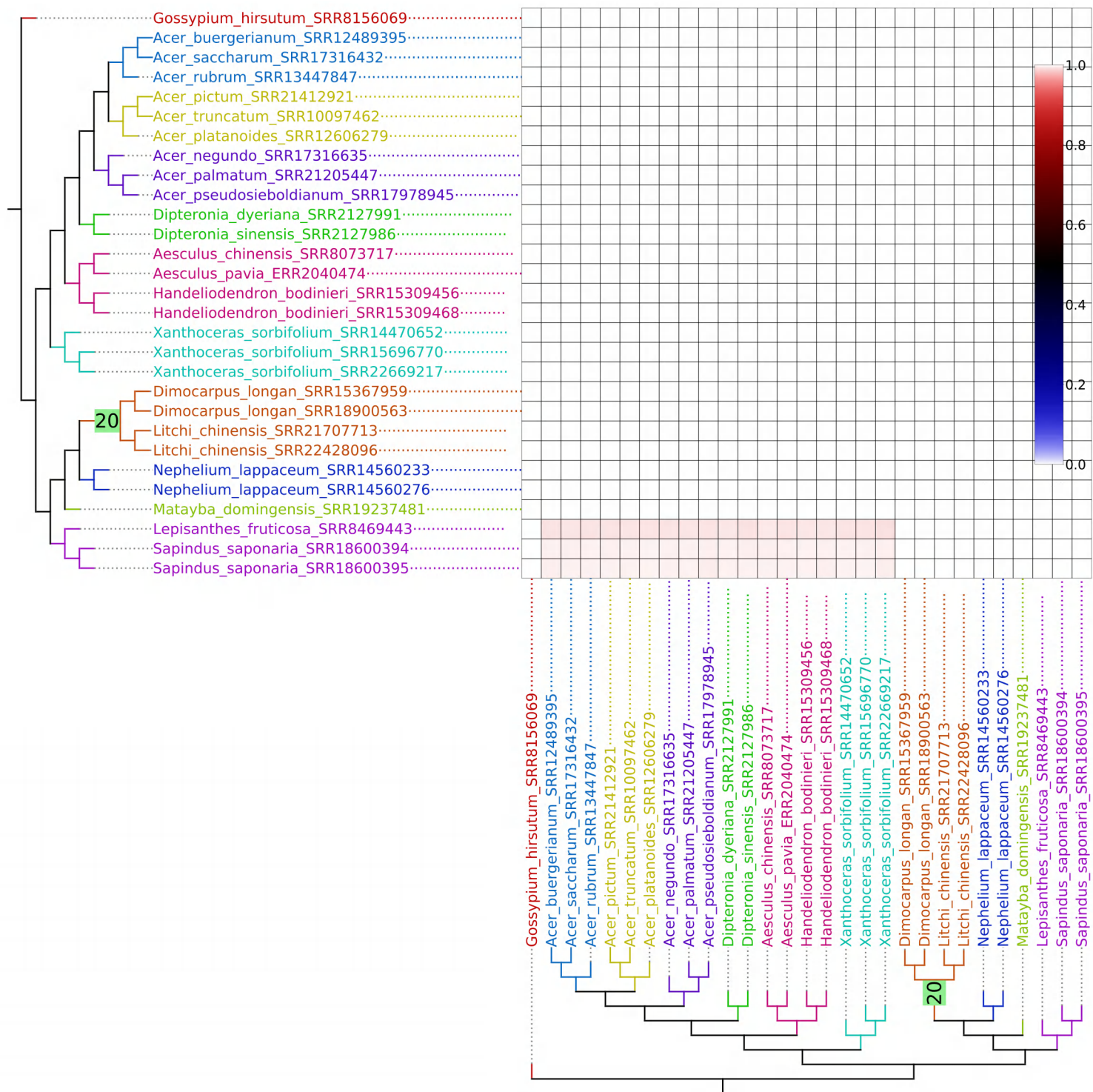

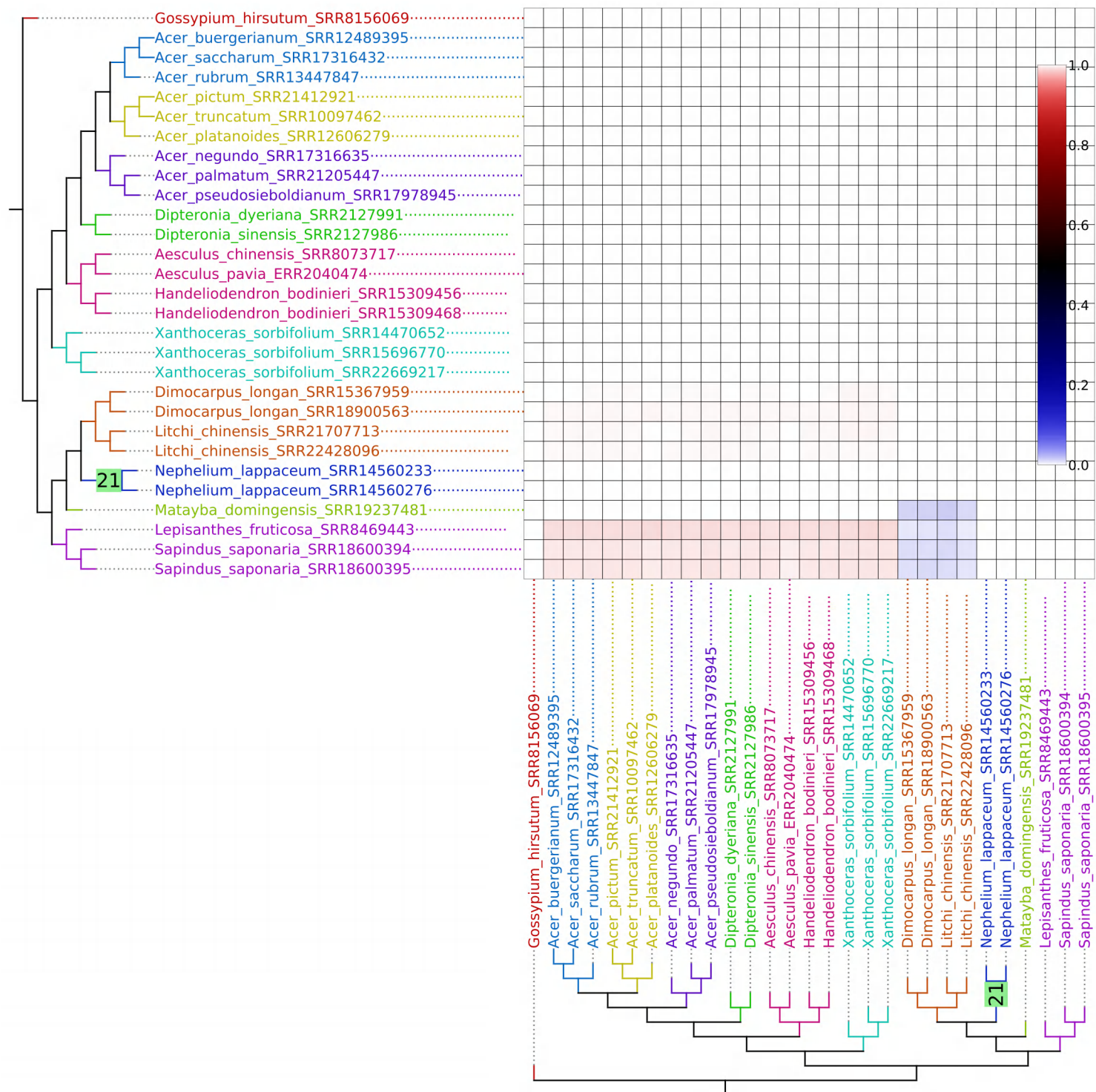

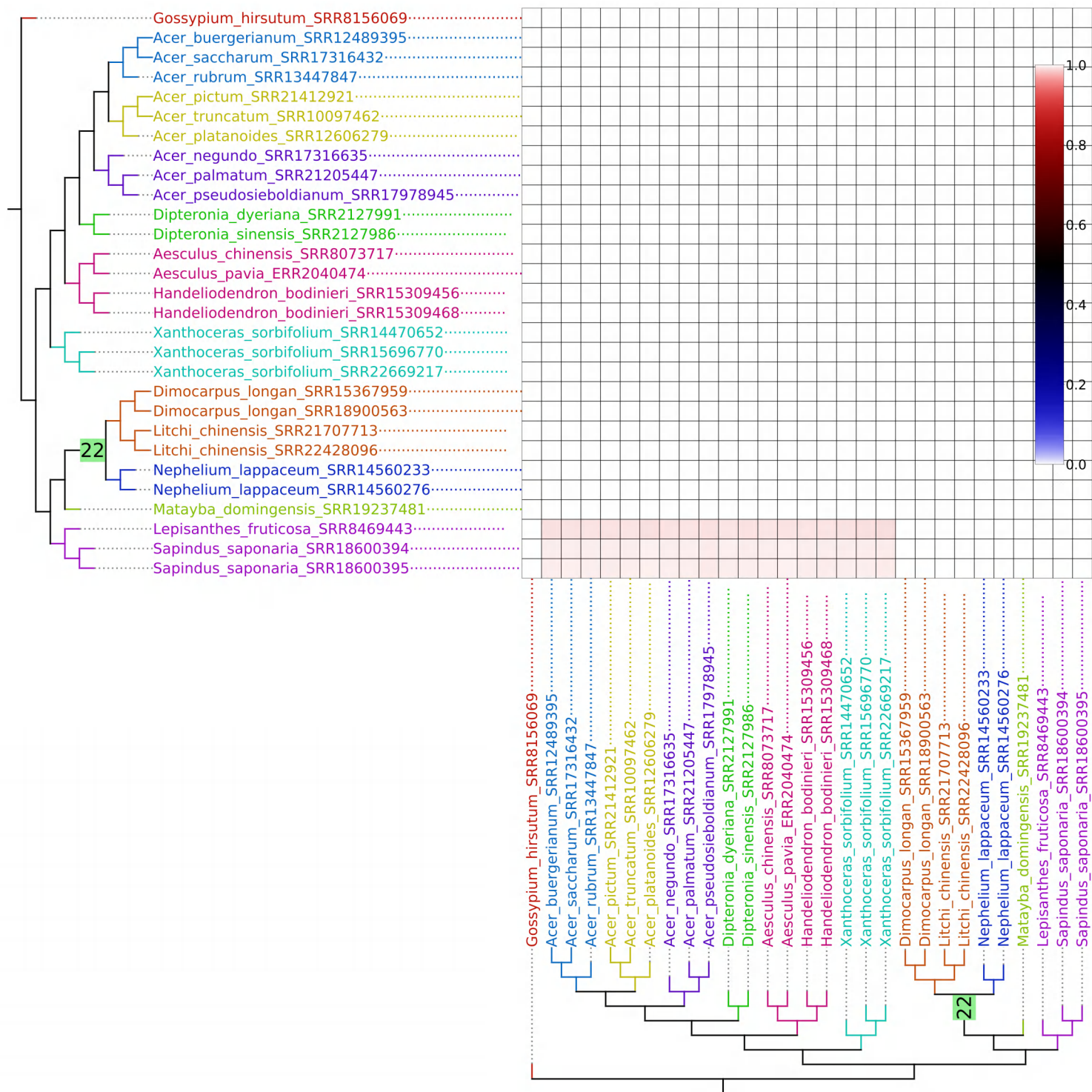

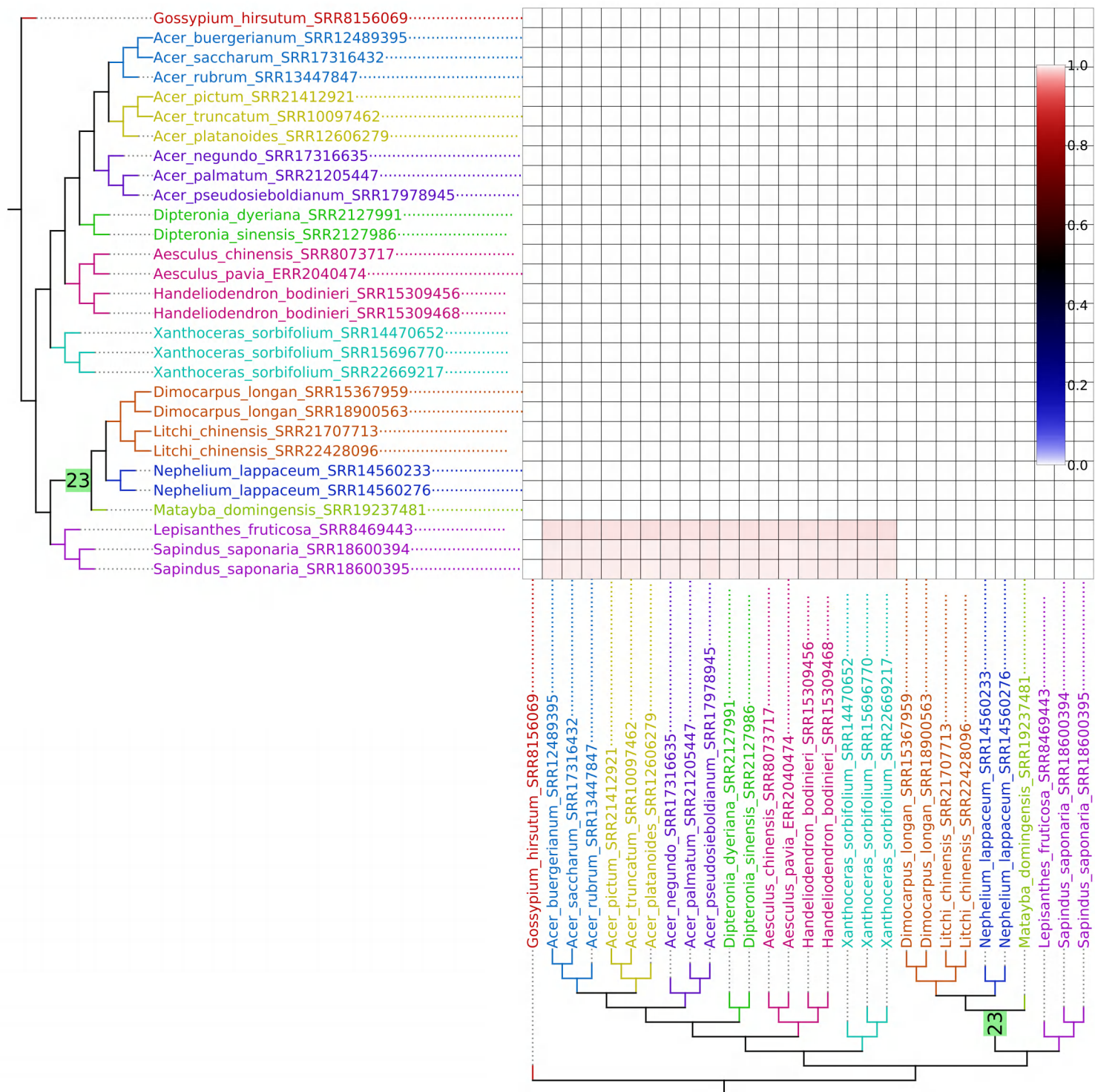

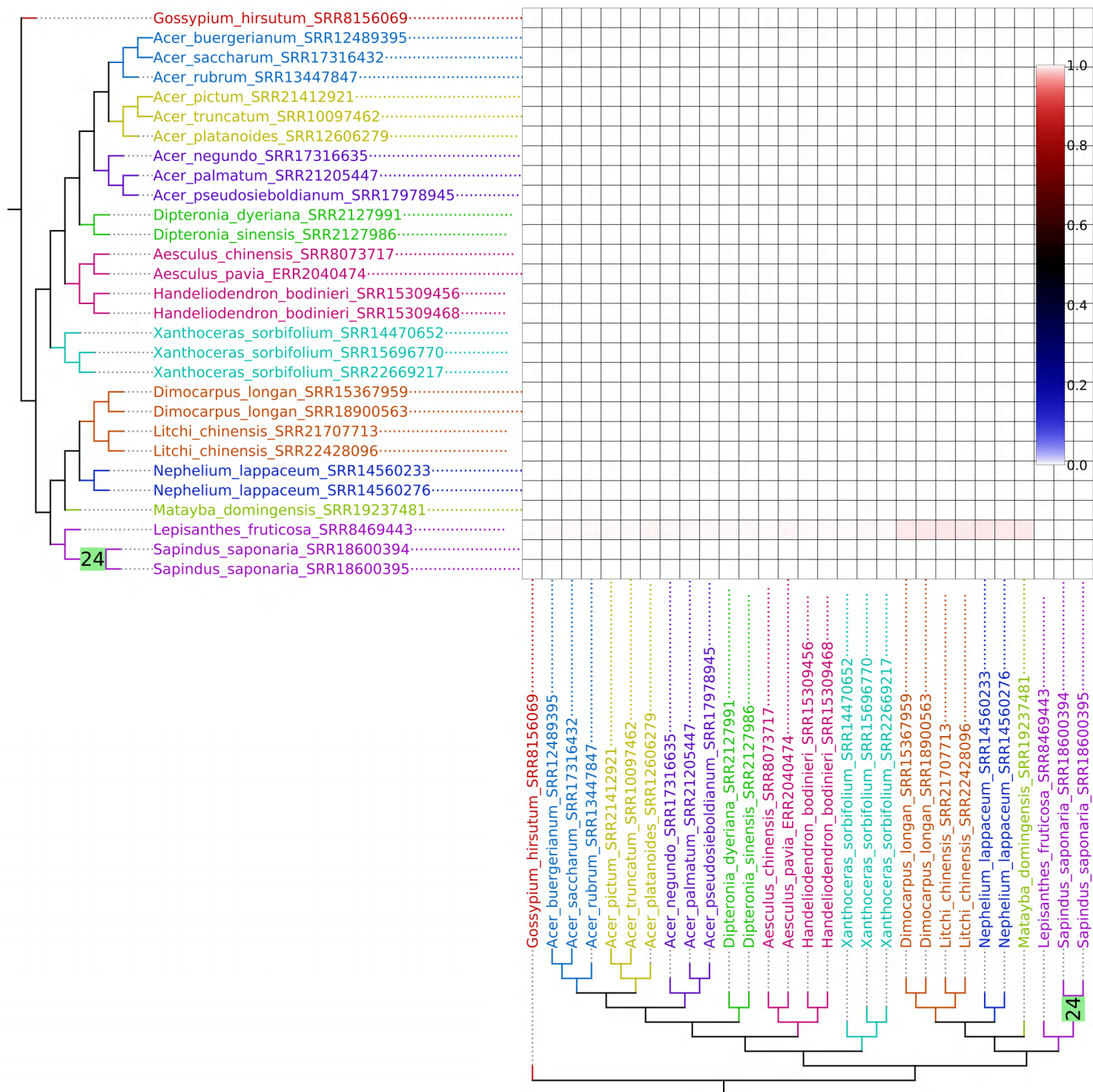

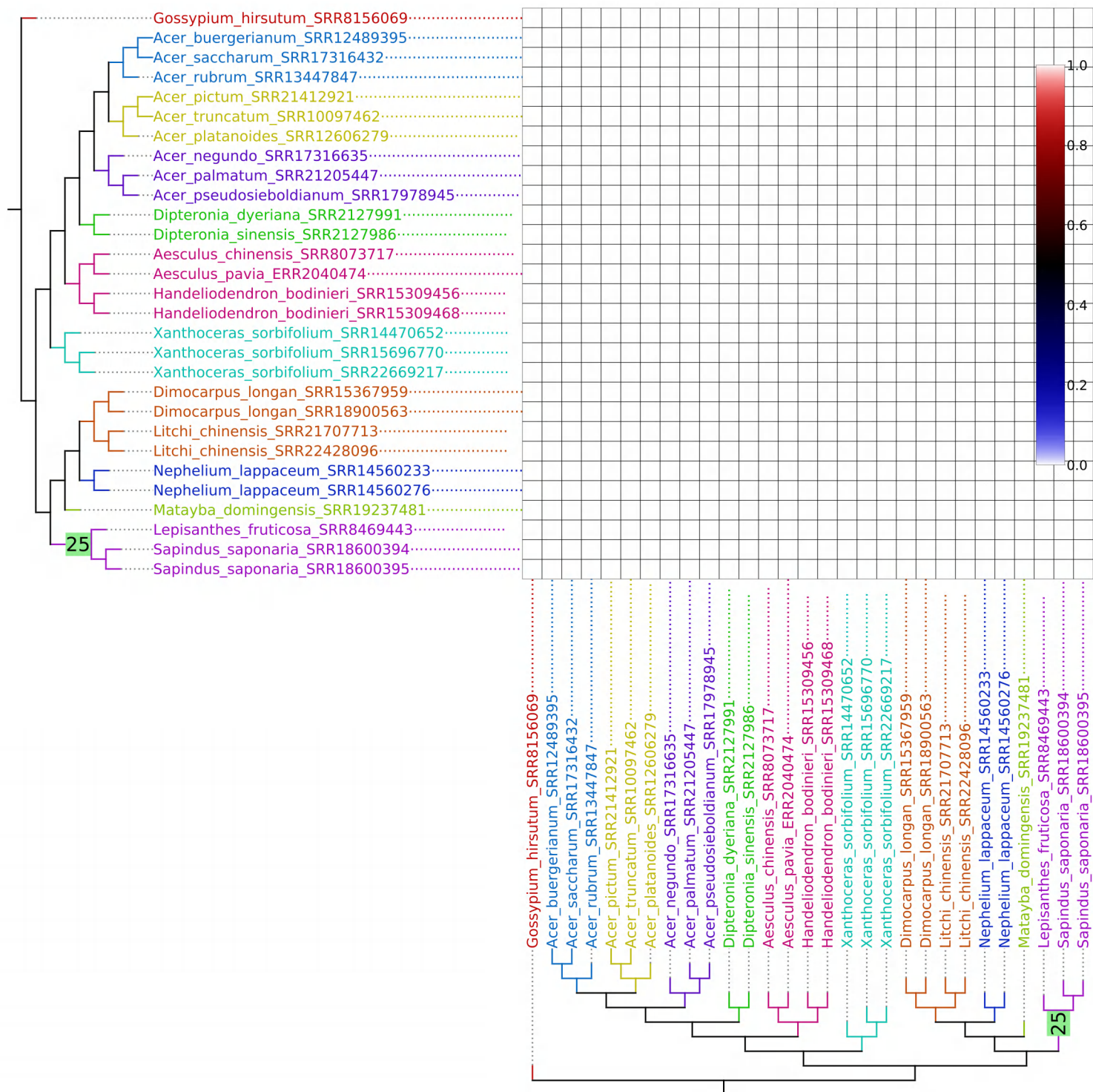

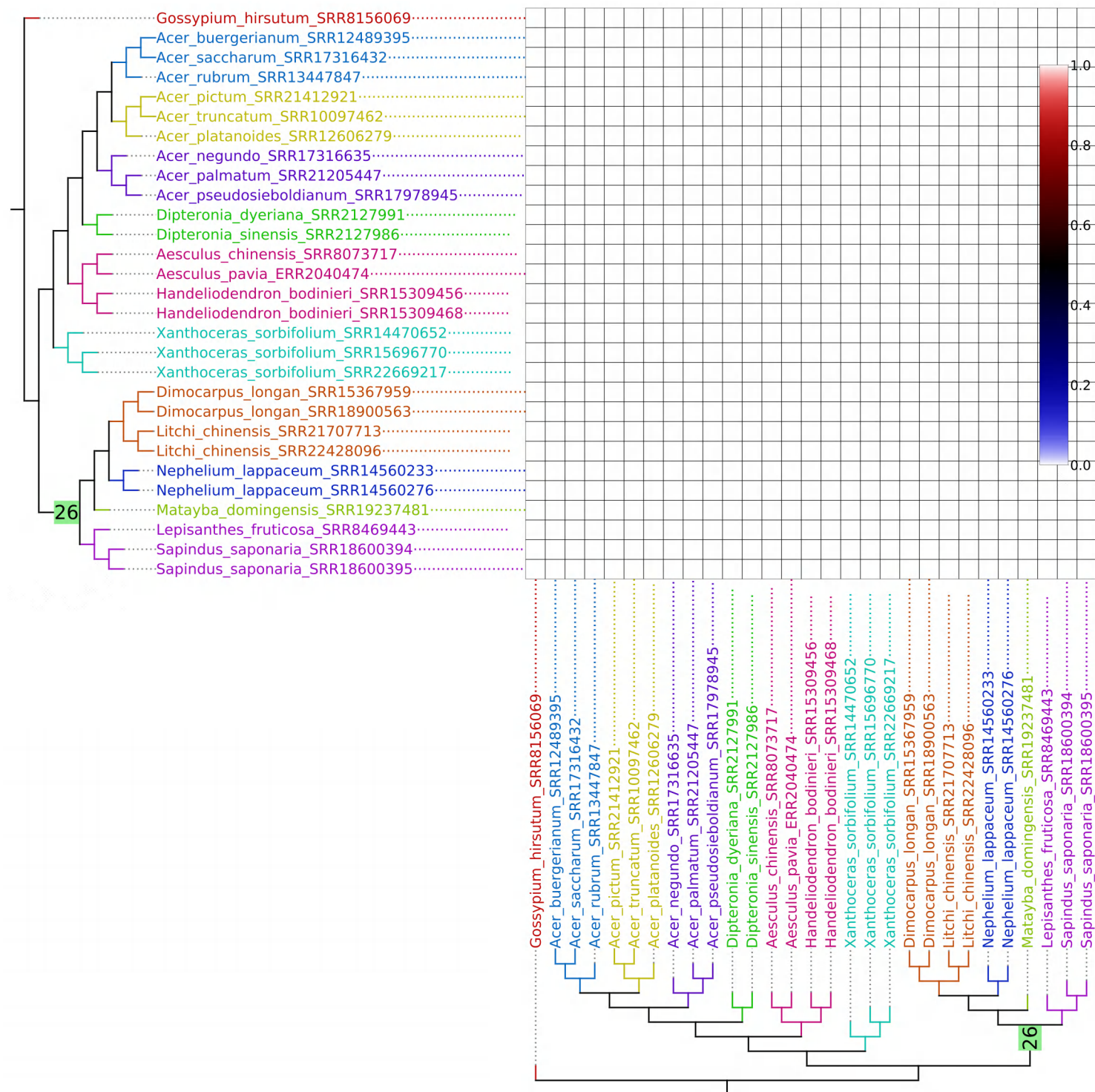

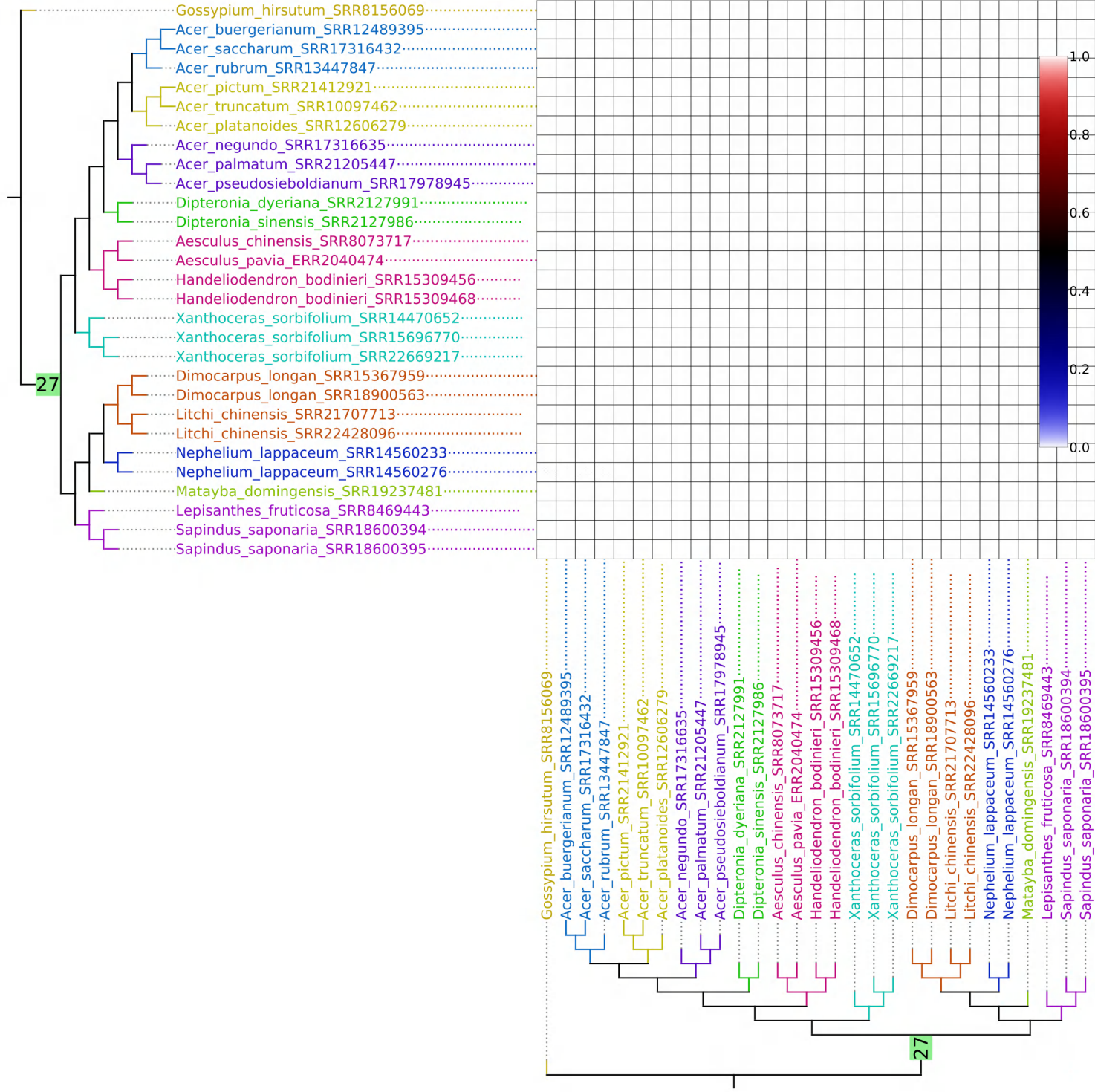

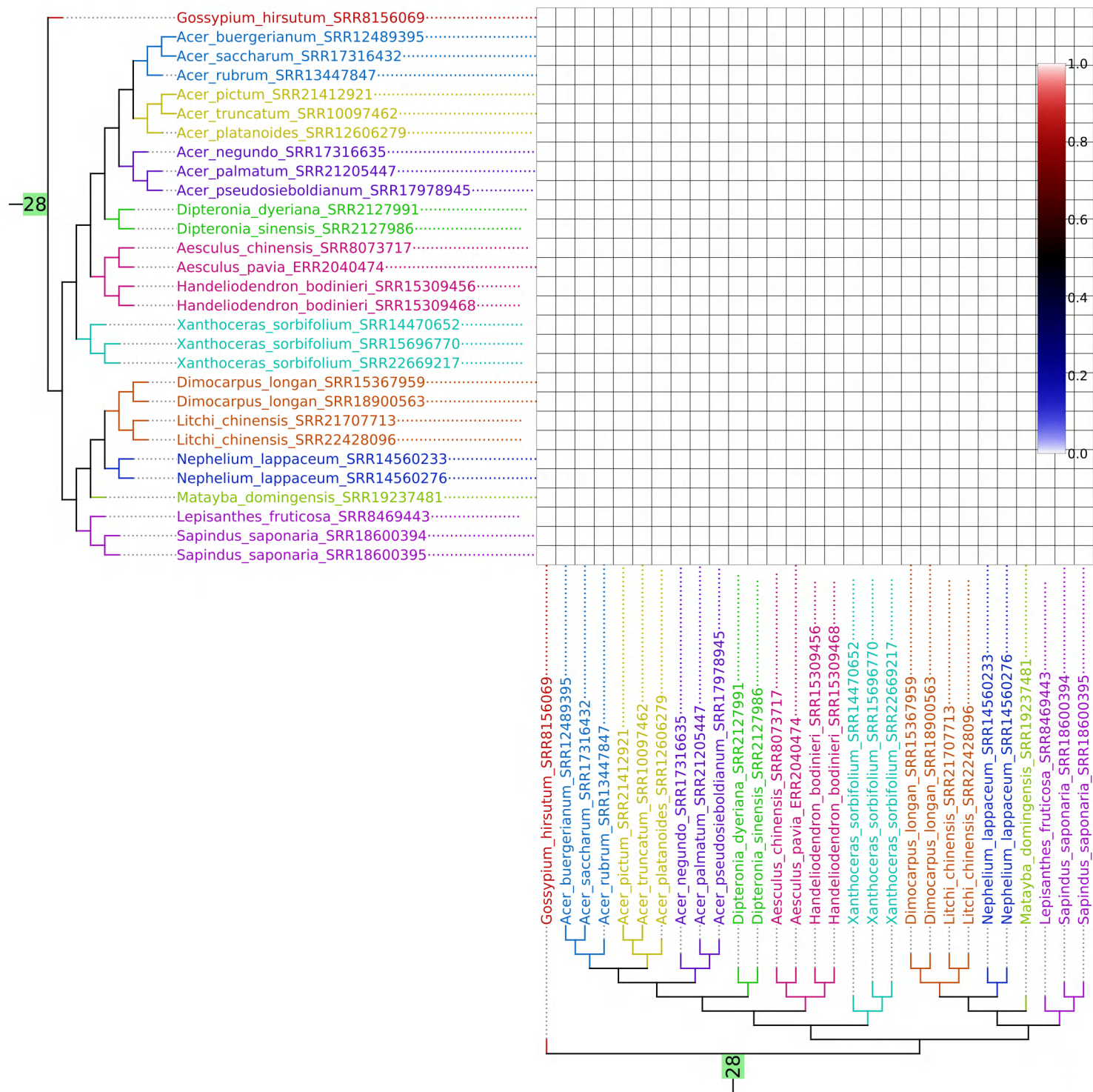

Supplement: Supplementary file 1 [file ijms-26-01581-s001.zip › Figure_S8.pdf]

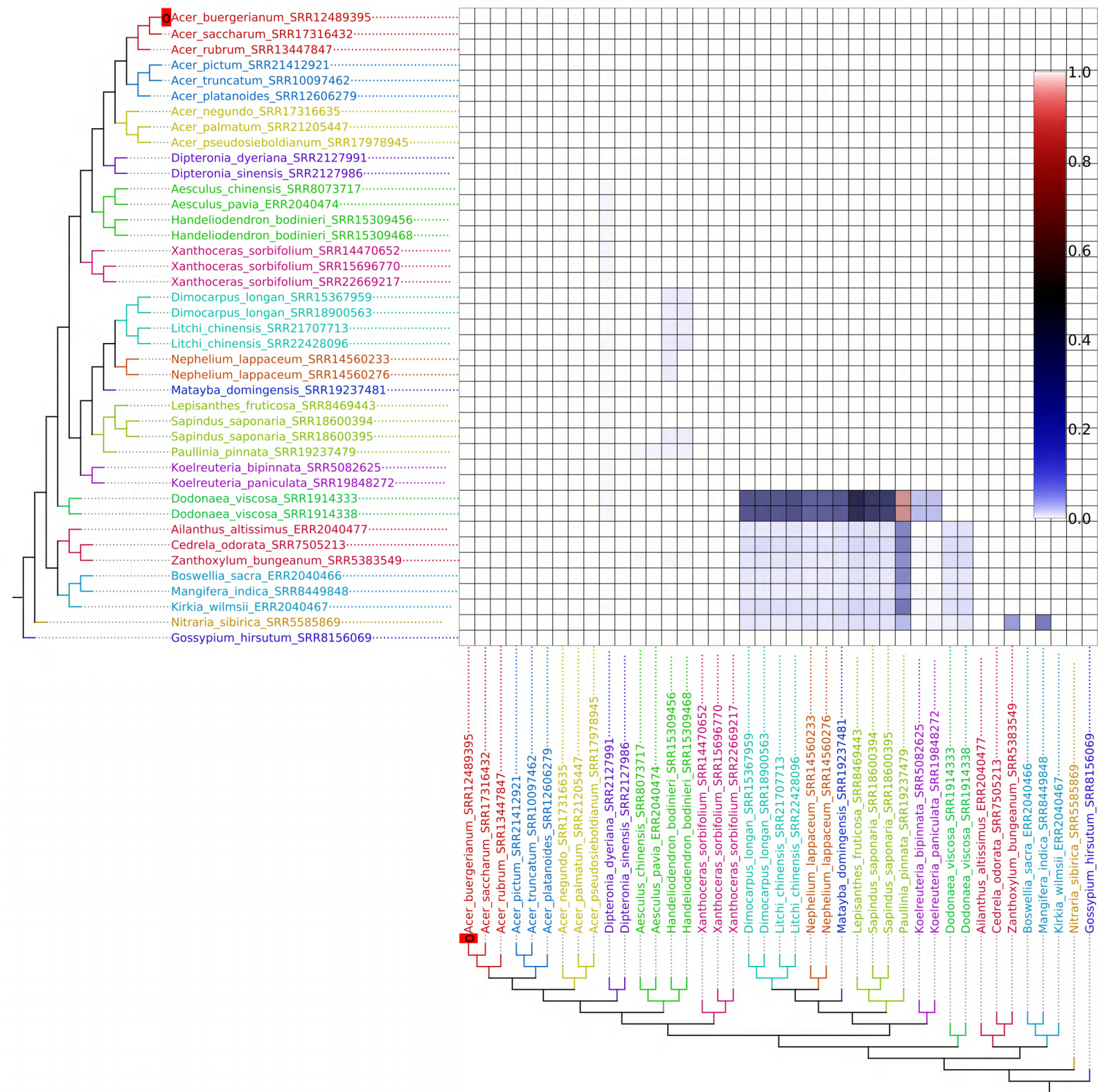

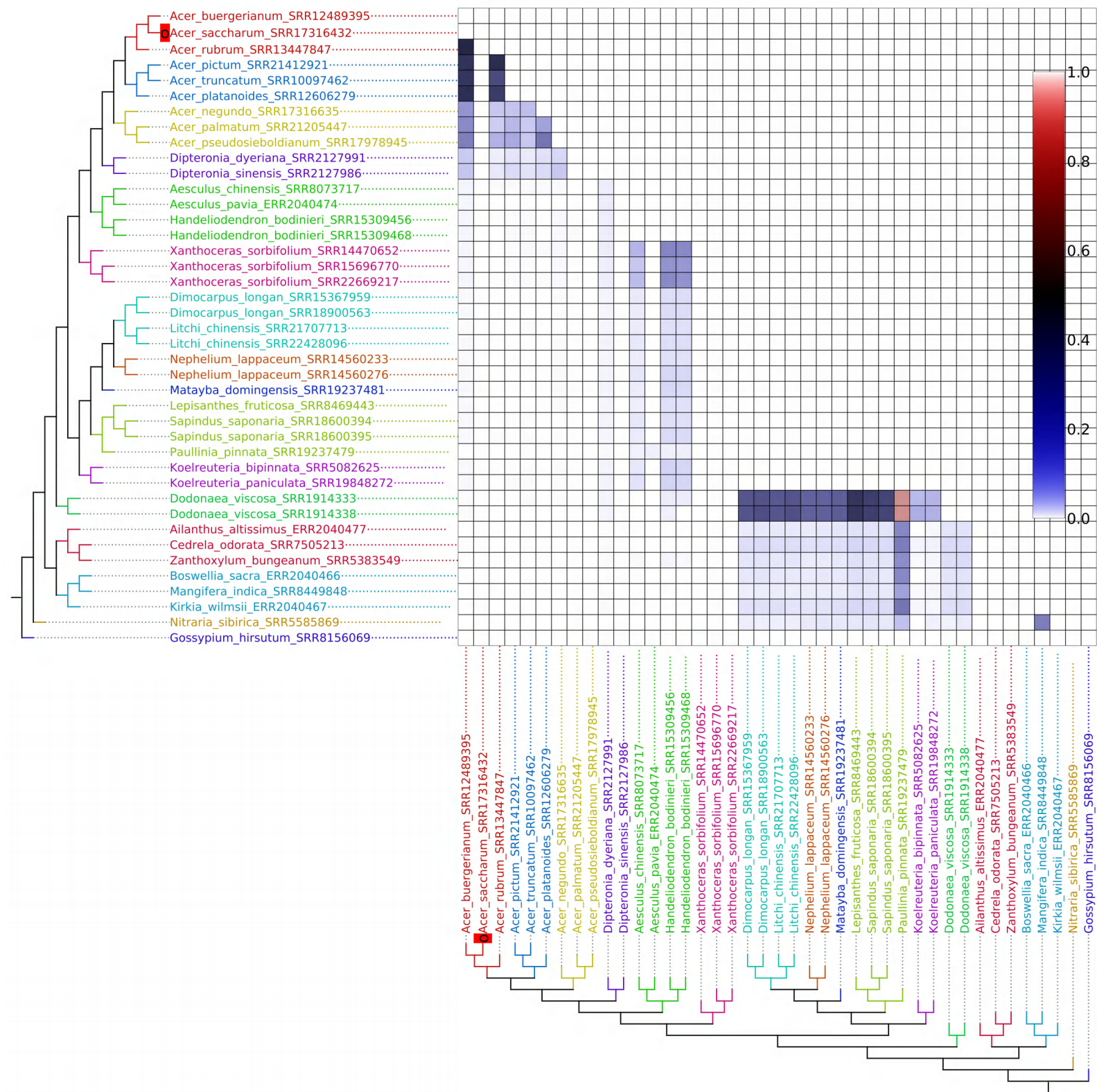

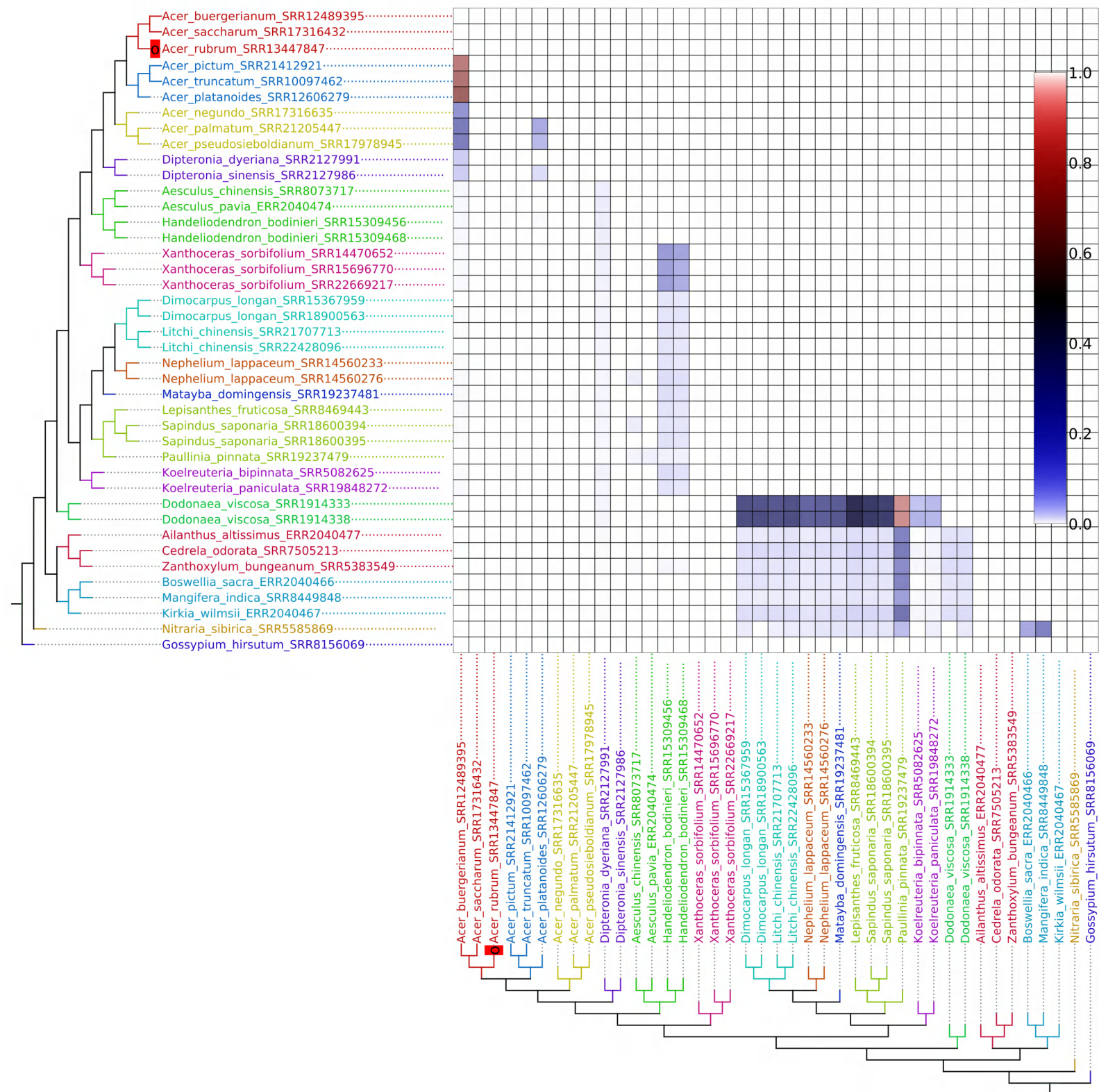

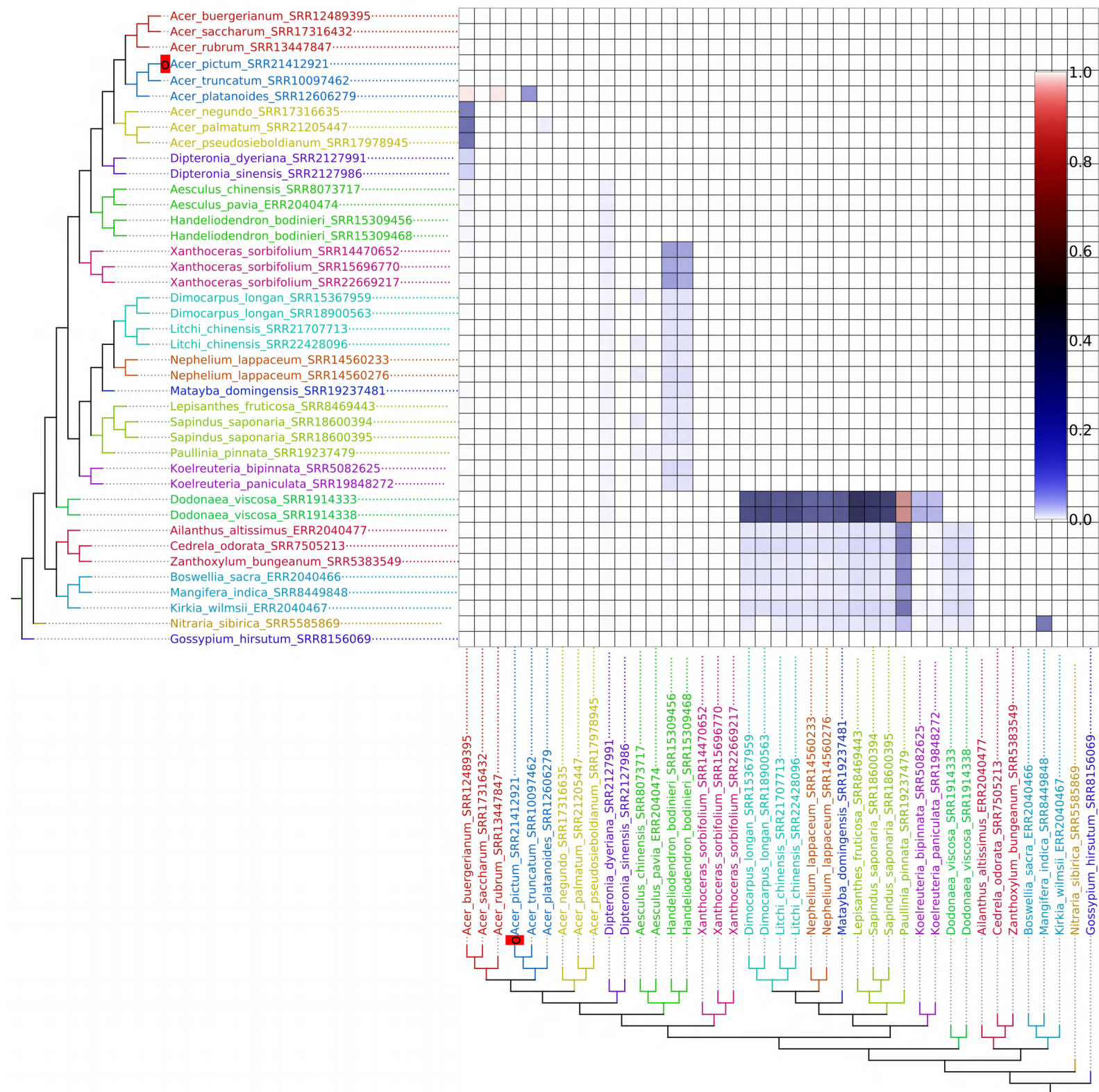

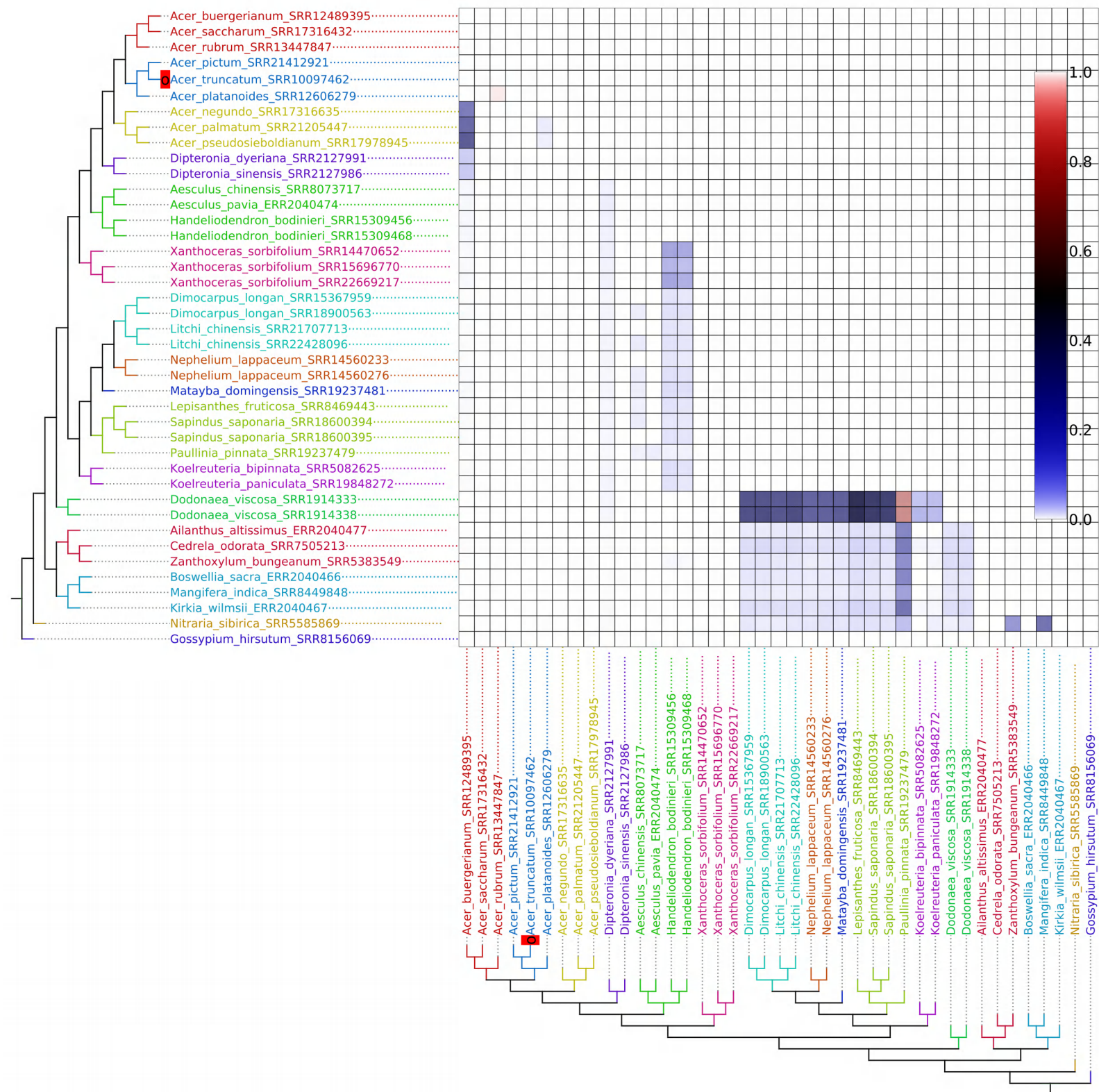

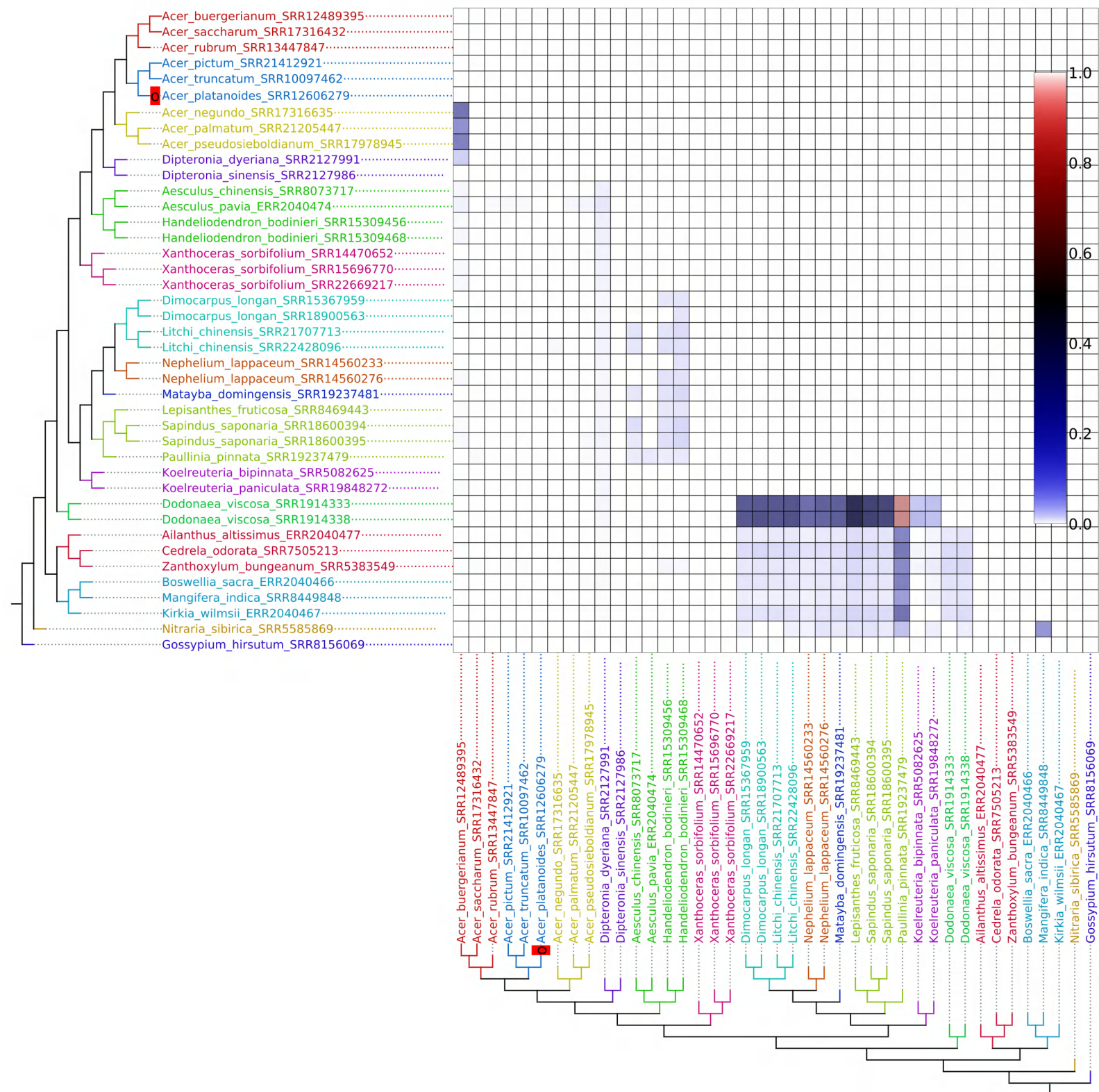



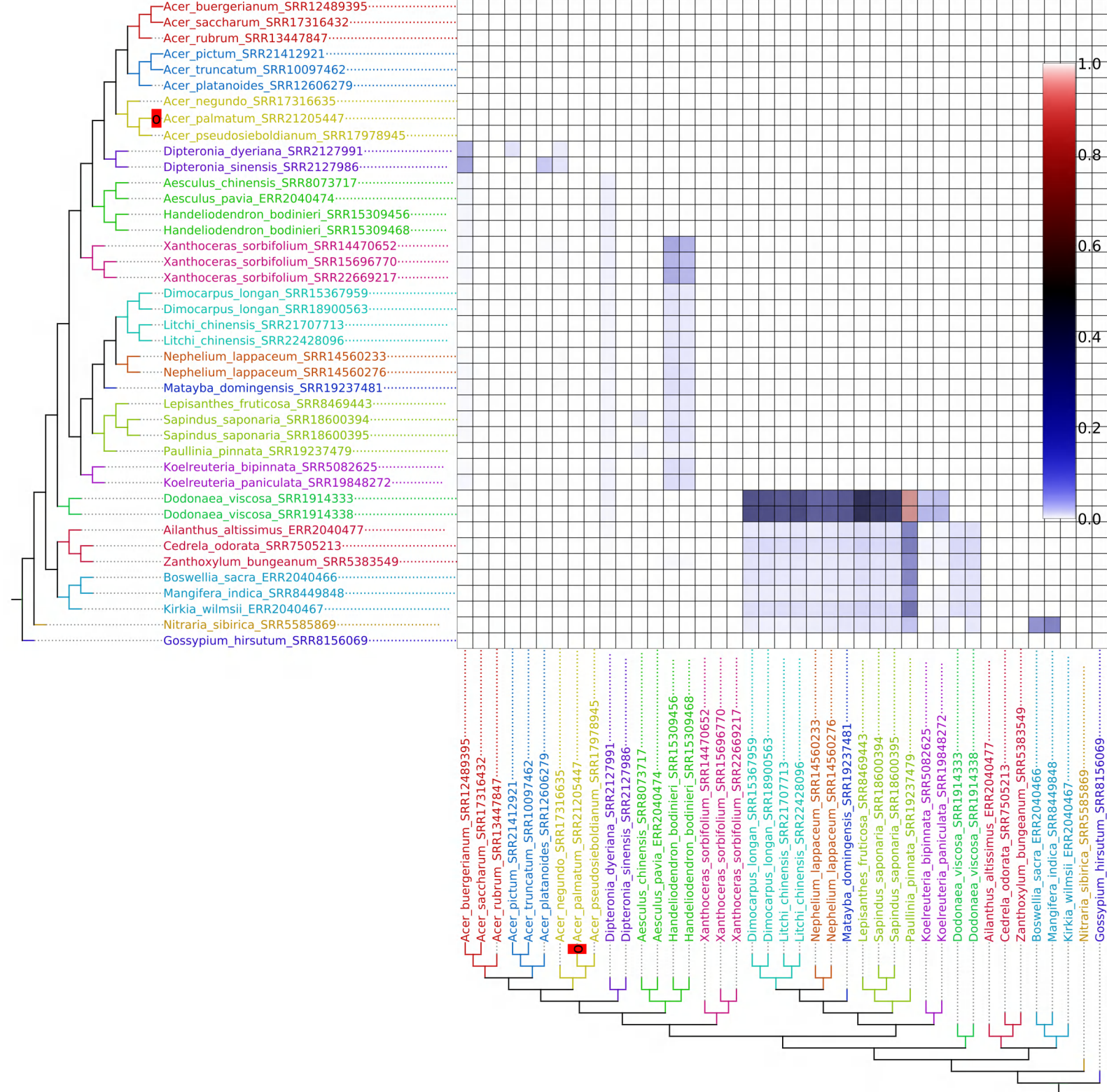

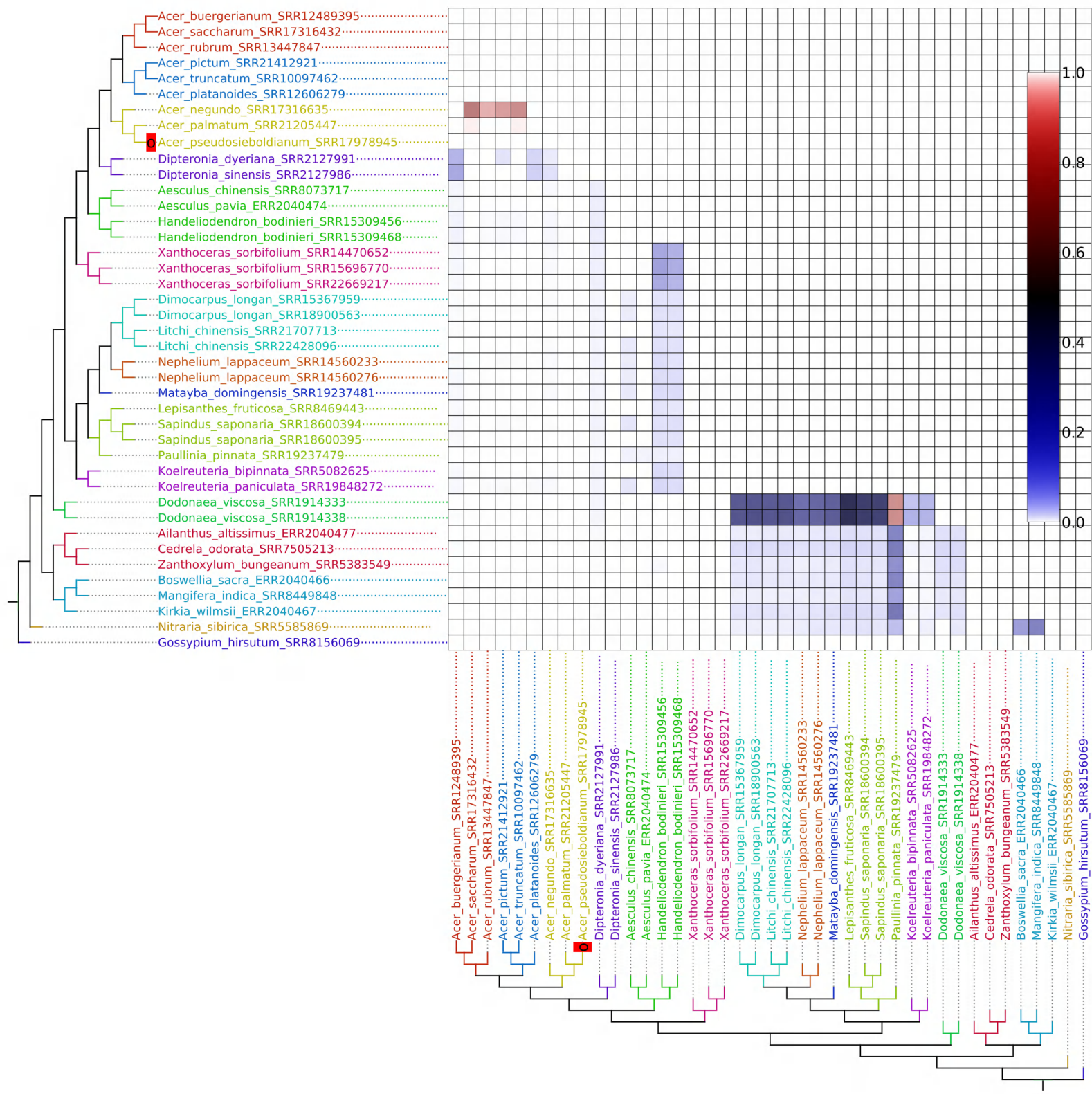

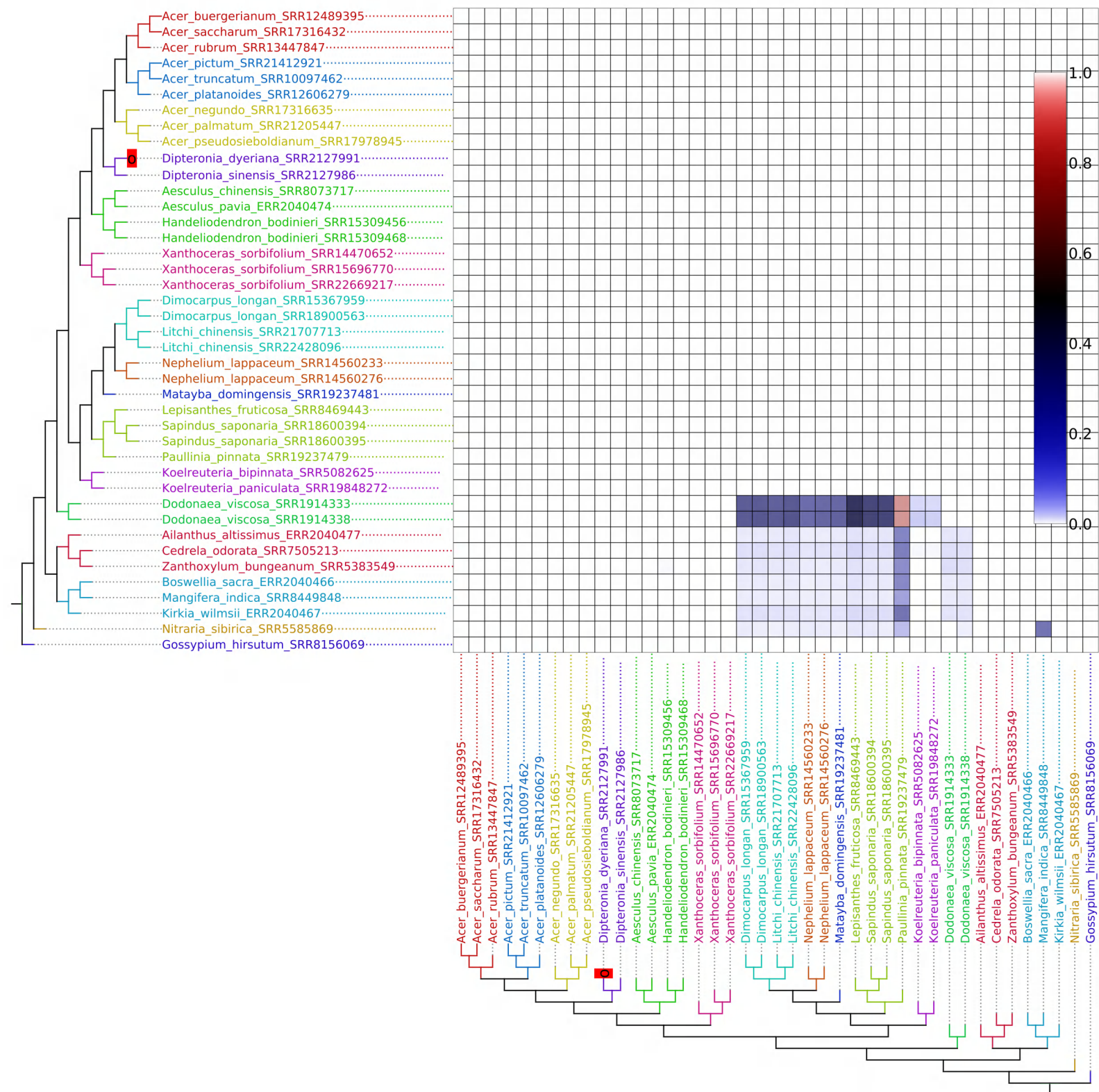

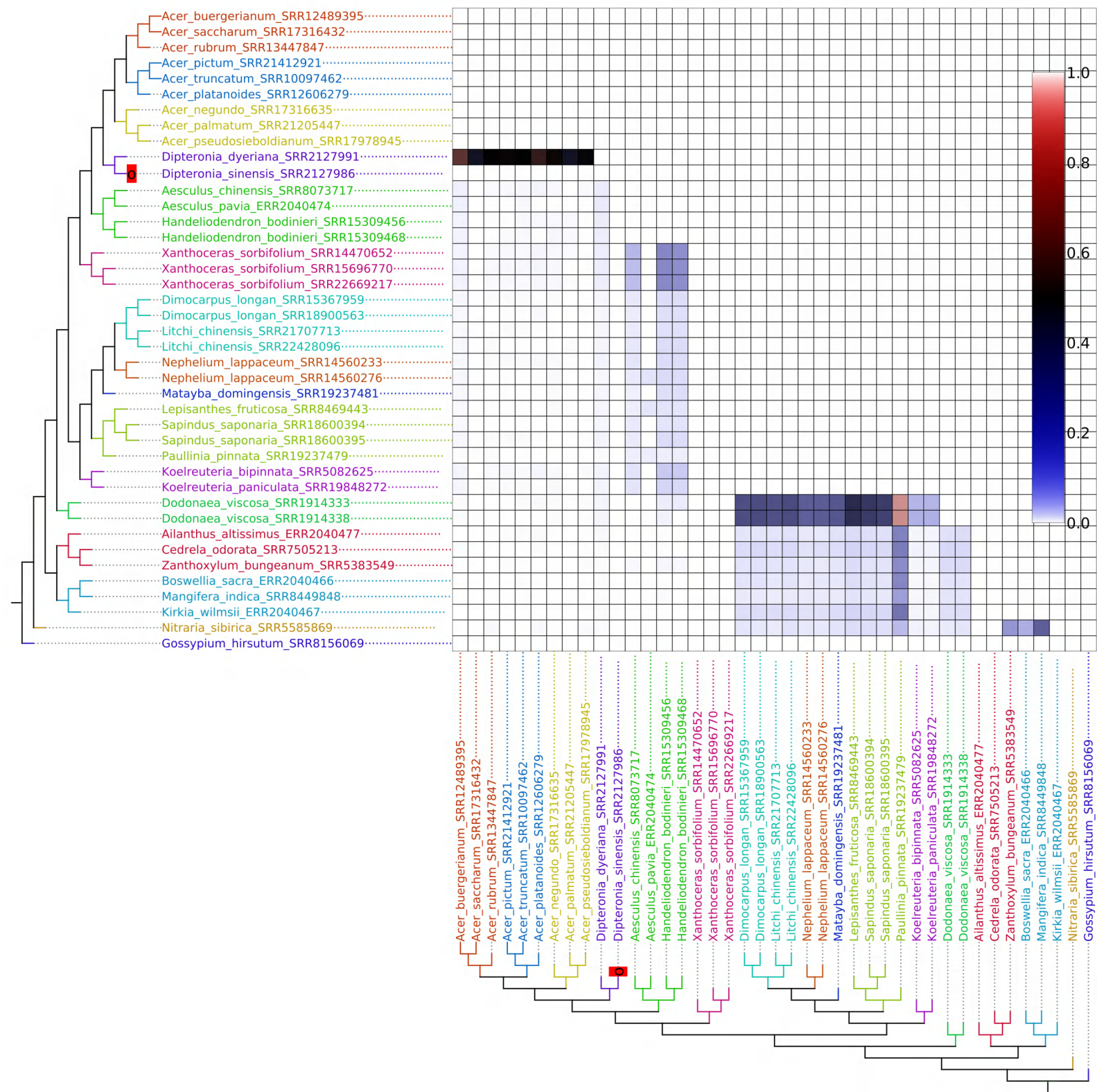

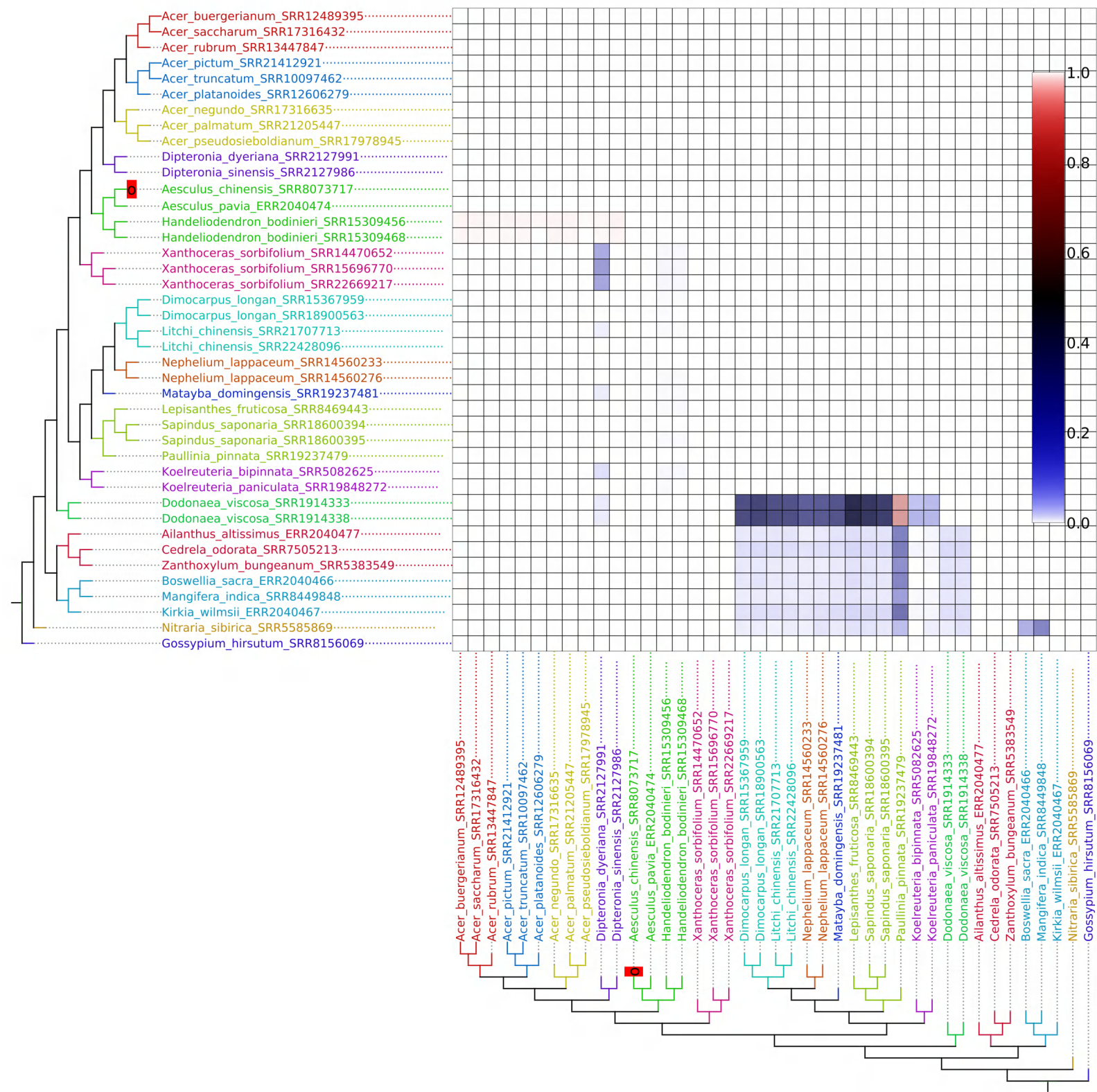

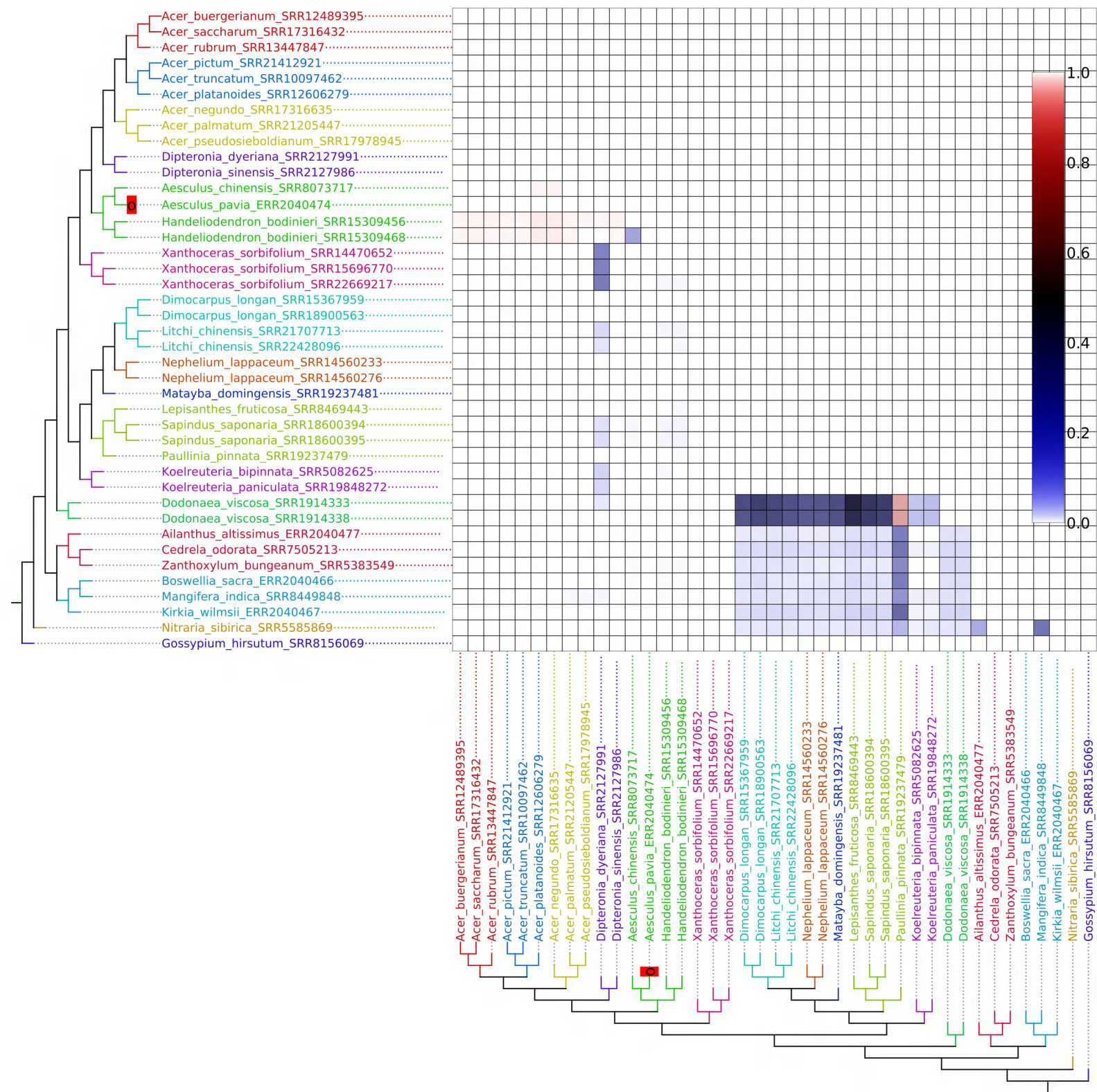

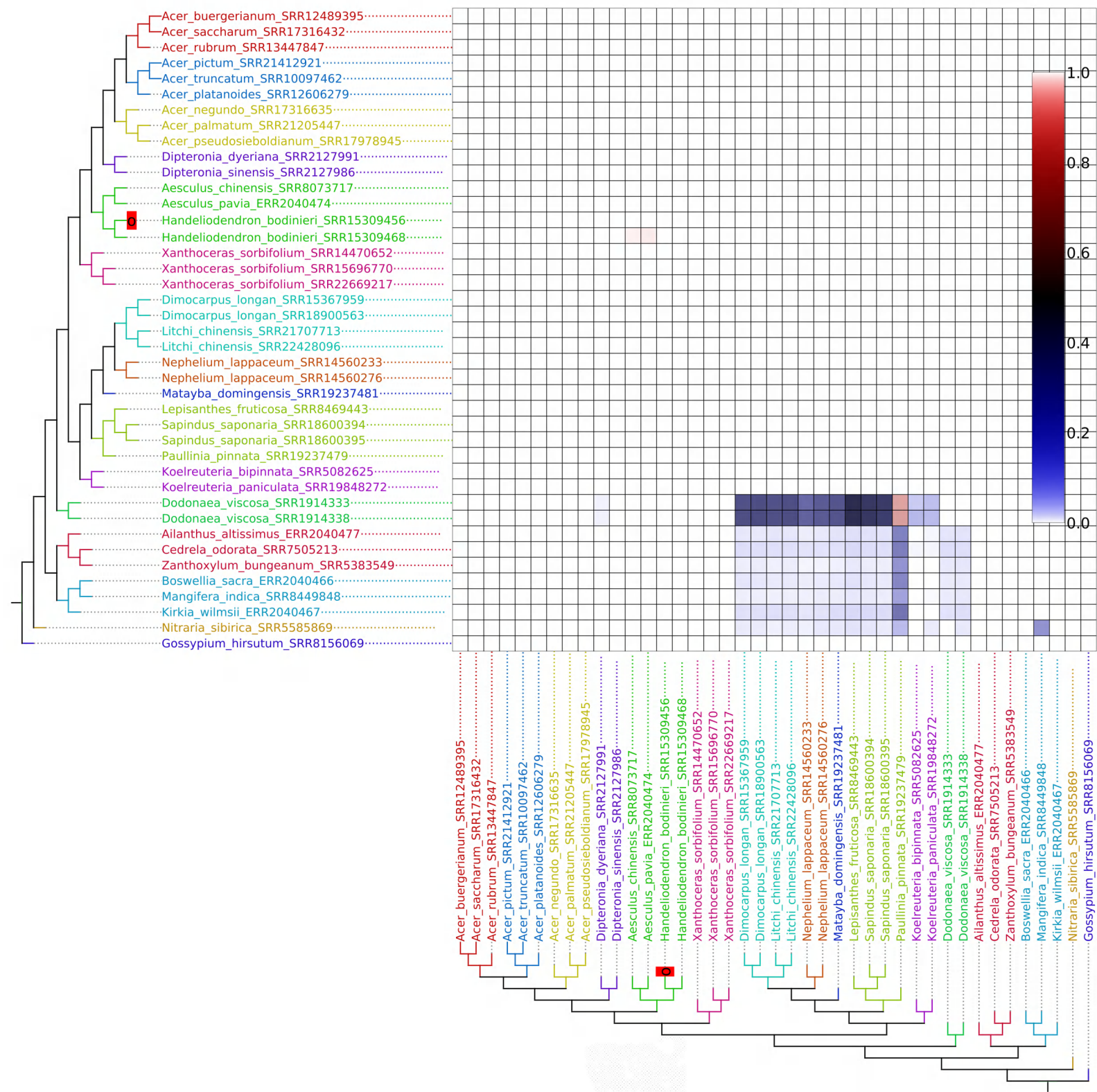

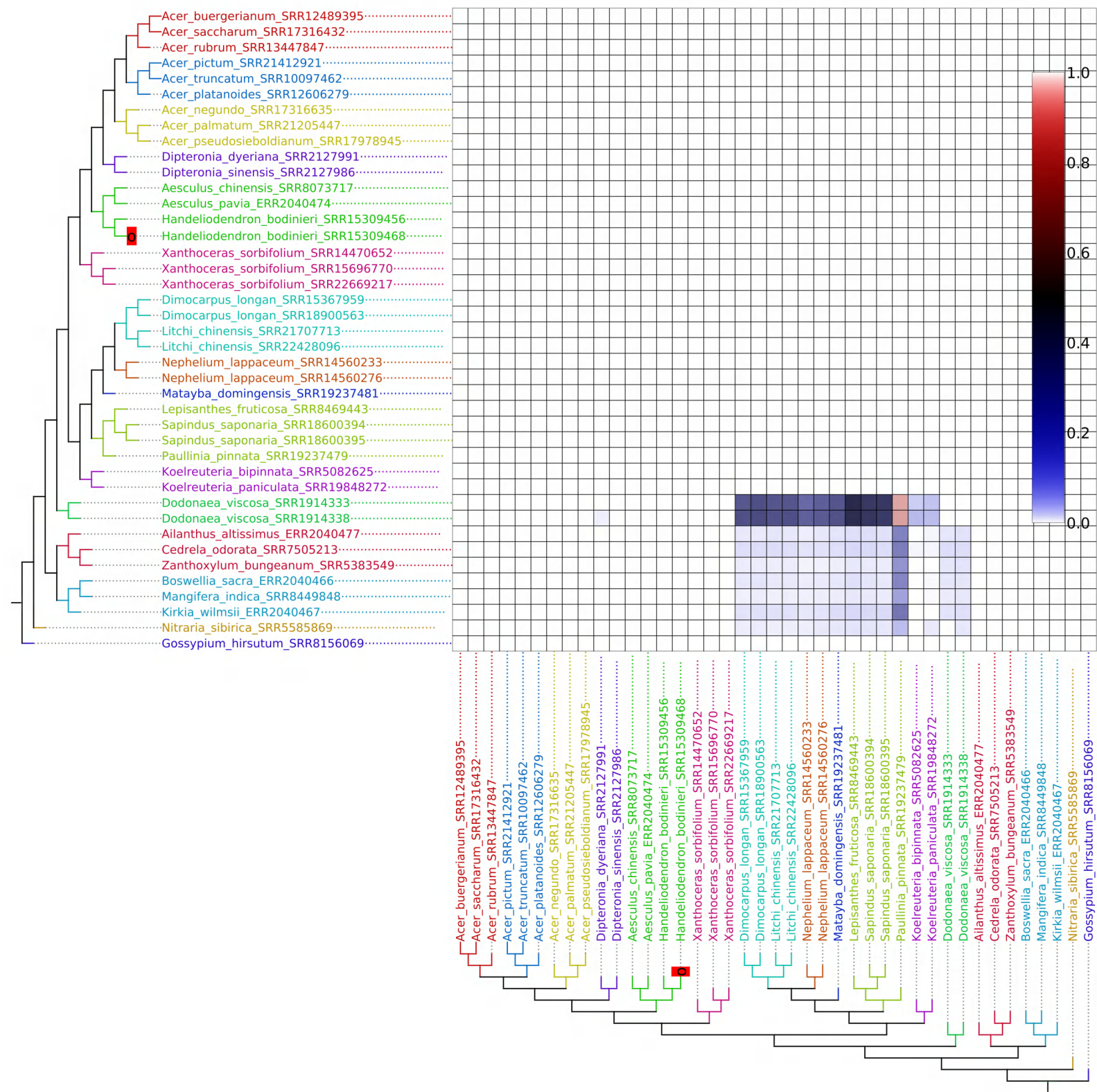

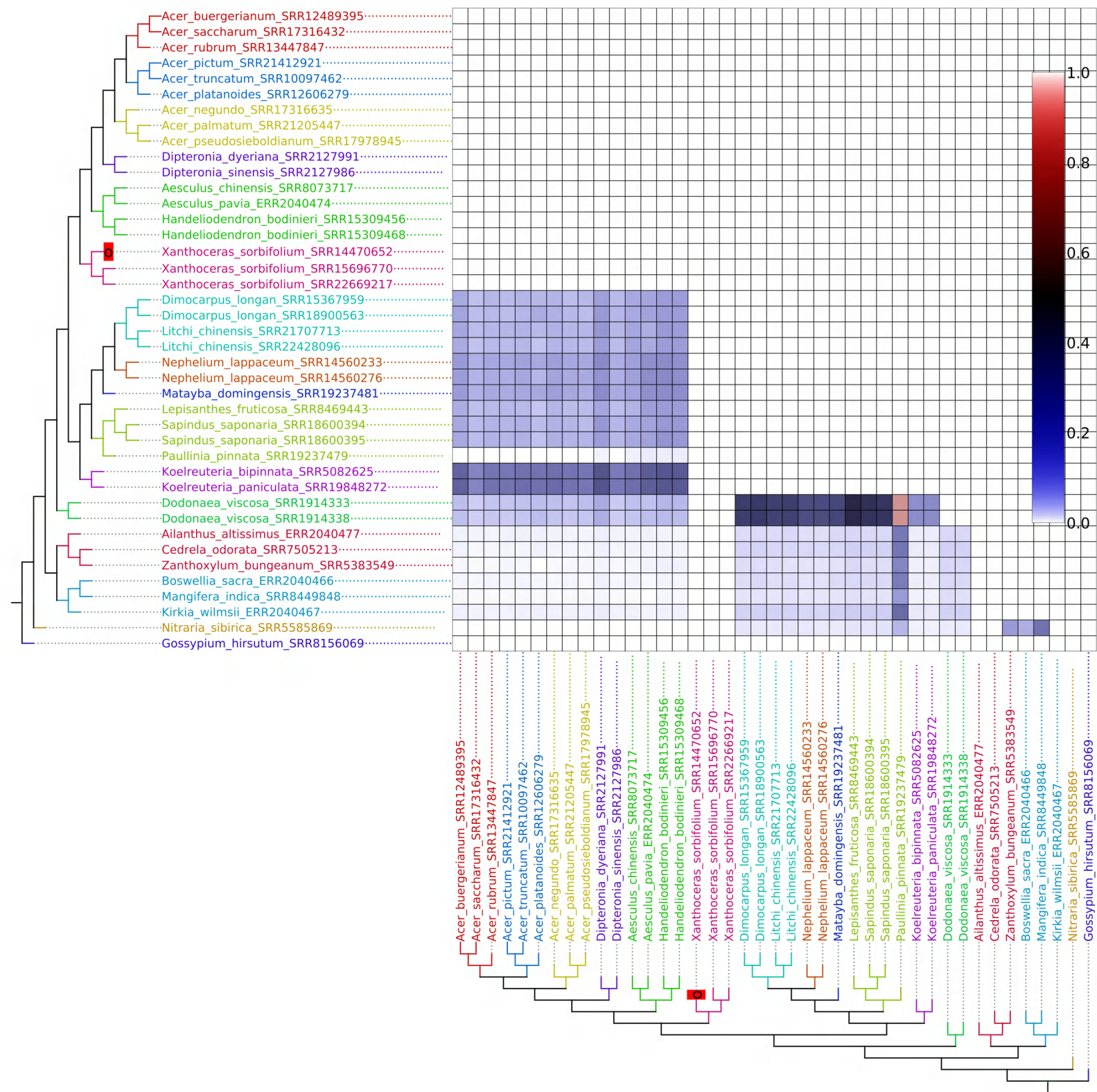

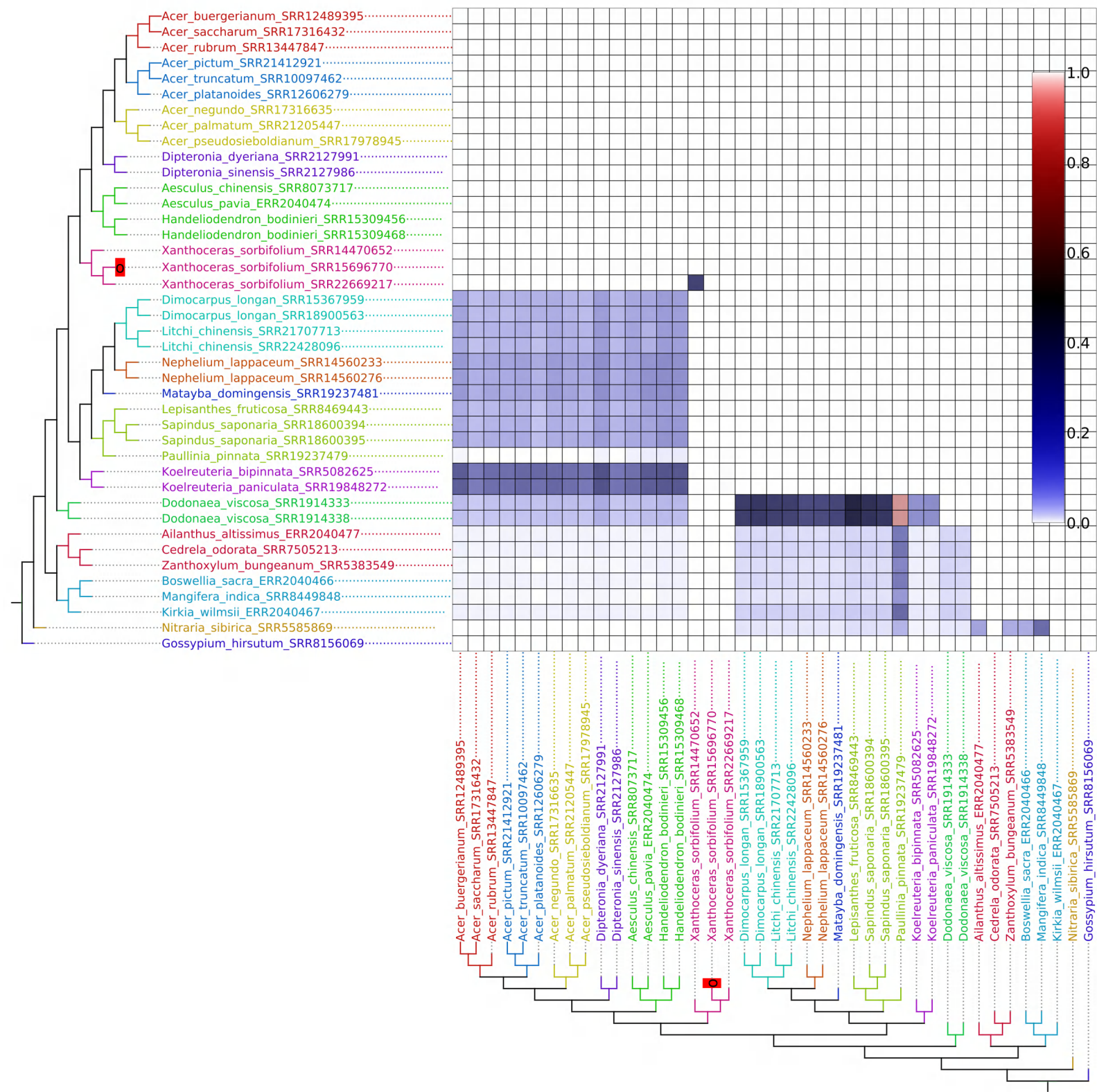

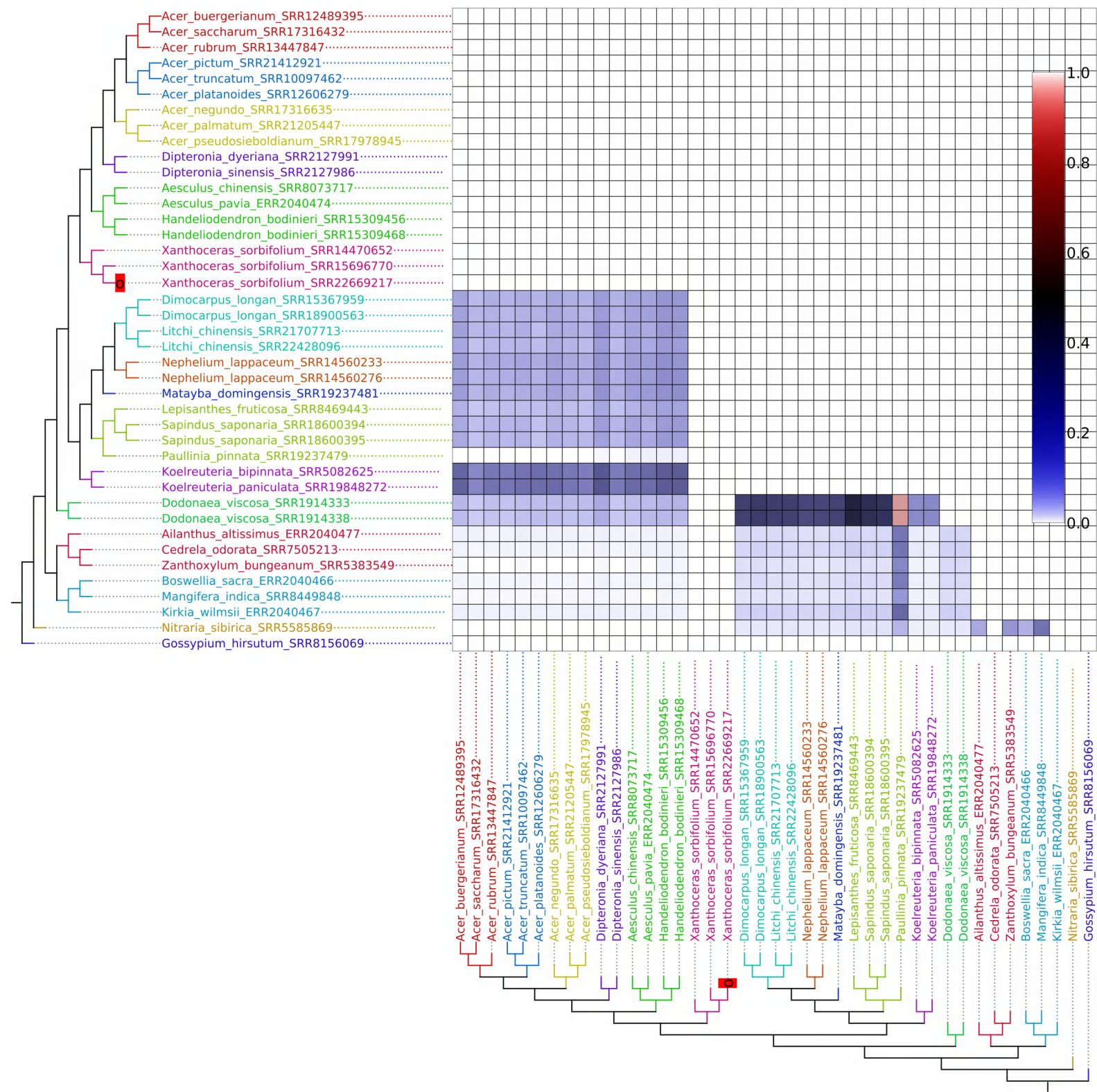

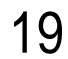

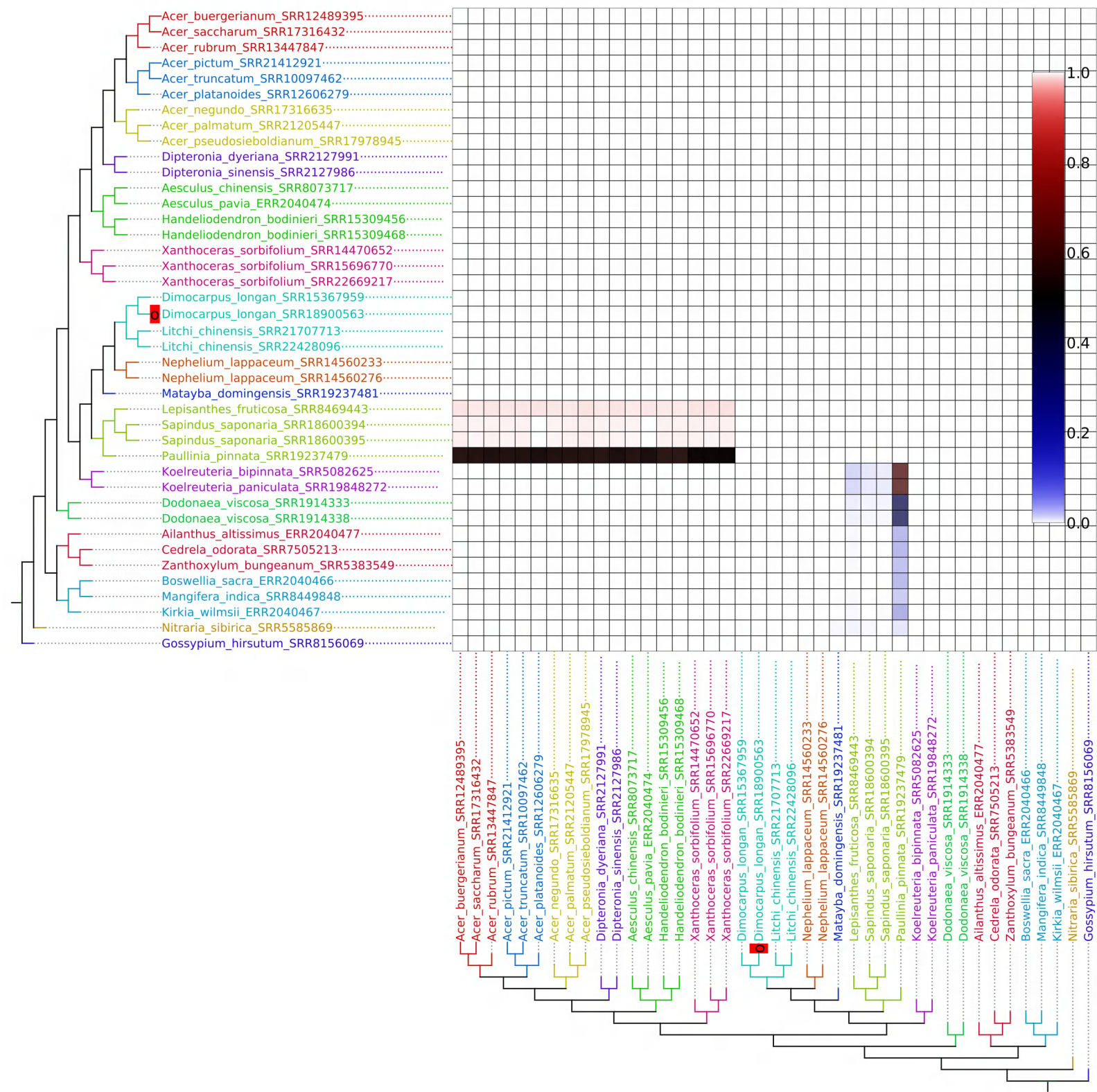

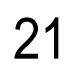

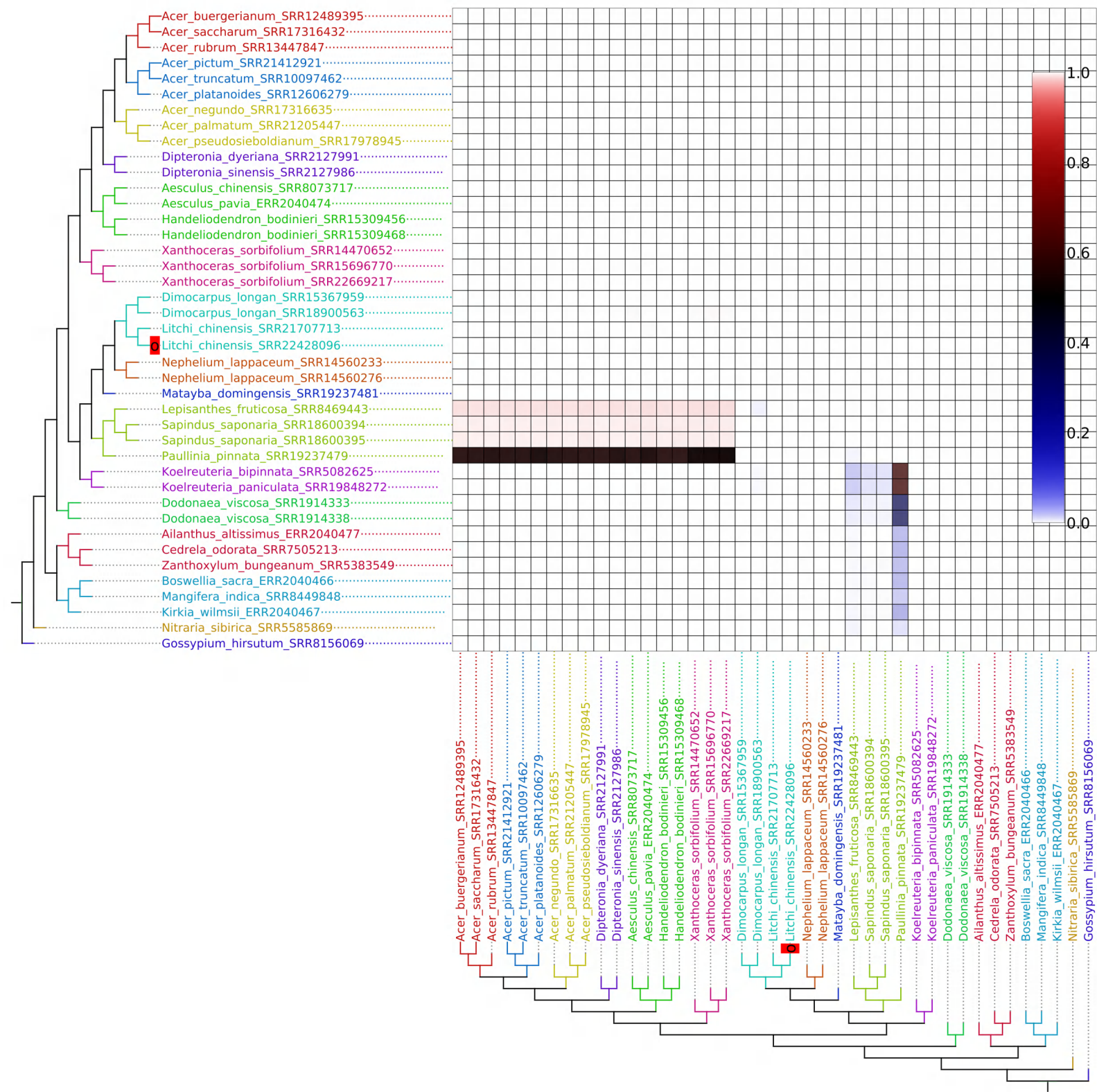

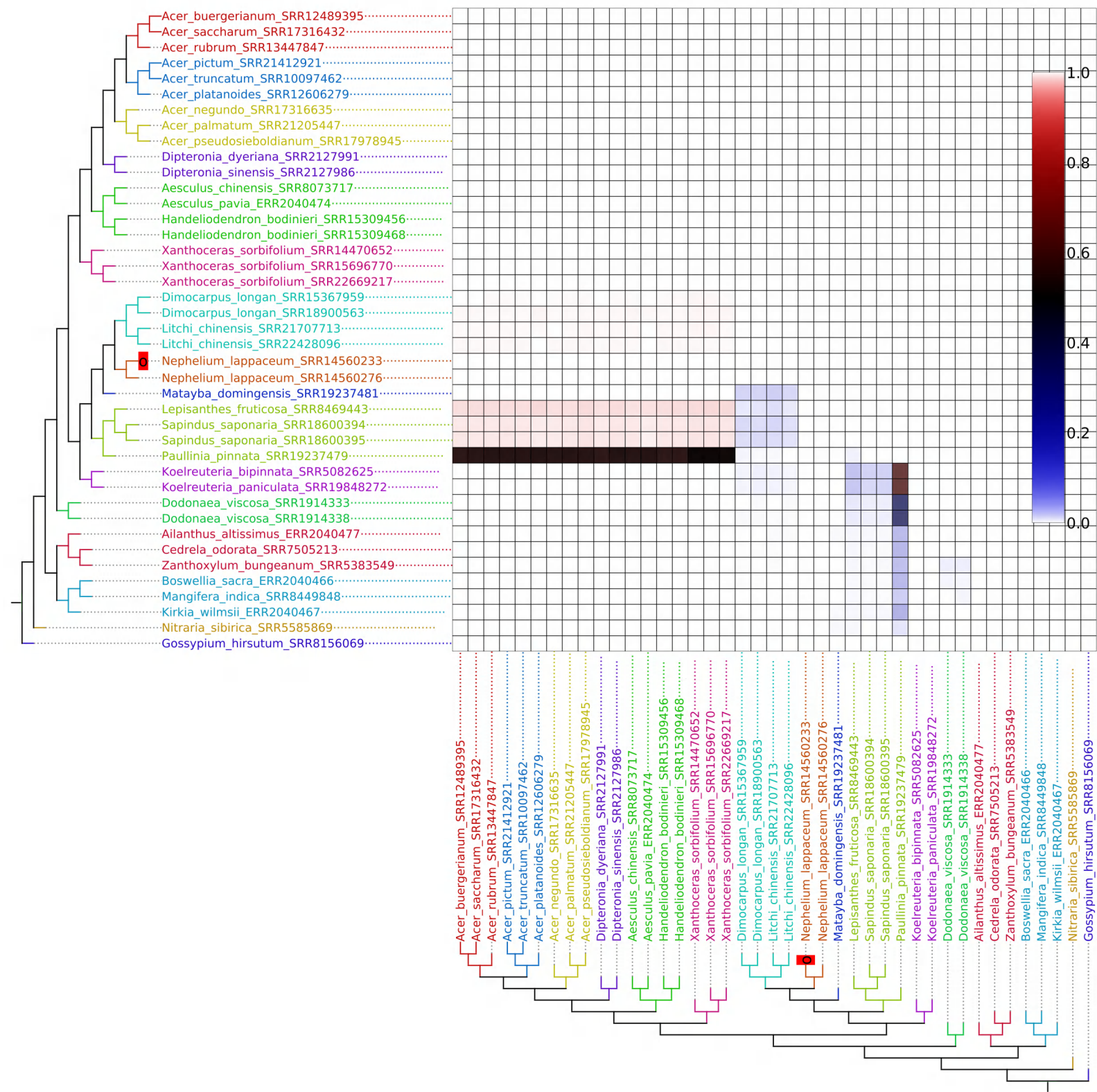

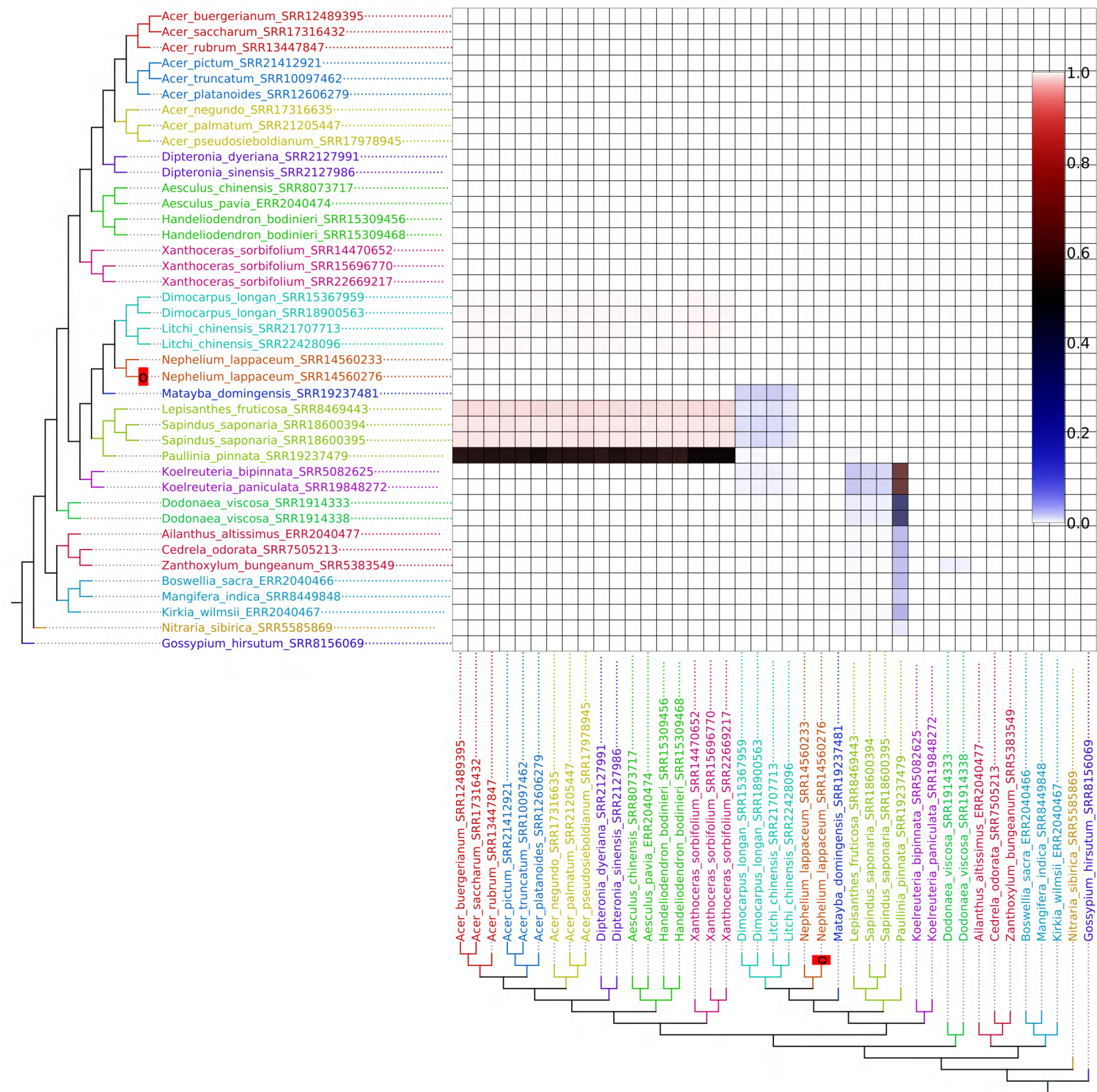

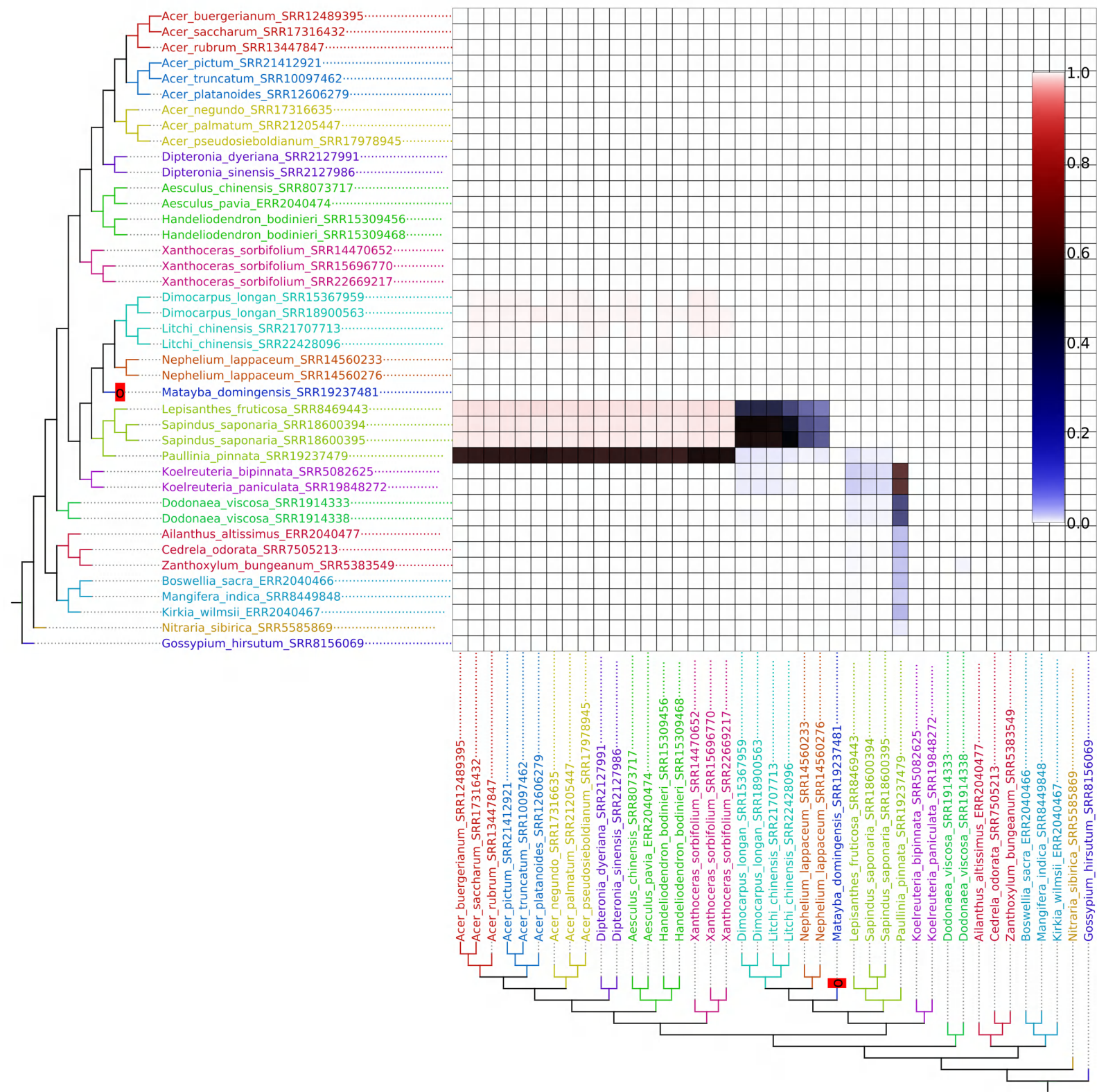

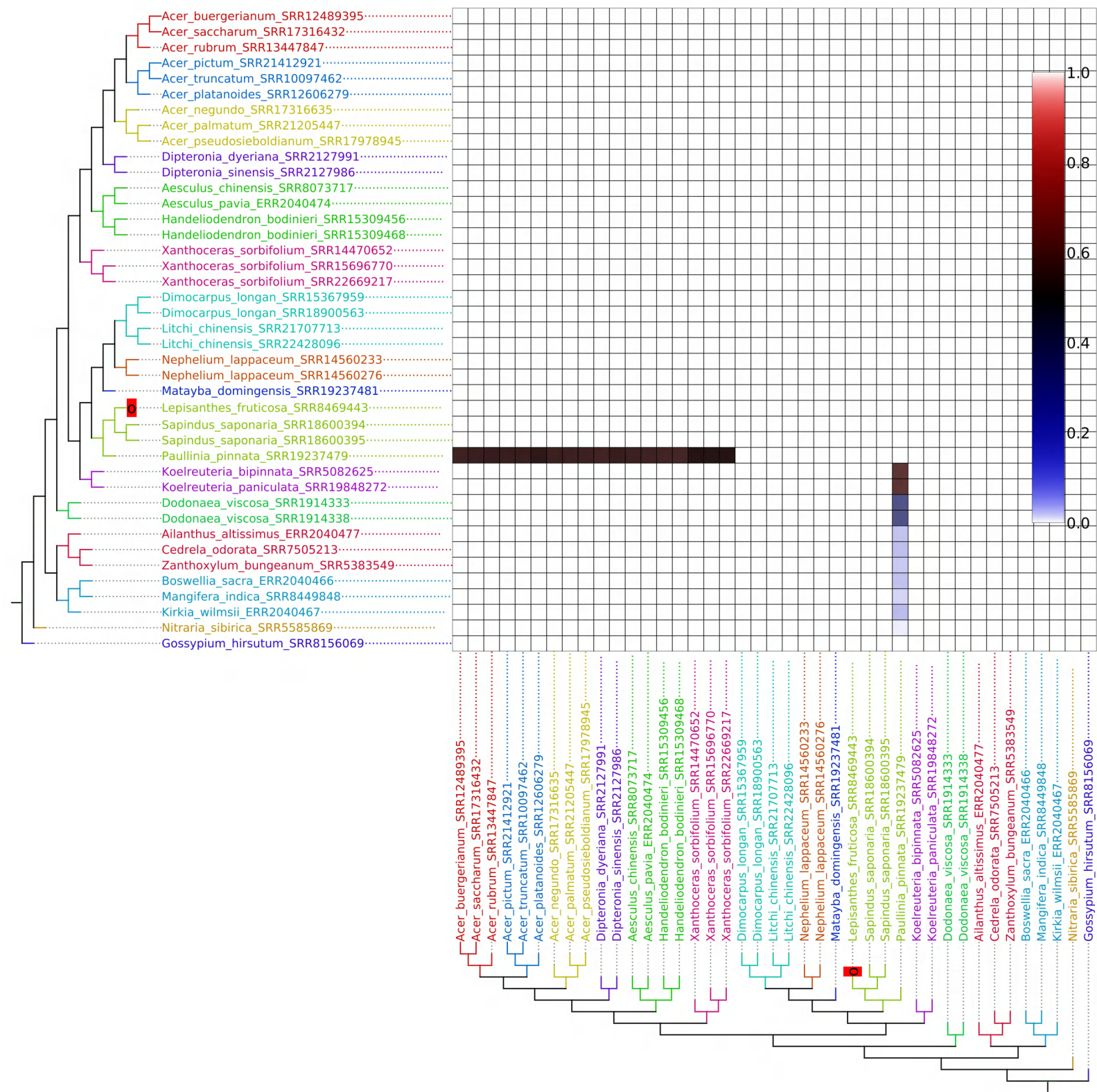

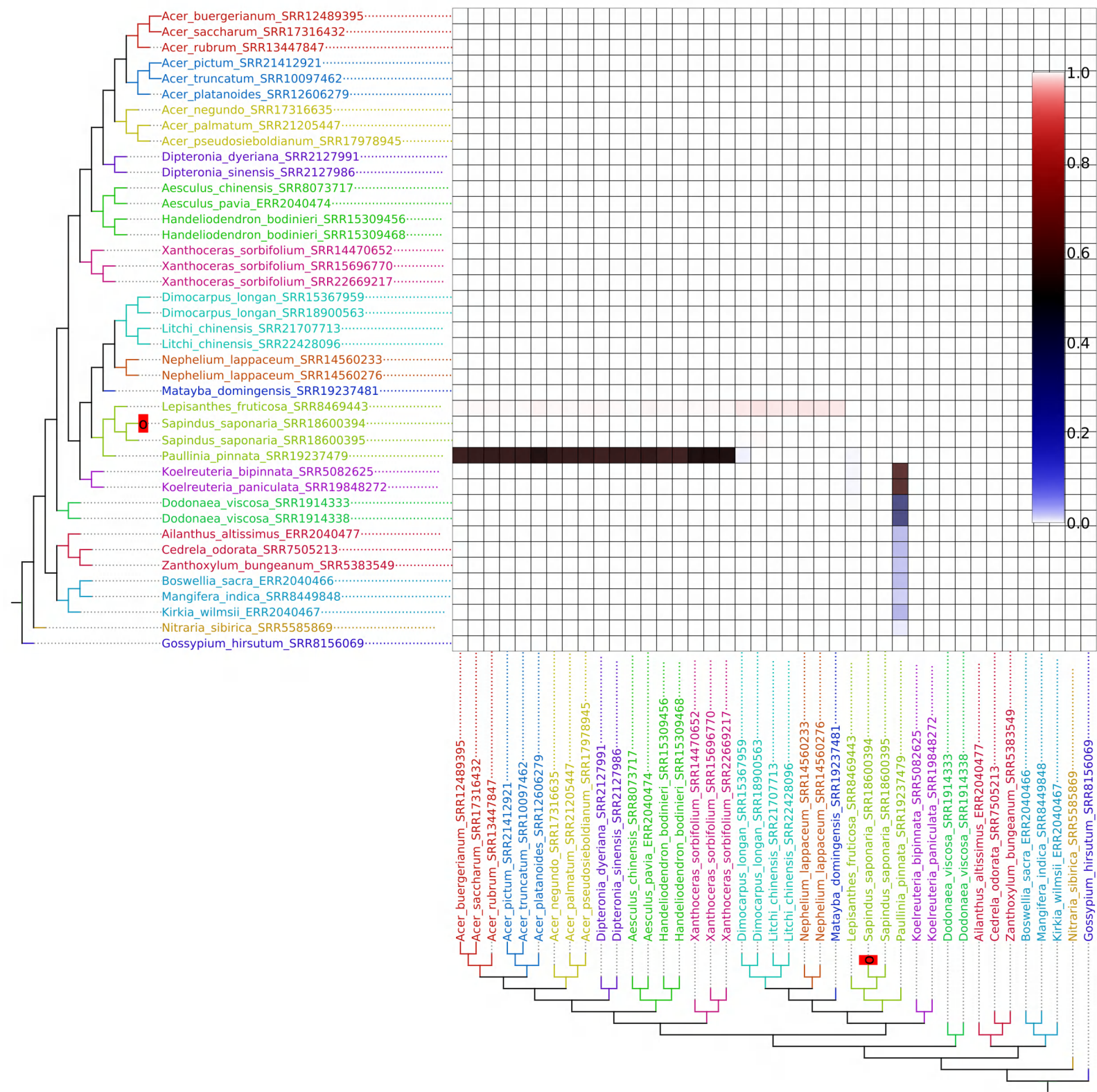

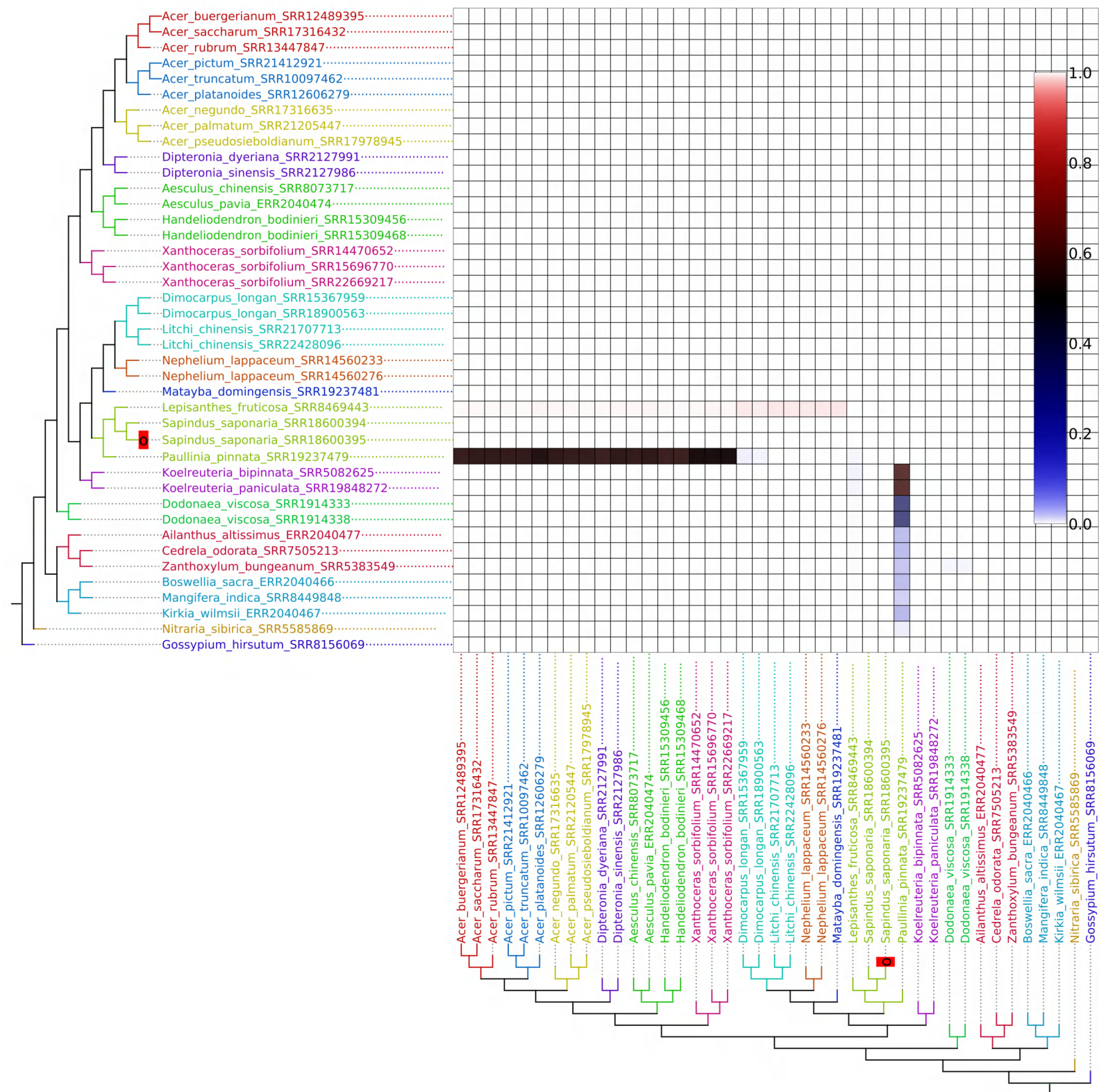

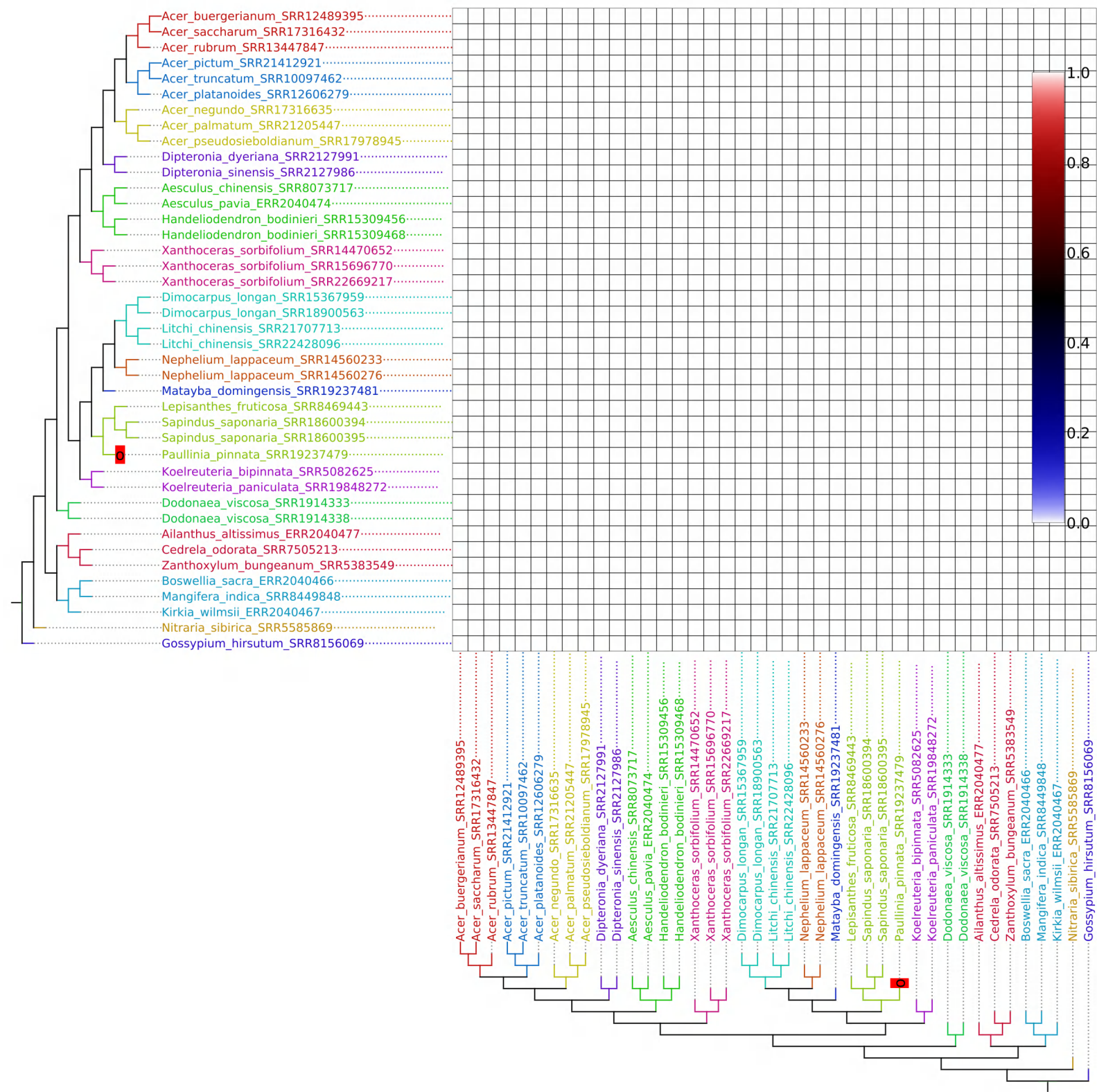

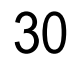

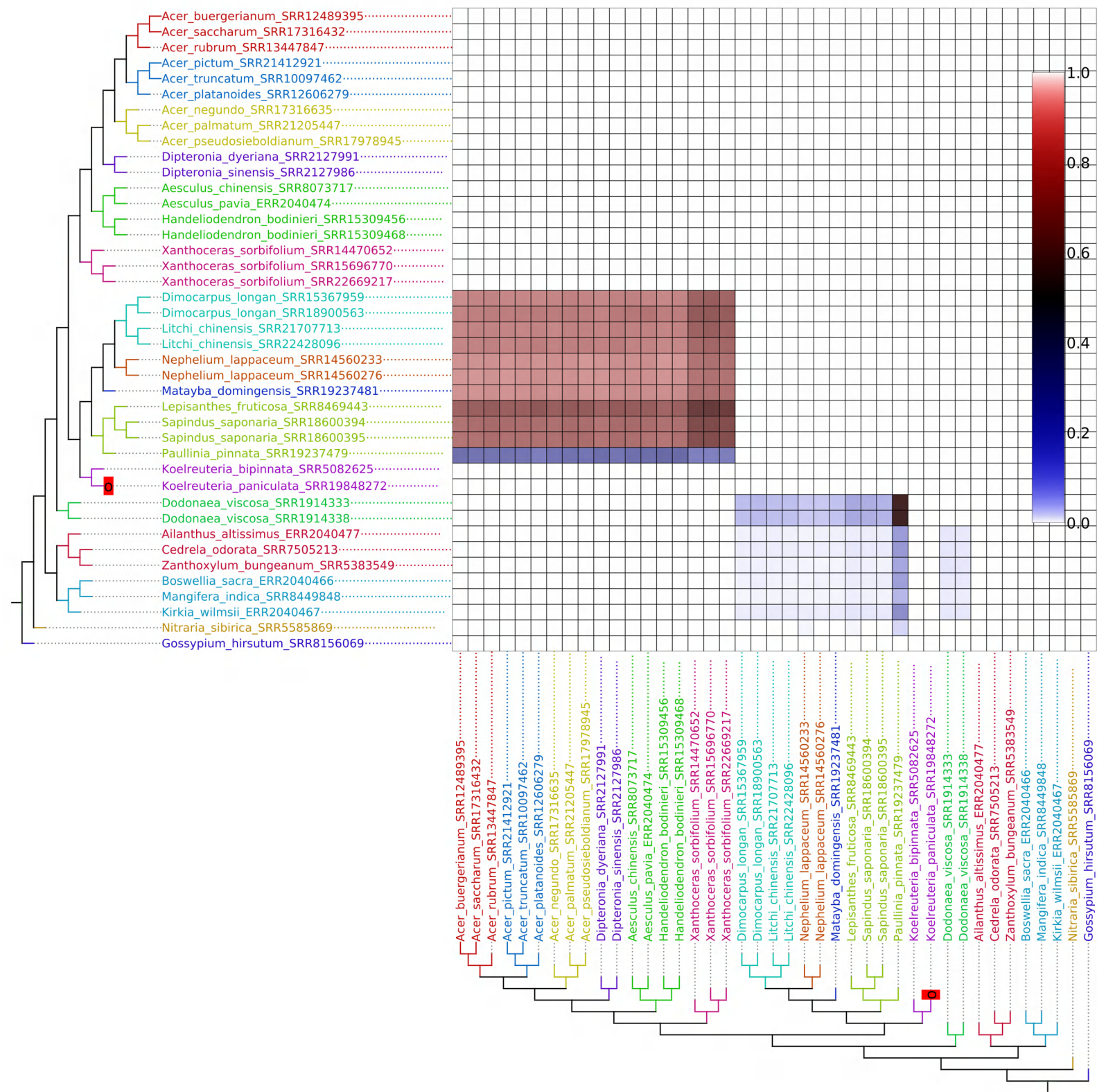

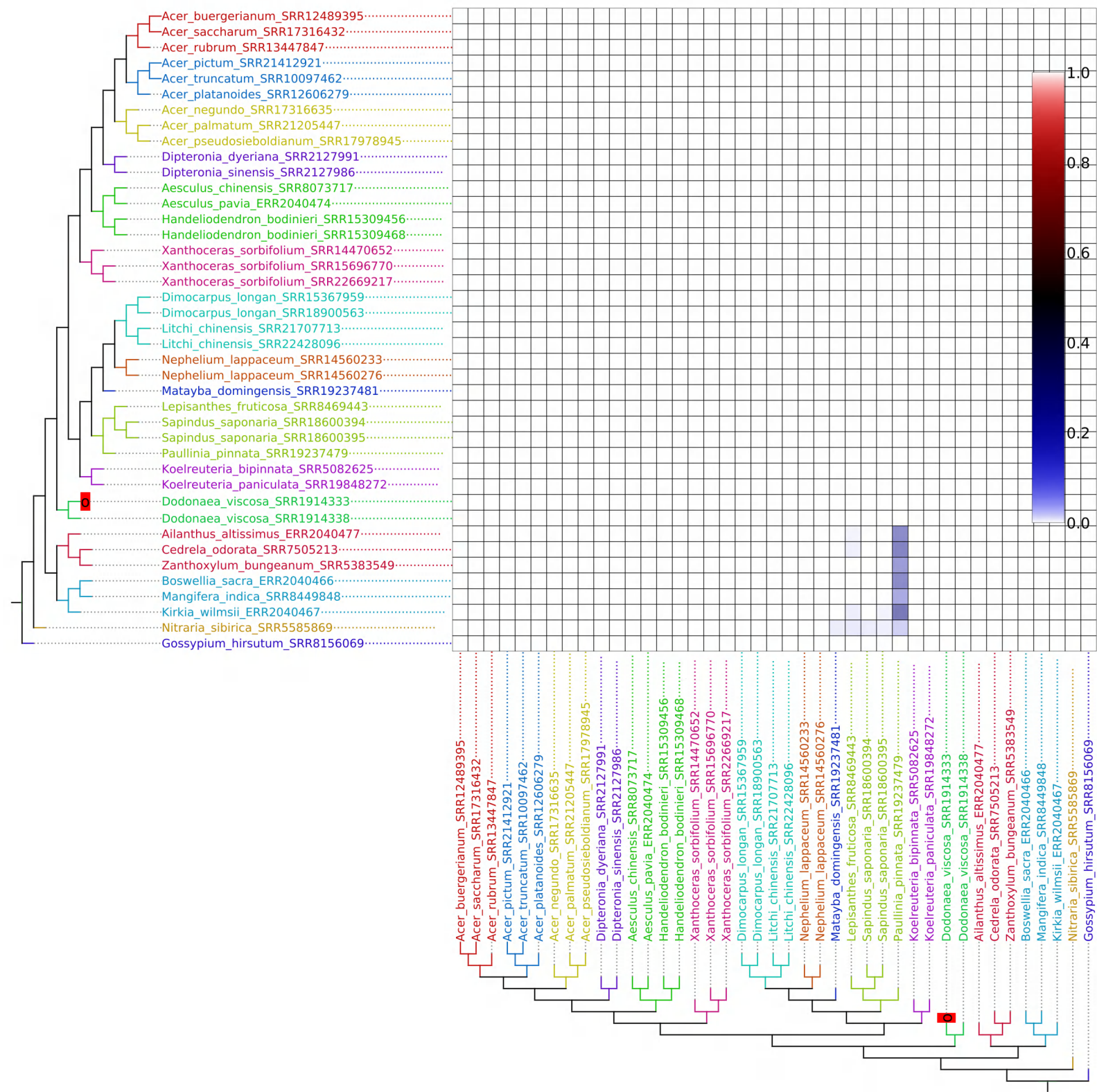

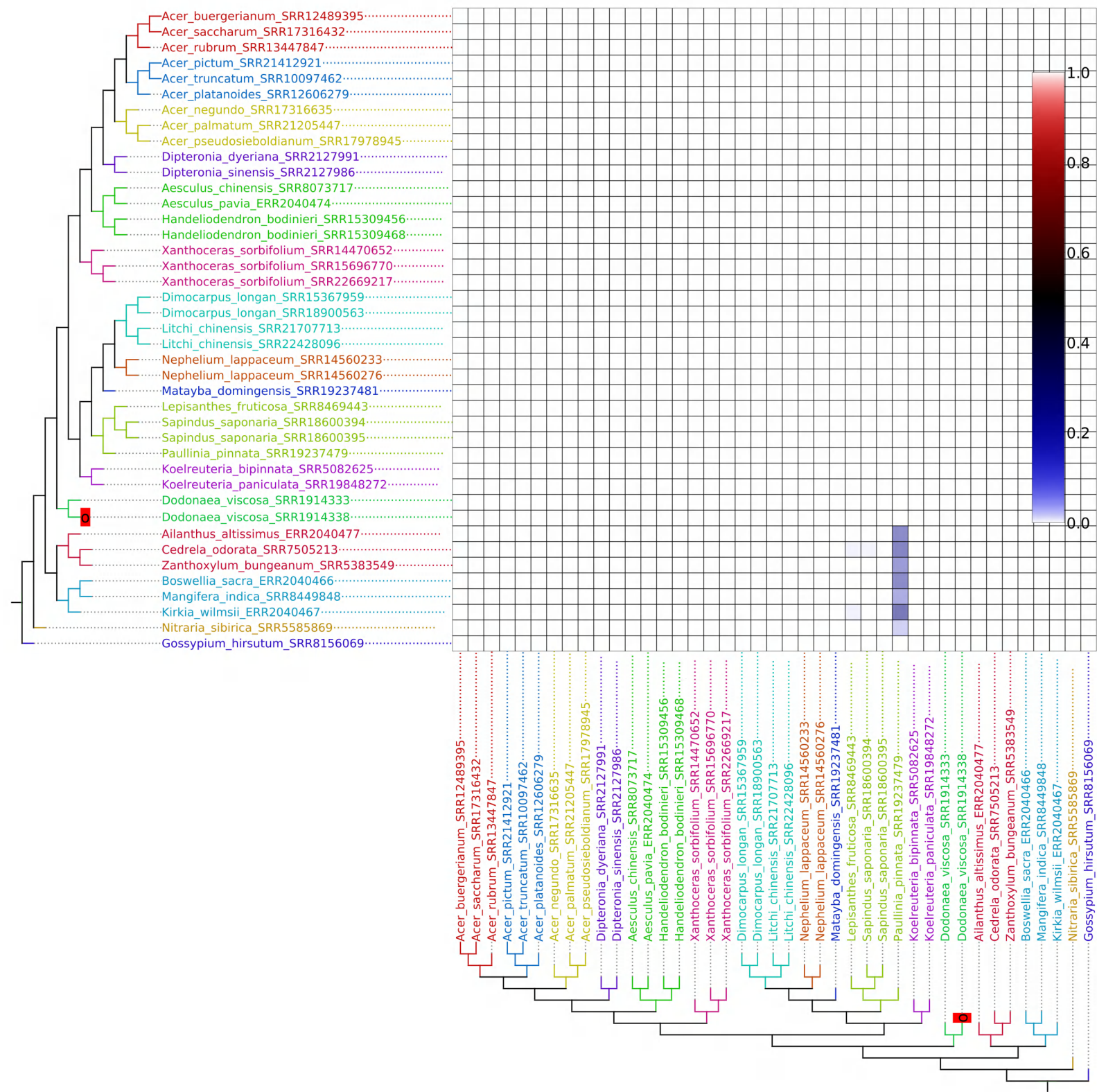

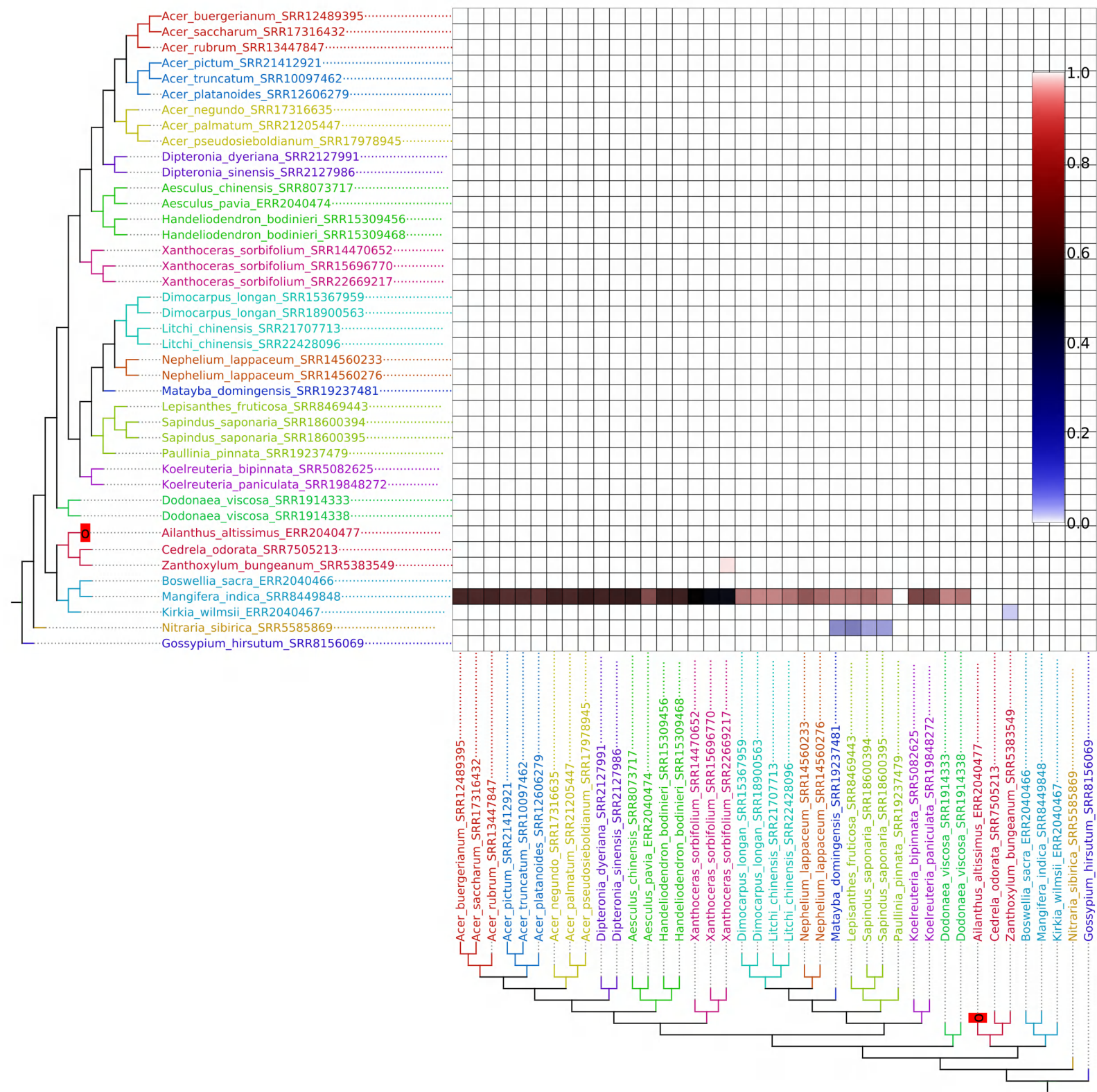

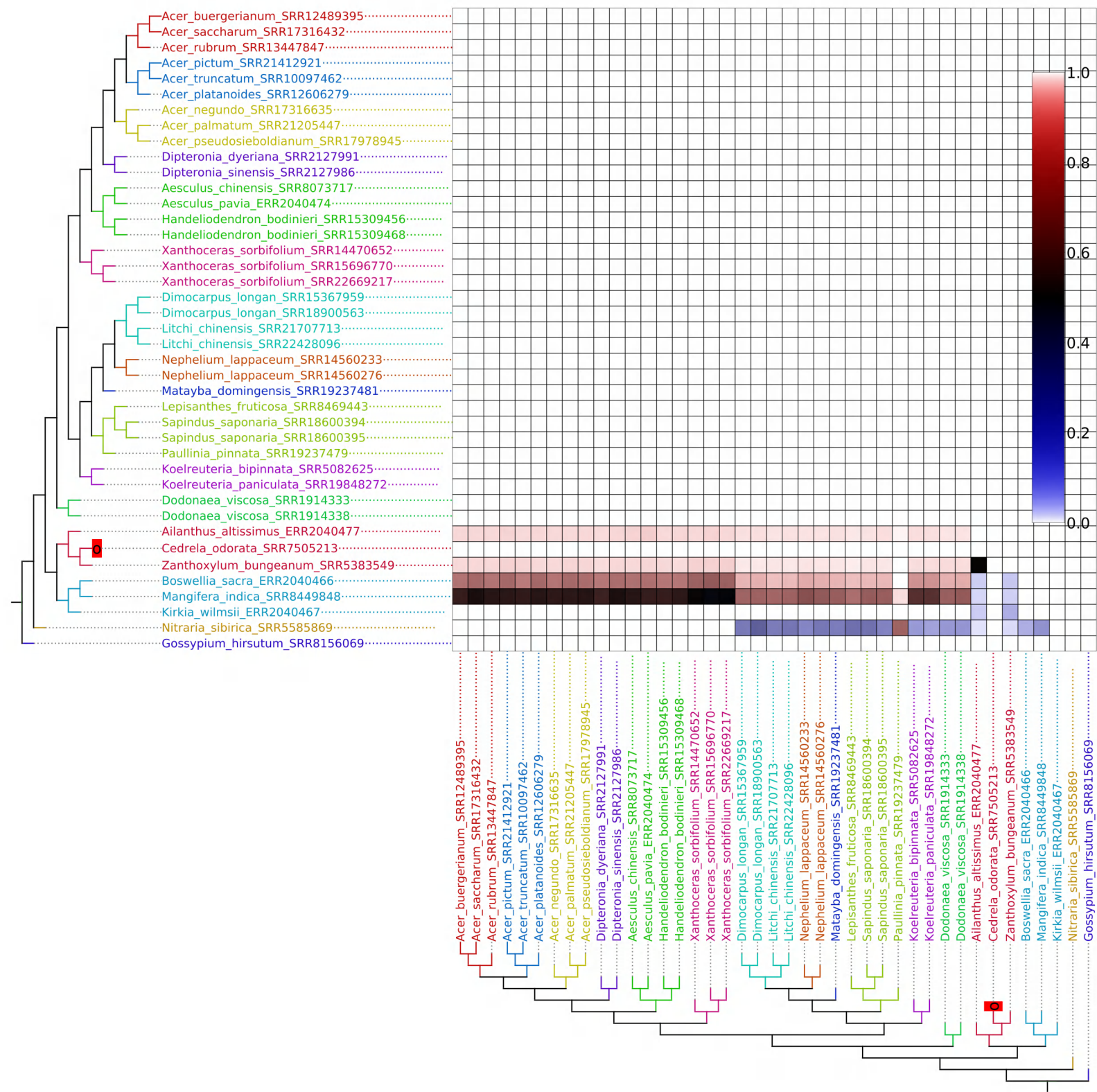

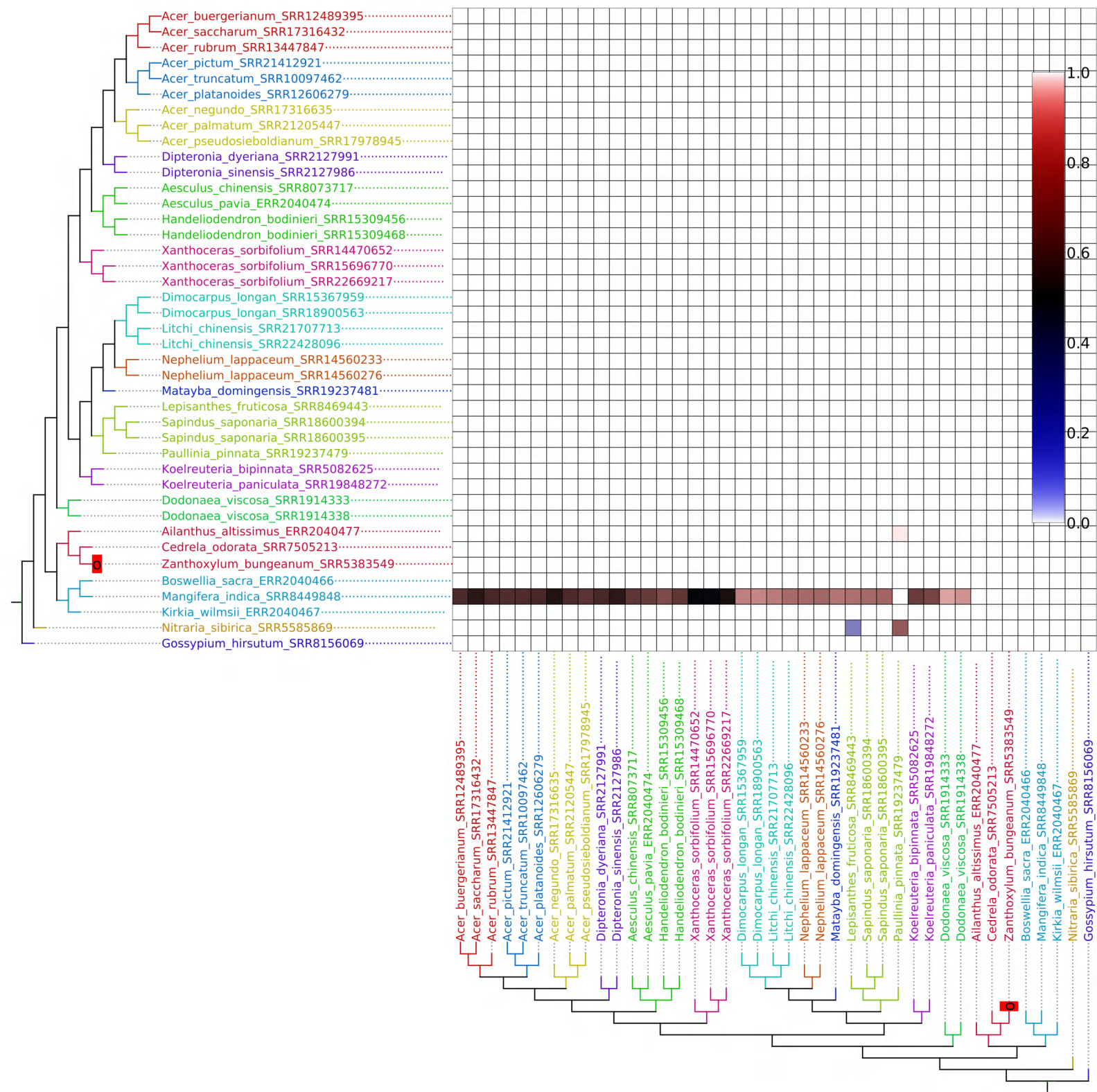

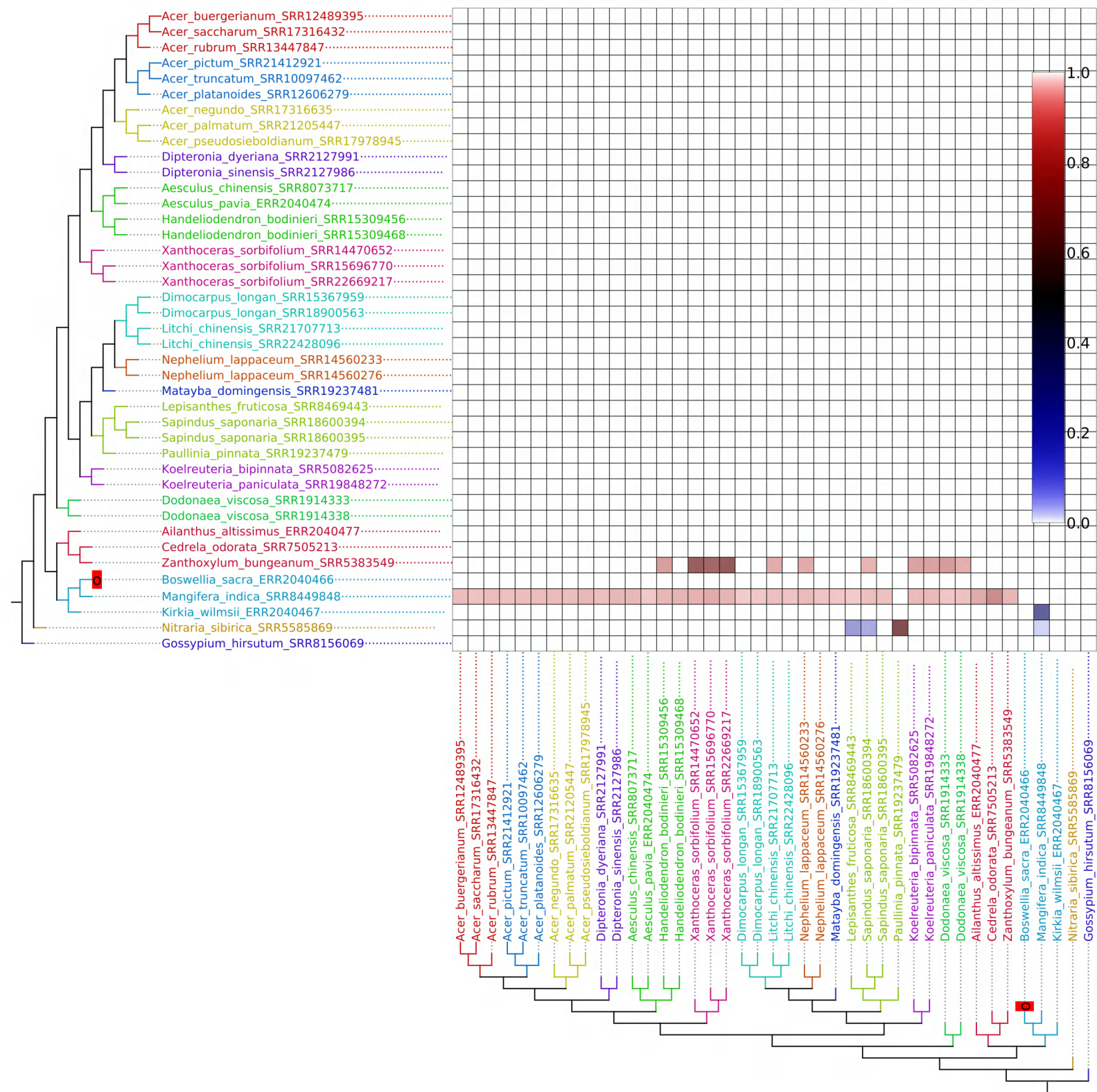

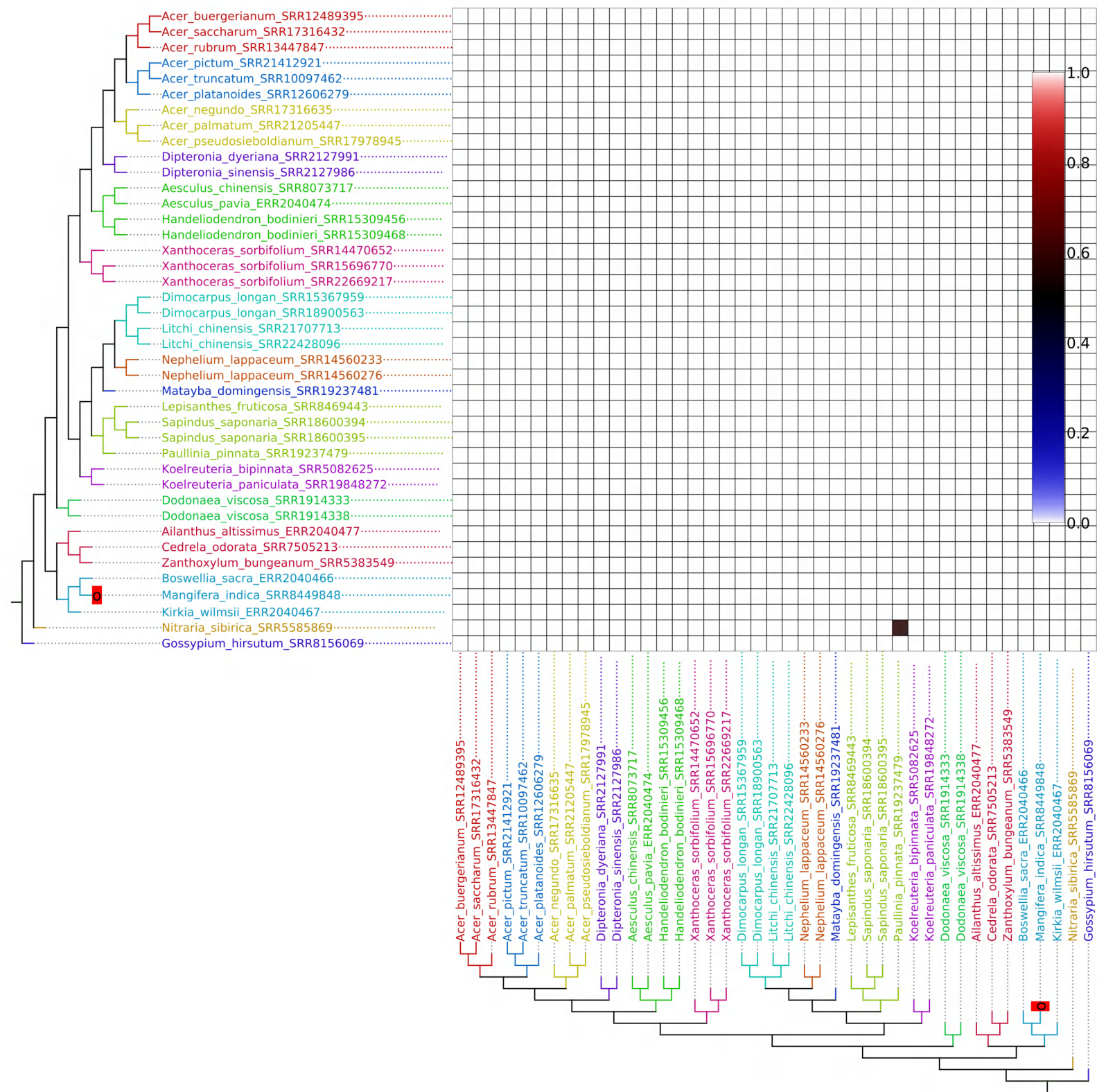

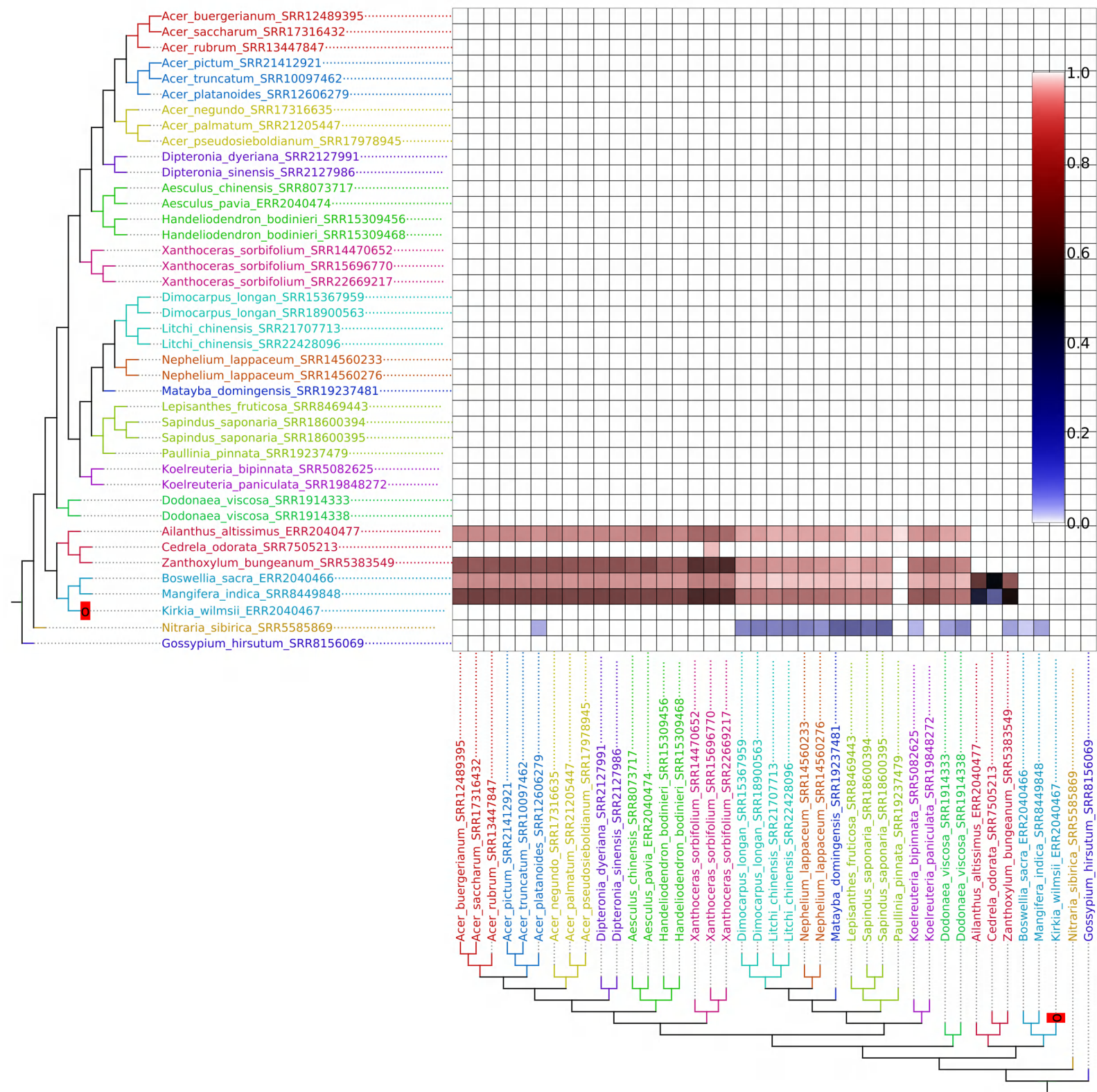

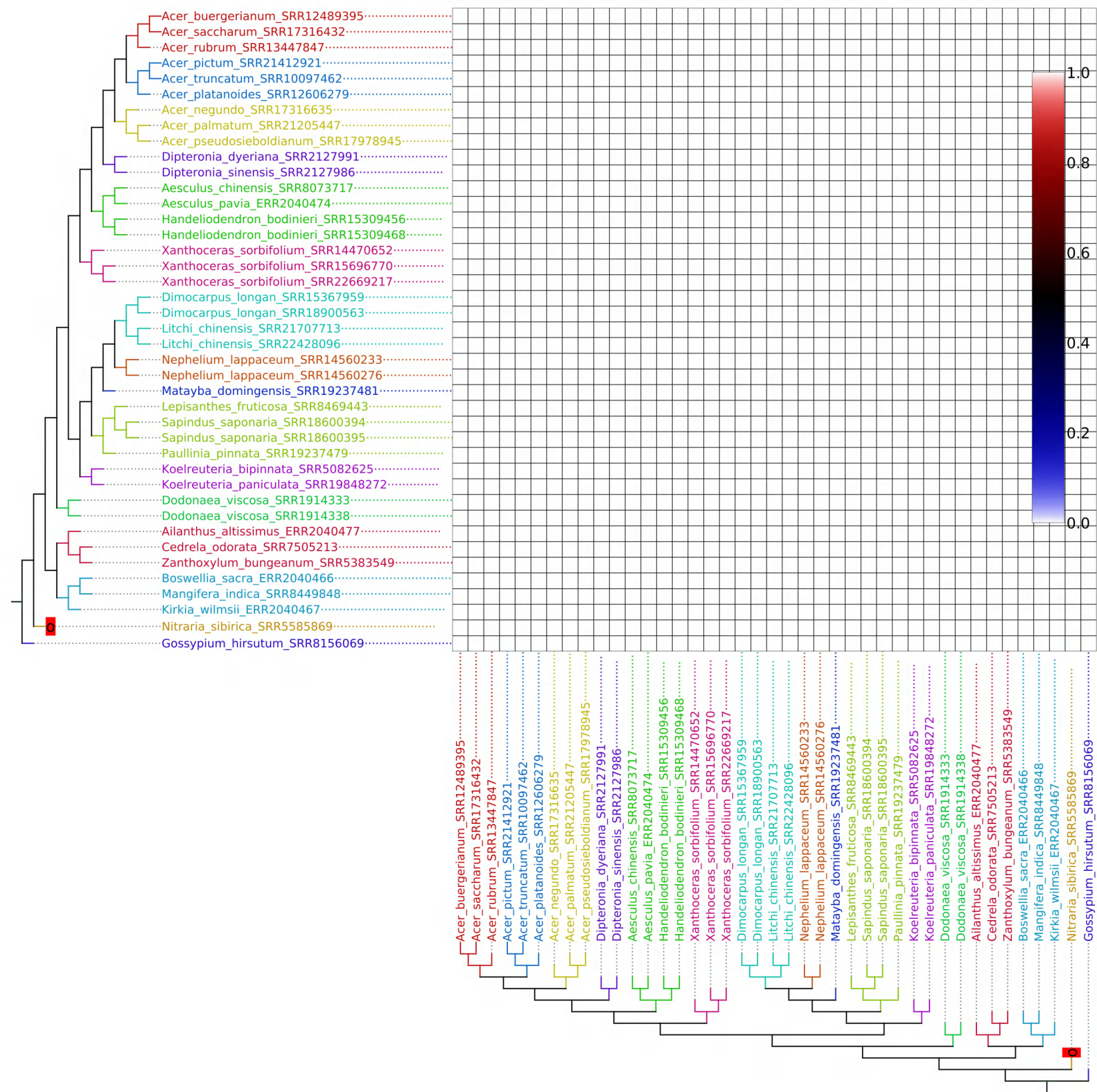

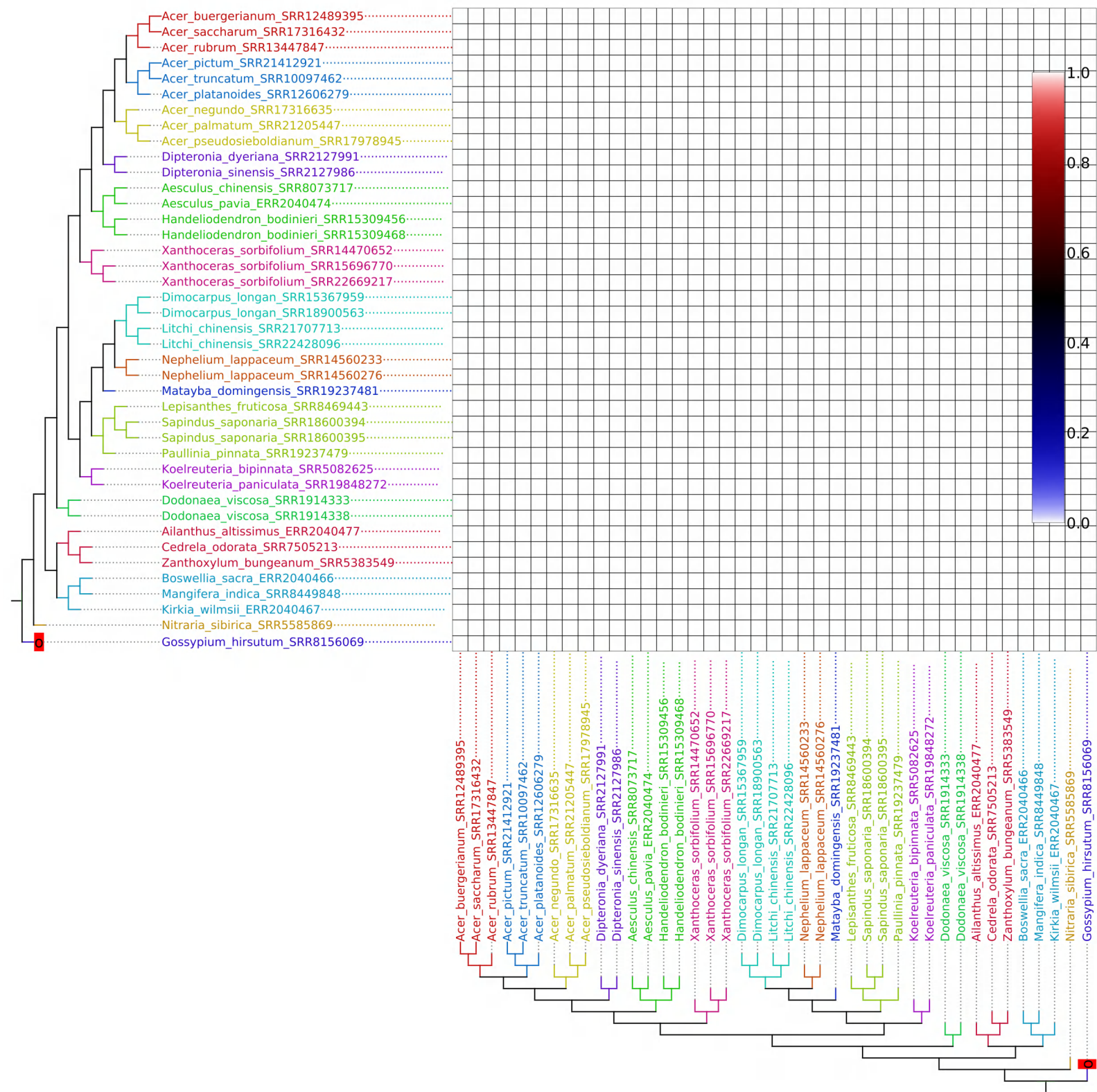

Supplement: Supplementary file 1 [file ijms-26-01581-s001.zip › Figure_S7.pdf]

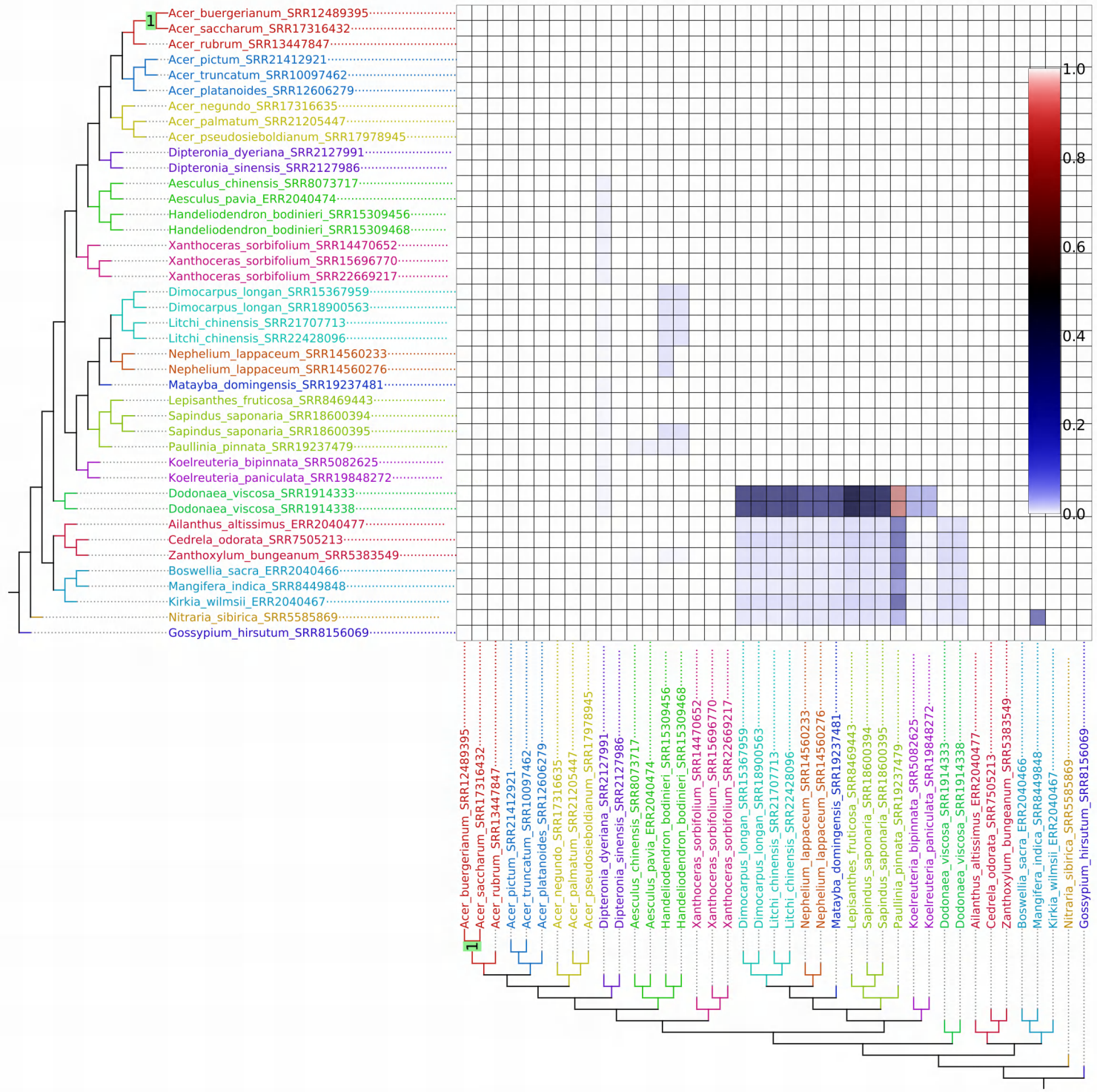

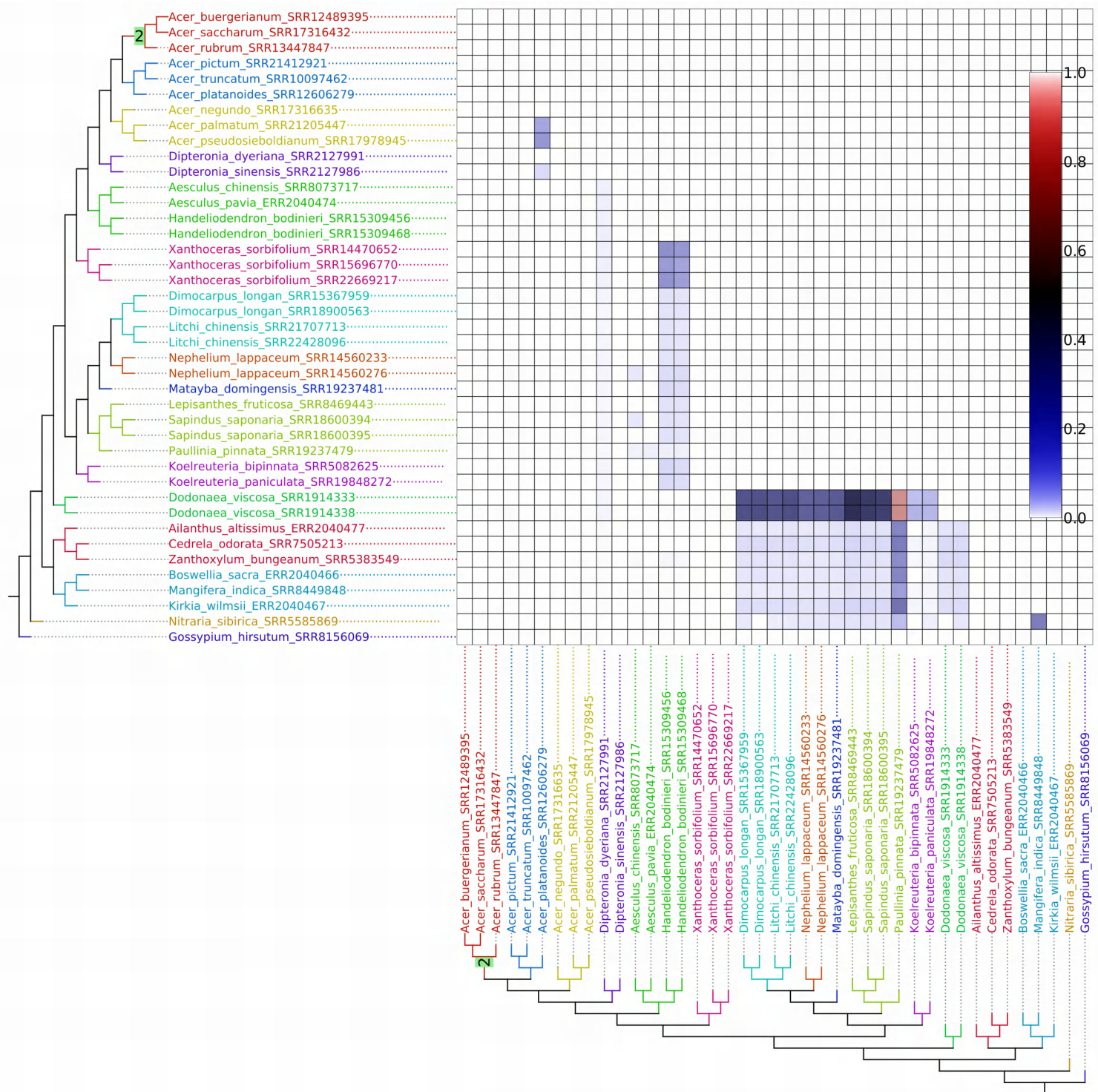

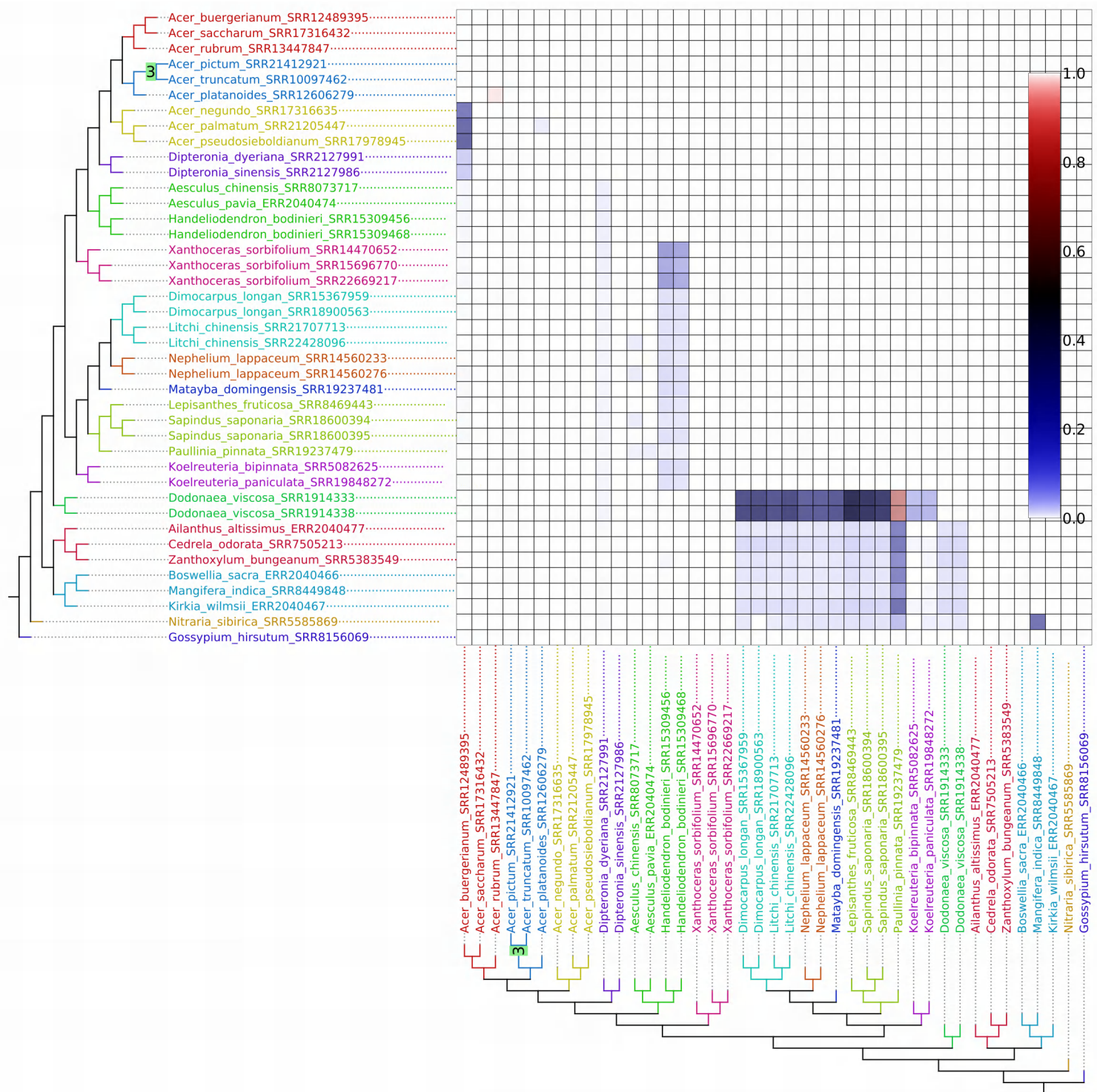

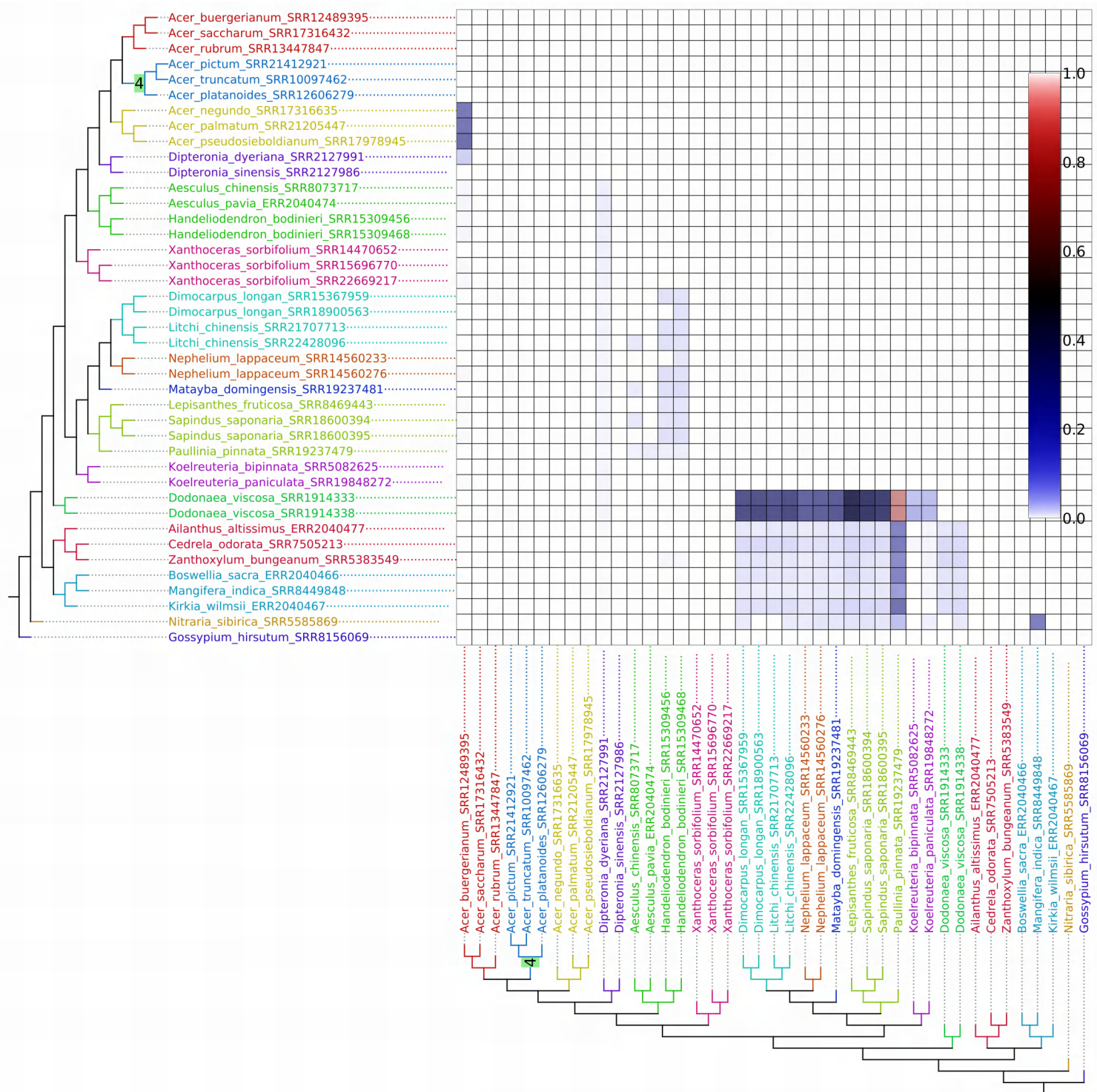

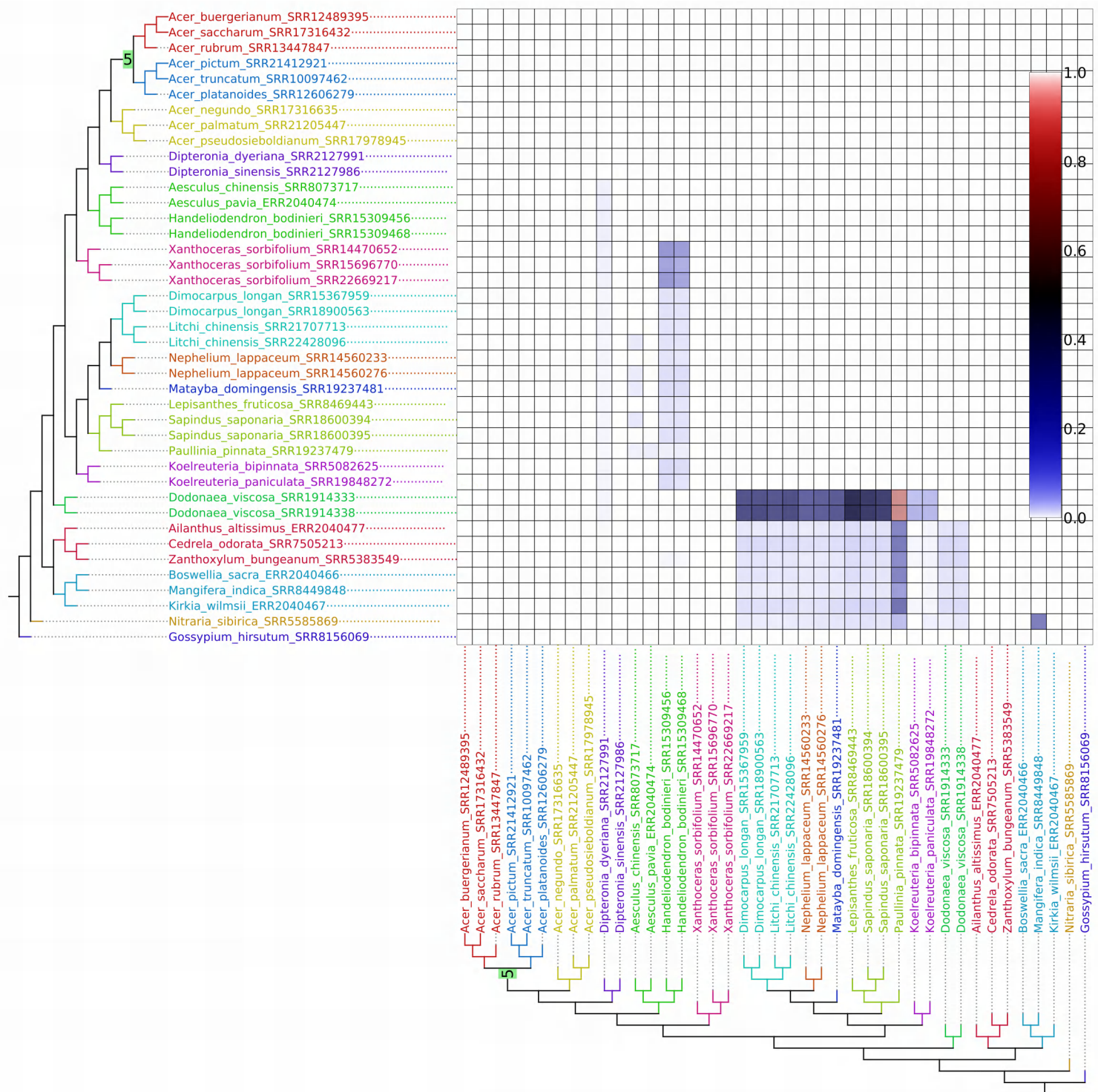

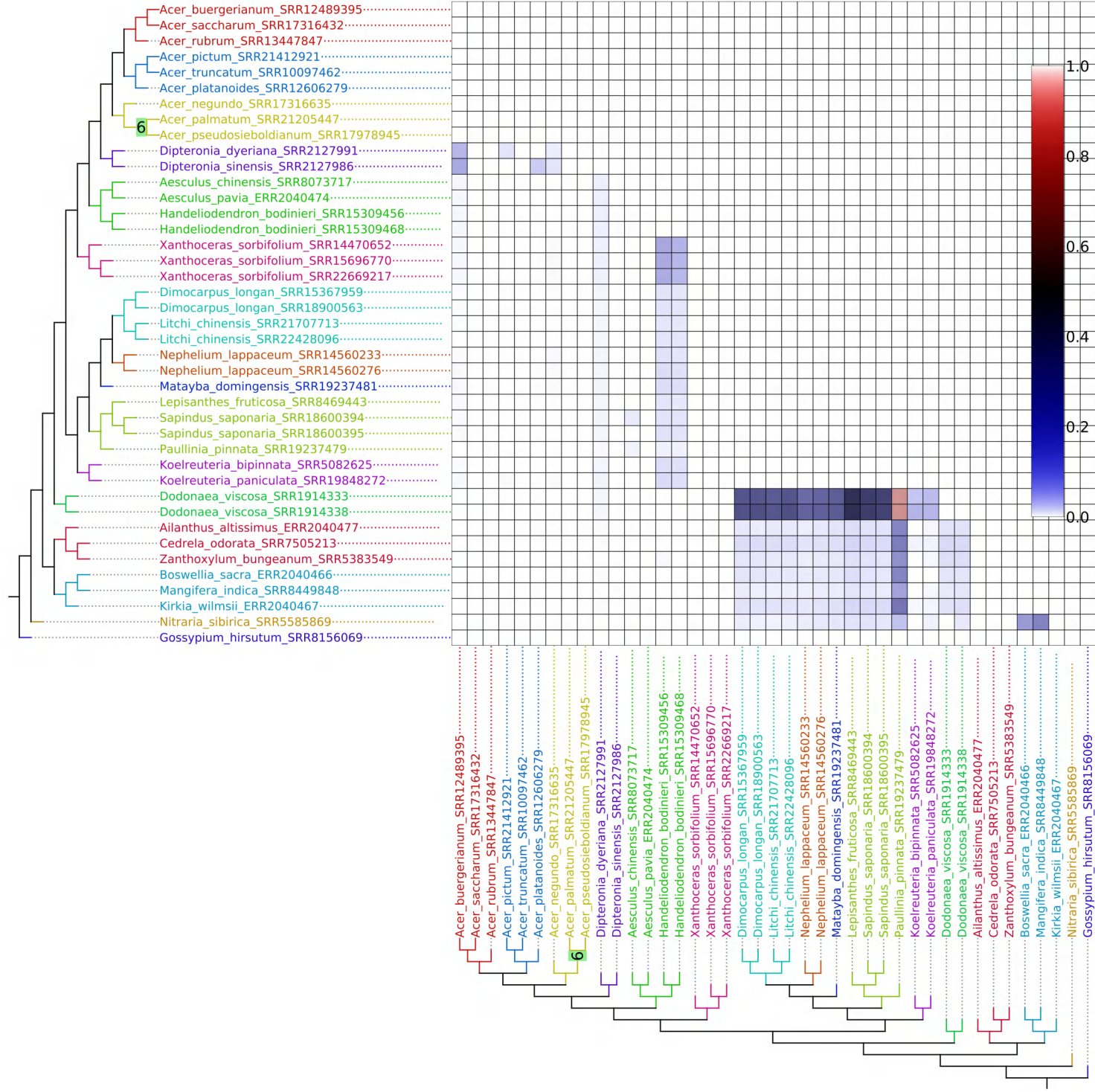

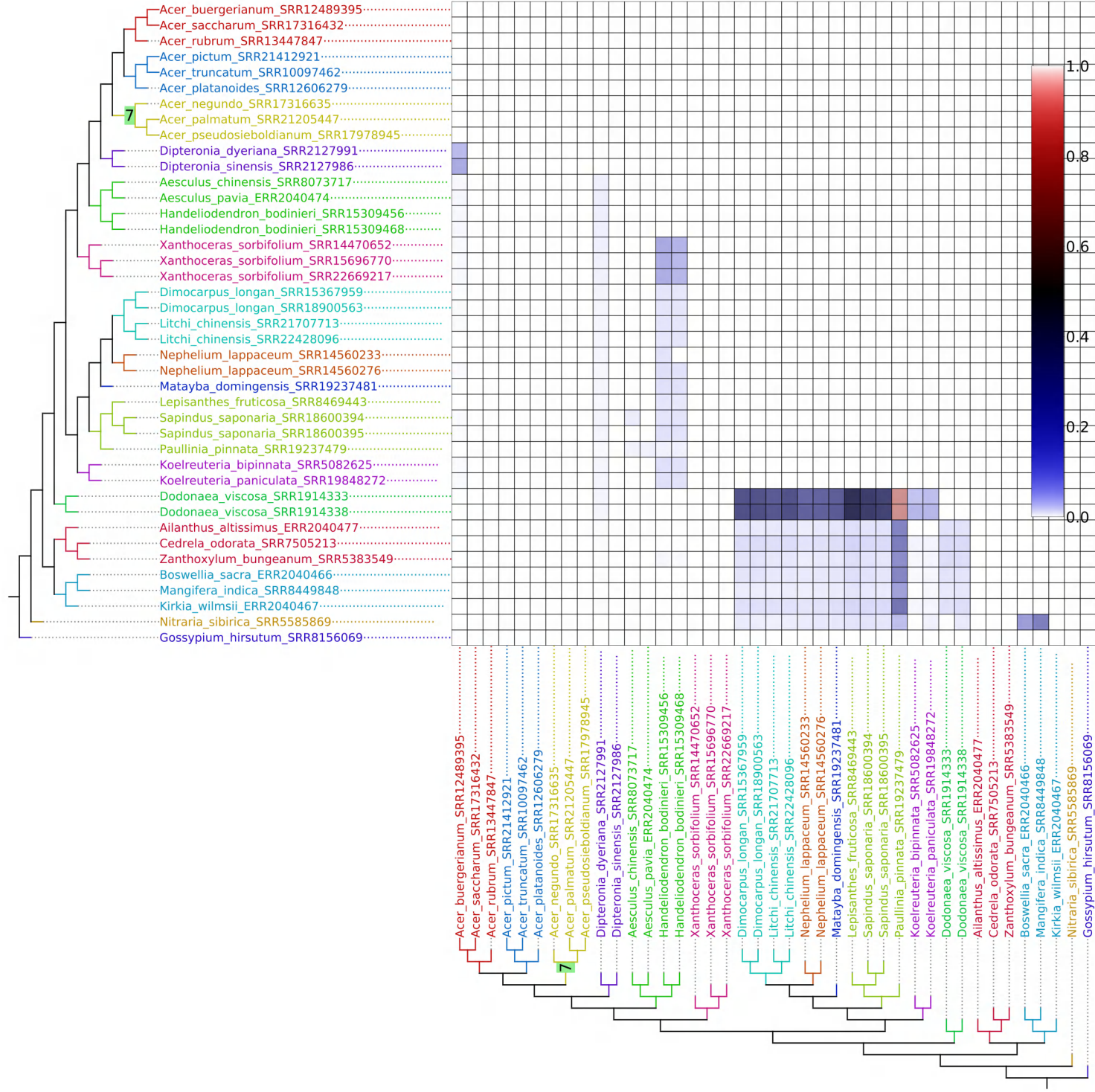

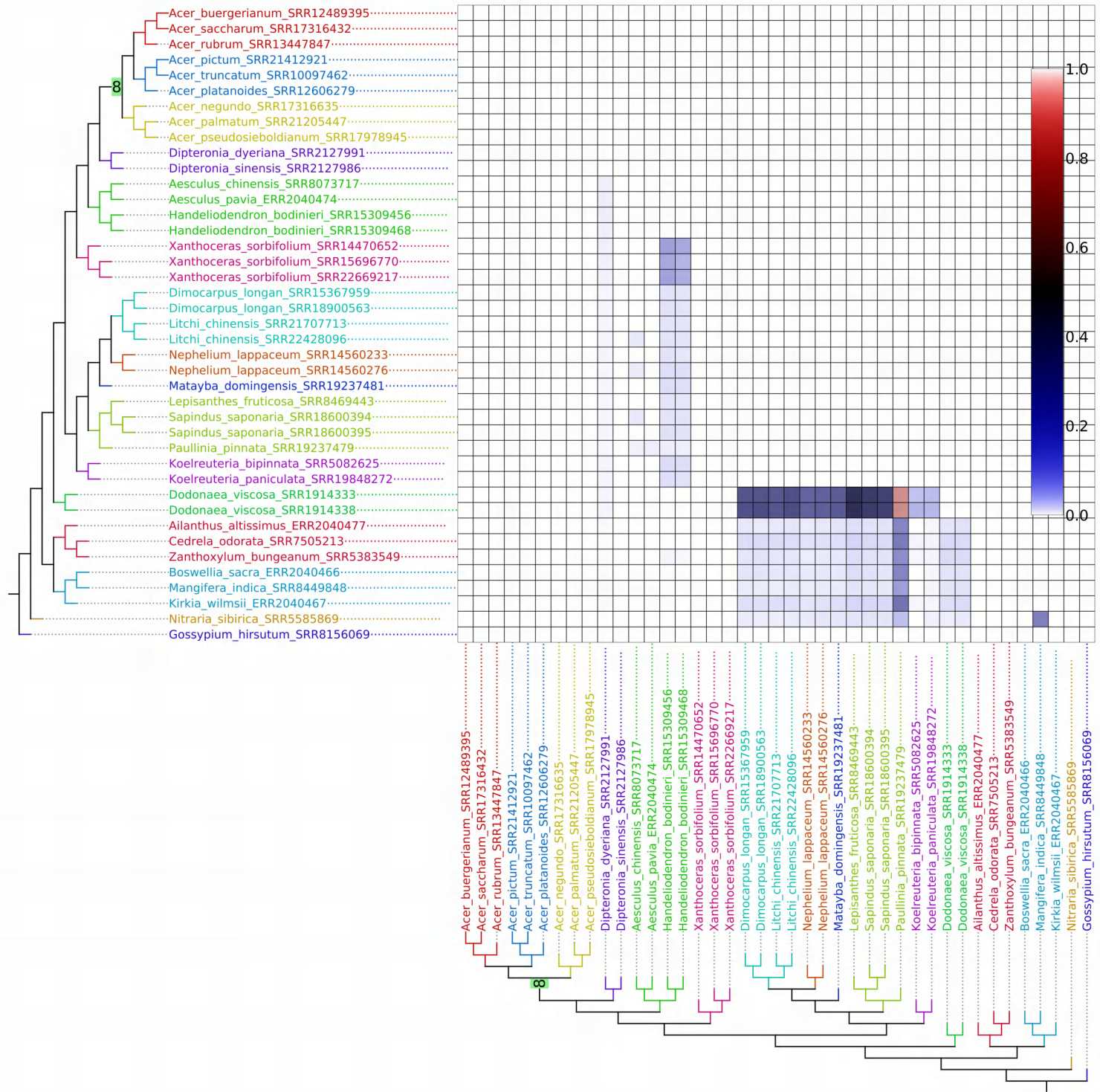

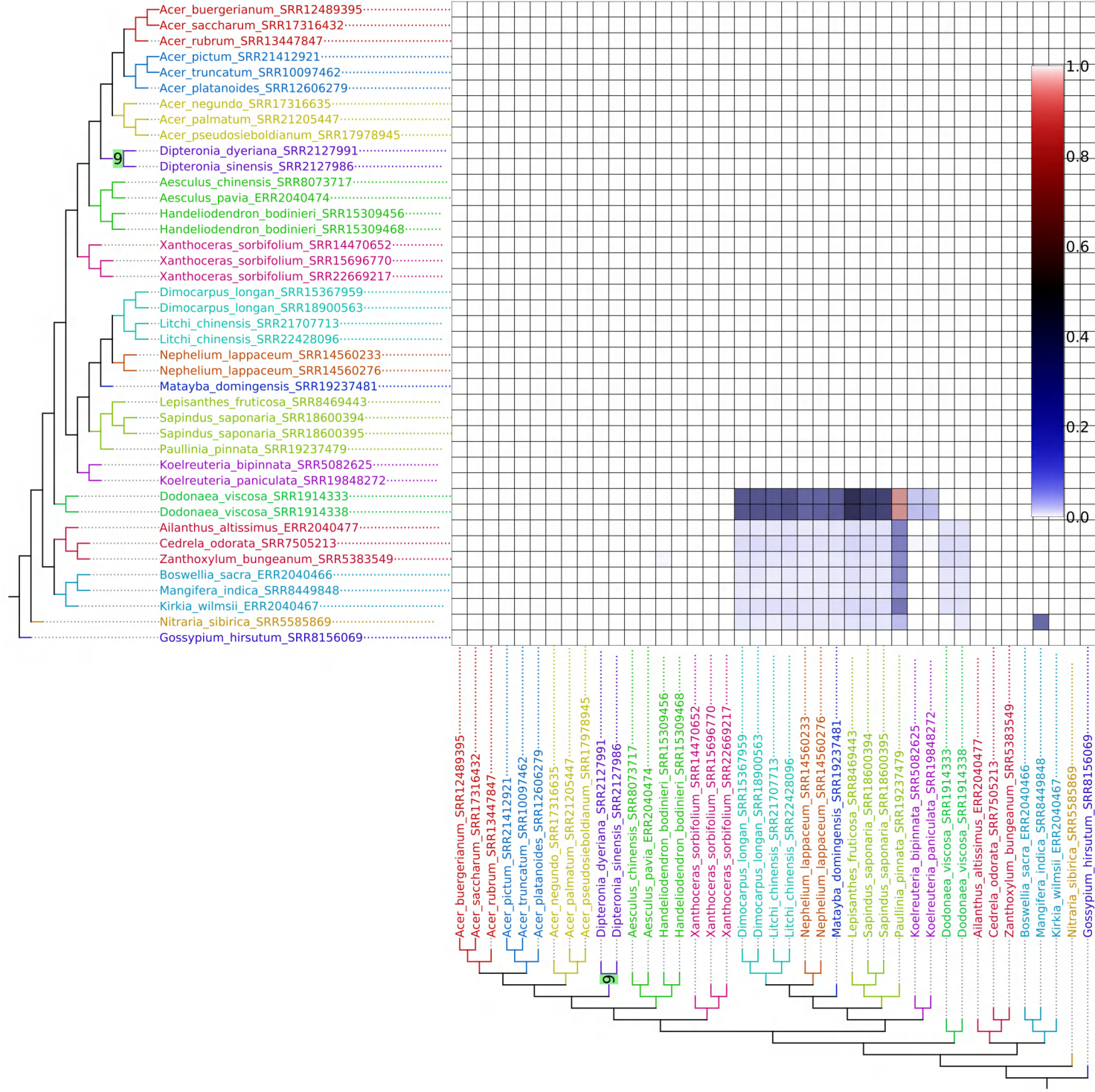

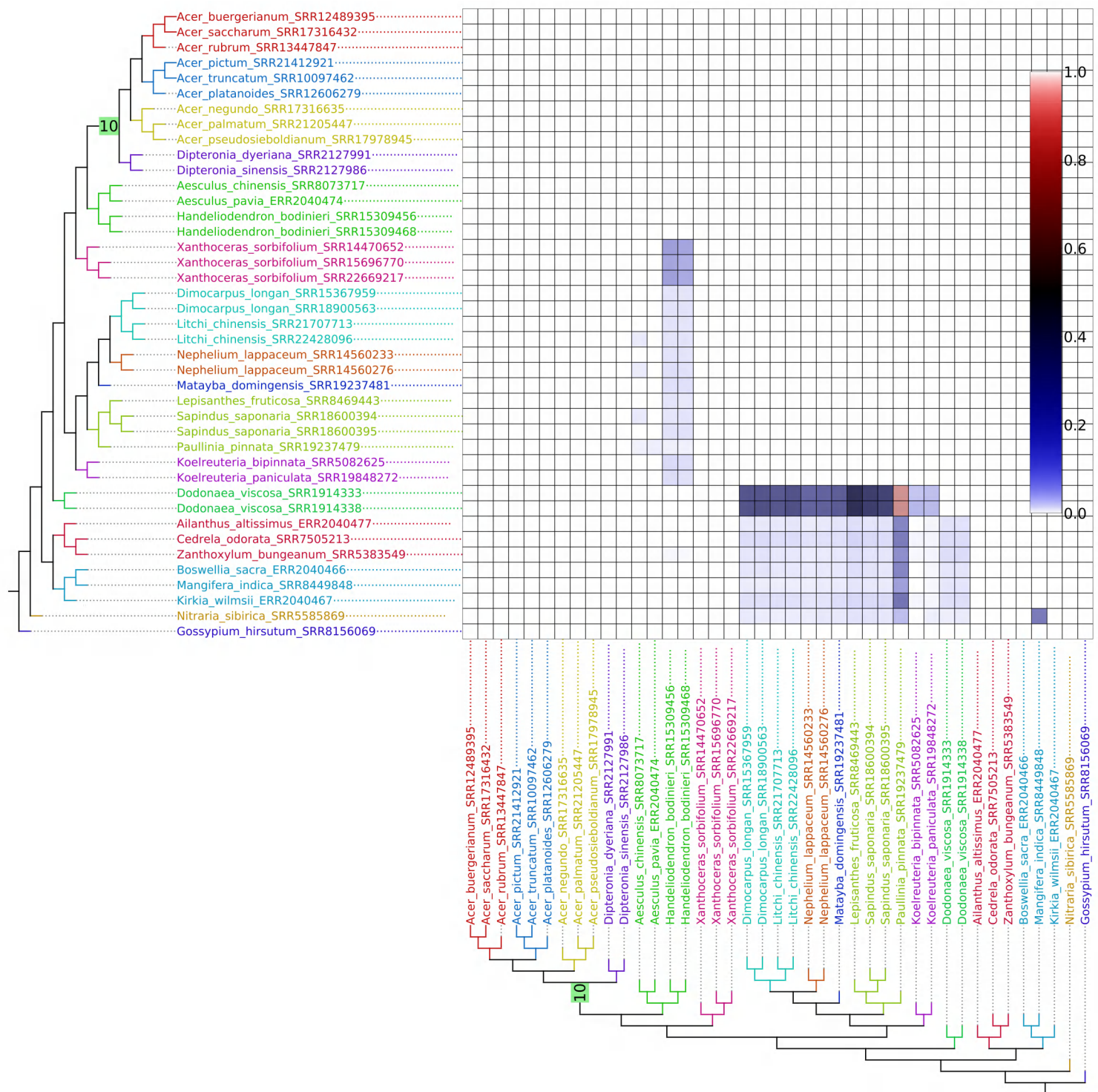

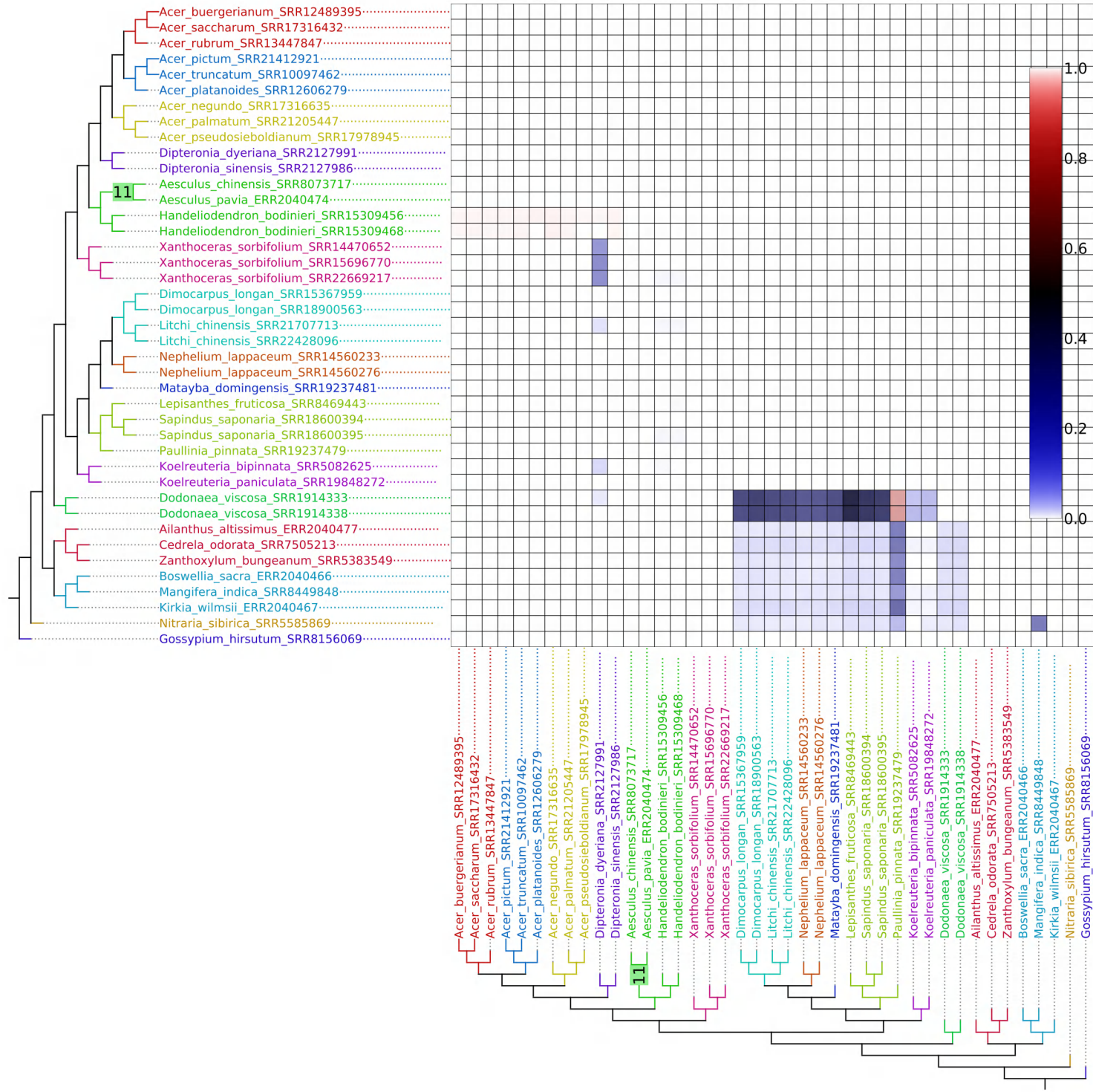

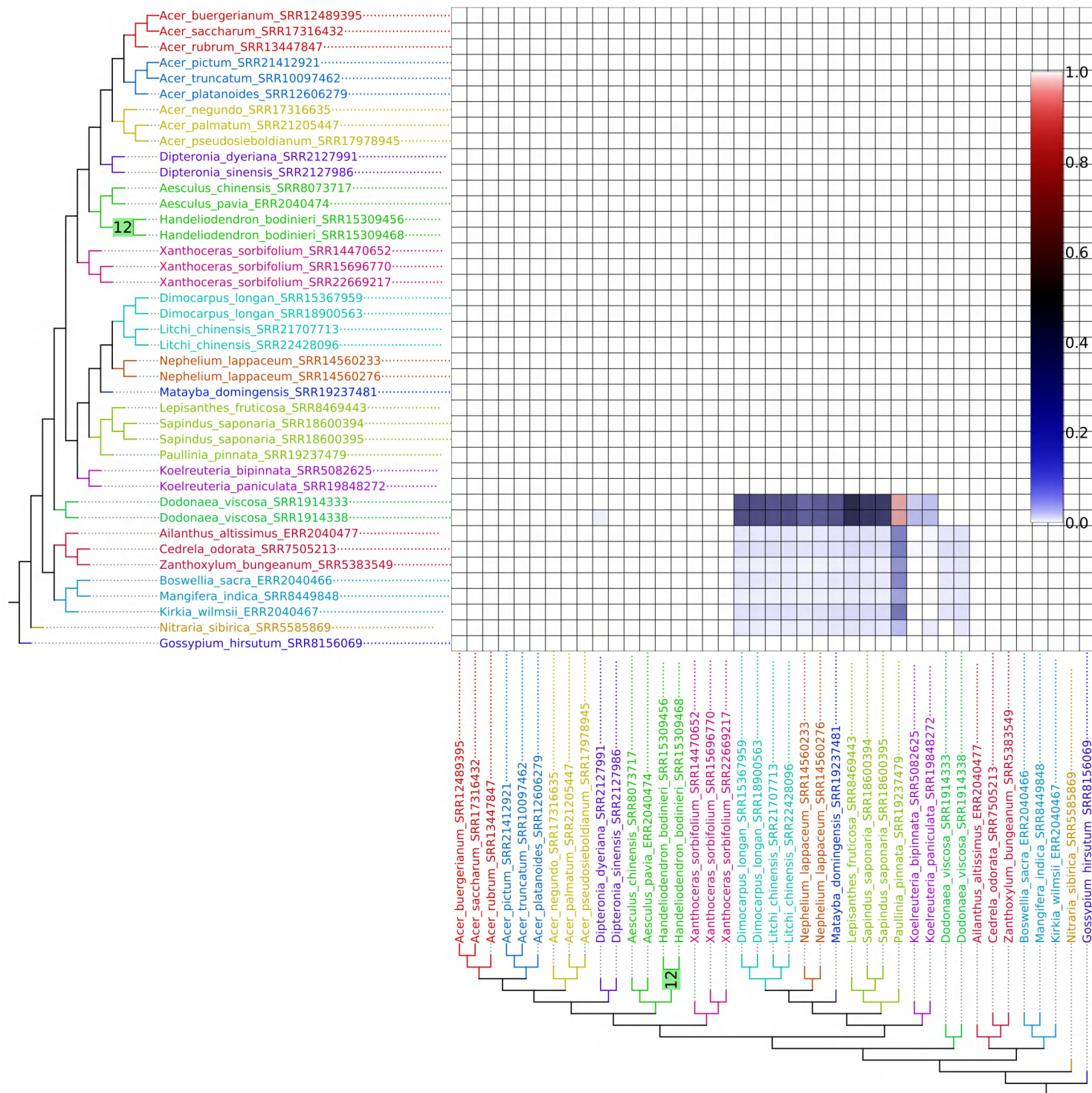

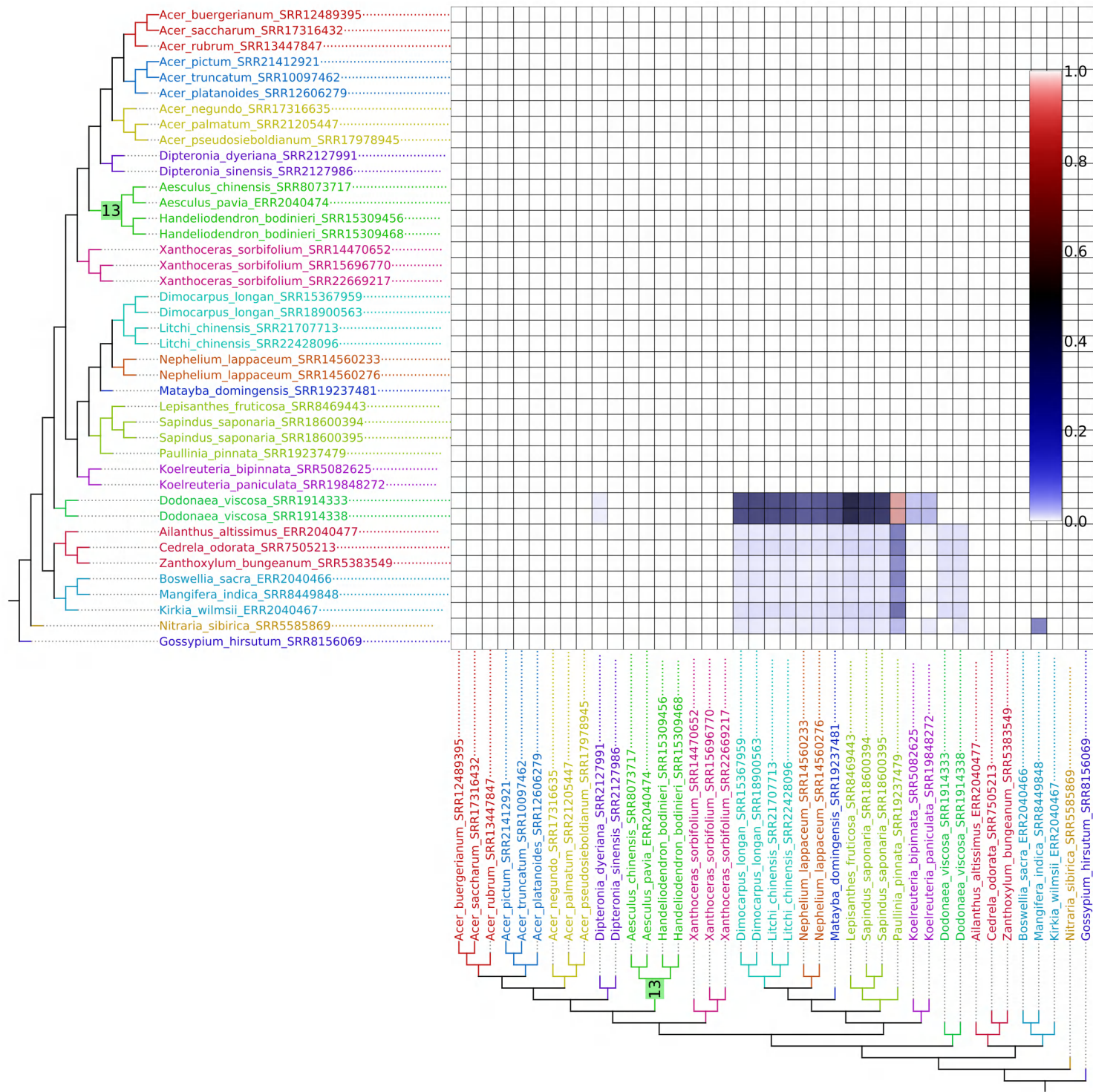

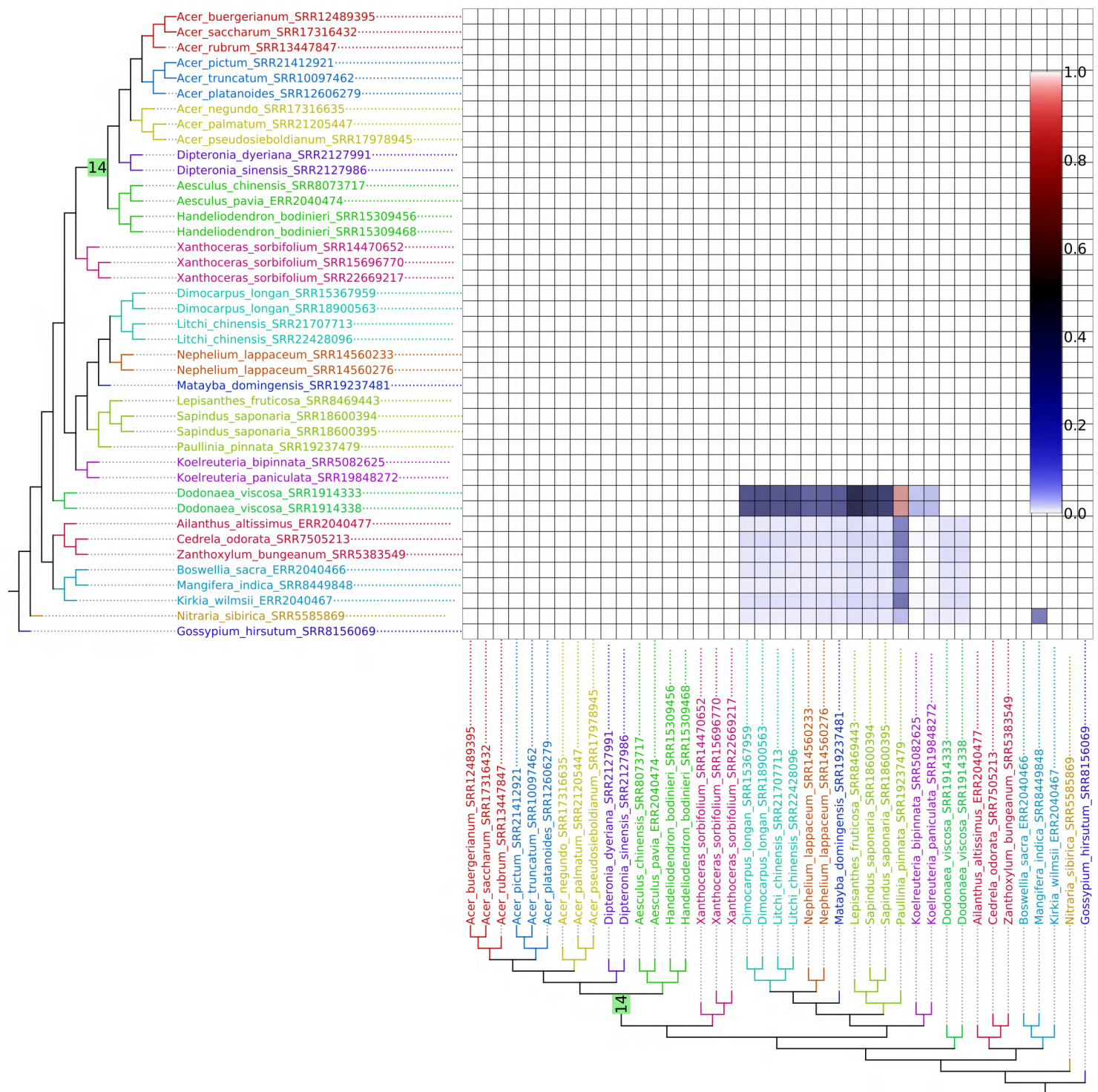

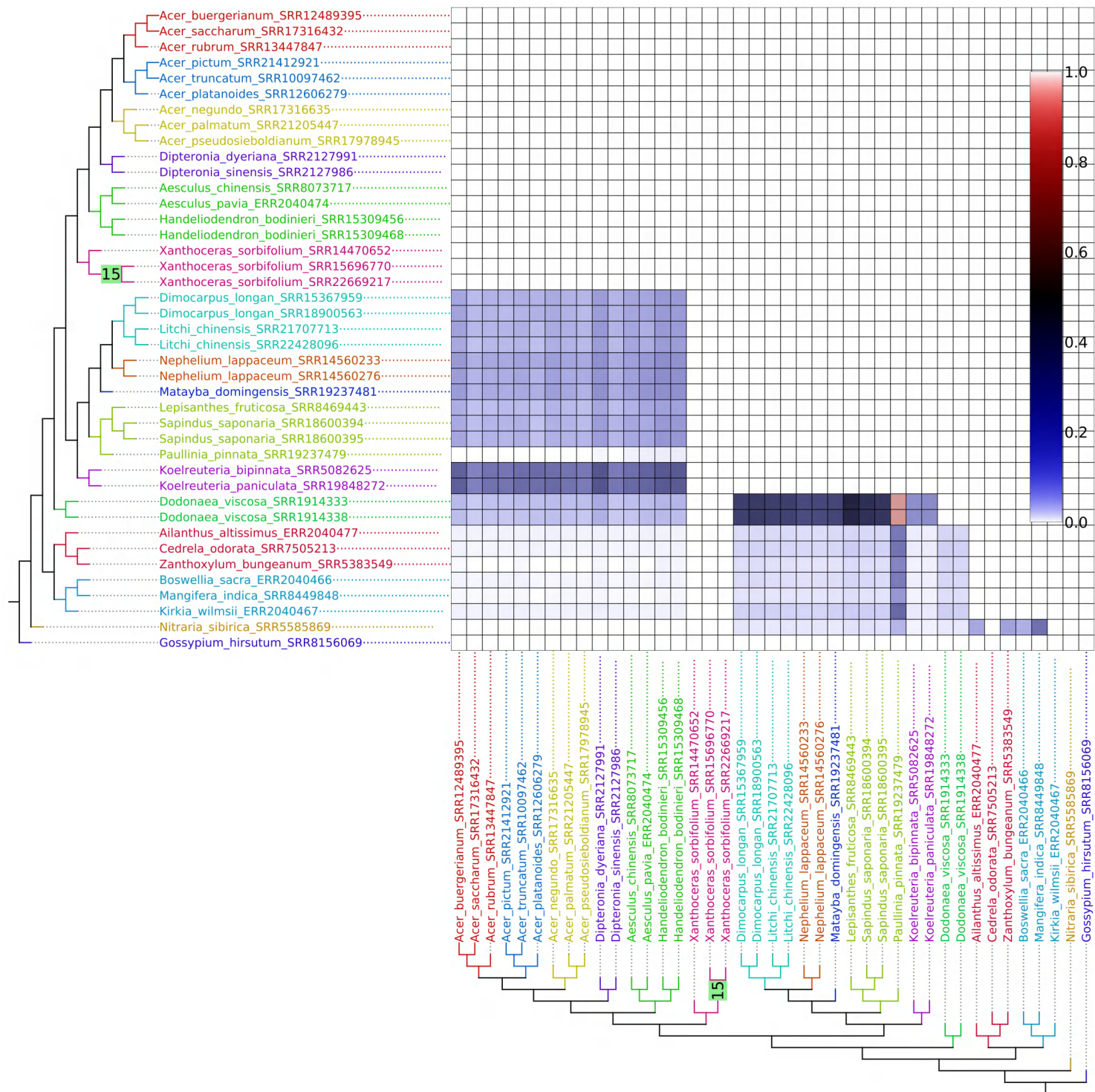

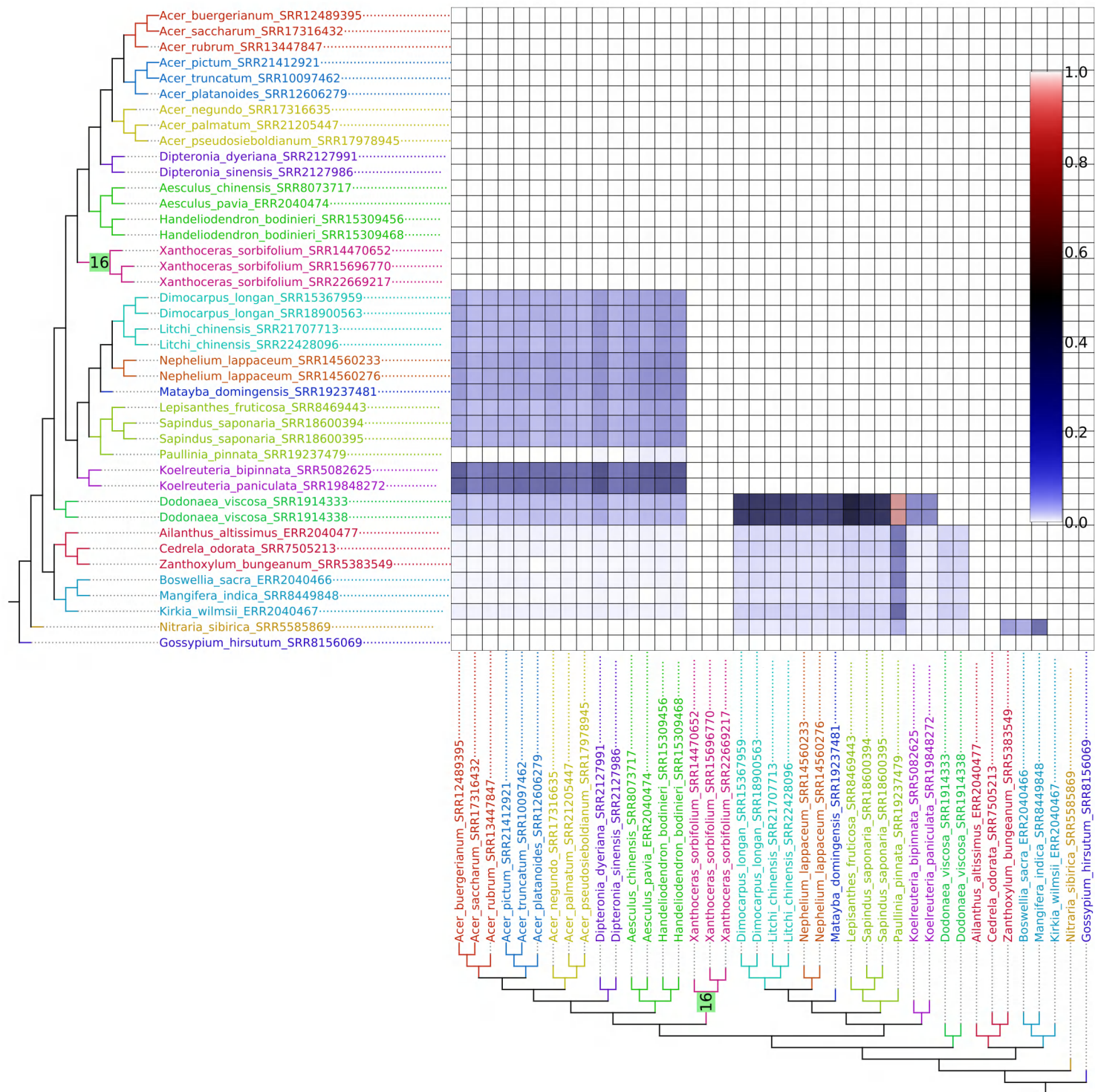

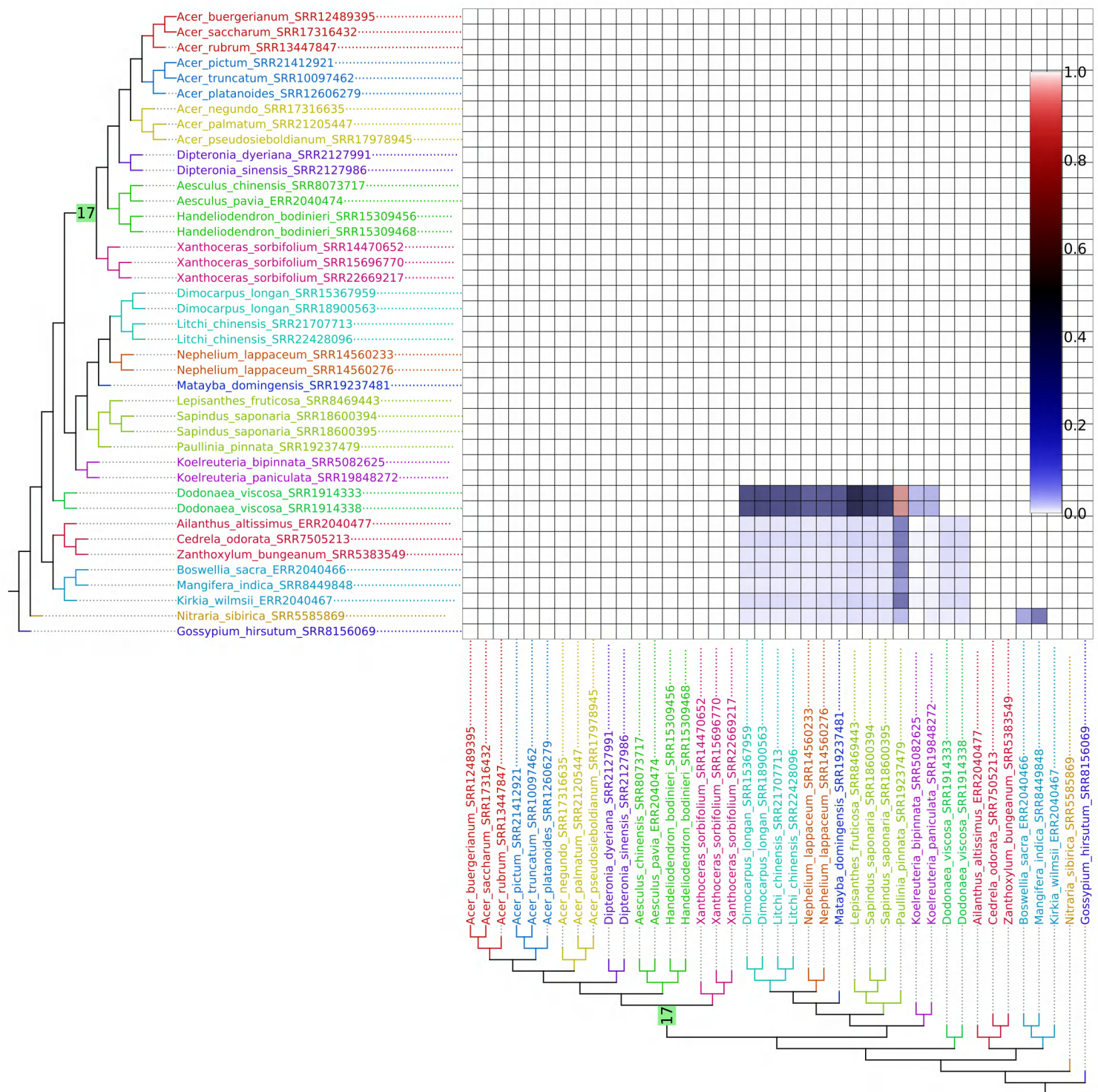

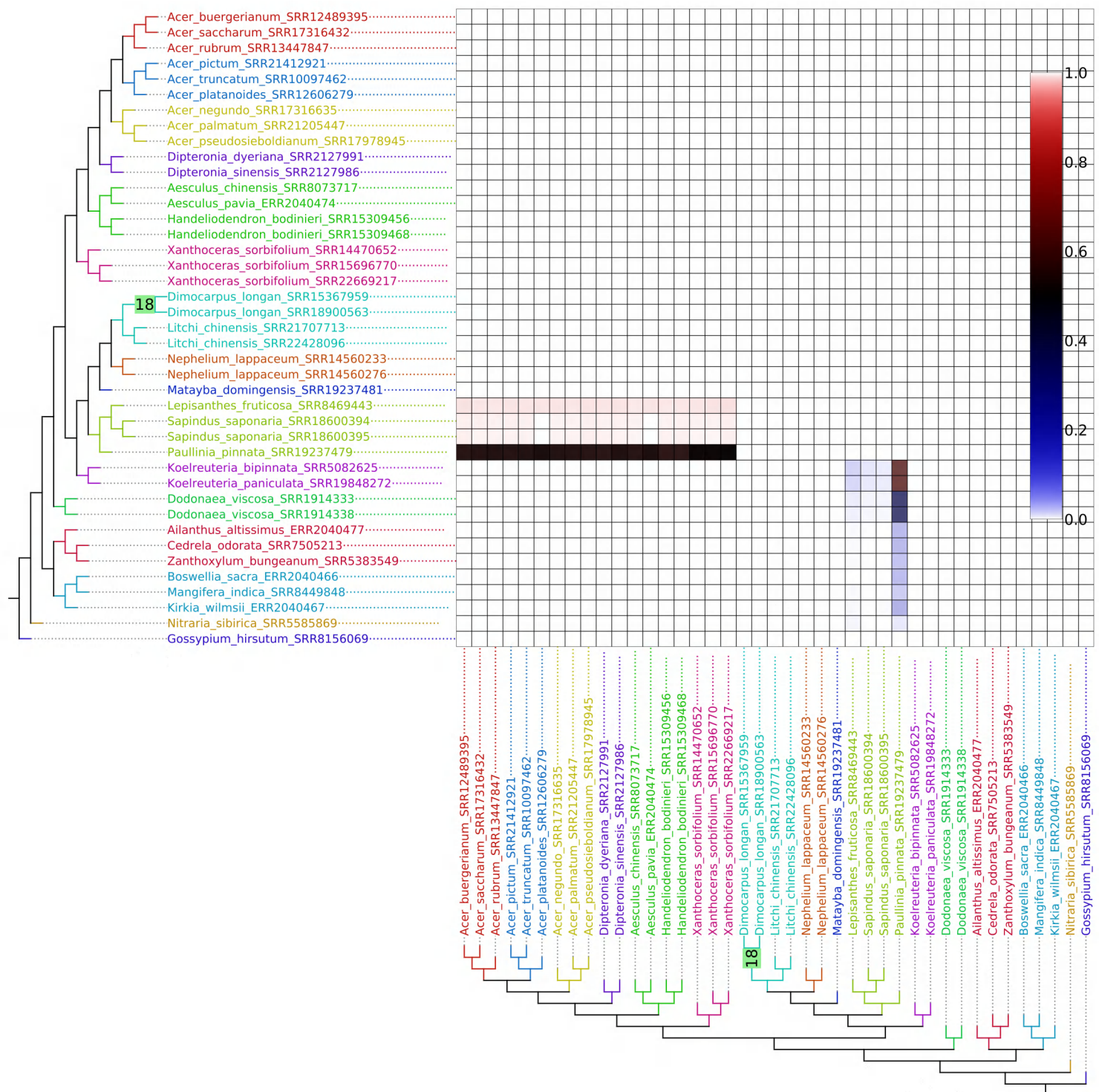

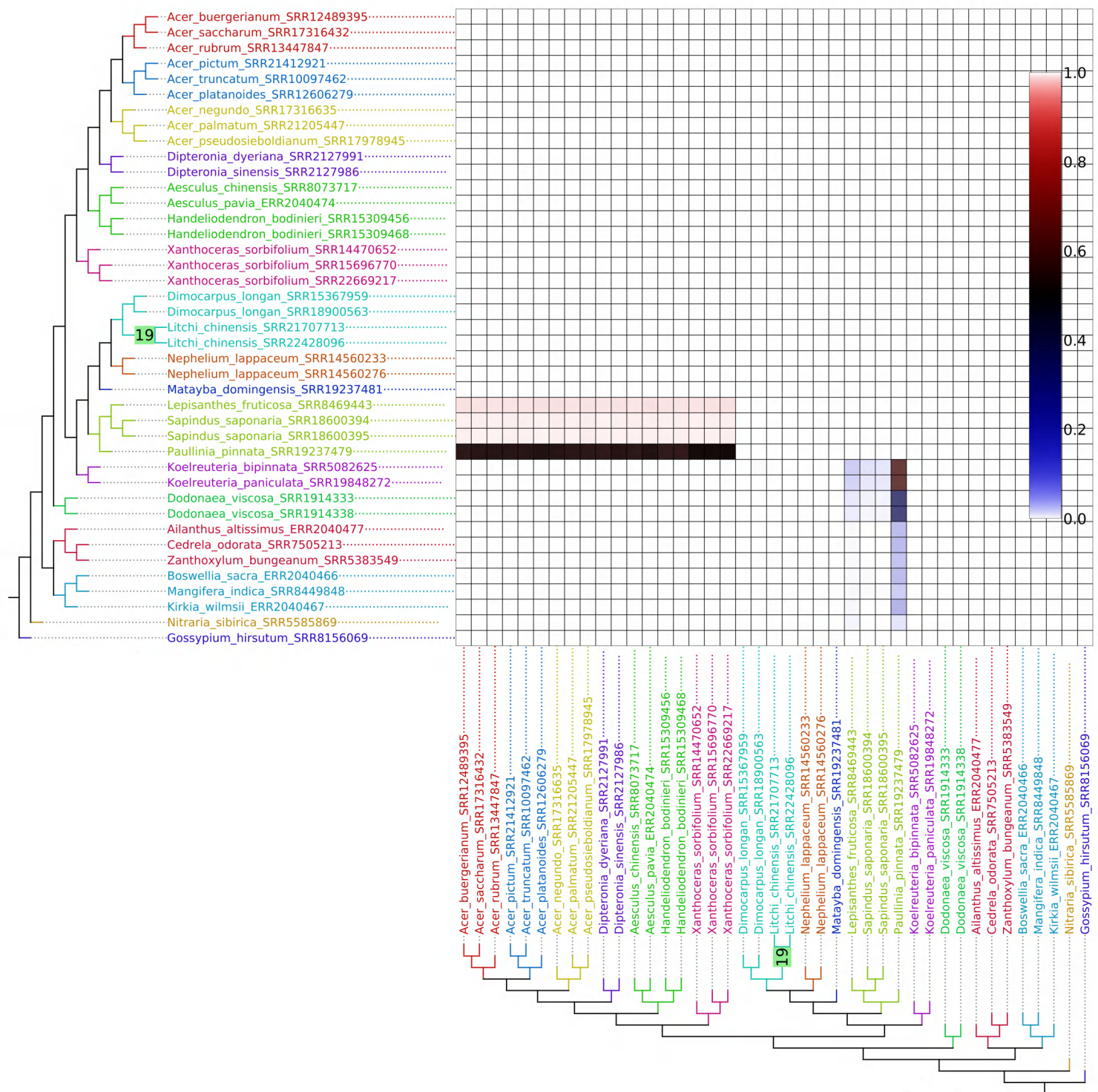

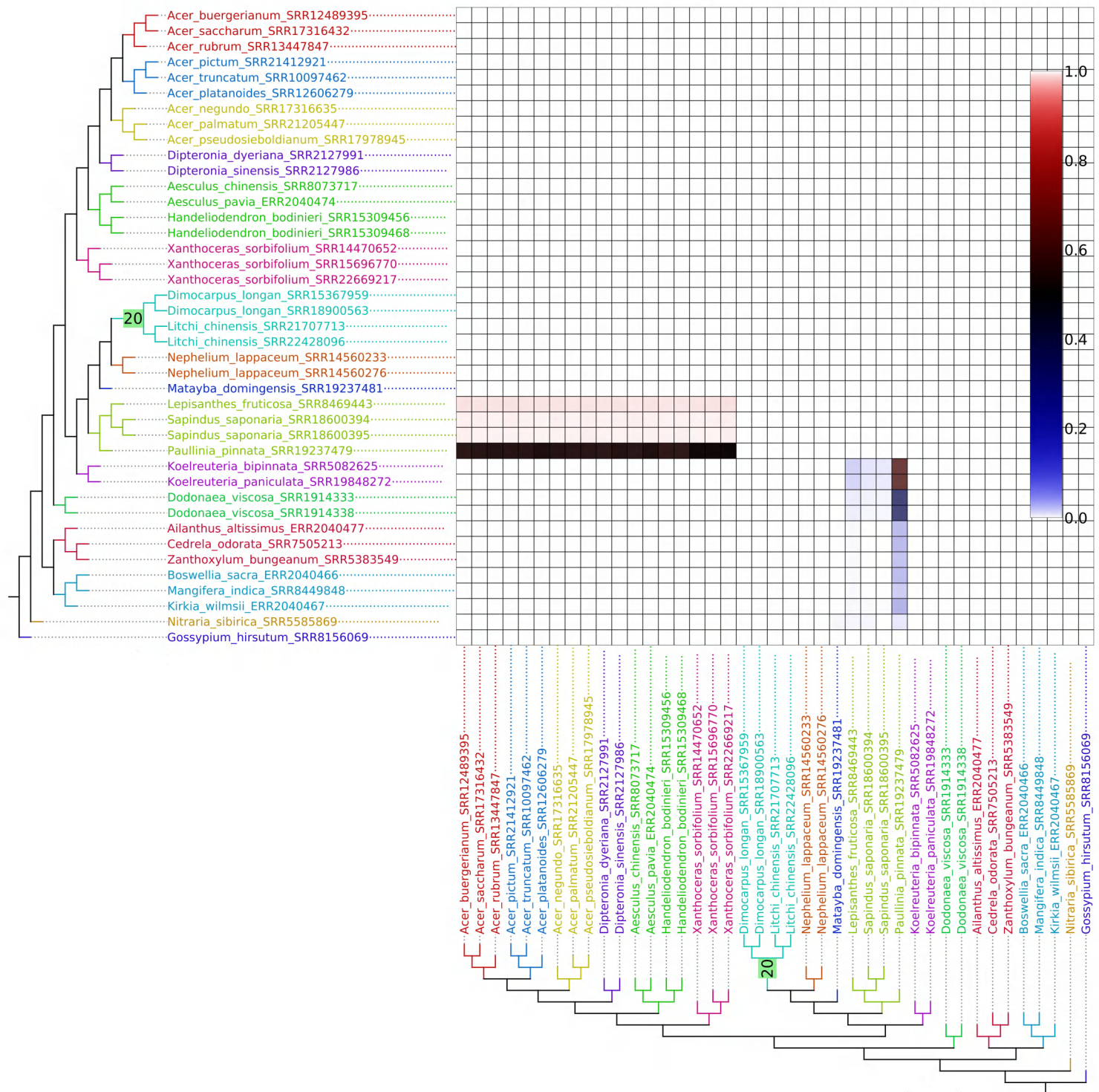

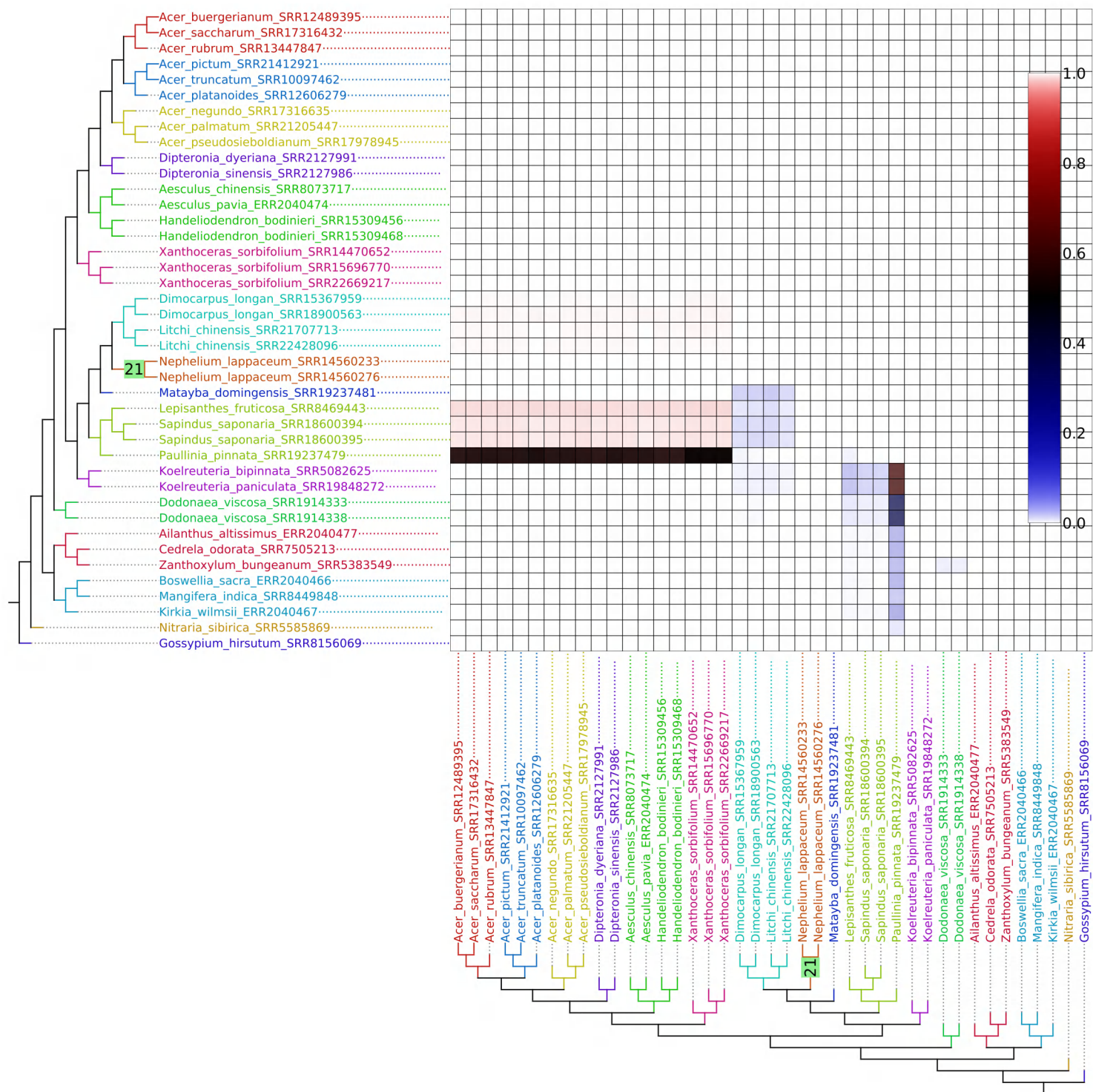

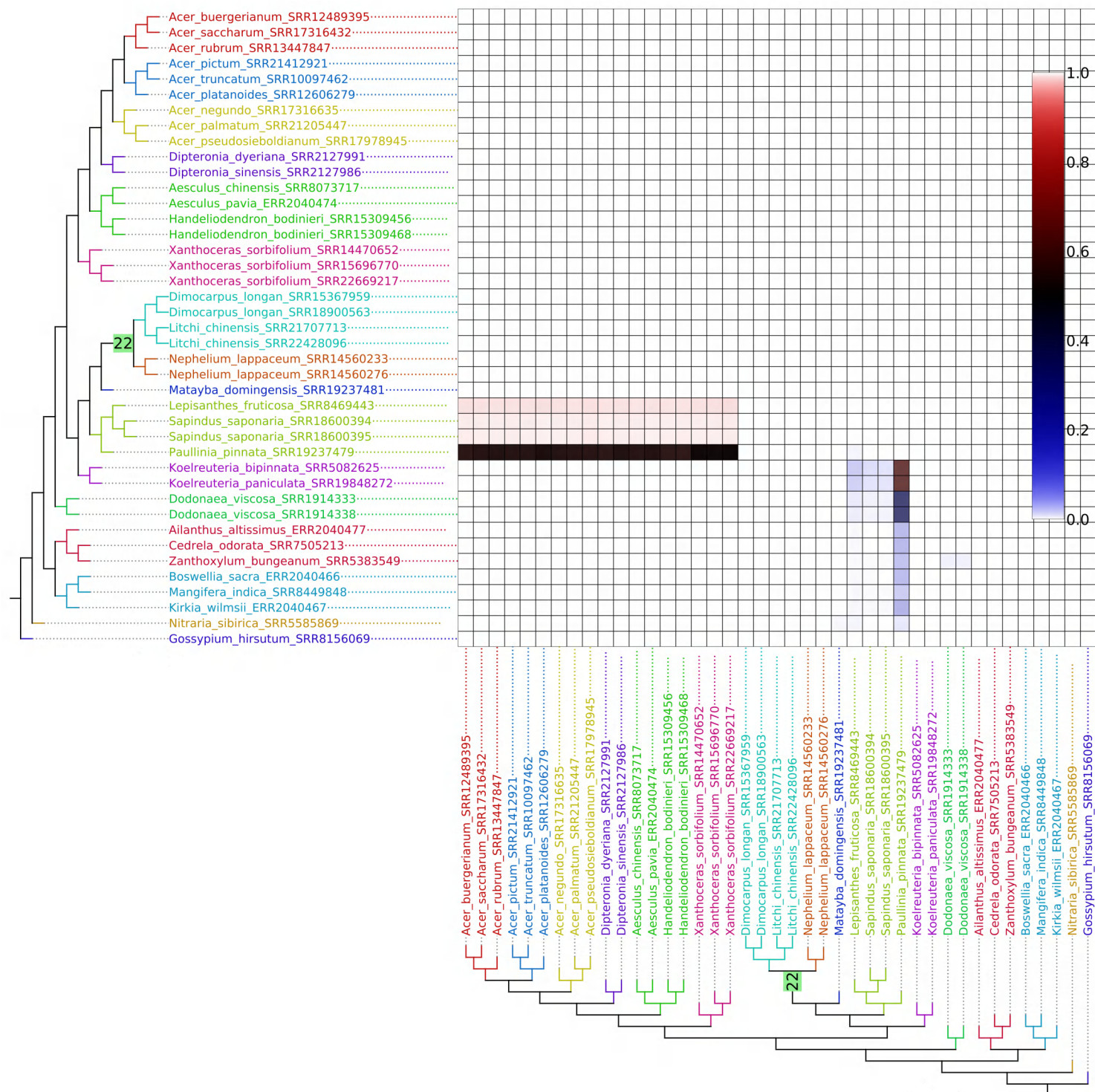

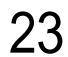

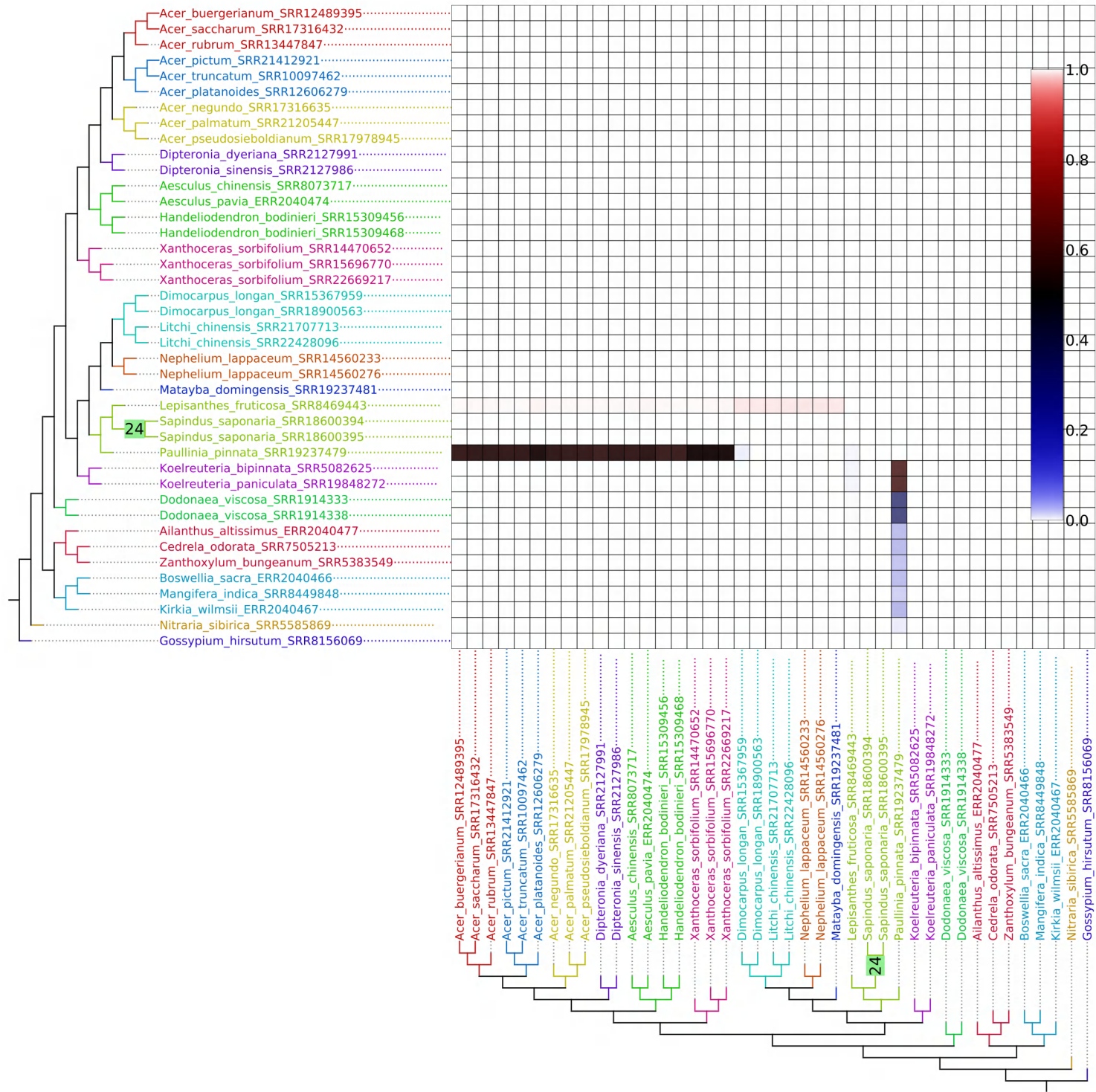

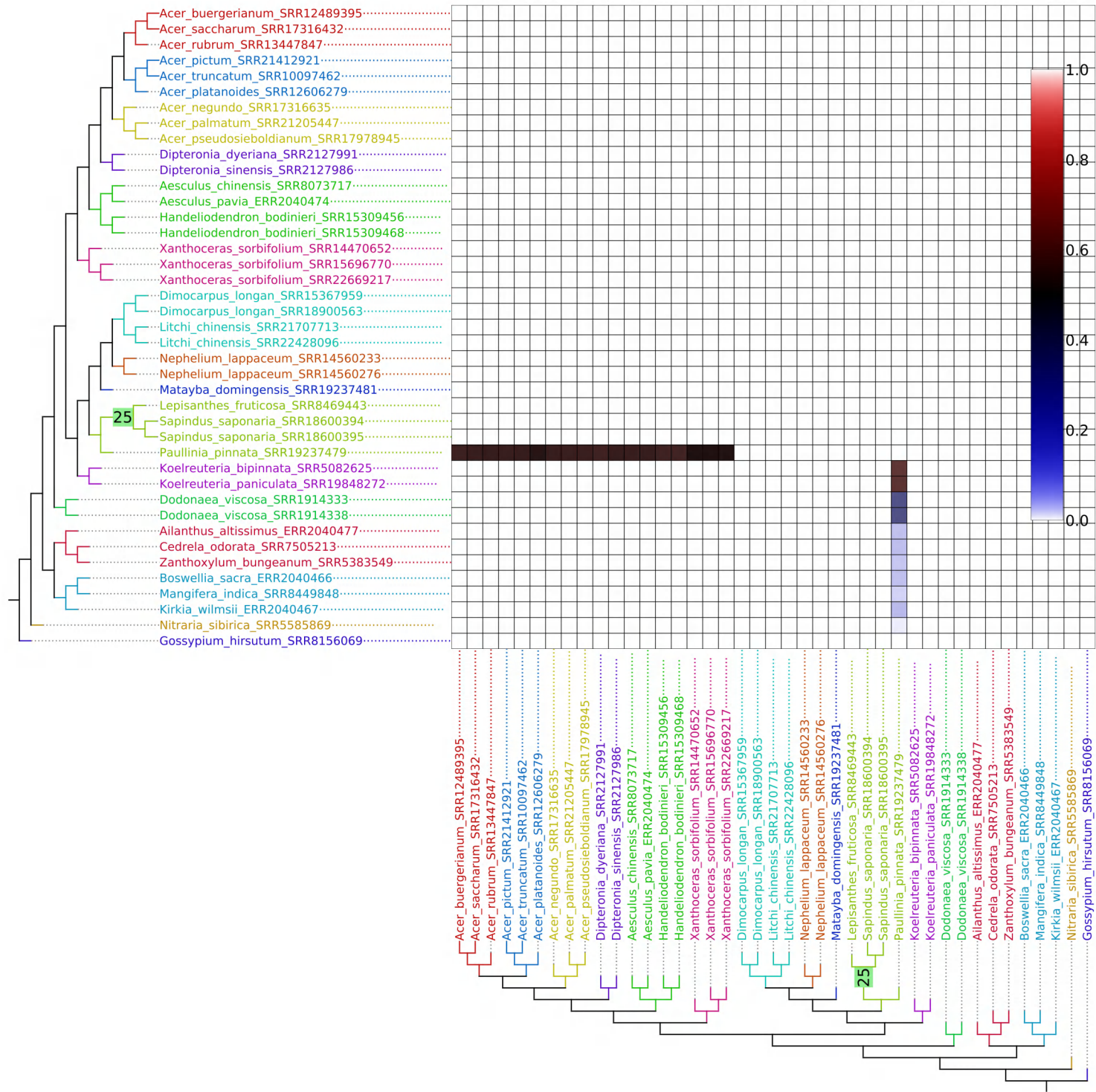

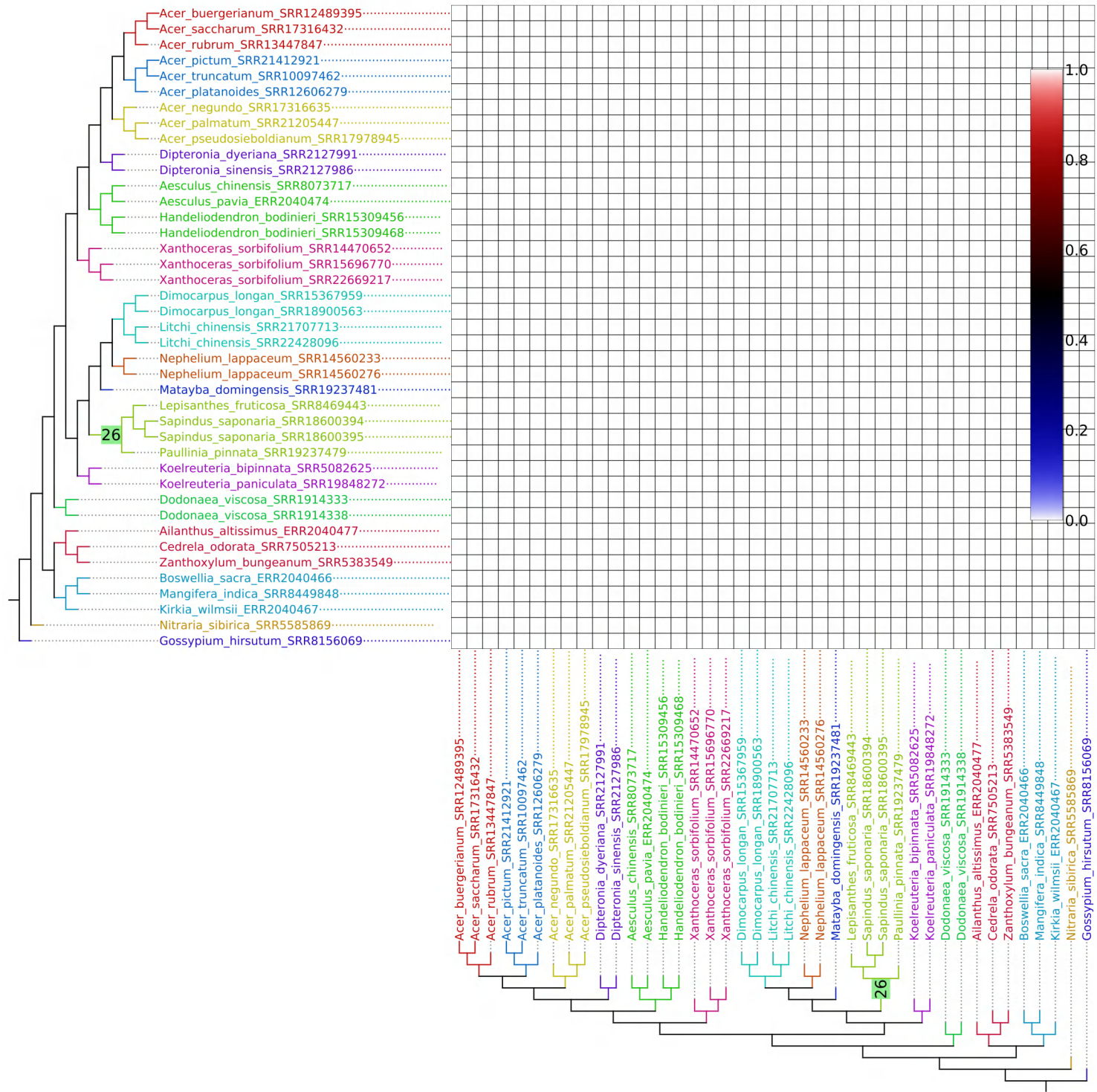

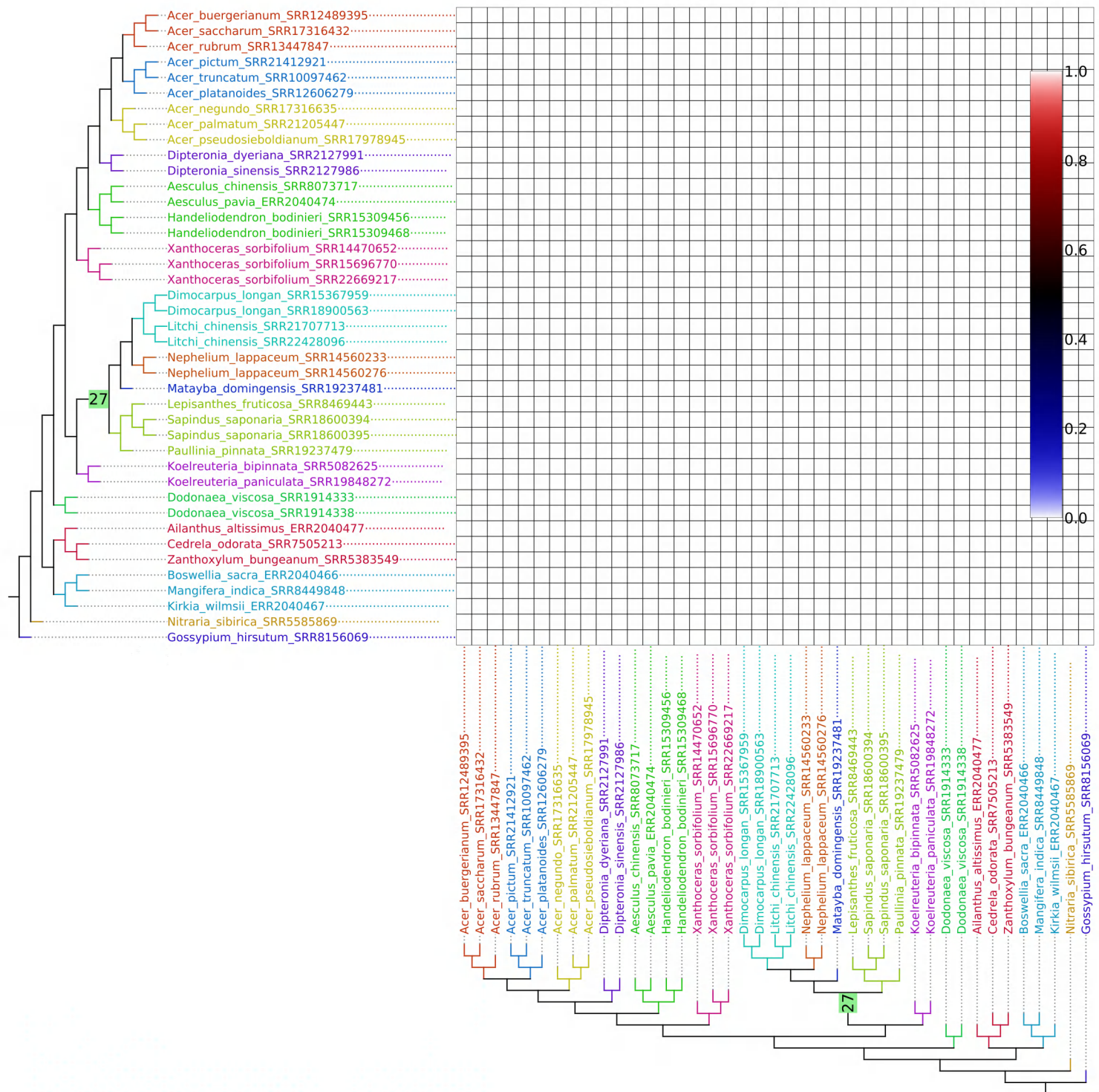

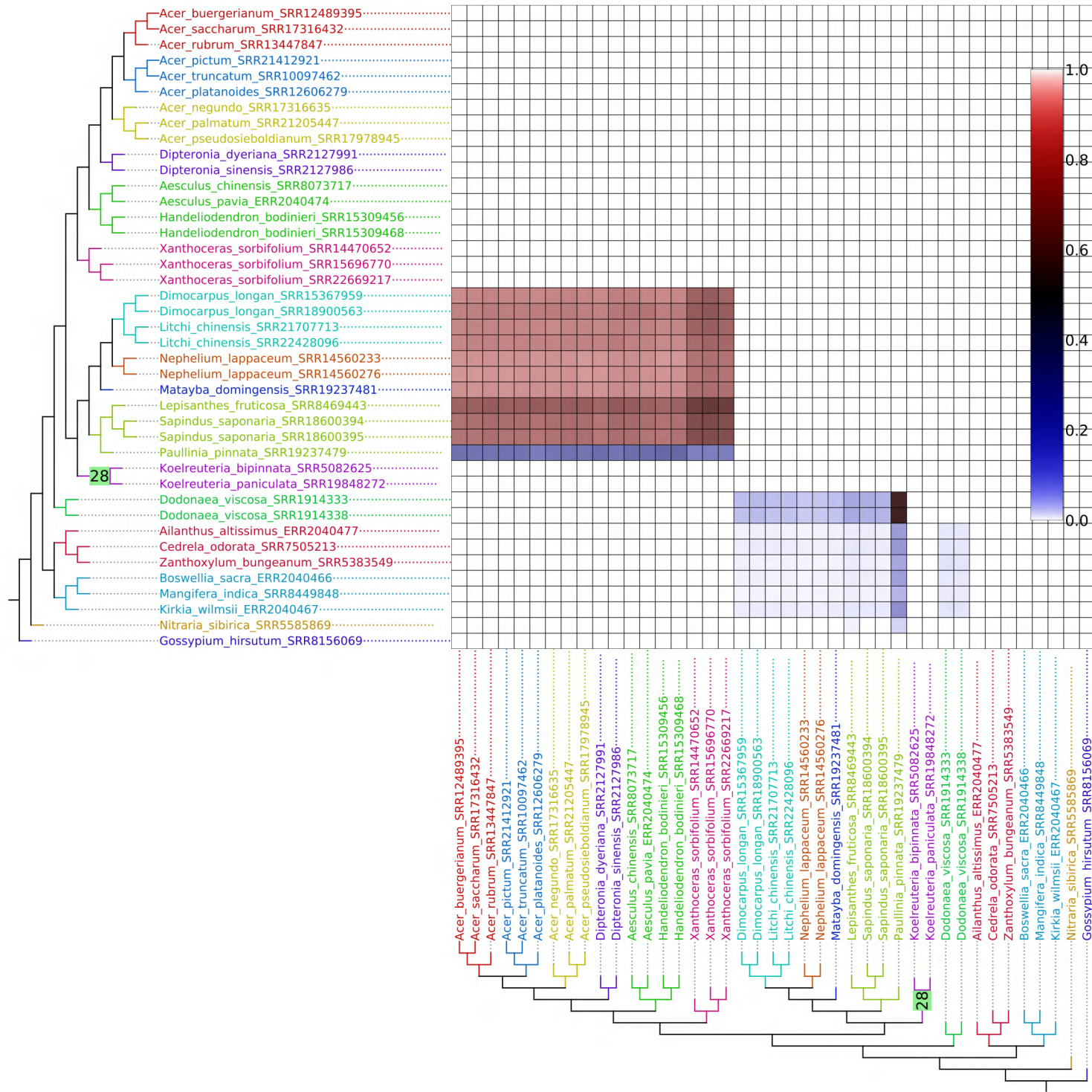

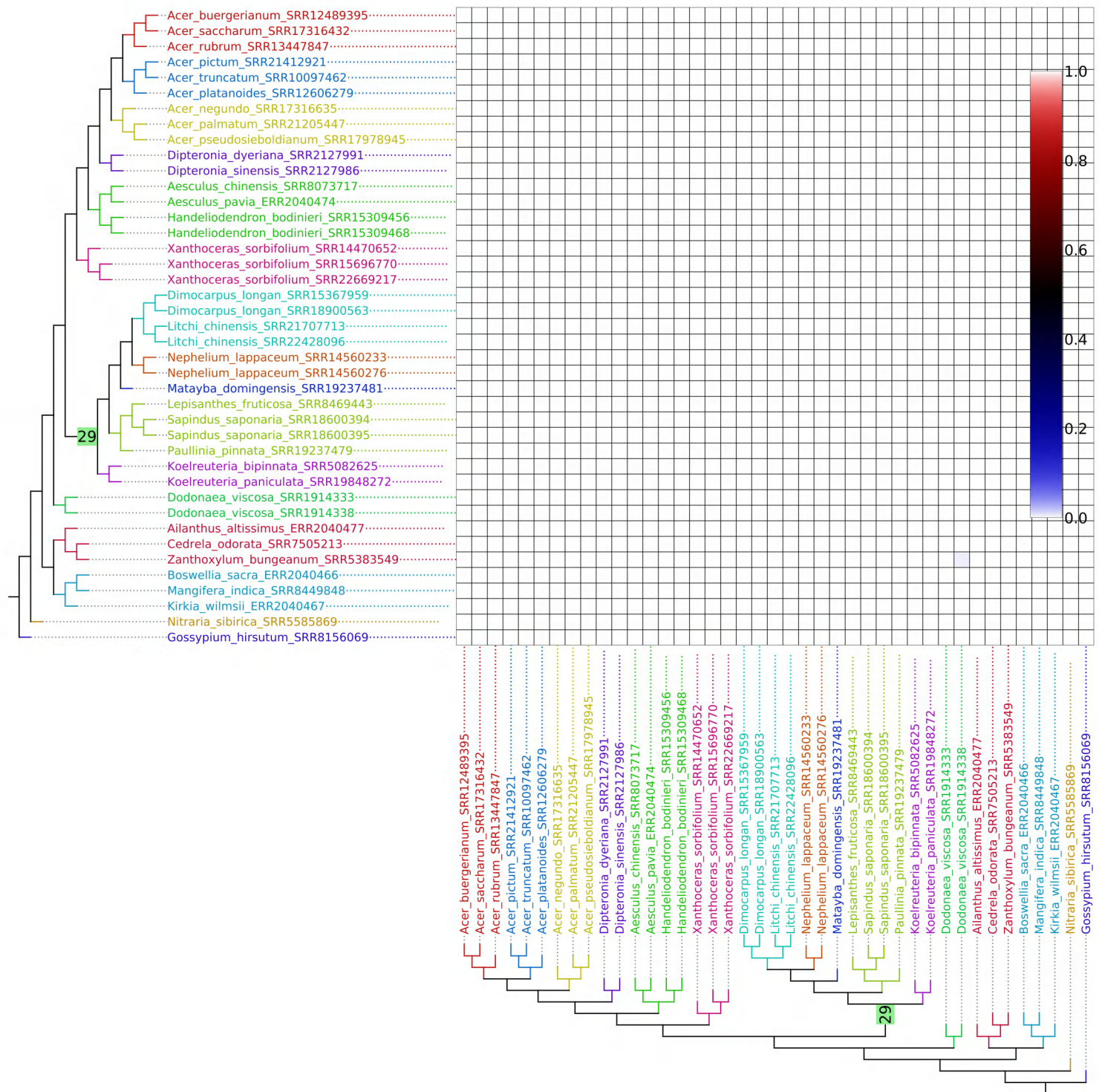

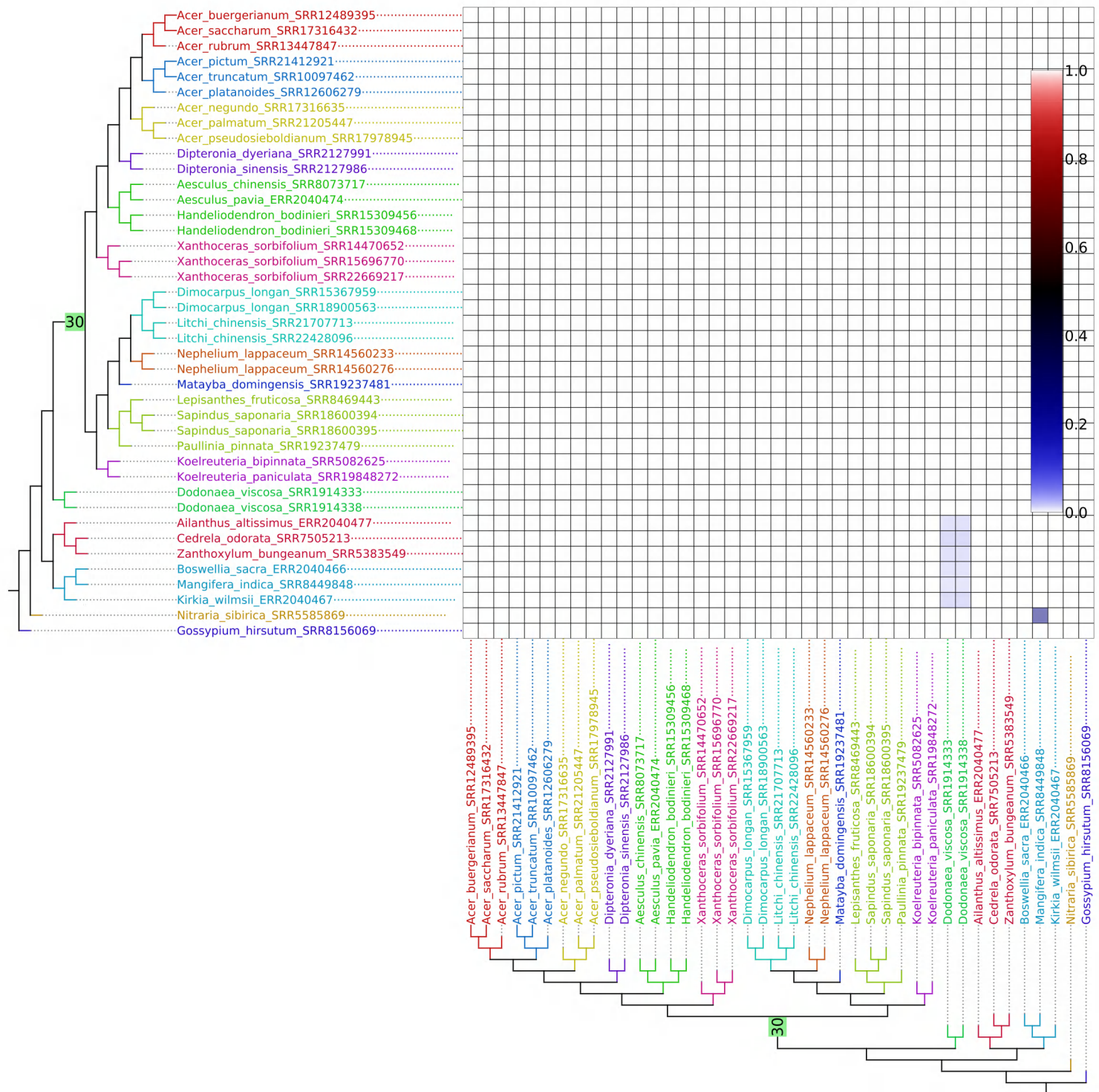

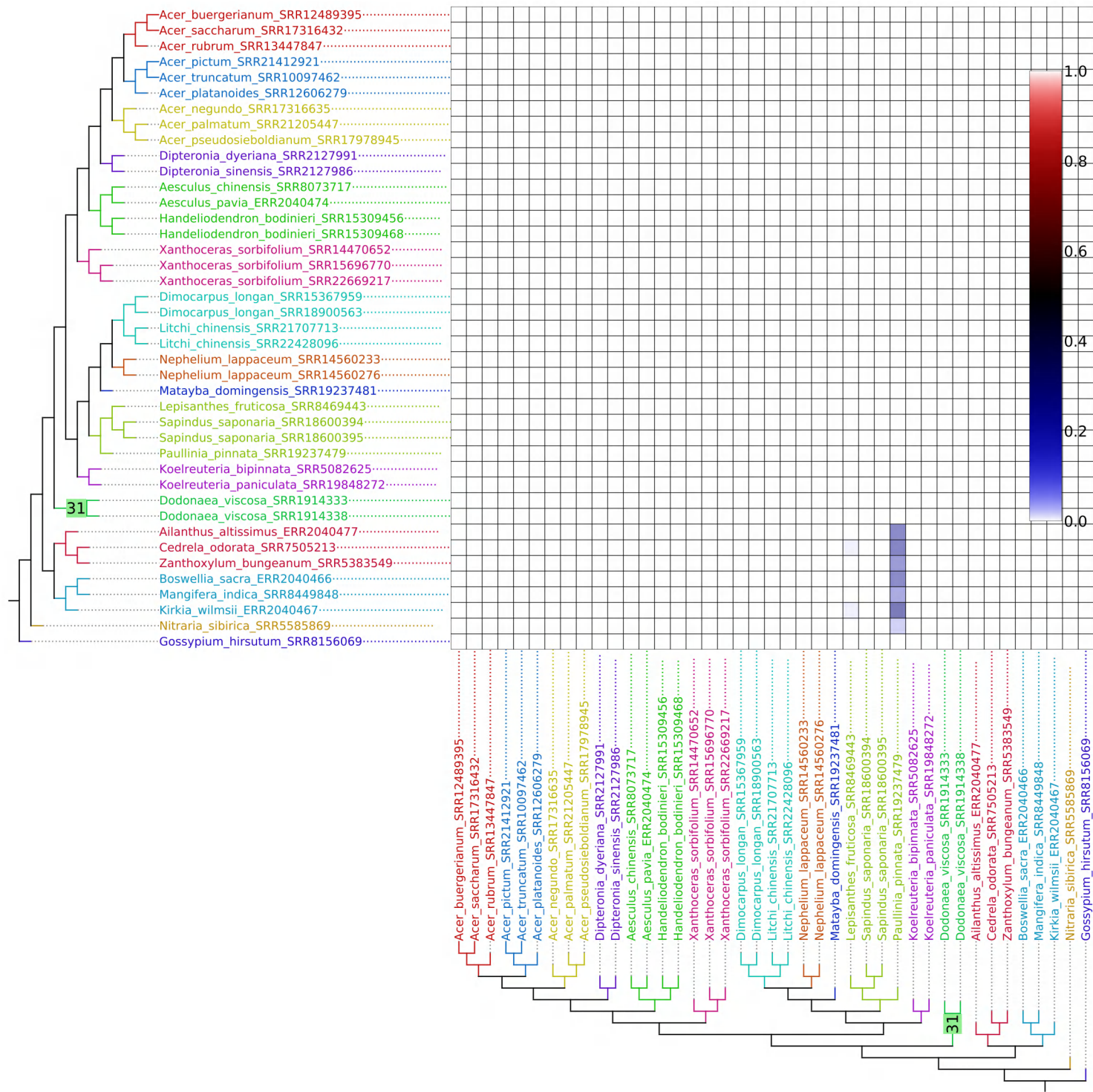

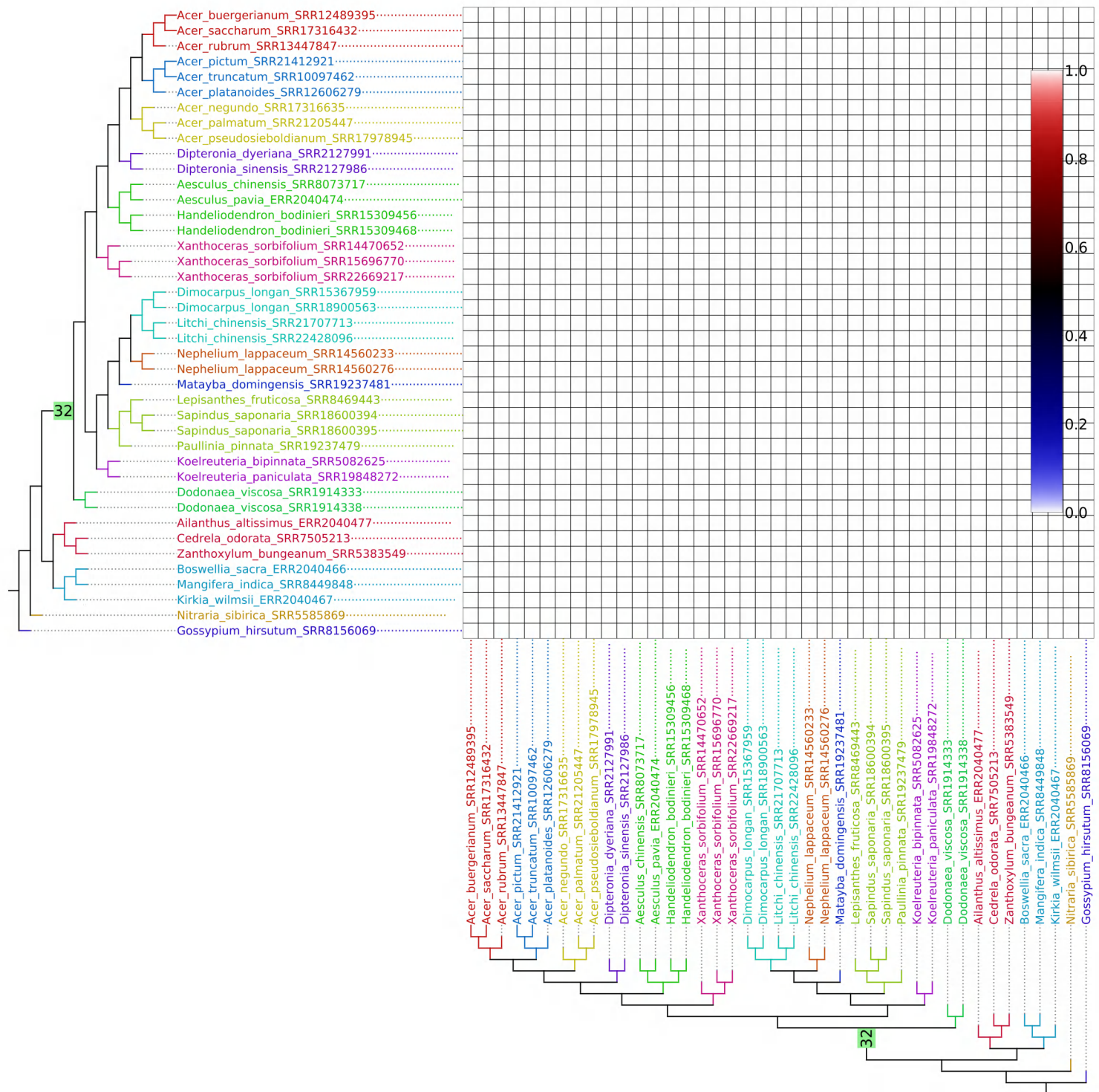

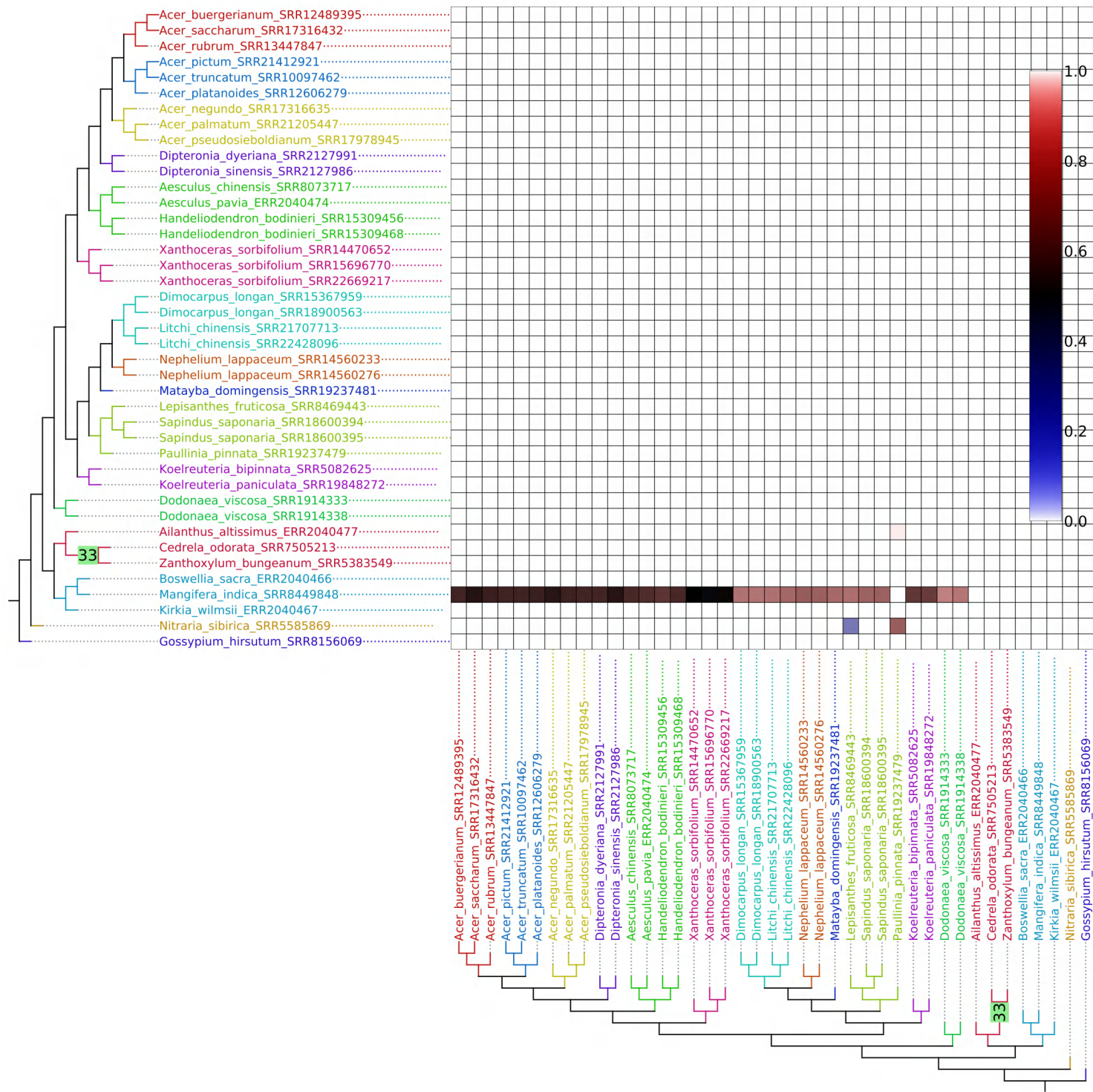

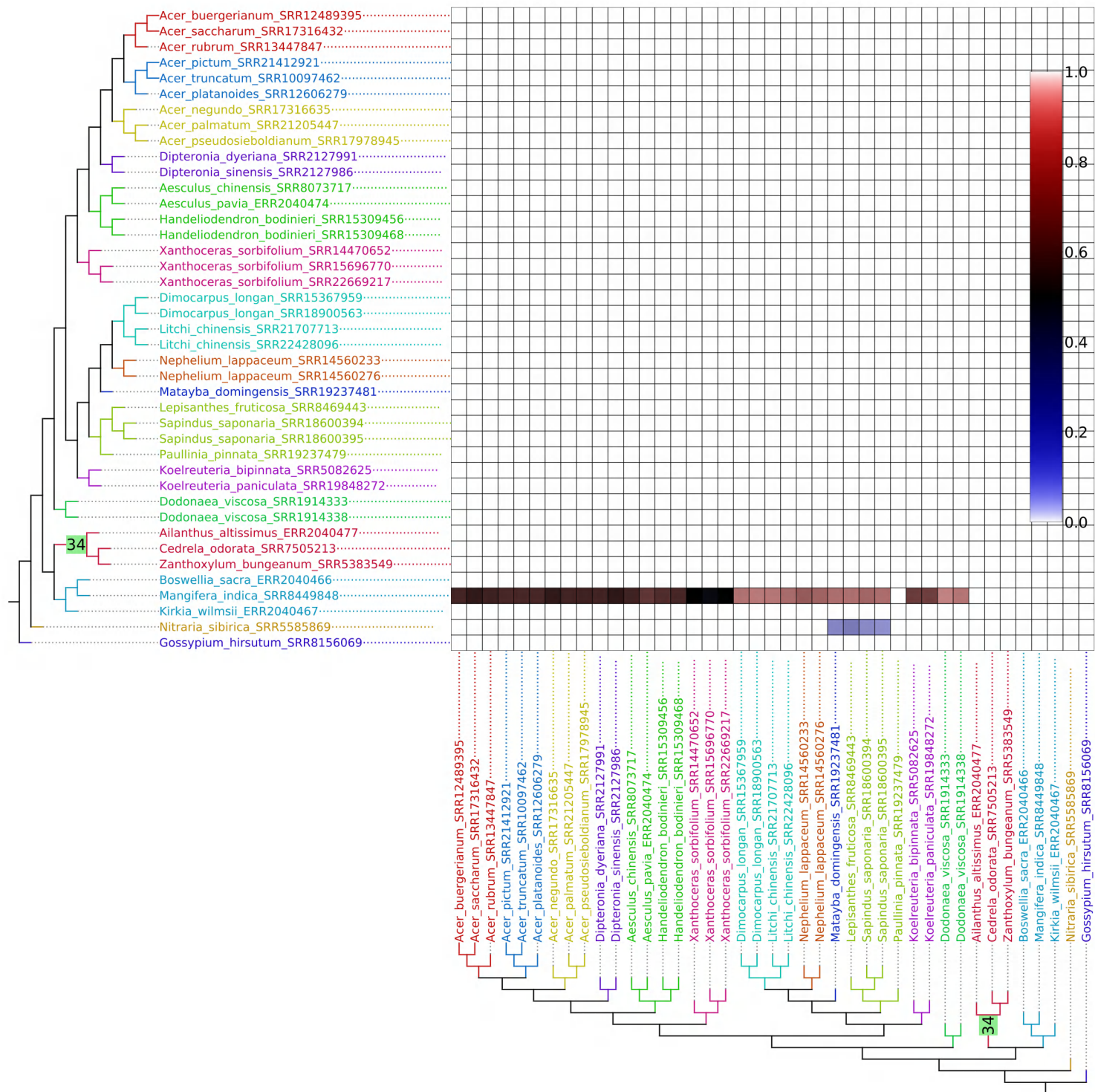

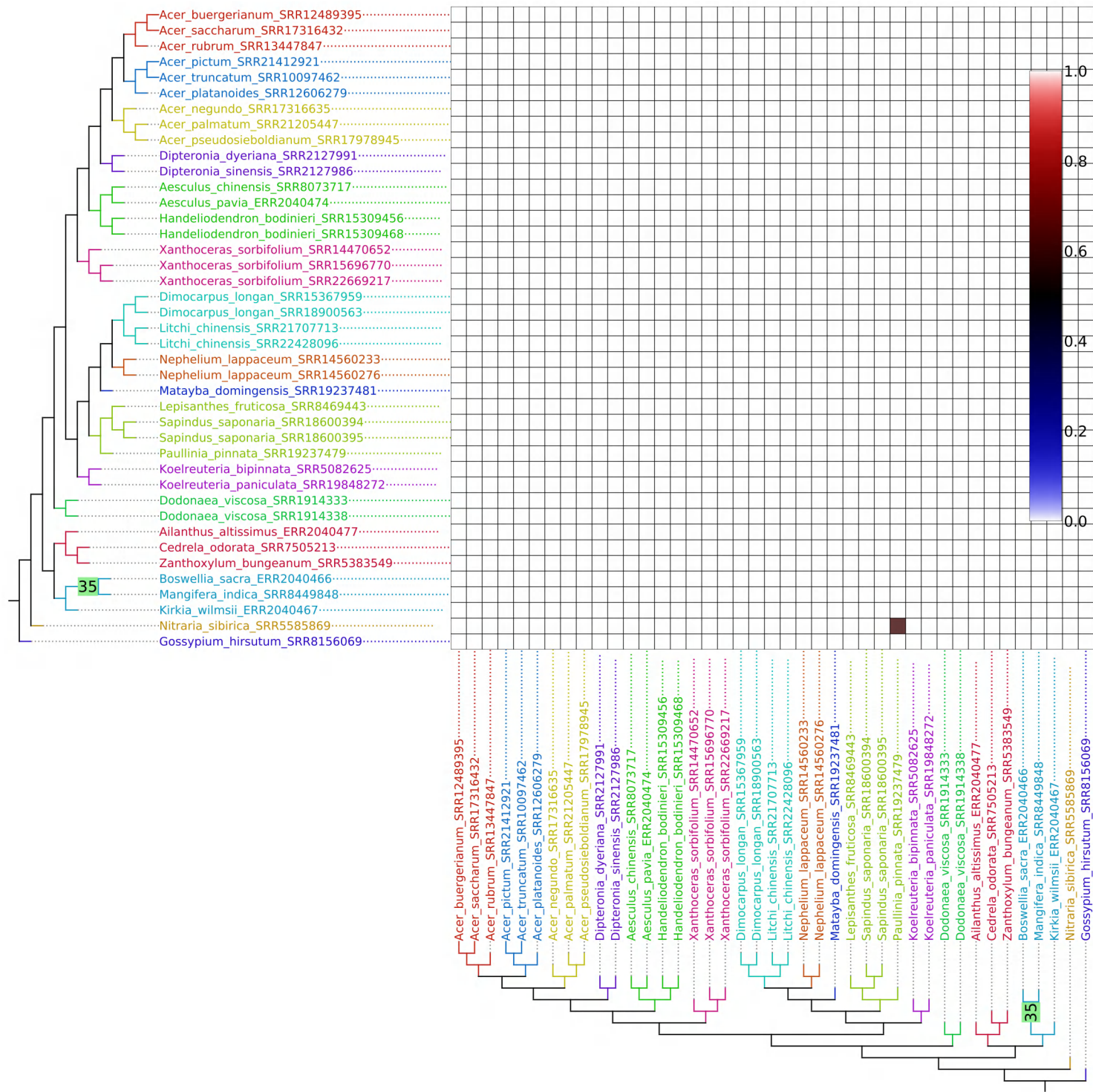

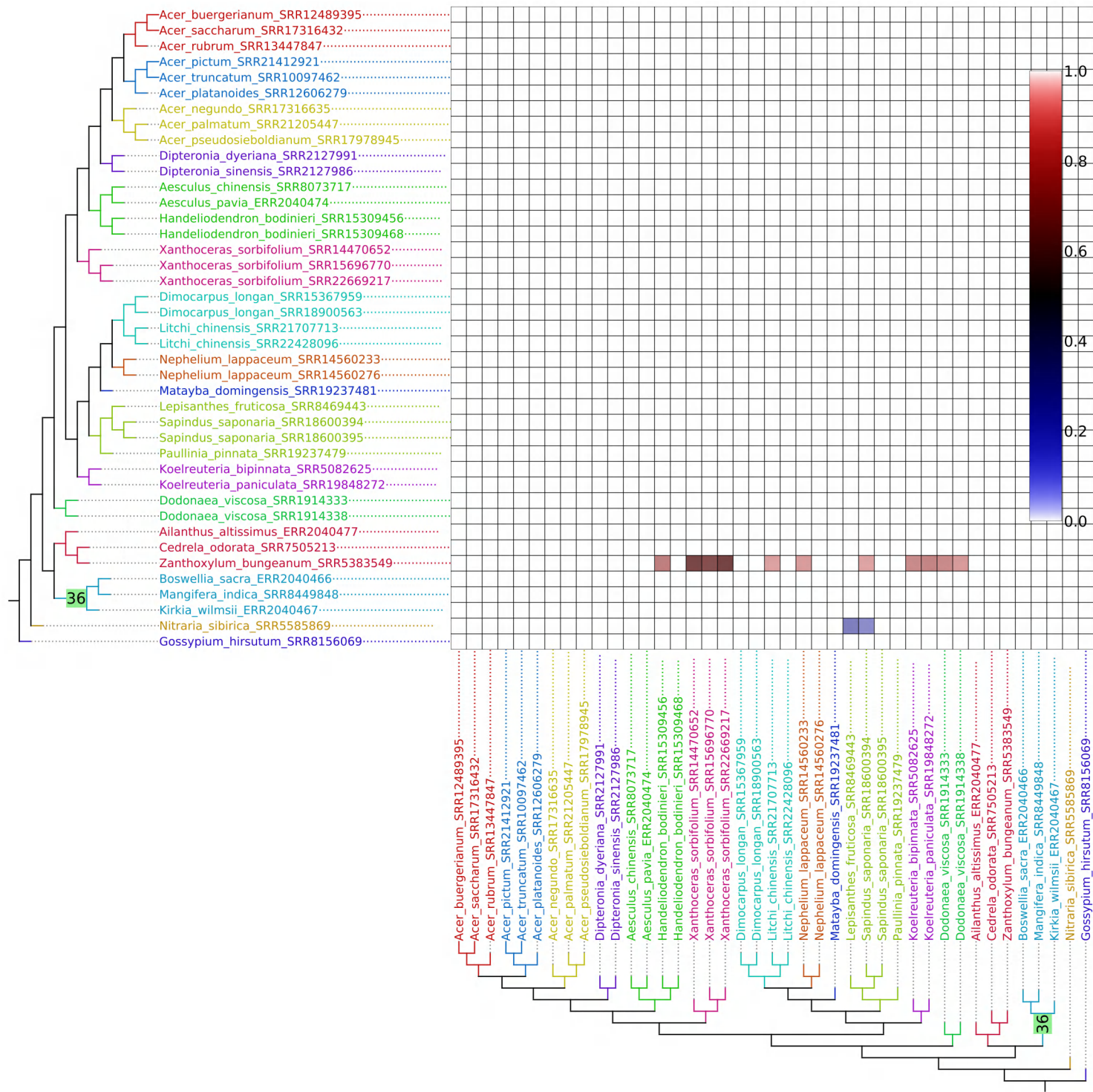

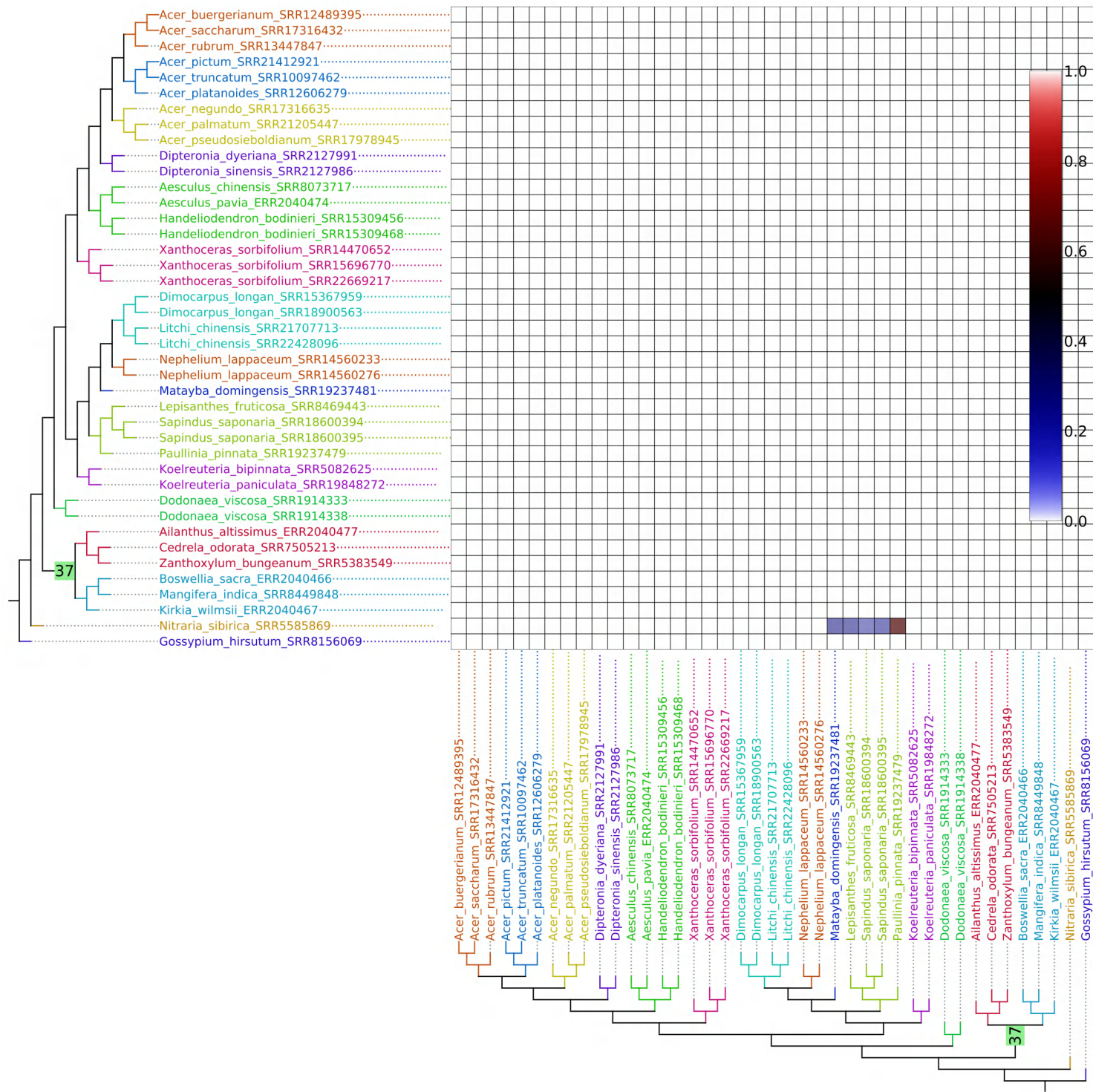

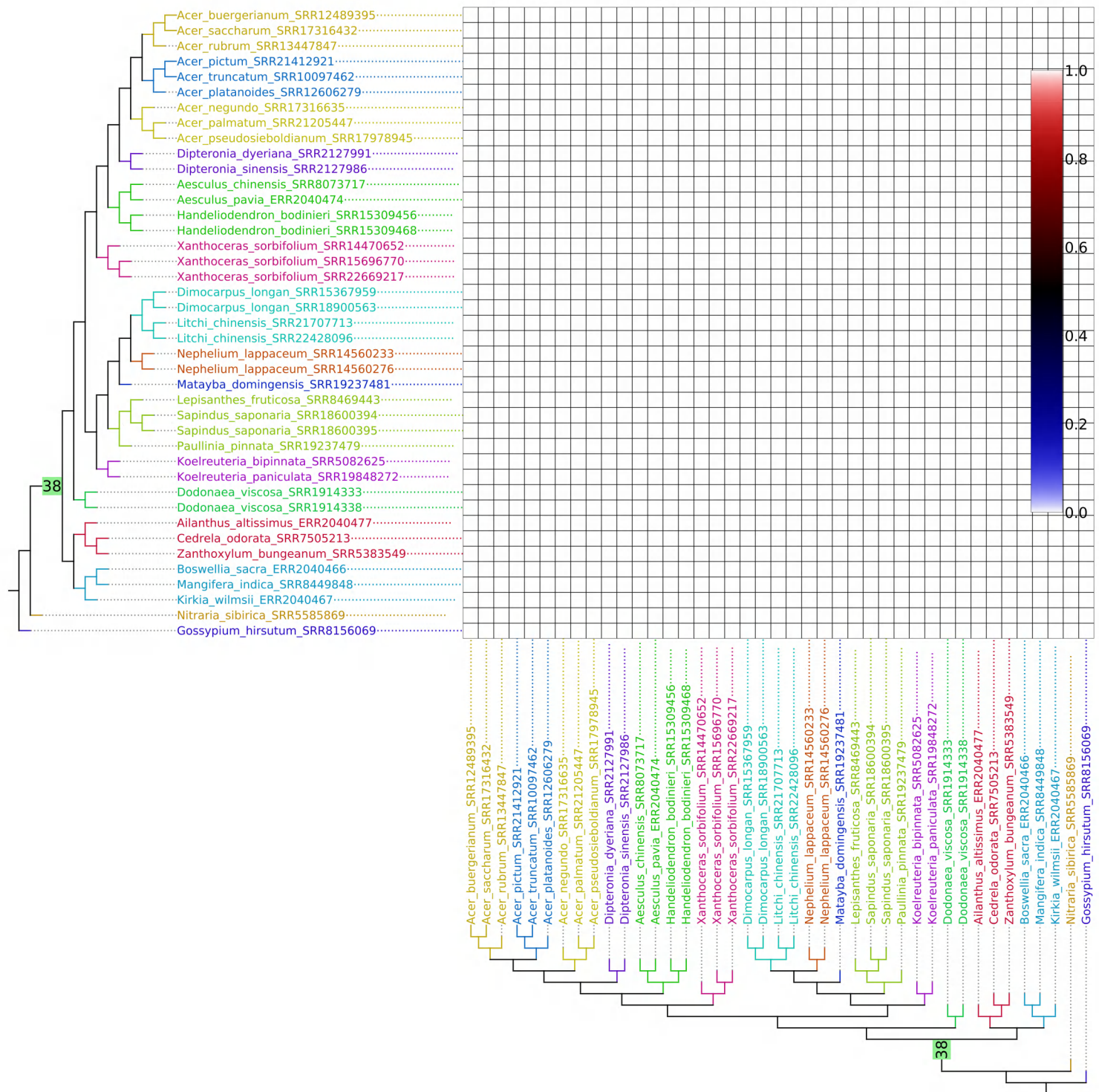

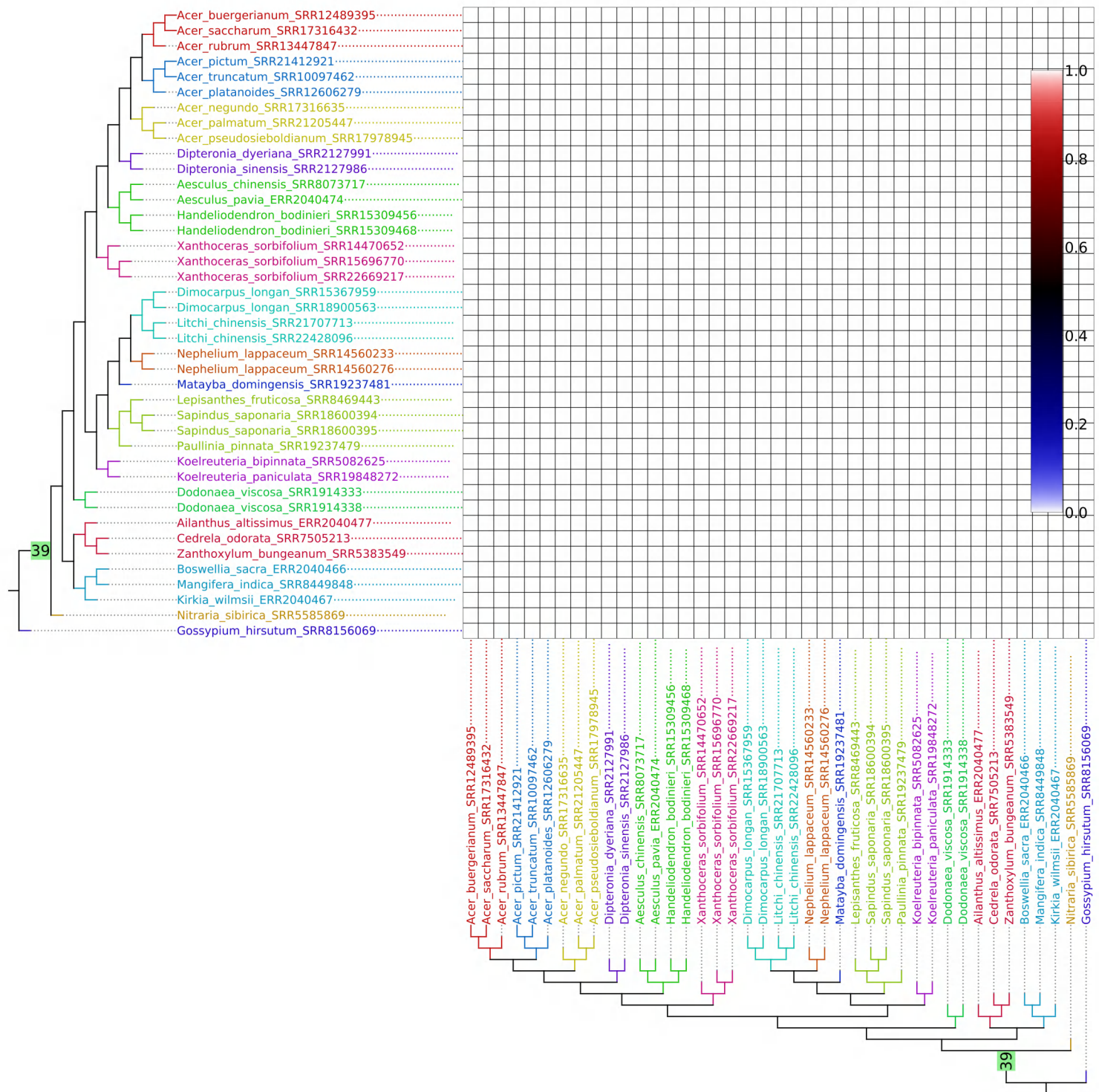

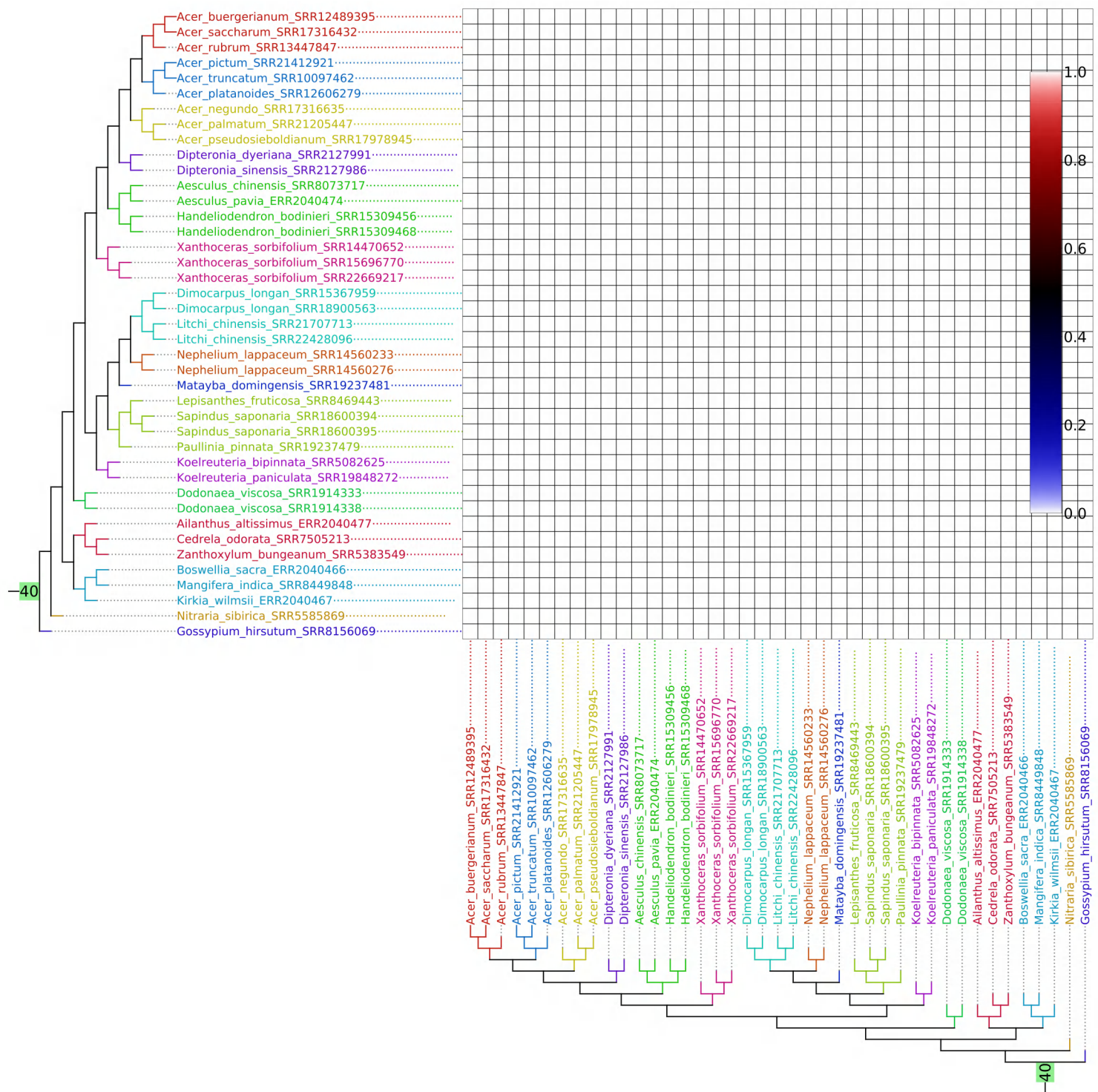

Supplement: Supplementary file 1 [file ijms-26-01581-s001.zip › Figure_S6.pdf]

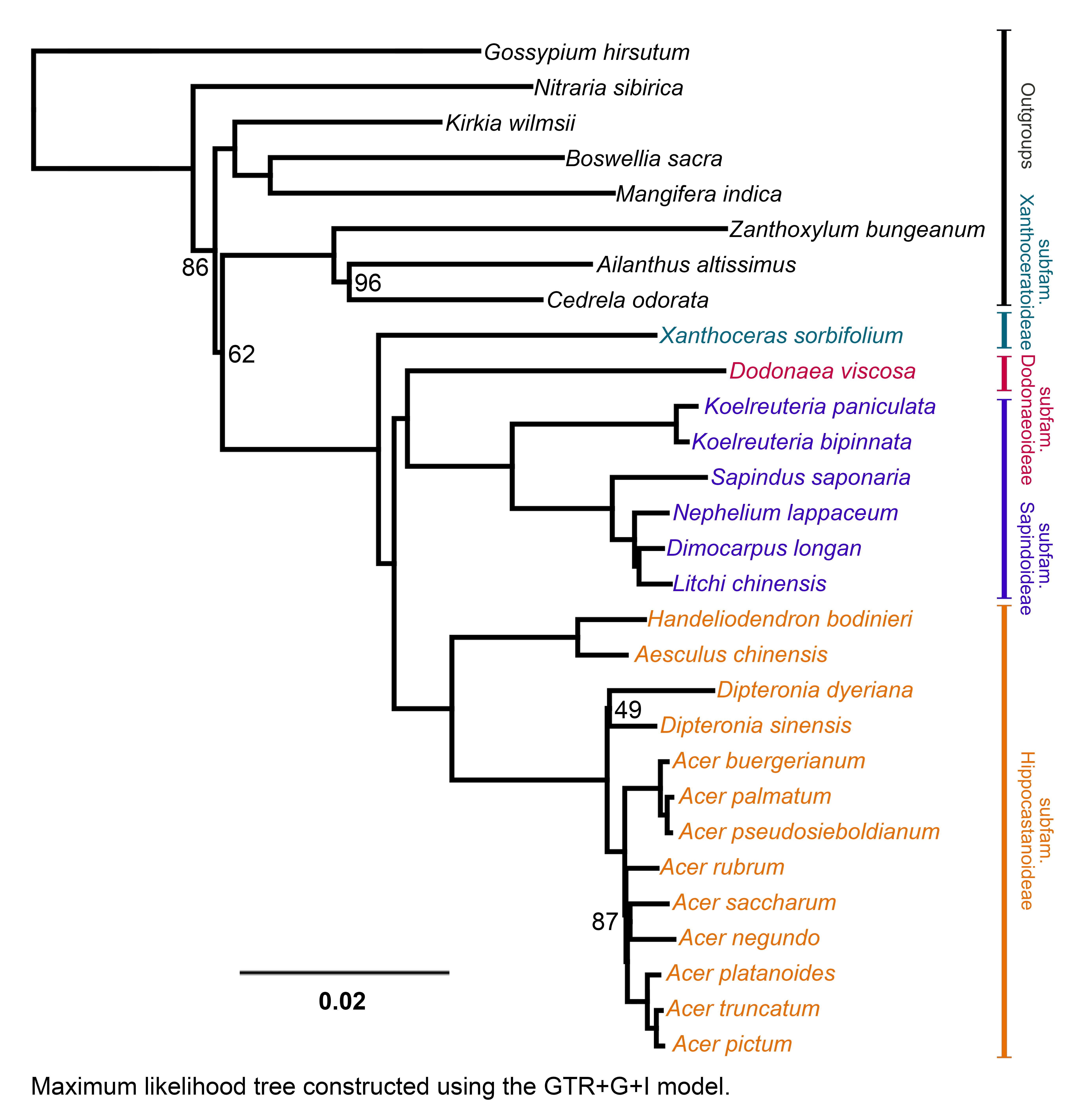

Supplement: Supplementary file 1 [file ijms-26-01581-s001.zip › Figure_S4.jpg]

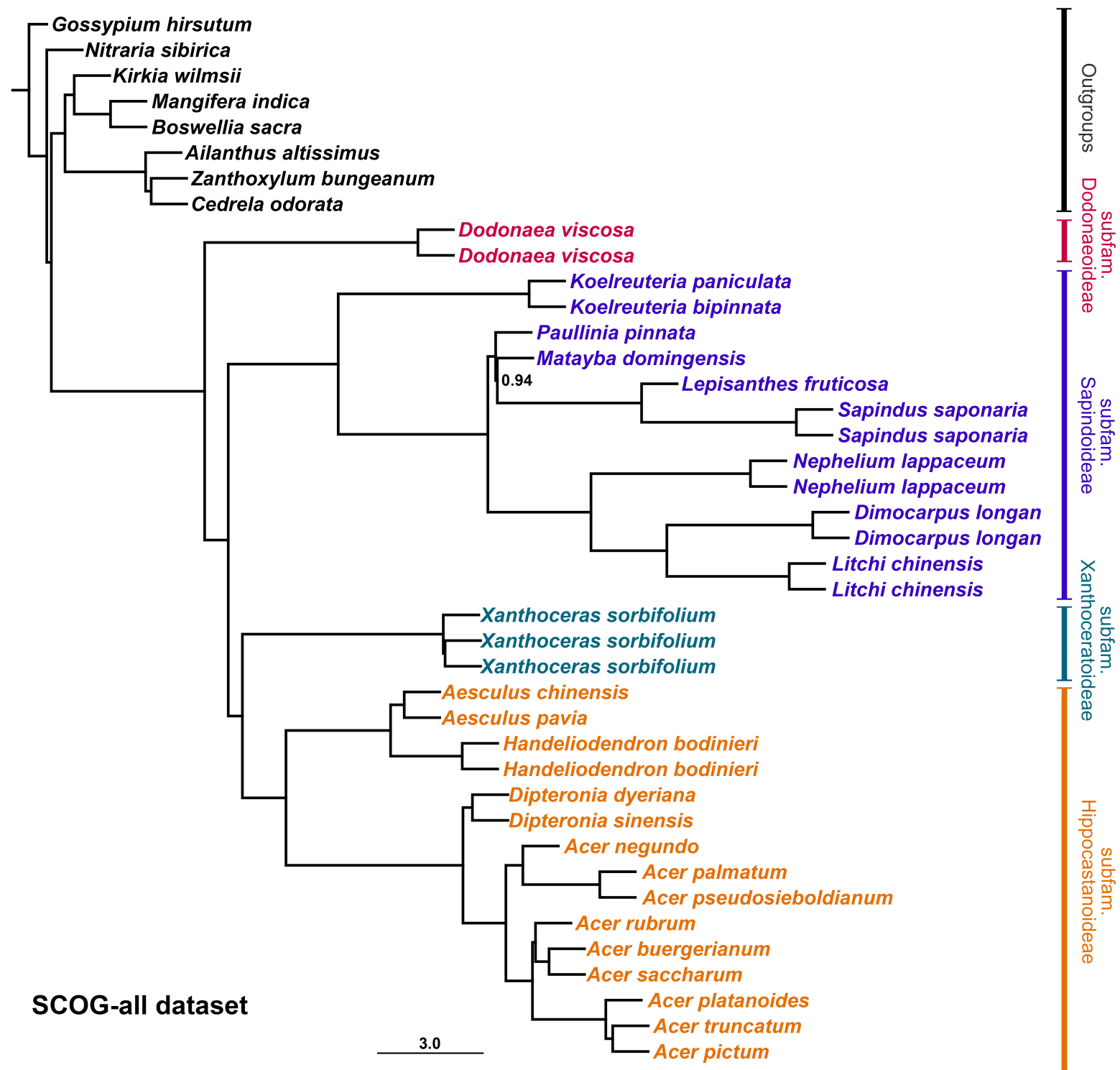

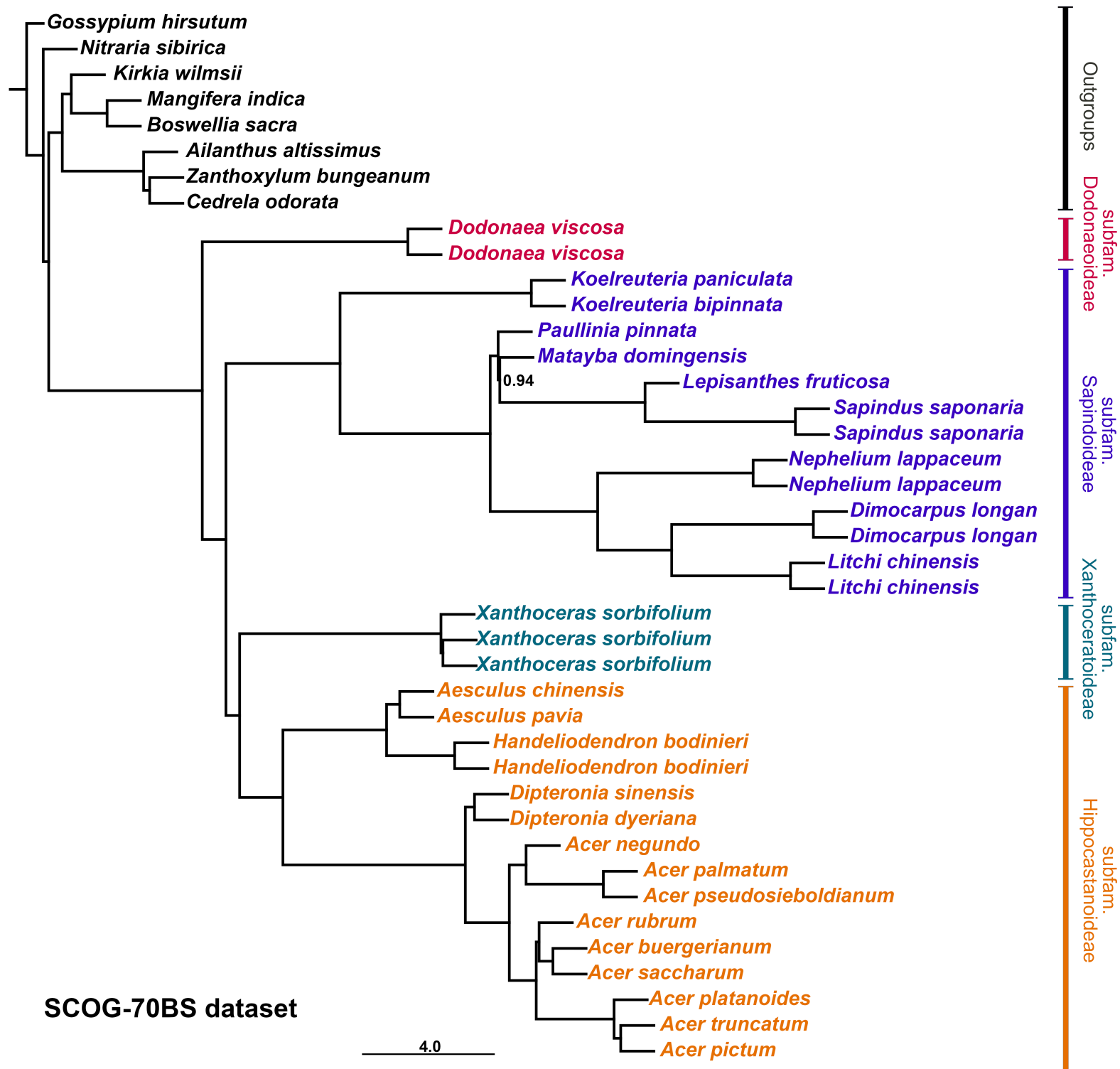

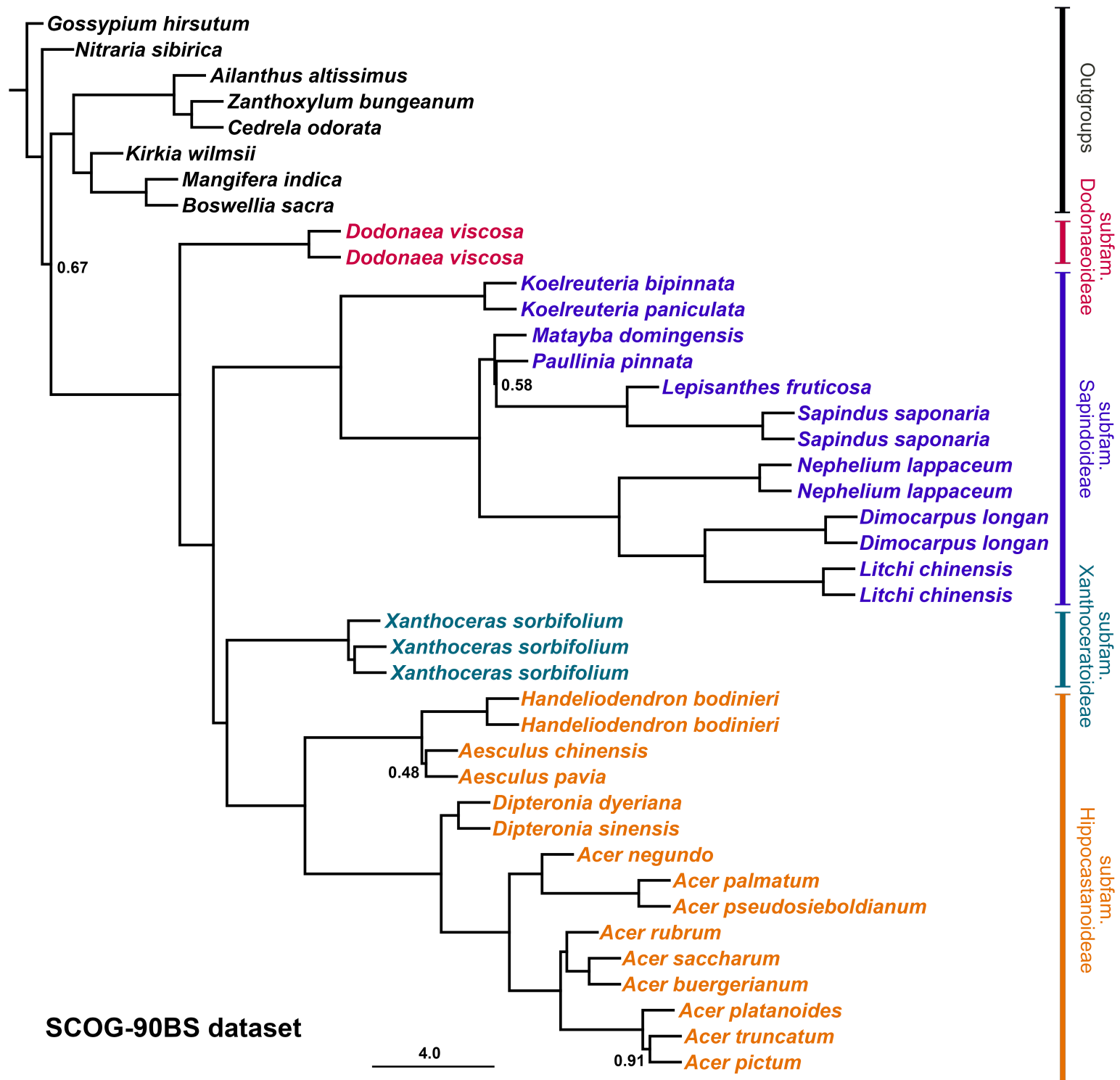

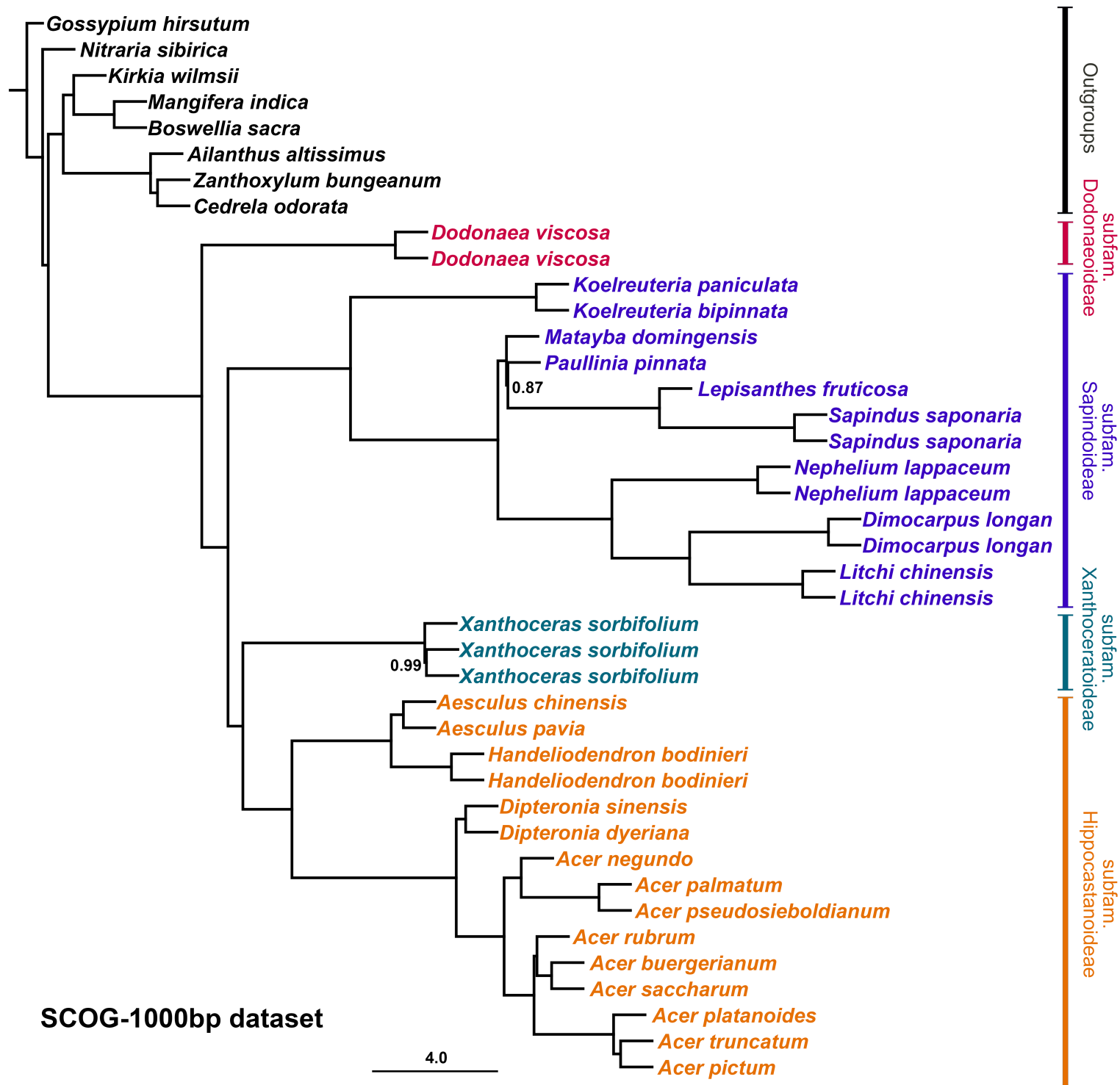

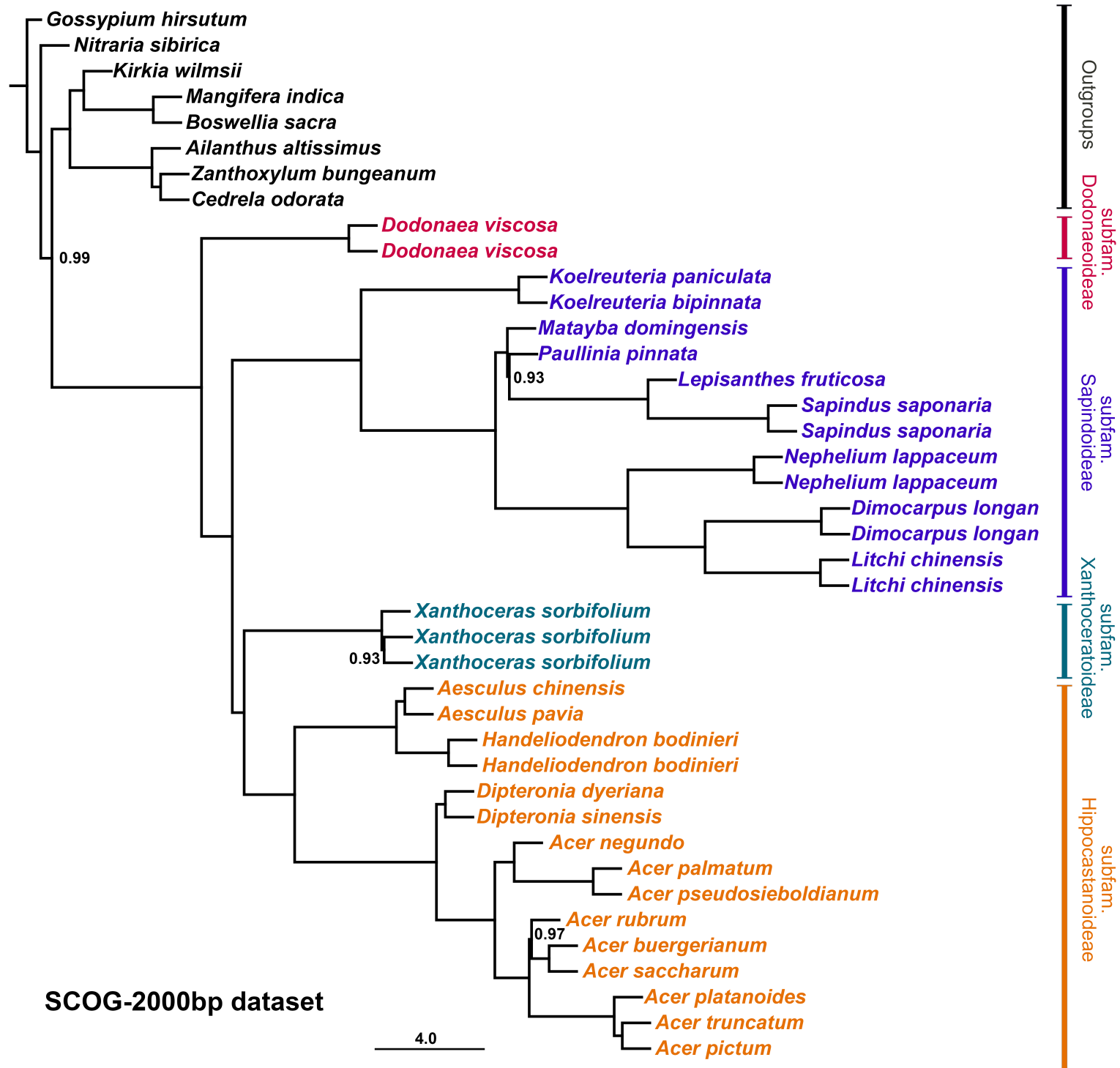

Supplement: Supplementary file 1 [file ijms-26-01581-s001.zip › Figure_S3.pdf]

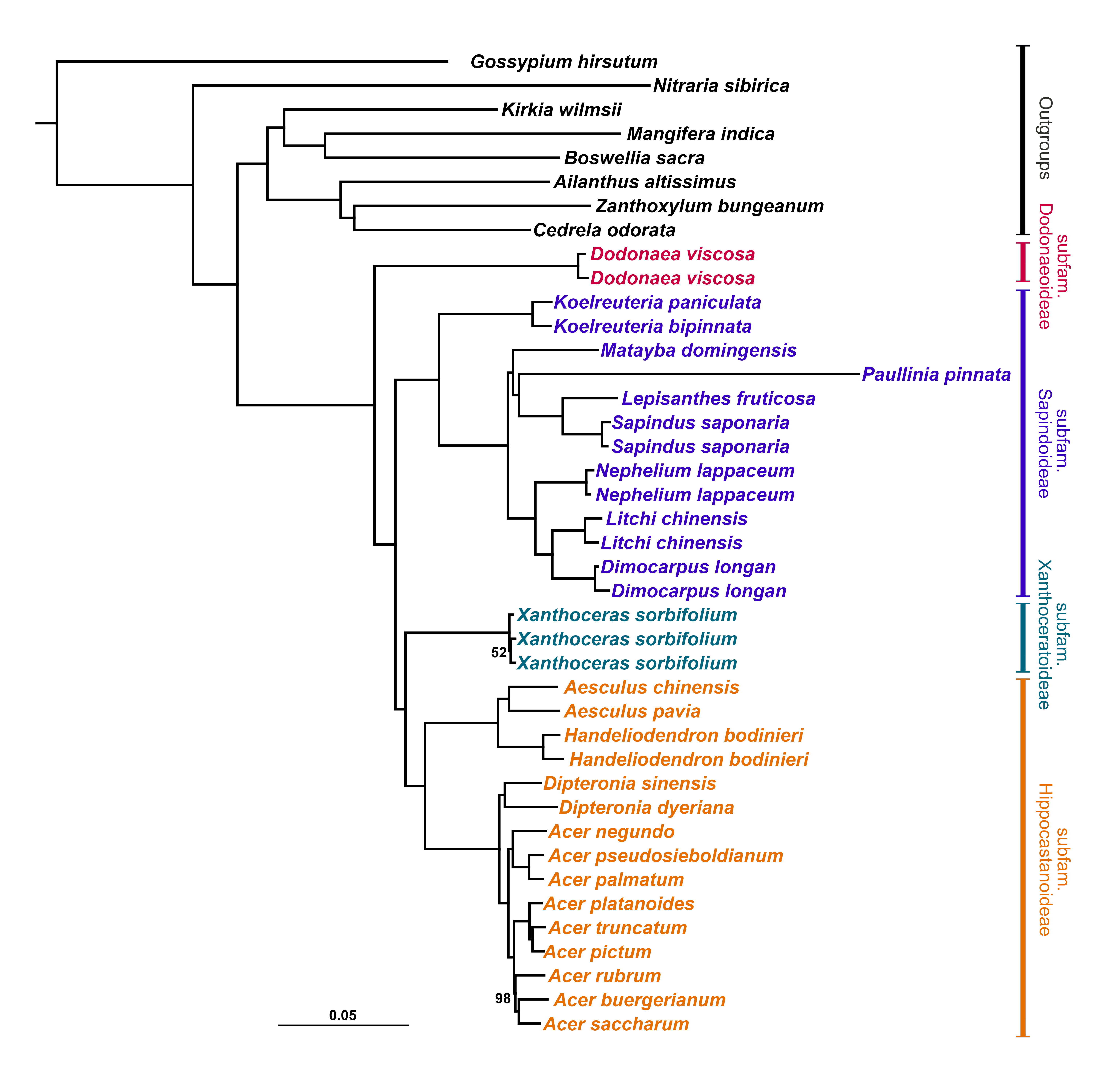

Supplement: Supplementary file 1 [file ijms-26-01581-s001.zip › Figure_S2.jpg]
